# A bi-directional Mendelian randomization study of sarcopenia-related traits and type 2 diabetes mellitus

**DOI:** 10.3389/fendo.2023.1109800

**Published:** 2023-03-08

**Authors:** Simin Chen, Shikang Yan, Nuerbiyamu Aiheti, Kaidiriyan Kuribanjiang, Xuemei Yao, Qian Wang, Tao Zhou, Lei Yang

**Affiliations:** ^1^ Department of Epidemiology and Health Statistics, School of Public Health, Xinjiang Medical University, Urumqi, Xinjiang, China; ^2^ State Key Laboratory of Causes and Prevention of High Morbidity in Central Asia jointly established by the Ministry and the Province Urumqi, Xinjiang, China

**Keywords:** type 2 diabetes mellitus, sarcopenia, mendelian randomization, hand-grip strength, appendicular lean mass, walking pace, aging

## Abstract

**Background:**

Previous studies have reported an association between sarcopenia and type 2 diabetes mellitus (T2DM), but causation was prone to confounding factors. A more robust research approach is urgently required to investigate the causal relationship between sarcopenia and T2DM.

**Methods:**

The bi-directional two-sample MR study was carried out in two stages: Sarcopenia-related traits were investigated as exposure while T2DM was investigated as an outcome in the first step, whereas the second step was reversed. The GWAS summary data for hand-grip strength (n = 256,523), appendicular lean mass (ALM, n = 450,243), and walking pace (n = 459,915) were obtained from the UK Biobank. T2DM data were obtained from one of the biggest case-control studies on diabetes (DIAGRAM; n = 180,834 cases and 492,191 controls), which was published in 2022. The inverse-variance weighted (IVW) approach was used to obtain MR estimates, and various sensitivity analysis was also performed.

**Results:**

Low hand-grip strength had a potential causal relationship with an increased incidence of T2DM (OR = 1.109; 95% CI, 1.008–1.222; *p* = 0.0350). T2DM risk was reduced by increasing ALM and walking pace: A 1 kg/m^2^ increase in ALM decreased the risk of T2DM by 10.2% (OR = 0.898; 95% CI, 0.830–0.952; *p* < 0.001). A 1 m/s increase in walking pace decreased the risk of T2DM by 90.0% (OR = 0.100; 95% CI, 0.053–0.186; *p* < 0.001). The relationship was bidirectional, with T2DM as a causative factor of sarcopenia-related traits (*p* < 0.05) except for ALM (β = 0.018; 95% CI, −0.008 to −0.044; *p* = 0.168).

**Conclusions:**

Hand-grip strength and T2DM had a potential bidirectional causal relationship, as did walking pace and T2DM. We suggest that sarcopenia and T2DM may mutually have a significant causal effect on each other.

## 1 Introduction

With the worldwide population aging, the incidence of sarcopenia and type 2 diabetes mellitus (T2DM) is rapidly increasing, which is related to a worse standard of living and higher mortality (1, 2). They have contributed to a serious global health problem, putting a heavy medical and economic burden on society (3). Sarcopenia and TDM are both age-related diseases with the same underlying pathophysiological mechanism (4, 5). People with sarcopenia have impaired glucose tolerance, and elevated blood insulin levels, and are at increased risk of T2DM. T2DM is characterized by insulin resistance and muscular metabolism, which may function as an accelerator for sarcopenia. Based on observational research, significant lines of evidence quantitatively revealed a tight association between sarcopenia and T2DM. The prevalence of sarcopenia among the elderly with T2DM ranges from 15.7–29.3 percent (6, 7). A cohort study of 6895 adults with an average age of 52 years reported that people with a muscle mass index in the lowest third of the population were twice as likely to get T2DM as those in the control group (8). These results make it difficult for researchers to determine which condition causes the other, particularly when confounders such as age, gender, and lifestyle are present (5). Assessing the causal relationship between sarcopenia and T2DM may help in the development of new techniques for the prevention, diagnosis, and treatment of T2DM.

Mendelian randomization (MR) investigates the causal effects associated with exposure and outcome by using genetic variants as instrumental variables (IVs), which avoids confounding variables and reverse causation (9, 10). To the authors’ knowledge, no MR between sarcopenia and T2DM has been studied. Moreover, no randomized controlled study (RCT) specifically evaluated the bi-directional relationship. Therefore, we used a bi-directional two-sample MR analysis to investigate the links between sarcopenia (measured as hand-grip strength, ALM, and walking pace) and T2DM.

## 2 Materials and methods

### 2.1 Study design

**Figure 1:**
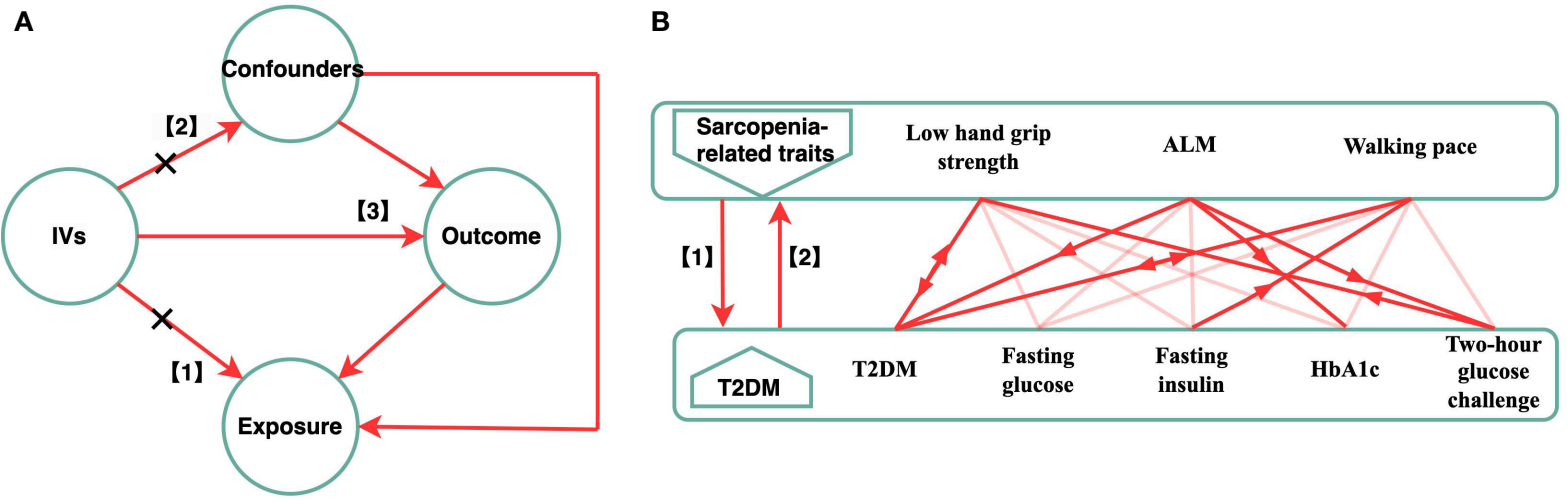
Schematic diagram of the Bi-directional MR Analysis. **(A)** illustrates three assumptions of MR analysis as follows: 1) the IVs have a significant correlation with the exposure; 2) the IVs also had no pleiotropic correlations with any known confounding factors; and 3) the IVs have no connection with the outcome, with the possible exception of how that link is mediated by their association with the exposure. **(B)** This bidirectional MR analysis was performed in two steps: sarcopenia-related traits (hand grip strength, ALM, walking speed) was studied as exposure while T2DM and glycemic characteristics were studied as outcome in the first step, whereas the second step was reversed. The arrows indicate direction of causality in our results. MR, mendelian randomization; IVs, instrumental variables; T2DM, Type 2 diabetes mellitus; ALM, appendicular lean mass.

In this study, we adopted a bi-directional MR study design, employing two-Sample MR methodologies and different GWAS summary level data sets to explain the causation and causative direction between the sarcopenia-related traits (hand-grip strength, appendicular lean mass, walking pace) and the risk of T2DM and glycemic traits (fasting insulin, fasting blood glucose, glycated hemoglobin, and two-hour blood glucose challenge) in a European population. This study consisted of two stages. In first, we investigated whether sarcopenia-related traits were causally related to T2DM and glycemic characteristics. In the second, we evaluated whether genetic T2DM and glycemic characteristics were linked to sarcopenia-related traits. (**Figure 1B**).

### 2.2 Data source

The open GWAS database established by the MRC Integrated Epidemiology Unit (IEU) (https://gwasmrcieu.ac.uk/) provided the majority of the summary-level data used in this investigation (11). The datasets are largely public and may be downloaded using R and Python packages for accessing the application programming interface. **Table 1** shows the phenotypic and consortium details.

**Table 1:** Phenotype source and description.

Phenotype	Consortium	Participants	Datatype	GWAS ID	Year of publication
Low hand grip strength (EWGSOP)	UKB	n = 48,596 cases and 207,927 controls	Binary	ebi-a-GCST90007526	2021
ALM	UKB	450,243	Continuous	ebi-a-GCST90000025	2020
Walking pace	UKB	459,915	Continuous	ukb-b-4711	2018
T2DM	DIAMANTE	n = 180,834 cases and 492,191 controls	Binary	NA	2022
Fasting glucose	MAGIC	200,622	Continuous	ebi-a-GCST90002232	2021
Fasting insulin	MAGIC	151,013	Continuous	ebi-a-GCST90002238	2021
HbA1c	MAGIC	46,368	Continuous	ieu-b-103	2010
Two-hour glucose challenge	MAGIC	15,234	Continuous	ebi-a-GCST000569	2010

T2DM, Type 2 diabetes mellitus; ALM, appendicular lean mass; HbA1c, glycated hemoglobin; EWGSOP, European Working Group on Sarcopenia in Older People.

#### 2.2.1 Source of exposure data

The GWAS summary data for hand-grip strength were obtained from a meta-analysis of 256,523 people of European origin ≥ 60 years of age and in 22 cohorts (12). Based on hand-grip strength using the European Working Group on Sarcopenia in Older People’s (EWGSOP) definition of 30 kg for men and 20 kg for women, 46,596 (18.9%) of all participants reported muscular weakness. We collected GWAS summary data from the UK Biobank for appendicular lean mass (ALM) in 450,243 people and walking pace in 459,915 people who were included in the discovery cohort (13). The UK Biobank is an expansive biomedical database and a research resource (14). It includes in-depth information on the genetic makeup and health of about half a million people between 40 and 69 years of age who participated in the study in the UK. Genetic association studies (GWAS) on sarcopenia-related variables were undertaken in European populations using data from population-based cohorts.

#### 2.2.2 Source of outcome data

In this study, the instrument for the outcome (T2DM) was received from the DIAMANTE consortium and from the summary statistics of a GWAS meta-analysis. The GWAS meta-analysis comprised 22 GWAS that involved 180,834 patients with type 2 diabetes and 492,191 controls and five pedigree groups (51.1% of European ancestry). The GWAS database of European ancestors was released by Mahajan et al. in 2022 (15). Summary data for fasting glucose, fasting insulin, glycated hemoglobin (HbA1c), and 2-hour glucose challenge were collected from an online source (https://gwas.mrcieu.ac.uk/) under the GWAS IDs ebi-a-GCST90002232 (fasting glucose, N = 200,622), ebi-a-GCST90002238 (fasting insulin, N =151), ieu-b-103 (HbA1C, N =46,368) and ebi-a-GCST000569 (two-hour glucose challenge, N = 15,234) (11).

### 2.3 IV selection and validation

Under the foundational premise of MR, specific SNPs for each exposure attribute were chosen as IVs. The IVs med three assumptions (**Figure 1A**). (1) The IVs had a significant relationship with the exposure. (2) The IVs had no pleiotropic correlations with any known confounding factors, and (3) had no association with the outcome, with the possible exception of how the link was mediated by their association with the exposure (16, 17). We obtained the IVs from the published GWAS using the clumping function in the Two-Sample MR package. Except for hand-grip strength, HbA1c, and two-hour glucose challenge (*p* < 5 × 10^-6^), all IV and exposure traits were significantly (*p* < 5 × 10^-8^) and independently [chain disequilibrium (LD) r^2^ < 0.01) associated (18). Following the guidelines, we first chose several independent SNPs strongly related to each exposure variable before matching them in the outcome database. During the harmonization process, the harmonies data function from the two-sample MR package was used so the effect allele of each SNP could be matched with the corresponding allele of the exposure. To correct for multiple comparisons, the Bonferroni method was utilized (five outcomes). The associations with *p*-values < 0.003 (0.05/15) were regarded as statistically significant associations, and associations with *p*-values between 0.05 and 0.003 were considered as suggestive associations.

### 2.4 Analysis of horizontal pleiotropy and heterogeneity

Causal estimates and findings may be skewed due to the pleiotropy of SNPs in the IVW study (19). We used the pleiotropy test function with the two-sample MR package to determine whether the SNPs were suitable IVs. If the pleiotropy was not significant (*p* > 0.05), then it was possible to use the IVs. We selected SNPs one by one on the Phenoscanner website (http://www.phenoscanner.medschl.cam.ac.uk/) if the *p*-value of the pleiotropy test was < 0.05 (20). Cochran’s Q test was used to approximate heterogeneity among the chosen IVs. The fixed-effects model was used if the heterogeneity was negligible. Otherwise, the IVW with random effects approach was acceptable.

### 2.5 Analysis of Mendelian randomization

Mendelian randomization with genetic variations as the IVs allows investigation and assessment of the causal influence of an exposure on an outcome. First, we divided the per-allele log-odds ratio (or beta) of each variation in the outcome data by the log-odds ratio (or beta) of the same variant in the exposure data to get the Wald ratio for each IV. After that, the inverse variance weighted (IVW) approach was used to arrive at an estimation of the connection between the exposures and the results. IVW weights the Wald ratio of each SNP by its inverse variance and meta-analysis impact estimates using random or fixed effects. If the *p*-value of the Cochran Q test was < 0.05, we used the use of random-effect models; otherwise, we relied on fixed-effect models. To complement the outcome of IVW, MR-Egger and the weighted median approach were used (21).

### 2.6 Sensitivity analysis

To further guarantee the reliability of the MR causal impact estimate, we performed sensitivity analyses (22). To start, the MR-Egger approach was used to determine whether SNPs had horizontal pleiotropy (23). If the *p*-value of the intercept term was < 0.05, then the IVs may have pleiotropic effects. But there is no evidence of horizontal pleiotropy across the chosen IVs if the *p*-value of the intercept term is not < 0.05. Second, the standard MR testing outliers were identified with Mendelian Randomization Pleiotropy Residual Sum and Outlier (MR-PRESSO) test, which produced a robust estimate after outlier correction. A sensitivity analysis was performed to test for substantial distortions in the IVW causal estimate before and after MR-PRESSO adjustment. In this study, random-effects IVW was replicated by taking removing individual SNPs and then comparing the total analysis. The sensitivity of each SNP was inferred from how much the findings changed before and after it was removed.

### 2.7 Reported results and software

MR findings were reported as odd ratios (ORs) with 95% confidence intervals (CIs) per standard deviation for dichotomous variables, and as beta values (β) with 95% CIs per standard deviation for continuous variables. Both were stated consistently as estimate values. A two-tailed *p*-value of 0.05 was the threshold of statistical significance. The statistical analysis was conducted with two-sample MR and MR-PRESSO packages of R version 4.0.0 (24).

## 3 Results

### 3.1 Stage 1: Effect of genetically predicted sarcopenia-related traits on T2DM and glycemic traits

Screening of the IVs found SNP beta, SE, and *p*-values strongly related to exposure. We found the beta, SE, and p-value corresponding to the SNP in the outcome database. **Supplementary Tables 1-3** has more information about IVs. For low hand-grip strength, ALM, and walking pace, we found 59, 690, and 57 LD-independent (r^2^ < 0.001) IVs that reached genome-wide significance (*p* < 5 × 10^−8^), except low hand-grip strength (*p* < 5 × 10^−6^). The horizontal pleiotropy test findings revealed that all IVs lacked horizontal pleiotropy (*p* > 0.05). One IV groups were not heterogeneous (*p* > 0.05). The IVW random-effect model for IV with heterogeneity was used; otherwise, the IVW fixed-effect model was used. The level pleiotropy and heterogeneity test results are shown in **Table 2**.

**Table 2:** Association sarcopenia-related traits with T2DM and glycaemic traits using heterogeneity test and pleiotropy test.

Exposures	Outcomes	No.of Ivs	Heterogeneity test		Pleiotropy_test
			Cochran's Q	*P*	*P*
Low hand grip strength (EWGSOP)	T2DM	52	225.3713	<0.001	0.377
	Fasting glucose	56	108.4448	<0.001	0.244
	Fasting insulin	56	95.9703	<0.001	0.661
	HbA1c	30	38.4647	0.112	0.675
	Two-hour glucose challenge	33	46.5341	0.047	0.305
ALM	T2DM	552	3214.1050	<0.001	0.289
	Fasting insulin	372	981.8712	<0.001	0.424
	Fasting glucose	617	1635.6020	<0.001	0.998
	HbA1c	362	474.1836	<0.001	0.903
	Two-hour glucose challenge	401	453.5987	0.033	0.886
Walking pace	T2DM	56	496.6391	<0.001	0.284
	Fasting glucose	56	112.1423	<0.001	0.962
	Fasting insulin	56	125.2184	<0.001	0.268
	HbA1c	43	59.7183	0.037	0.745
	Two-hour glucose challenge	46	67.2943	0.017	0.473

T2DM, Type 2 diabetes mellitus; ALM, appendicular lean mass; HbA1c, glycated hemoglobin; EWGSOP, European Working Group on Sarcopenia in Older People.

The findings of this MR study are mostly concerned with IVW analysis. Our findings reveal that sarcopenia-related features were all linked to T2DM. Low hand-grip strength had a potential causal relationship with an increased incidence of T2DM (OR = 1.109; 95% CI: 1.008–1.222; *p* = 0.035). T2DM risk was reduced by increasing ALM and walking pace. A 1 kg^2^ increase in ALM decreased the risk of T2DM by 10.2% (OR = 0.898; 95% CI: 0.830-0.952; *p* < 0.001), but a 1 m/s increase in walking pace decreased the odds of T2DM by 90.0% (OR = 0.100; 95% CI: 0.053–0.186 *p* < 0.001). ALM also had a potential causal relationship with HbA1C (OR = −0.006; 95% CI: −0.029 to −0.017; *p* = 0.012) and 2-hour glucose post-challenge (OR = −0.292; 95% CI: −0.401 to −0.184; *p* = 0.001). **Table 3** shows the findings of the thorough MR analysis. According to the intercept of the MR–Egger regression, there was no evidence of horizontal pleiotropy between the exposures and the outcomes (*p* > 0.05). As expected, IVW findings were consistent after MR-PRESSO adjustment. The sensitivity analysis findings are shown in **Table 4**.

**Table 3:** Association sarcopenia-related traits with T2DM and glycaemic traits using Mendelian randomization.

Exposures	Outcomes	No.of Ivs	MR Egger		Weighted median		IVW(random-effect model)	
			Estimate (95%CI)	*P*	Estimate (95%CI)	*P*	Estimate (95%CI)	*P*
Low hand grip strength (EWGSOP)	T2DM	52	1.285(0.917,1.802)	0.151	1.090(1.006,1.180)	0.032	1.109(1.008,1.222)	0.035
	Fasting glucose	56	-0.030(-0.102,0.042)	0.413	0.001(-0.020,0.021)	0.945	0.011(-0.007,0.030)	0.224
	Fasting insulin	56	-0.004(-0.081,0.073)	0.914	0.030(0.003,0.050)	0.027	0.012(-0.007,0.032)	0.213
	HbA1c	30	-0.020(-0.213,0.172)	0.836	0.012(-0.037,0.060)	0.633	0.020(-0.017,0.058)	0.226
	Two-hour glucose challenge	33	-0.319(-1.280,0.642)	0.520	0.227(-0.034.0.489)	0.077	0.181(-0.021,0.383)	0.080
ALM	T2DM	552	0.823(0.704,0.964)	0.016	0.843(0.797,0.893)	<0.001	0.889(0.830,0.952)	<0.001
	Fasting glucose	617	0.004(-0.03,0.03)	0.800	-0.005(-0.02,0.01)	0.526	0.004(-0.009,0.017)	0.550
	Fasting insulin	372	-0.041(-0.110,0.032)	0.263	-0.019(-0.051,0.0004)	0.054	-0.011(-0.040,0.010)	0.264
	HbA1c	362	-0.003(-0.059,0.053)	0.921	-0.019(-0.052,0.015)	0.266	-0.006(-0.029,0.017)	0.012
	Two-hour glucose challenge	401	-0.311(-0.591,-0.031)	0.030	-0.292(-0.459,-0.125)	<0.001	-0.292(-0.401,-0.184)	<0.001
Walking pace	T2DM	56	0.403(0.030,5.440)	0.496	0.152(0.102,0.226)	<0.001	0.100(0.053,0.186)	<0.001
	Fasting glucose	56	0.004(-0.387,0.379)	0.989	0.008(-0.087,0.103)	0.864	-0.005(-0.090,0.080)	0.906
	Fasting insulin	56	0.193(-0.268,0.655)	0.415	-0.026(-0.144,0.092)	0.641	-0.063(-0.166,0.039)	0.224
	HbA1c	43	-0.020(-0.708,0.668)	0.955	-0.119(-0.313,0.075)	0.230	-0.128(-0.285,0.030)	0.113
	Two-hour glucose challenge	46	-1.157(-5.000,2.682)	0.558	0.402(-0.618,1.423)	0.440	0.227(-0.613,1.066)	0.597

T2DM, Type 2 diabetes mellitus; ALM, appendicular lean mass; HbA1c, glycated hemoglobin; EWGSOP, European Working Group on Sarcopenia in Older People.

**Table 4:** Association sarcopenia-related traits with T2DM and glycaemic traits using MR-PRESSO and MR Egger.

Exposures	Outcomes	MR-PRESSO				MR Egger		
		No.of Ivs	Estimate(95%CI)	*P*	Ivs outliers	No.of Ivs	Intercept	*P*
Low hand grip strength (EWGSOP)	T2DM	50	1.111(1.032,1.190)	0.007	rs10952289,rs34415150, rs8061064,rs823130	54	-0.008	0.322
	Fasting glucose	59	0.013(-0.007,0.031)	0.239	NA	59	0.003	0.234
	Fasting insulin	58	0.013(-0.007,0.031)	0.244	rs7740188	59	0.001	0.569
	HbA1c	36	0.021(-0.022,0.049)	0.335	NA	36	0.002	0.669
	Two-hour glucose challenge	35	0.192(-0.001,0.380)	0.060	NA	35	0.024	0.257
ALM	T2DM	511	0.872(0.831,0.900)	<0.001	rs10225945,rs11260035,rs11605297,rs12074850,rs12150907,rs1260326,rs12724708,rs12909863,rs13391980,rs17205463,rs173135,rs2071450,rs2112617,rs2142331,rs2194411,rs2236406,rs2287821,rs2289629,rs28379706,rs28592876,rs28678024,rs2871960,rs34522021,rs3764002,rs40270,rs4274112,rs4752689,rs4788218,rs4985445,rs55872725,rs6142059,rs610694,rs61919240,rs62106258,rs62370472,rs6860245,rs6931421,rs6977416,rs7020491,rs7144307,rs73052033,rs73125634,rs7418410,rs7563362,rs7610055,rs7701233,rs7768973,rs7816345,rs7826059,rs9388490,s951366,rs9634212,rs9647379,rs9669278	565	0.002	0.257
	Fasting glucose	596	0.001(-0.009,0.010)	0.907	rs12188208,rs12188208,rs17205463,rs17681189,rs2025609,rs2142331,rs2237485,rs2871960,rs34517439,rs40270,rs4752829,rs4852257,rs488621,rs57513571,rs7107356,rs7328187,rs73384223,rs7448554,rs9375188,rs9391254,rs9669278	617	6.60E-05	0.842
	Fasting insulin	377	-0.021(-0.042,0.001)	0.042	rs11098677,rs11098677,rs12724708,rs13391980,rs2142331,rs2237485,rs2287821,rs310796,rs36012032,rs40270,rs4865956,rs7229520,rs73006226,rs7328187,rs7816345	392	0.001	0.323
	HbA1c	359	0.003(-0.017,0.023)	0.747	rs12074850,rs2923411,rs3184504	362	-0.0002	0.786
	Two-hour glucose challenge	398	-0.252(-0.351,-0.152)	<0.001	rs1260326,rs17205463,rs7610055	401	0.0004	0.896
Walking pace	T2DM	52	0.133(0.091,0.200)	<0.001	rs113825410,rs11732213,rs1592,rs4643373,rs62048402	57	-0.013	0.274
	Fasting glucose	54	0.031(-0.040,0.110)	0.414	rs113825410,rs7896518	56	-2.08E-06	0.999
	Fasting insulin	54	-0.071(-0.162,0.021)	0.147	rs11039324,rs28519617	56	-0.003	0.303
	HbA1c	43	-0.153(-0.301,-0.002)	0.053	NA	43	-0.001	0.821
	Two-hour glucose challenge	46	0.231(-0.613,1.071)	0.600	NA	46	0.013	0.473

T2DM, Type 2 diabetes mellitus; ALM, appendicular lean mass; HbA1c, glycated hemoglobin; EWGSOP, European Working Group on Sarcopenia in Older People.

### 3.2 Stage 2: Genetically predicted T2DM and glycemic traits influence sarcopenia-related traits

We obtained beta, standard error, and *p*-values of SNPs significantly associated with exposure using the instrumental variables (IV) screening. In the outcomes database, we discovered the beta value, standard error, and *p*-values that correlated with the SNPs. For fasting glucose, fasting insulin, and T2DM, we found 66, 38, and 187 linkage disequilibrium (LD)-independent (r^2^ < 0.001) IVs that reached genome-wide significance level (*p* < 5×10^-8^). For HbA1c, 2-hour blood glucose challenge, we found 31 and 11 linkage disequilibrium (LD)-independent (r^2^ < 0.001) IVs that reached genome-wide significance level (*p* < 5×10^-6^). **Supplementary Tables 4-8** has detailed information about IVs. The horizontal pleiotropy test confirmed the of absence horizontal pleiotropy (*p* > 0.05). There were one IV groups that were not heterogeneous (*p* > 0.05). The IVW random-effect model was used if heterogeneity was present. Otherwise, the IVW fixed-effect model was used. The level pleiotropy and heterogeneity test results are shown in **Table 5**.

**Table 5:** Association T2DM and glycaemic traits with sarcopenia-related traits using heterogeneity test and pleiotropy test.

Exposures	Outcomes	No.of Ivs	Heterogeneity test		Pleiotropy_test
			Cochran's Q	*P*	*P*
T2DM	Low hand grip strength (EWGSOP)	180	367.2484	<0.001	0.287
	ALM	181	5109.0300	<0.001	0.157
	Walking pace	163	395.5066	<0.001	0.139
Fasting glucose	Low hand grip strength (EWGSOP)	64	106.3683	<0.001	0.886
	ALM	64	1120.4190	<0.001	0.648
	Walking pace	40	113.3818	<0.001	0.141
Fasting insulin	Low hand grip strength (EWGSOP)	38	80.3508	<0.001	0.691
	ALM	38	2377.4080	<0.001	0.326
	Walking pace	37	164.1926	<0.001	0.523
HbA1c	Low hand grip strength (EWGSOP)	28	43.4848	0.023	0.061
	ALM	29	368.1774	<0.001	0.073
	Walking pace	29	60.4644	<0.001	0.970
Two-hour glucose challenge	Low hand grip strength (EWGSOP)	7	8.6807	0.192	0.086
	ALM	7	288.4325	<0.001	0.406
	Walking pace	7	25.4576	<0.001	0.528

T2DM, Type 2 diabetes mellitus; ALM, appendicular lean mass; HbA1c, glycated hemoglobin; EWGSOP, European Working Group on Sarcopenia in Older People.

This MR analysis mostly refers to IVW. It was shown that T2DM was a potential pathogenic factor in sarcopenia-related characteristics (*p <* 0.05), except for ALM (β = 0.018; 95% CI: −0.008 to −0.044; *p* = 0.168). There was a potential positive correlation between fasting insulin and walking pace (β = 0.062; 95% CI: −0.0001 to −0.125; *p* = 0.050). There was no correlation between HbA1c and sarcopenia-related characteristics (*p* > 0.05). Low hand-grip strength (OR = 0.058; 95% CI: 0.005–0.111; *p* = 0.031) was the only sarcopenia-related characteristic causally linked with a 2-hour glucose test. A gene predictive of 2-hour glucose test results after the challenge would increase the of having a weak hand-grip strength. **Table 6** shows the findings of the MR analysis. No horizontal pleiotropy (*p* > 0.05) was found in the MR-Egger regression of exposures and outcomes, as shown by the intercept. As expected, the IVW findings were consistent after MR-PRESSO adjustment. The sensitivity analysis findings are shown in **Table 7**.

**Table 6:** Association T2DM and glycaemic traits with sarcopenia-related traits using Mendelian randomization.

Exposures	Outcomes	No.of Ivs	MR Egger		Weighted median		IVW(random-effect model)	
			OR(95%CI)	*P*	Estimate(95%CI)	*P*	Estimate(95%CI)	*P*
T2DM	Low hand grip strength (EWGSOP)	180	1.073(1.008,1.142)	0.030	1.043(1.004,1.082)	0.028	1.043(1.012.1.069)	0.005
	ALM	181	-0.020(-0.080,0.039)	0.504	0.009(-0.003,0.021)	0.117	0.018(-0.008,0.044)	0.168
	Walking pace	182	0.002 (-0.013,0.017)	0.799	-0.002(-0.008,0.004)	0.504	-0.013(-0.020,-0.006)	<0.001
Fasting glucose	Low hand grip strength (EWGSOP)	64	-0.033(-0.251,0.18)4	0.764	-0.102(-0.255,0.052)	0.194	-0.017(-0.136,0.101)	0.769
	ALM	64	0.073(-0.109,0.254)	0.436	0.062(0.017,0.106)	0.006	0.037(-0.062,0.136)	0.463
	Walking pace	40	-0.040(-0.101,0.031)	0.290	-0.022(-0.051,0.019)	0.386	0.011(-0.013,0.045)	0.609
Fasting insulin	Low hand grip strength (EWGSOP)	38	0.026(-0.728,0.781)	0.946	-0.141(-0.421,0.139)	0.324	-0.119(-0.367,0.128)	0.345
	ALM	38	0.773(-0.248,1.794)	0.147	0.243(0.089,0.395)	<0.001	0.283(-0.058.0.625)	0.104
	Walking pace	37	0.0002(-0.199.0.199)	0.998	0.044(-0.009,0.098)	0.106	0.062(-0.0001,0.125)	0.050
HbA1c	Low hand grip strength (EWGSOP)	29	-0.324(-0.651,0.003)	0.063	-0.076(-0.247,0.095)	0.385	0.046(-0.109,0.201)	0.385
	ALM	29	0.141(-0.084,0.366)	0.230	0.064(0.015,0.113)	0.010	-0.051(-0.154,0.052)	0.329
	Walking pace	29	-0.011(-0.076,0.054)	0.743	-0.013(-0.046,0.020)	0.453	-0.012(-0.040,0.016)	0.394
Two-hour glucose challenge	Low hand grip strength (EWGSOP)	7	0.298(0.071,0.524)	0.049	0.084(0.013,0.154)	0.020	0.058(0.005,0.122)	0.031
	ALM	7	0.110(-0.298,0.517)	0.620	0.007(-0.014,0.029)	0.514	-0.073(-0.167,0.021)	0.126
	Walking pace	7	0.034(-0.050,0.119)	0.458	-0.002(-0.015,0.011)	0.739	0.006(-0.013,0.025)	0.517

T2DM, Type 2 diabetes mellitus; ALM, appendicular lean mass; HbA1c, glycated hemoglobin; EWGSOP, European Working Group on Sarcopenia in Older People.

**Table 7:** Association T2DM and glycaemic traits with sarcopenia-related traits using MR-PRESSO and MR Egger.

Exposures	Outcomes	MR-PRESSO				MR Egger		
		No.of Ivs	Estimate(95%CI)	*P*	Ivs outliers	No.of Ivs	Intercept	*P*
T2DM	Low hand grip strength (EWGSOP)	182	1.031(1.012,1.060)	0.002	rs1117610,rs601945,rs9379084	185	-0.002	0.411
	ALM	118	0.014(0.004,0.025)	0.007	rs1007090,rs10097617,rs10419627,rs11063069,rs1117610,rs115505614,rs11699802,rs11708067,rs11709077,rs11759026,rs11856307,rs12325539,rs1260326,rs12910361,rs12920022,rs13022337,rs13385171,rs13389219,rs1359790,rs1426371,rs1517037,rs1562396,rs1665901,rs1705263,rs17744783,rs2023681,rs2277536,rs2383205,rs2820441,rs2925979,rs2972144,rs3094682,rs34341,rs348330,rs35777422,rs35895680,rs3768321,rs3786900,rs3798519,rs459193,rs4686471,rs4709746,rs474513,rs4804833,rs490689,rs4925109,rs4977213,rs505922,rs55653563,rs55872725,rs58432198,rs58642235,rs601945,rs62107261,rs62271373,rs6937438,rs72802358,rs739846,rs7633675,rs7667864,rs76895963,rs7732130,rs77464186,rs8097210,rs8192675,rs878521,rs9368222,rs9379084	186	0.003	0.132
	Walking pace	159	-0.007(-0.012,-0.002)	0.010	rs13330951,rs34990153,rs601945, rs9873519	167	-0.001	0.160
Fasting glucose	Low hand grip strength (EWGSOP)	66	-0.021(-0.142,0.092)	0.695	NA	66	5.89E-05	0.981
	ALM	44	-0.010(-0.071,0.041)	0.528	rs10838693,rs11603349,rs11708067,rs1260326,rs12888855,rs157512,rs16851397,rs16913693,rs17265513,rs174583,rs1820176,rs189548,rs2238435,rs3842753,rs6113722,rs6489811,rs7163757,rs77981966,rs78132593,rs878521	64	-0.001	0.648
	Walking pace	42	-0.002(-0.032,0.028)	0.885	rs10974438,rs11603349,rs4862423,rs6662924	42	0.001	0.240
Fasting insulin	Low hand grip strength (EWGSOP)	36	-0.133(-0.341,0.081)	0.238	rs1351394,rs6905288	68	-0.002	0.691
	ALM	38	0.279(-0.061,0.632)	0.112	NA	38	-0.009	0.326
	Walking pace	32	0.080(0.031,0.122)	0.003	rs10865959,rs11708067,rs1351394,rs3775380,rs5017305	37	0.001	0.523
HbA1c	Low hand grip strength (EWGSOP)	30	0.022(-0.121,0.150)	0.822	NA	30	0.009	0.051
	ALM	19	0.021(-0.033,0.232)	0.502	rs12819124,rs16926246,rs17533945,rs1800562,rs2723517,rs4844390,rs552976,rs7355559,rs7805661,rs837763	29	-0.006	0.052
	Walking pace	28	-0.002(-0.031,0.030)	0.884	rs837763	29	0.0003	0.722
Two-hour glucose challenge	Low hand grip strength (EWGSOP)	7	0.061(-0.005,0.122)	0.122	NA	7	-0.029	0.086
	ALM	7	-0.070(-0.171,0.022)	0.177	NA	7	-0.022	0.406
	Walking pace	6	-0.005(-0.010,0.003)	0.28	rs10423928	7	-0.003	0.528

T2DM, Type 2 diabetes mellitus; ALM, appendicular lean mass; HbA1c, glycated hemoglobin; EWGSOP, European Working Group on Sarcopenia in Older People.

## 4 Discussion

The bi-directional two-sample MR analysis in this study was performed using summary-level data. We examined the potential causative connections between sarcopenia-related characteristics (hand-grip strength, ALM, and walking pace) and T2DM and glycemic features. Major findings were: (1) Sarcopenia-related variables predicted genetically were all causally associated with T2DM; (2) There was a potential causal relationship between genetically predicted T2DM and sarcopenia-related characteristics but not expected ALM in the other direction. (3) The results showed there was potential bi-directional causation between sarcopenia and T2DM to a greater extent.

In observational studies, hand-grip strength was shown to have a bidirectional relationship with T2DM. A previous English longitudinal study found that for each unit gain in hand-grip strength, T2DM risk was reduced by 2% (HR = 0.98; 95% CI: 0.96–0.99) (25). In another cohort study, T2DM was an independent predictor of weak hand-grip strength (26). A TSMR investigation was conducted to evaluate the causal relationship and discovered a bi-directional causal relationship between hand-grip strength and T2DM (27). Our results were consistent with these previous studies and provided evidence of a potential bi-directional causality between hand-grip strength and T2DM. In a long-term retrospective analysis of 159 older women, researchers found that patients with low ALM had a 3.81 higher incidence of IFG/T2DM than those with normal ALM (OR = 3.81; 95% CI: 1.09-9.80) (28). Our MR findings follow prior research that ALM decreased the risk of T2DM. But in the other direction, T2DM was not causally associated with ALM. Notably, the major glucose metabolic reservoir of T2DM was in muscle mass, suggesting that our results only partially demonstrate a causal relationship between ALM and diabetes. More studies with better MR methods and data are needed in the future to validate the causal relationship between ALM and diabetes. Prospective studies have shown that a slower walking pace was associated with an increased risk of T2DM. Men and women who walked slowly were shown to have an increased risk of incident T2DM (29). A Baltimore Longitudinal Study of Aging found that T2DM was a significant predictor of slower gait speed in the elderly (30). Our MR findings, in contrast to those of the traditional studies, included a strong bi-directional causative connection between walking pace and T2DM.

Many previous studies had demonstrated the positive association between sarcopenia and T2DM, and the interaction between the two conditions seems to tilt more toward a bidirectional causation correlation owing to the complexity and proximity of their relationship. Landin et al. described sarcopenia and T2DM as two sides of the same coin when discussing the correlation between the two conditions (31). Through the use of transcriptome analysis, Huang et al. discovered 15 shared genes associated with both sarcopenia and T2DM, in addition to multiple shared pathways between the two disorders (32). A meta-analysis of community-dwelling Asian populations found that T2DM patients had a considerably greater incidence of sarcopenia than non-diabetics (33). In another recent meta-analysis of observational studies, T2DM was related to an elevated risk of sarcopenia (34). Our bidirectional MR study further complements previous studies and provides evidence for a potential causal relationship between sarcopenia and T2DM. Sarcopenia and TDM are both age-related diseases with the same underlying pathophysiological mechanism (4, 5, 35). T2DM is often associated with insulin resistance, increased inflammation, accumulation of advanced glycation end products, and elevated oxidative stress (36). These pathways would result in activated inflammation, mitochondrial and vascular dysfunction, and protein metabolism deficiencies, all of which would be harmful to muscle health, including muscle mass, muscular strength, and muscle quality (32). Because of reduced insulin sensitivity, the anabolic activity of insulin in skeletal muscle may be gradually lost in T2DM. Furthermore, poor insulin action may result in increased protein breakdown and reduced protein synthesis, resulting in a loss of muscle mass and strength (37, 38). As a result, T2DM patients are at a higher risk of sarcopenia (39, 40). On the other hand, older people may be at a higher risk for developing T2DM due to the effects of sarcopenia. Sarcopenia is a condition defined by decreased muscular mass, low muscle strength, or poor athletic performance (41), and may contribute to impaired glucose clearance, increased inflammation, and reduced metabolic rate and physical activity. Skeletal muscle is essential for glucose clearance, accounting for more than 80% of postprandial glucose uptake. As a result, sarcopenia alters glucose disposal by lowering muscle mass and increasing localized inflammation, which may contribute to the development and progression of T2DM (42, 43).

Our research has several advantages. First, this was the first bidirectional MR study to investigate the causal association between three sarcopenia-related traits (EWGSOP) and T2DM, especially evaluating the causal relationship between walking pace and T2DM, which had been neglected in previous studies. Second, the database for this study was the most recent and reliable. The GWAS summary data for low hand-grip strength was defined by EWGSOP; T2DM data originated from one of the largest diabetes case-control studies, which was published in 2022. Third, different MR analysis methods were used to confirm the correctness and validity of the results, and sensitivity analyses such as MR-PRESSO were performed to obtain consistent estimations of the causal impact sizes of MR. Finally, we investigated the link between walking pace and diabetes, which had been neglected in earlier research. Still, the study’s weaknesses should be mentioned. To begin, this study was carried out using summary-level data, which restricted our capacity to carry out subgroup analyses, such as those regarding low hand-grip strength and ALM according to gender. Besides, we applied ALM (appendicular lean mass) rather than ASM (appendicular skeletal muscle mass) to evaluate muscle mass, which may be inaccurate due to bias from other non-fat soft tissue components such as lungs, kidneys, and other internal organs. In addition, our research population was exclusive of European descent, so our results may not apply to other racial or ethnic groups. At last, residual bias cannot be avoided, as it is a recognized shortcoming of the MR technique, even when the pleiotropy test and MR-PRESSO procedures are used to prevent confounding by pleiotropy.

In conclusion, sarcopenia and T2DM may mutually have a potential causal influence on each other. Our study suggests that sarcopenia-related traits (hand-grip strength, ALM, walking pace) may benefit T2DM. Instead, T2DM may be linked to decreased hand-grip strength and walking pace. This study provides solid evidence that hand-grip strength, ALM and walking pace are possible predictors of T2DM in middle-aged and elderly people.

## Data availability statement

The original contributions presented in the study are included in the article/**Supplementary Materials**. Further inquiries can be directed to the corresponding author.

## Author contributions

Study design: LY, SC. Manuscript writing: SC, SY. Statistical analysis and data interpretation: SY, NA, KK. Critical revision of the manuscript: LY, XY. Literature Search: SC, SY, NA, KK, QW, TZ. All authors contributed to the article and approved the submitted version.
