# Supplementary material for: A bi-directional Mendelian randomization study of sarcopenia-related traits and type 2 diabetes mellitus
Source: Front Endocrinol (Lausanne). 2023 Mar 8;14:1109800. doi: 10.3389/fendo.2023.1109800 (PMC10031051; doi:10.3389/fendo.2023.1109800)
Supplement: Supplementary file 1 [file DataSheet_1.docx]

Supplementary Material

**Supplementary Table 1.** Genome-wide significant SNPs for Low hand grip strength (60 years and older) (EWGSOP).1

**Supplementary Table 2.** Genome-wide significant SNPs for ALM.7

**Supplementary Table 3.** Genome-wide significant SNPs for Walking pace.59

**Supplementary Table4.** Genome-wide significant SNPs for T2DM.65

**Supplementary Table 5.** Genome-wide significant SNPs for Fasting glucose 77

**Supplementary Table 6.** Genome-wide significant SNPs for Fasting insulin 82

**Supplementary Table 7.** Genome-wide significant SNPs for HbA1c 85

**Supplementary Table 8.** Genome-wide significant SNPs for Two-hour glucose challenge 87

**Supplementary Table 1.** Genome-wide significant SNPs for Low hand grip strength (60 years and older) (EWGSOP)

| **SNP** | **exposure** | **outcome** | **beta.exposure** | **se.exposure** | **pval.exposure** | **beta.outcome** | **se.outcome** | **pval.outcome** |
| --- | --- | --- | --- | --- | --- | --- | --- | --- |
| rs10070428 | Low hand grip strength | Fasting glucose | 0.0397 | 0.0074 | 7.24002E-08 | -0.0021 | 0.0018 | 0.4111 |
| rs1017323 | Low hand grip strength | Fasting glucose | -0.0537 | 0.0117 | 4.21105E-06 | 0.0003 | 0.003 | 0.7819 |
| rs1056074 | Low hand grip strength | Fasting glucose | 0.0461 | 0.0096 | 1.57801E-06 | -0.0008 | 0.0025 | 0.6148 |
| rs10872469 | Low hand grip strength | Fasting glucose | -0.0429 | 0.009 | 1.81798E-06 | -0.0015 | 0.0021 | 0.5801 |
| rs10940166 | Low hand grip strength | Fasting glucose | 0.0364 | 0.0075 | 0.000001236 | -0.0016 | 0.0017 | 0.139 |
| rs10952289 | Low hand grip strength | Fasting glucose | -0.0435 | 0.0078 | 2.10402E-08 | 0.0025 | 0.0018 | 0.1184 |
| rs11236213 | Low hand grip strength | Fasting glucose | -0.0504 | 0.008 | 3.01099E-10 | -0.0004 | 0.002 | 0.9607 |
| rs112689121 | Low hand grip strength | Fasting glucose | 0.0479 | 0.01 | 1.56498E-06 | 0.0038 | 0.0026 | 0.1951 |
| rs12140813 | Low hand grip strength | Fasting glucose | 0.0511 | 0.0094 | 4.76299E-08 | -0.003 | 0.0023 | 0.2317 |
| rs12520233 | Low hand grip strength | Fasting glucose | 0.036 | 0.0074 | 0.000001096 | 0.0045 | 0.0018 | 0.0198802 |
| rs12790261 | Low hand grip strength | Fasting glucose | 0.0685 | 0.0134 | 3.41602E-07 | 0.0141 | 0.0055 | 0.00505406 |
| rs13023281 | Low hand grip strength | Fasting glucose | 0.042 | 0.0089 | 2.08502E-06 | 0.0026 | 0.0032 | 0.6742 |
| rs13107325 | Low hand grip strength | Fasting glucose | 0.0897 | 0.0138 | 7.41822E-11 | -0.0031 | 0.0038 | 0.3045 |
| rs143384 | Low hand grip strength | Fasting glucose | -0.0545 | 0.0075 | 4.47301E-13 | 0.0028 | 0.0017 | 0.0454203 |
| rs143459567 | Low hand grip strength | Fasting glucose | 0.1185 | 0.0189 | 3.40502E-10 | -0.001 | 0.0069 | 0.9185 |
| rs145933237 | Low hand grip strength | Fasting glucose | 0.1344 | 0.0283 | 2.03798E-06 | 0.0129 | 0.0096 | 0.204 |
| rs146169218 | Low hand grip strength | Fasting glucose | -0.0946 | 0.0206 | 4.28302E-06 | -0.0003 | 0.0044 | 0.9353 |
| rs151049793 | Low hand grip strength | Fasting glucose | 0.1357 | 0.029 | 3.00698E-06 | -0.0138 | 0.0106 | 0.1838 |
| rs1556659 | Low hand grip strength | Fasting glucose | -0.0388 | 0.0076 | 3.47896E-07 | -0.0055 | 0.0017 | 0.008077 |
| rs1635527 | Low hand grip strength | Fasting glucose | -0.038 | 0.0074 | 2.87899E-07 | 0.0014 | 0.0018 | 0.4138 |
| rs16892234 | Low hand grip strength | Fasting glucose | -0.0553 | 0.0121 | 4.45903E-06 | -0.0065 | 0.003 | 0.0261999 |
| rs17488051 | Low hand grip strength | Fasting glucose | -0.0361 | 0.0079 | 4.71498E-06 | -0.0029 | 0.002 | 0.2153 |
| rs17743567 | Low hand grip strength | Fasting glucose | -0.0965 | 0.0208 | 3.57001E-06 | 0.0095 | 0.0056 | 0.0764592 |
| rs183231946 | Low hand grip strength | Fasting glucose | 0.1358 | 0.029 | 0.00000274 | 0.006 | 0.0146 | 0.4558 |
| rs185320691 | Low hand grip strength | Fasting glucose | 0.0913 | 0.0146 | 3.84397E-10 | 0.0098 | 0.0106 | 0.4332 |
| rs1989803 | Low hand grip strength | Fasting glucose | 0.0386 | 0.0078 | 6.38602E-07 | 0.0022 | 0.0017 | 0.338 |
| rs2293955 | Low hand grip strength | Fasting glucose | 0.0568 | 0.0122 | 2.98497E-06 | 0.0059 | 0.0032 | 0.1815 |
| rs2371292 | Low hand grip strength | Fasting glucose | -0.0579 | 0.0122 | 1.96101E-06 | -0.0011 | 0.0032 | 0.746401 |
| rs263292 | Low hand grip strength | Fasting glucose | 0.0344 | 0.0075 | 4.83905E-06 | 0.0047 | 0.0019 | 0.00471998 |
| rs2723521 | Low hand grip strength | Fasting glucose | -0.0365 | 0.0076 | 1.68702E-06 | 0.0021 | 0.0019 | 0.4834 |
| rs2899611 | Low hand grip strength | Fasting glucose | 0.0431 | 0.0074 | 6.01395E-09 | -0.001 | 0.0018 | 0.5924 |
| rs3025020 | Low hand grip strength | Fasting glucose | -0.0386 | 0.0084 | 3.75803E-06 | -0.0071 | 0.003 | 0.0485501 |
| rs3118903 | Low hand grip strength | Fasting glucose | 0.0575 | 0.0088 | 6.71429E-11 | 0.0006 | 0.0022 | 0.3961 |
| rs326217 | Low hand grip strength | Fasting glucose | 0.0375 | 0.008 | 2.78798E-06 | -0.0017 | 0.0018 | 0.4111 |
| rs34415150 | Low hand grip strength | Fasting glucose | 0.0833 | 0.0099 | 4.42181E-17 | -0.0057 | 0.0036 | 0.0318302 |
| rs34464763 | Low hand grip strength | Fasting glucose | 0.0544 | 0.0086 | 3.15203E-10 | -0.0176 | 0.0068 | 0.0104501 |
| rs35661497 | Low hand grip strength | Fasting glucose | 0.0701 | 0.0147 | 1.88899E-06 | -0.0018 | 0.0039 | 0.3501 |
| rs4292598 | Low hand grip strength | Fasting glucose | -0.0415 | 0.0086 | 0.00000126 | 0.0003 | 0.0024 | 0.9498 |
| rs4297869 | Low hand grip strength | Fasting glucose | 0.035 | 0.0075 | 2.94002E-06 | 0.0019 | 0.0019 | 0.2409 |
| rs4392169 | Low hand grip strength | Fasting glucose | -0.0418 | 0.0089 | 2.63603E-06 | 0.0032 | 0.0022 | 0.0628203 |
| rs55941591 | Low hand grip strength | Fasting glucose | -0.0543 | 0.0114 | 2.01201E-06 | -0.0061 | 0.0027 | 0.0235299 |
| rs577267 | Low hand grip strength | Fasting glucose | -0.039 | 0.0079 | 8.57097E-07 | -0.0008 | 0.0021 | 0.6897 |
| rs62102286 | Low hand grip strength | Fasting glucose | -0.0487 | 0.0074 | 5.48909E-11 | 0.0016 | 0.0018 | 0.8265 |
| rs62333225 | Low hand grip strength | Fasting glucose | 0.0407 | 0.0089 | 4.33701E-06 | 0.0041 | 0.0023 | 0.1768 |
| rs6906899 | Low hand grip strength | Fasting glucose | 0.0348 | 0.0074 | 2.64899E-06 | 0.0019 | 0.0018 | 0.2388 |
| rs7185040 | Low hand grip strength | Fasting glucose | 0.052 | 0.0096 | 5.57006E-08 | 0.0014 | 0.0024 | 0.4131 |
| rs72815799 | Low hand grip strength | Fasting glucose | 0.0477 | 0.0098 | 0.000001102 | -0.0002 | 0.0024 | 0.6962 |
| rs7624084 | Low hand grip strength | Fasting glucose | -0.0428 | 0.0074 | 8.50707E-09 | -0.0065 | 0.0018 | 0.00337404 |
| rs76652726 | Low hand grip strength | Fasting glucose | 0.0361 | 0.0075 | 1.76799E-06 | -0.0001 | 0.0019 | 0.8104 |
| rs7740188 | Low hand grip strength | Fasting glucose | 0.0396 | 0.0081 | 9.8951E-07 | -0.0041 | 0.002 | 0.0361002 |
| rs7967622 | Low hand grip strength | Fasting glucose | 0.0392 | 0.0078 | 4.94299E-07 | 0.0001 | 0.0019 | 0.7694 |
| rs79723785 | Low hand grip strength | Fasting glucose | 0.1674 | 0.0293 | 1.15899E-08 | -0.0019 | 0.0126 | 0.679 |
| rs8061064 | Low hand grip strength | Fasting glucose | 0.0407 | 0.0074 | 3.54601E-08 | 0.0027 | 0.0016 | 0.2561 |
| rs823130 | Low hand grip strength | Fasting glucose | 0.0341 | 0.0074 | 4.25902E-06 | -0.0039 | 0.0018 | 0.0284001 |
| rs9309884 | Low hand grip strength | Fasting glucose | -0.0388 | 0.0075 | 2.25102E-07 | -0.0024 | 0.0019 | 0.1426 |
| rs9579628 | Low hand grip strength | Fasting glucose | -0.0444 | 0.0095 | 2.90898E-06 | -0.0005 | 0.0024 | 0.8807 |
| rs958685 | Low hand grip strength | Fasting glucose | -0.0428 | 0.0074 | 6.51793E-09 | -0.0001 | 0.0018 | 0.8694 |
| rs9830552 | Low hand grip strength | Fasting glucose | -0.0351 | 0.0075 | 2.97797E-06 | 0.001 | 0.0019 | 0.3369 |
| rs10070428 | Low hand grip strength | Fasting insulin | 0.0397 | 0.0074 | 7.24E-08 | 0.0031 | 0.002 | 0.1535 |
| rs1017323 | Low hand grip strength | Fasting insulin | -0.0537 | 0.0117 | 4.21E-06 | -0.0044 | 0.0033 | 0.0715797 |
| rs1056074 | Low hand grip strength | Fasting insulin | 0.0461 | 0.0096 | 1.58E-06 | -0.0003 | 0.0028 | 0.8469 |
| rs10872469 | Low hand grip strength | Fasting insulin | -0.0429 | 0.009 | 1.82E-06 | 0.0014 | 0.0023 | 0.3157 |
| rs10940166 | Low hand grip strength | Fasting insulin | 0.0364 | 0.0075 | 0.000001236 | -0.0014 | 0.0019 | 0.2193 |
| rs10952289 | Low hand grip strength | Fasting insulin | -0.0435 | 0.0078 | 2.10E-08 | 0.0046 | 0.0021 | 0.02784 |
| rs11236213 | Low hand grip strength | Fasting insulin | -0.0504 | 0.008 | 3.01E-10 | -0.0013 | 0.0022 | 0.3583 |
| rs112689121 | Low hand grip strength | Fasting insulin | 0.0479 | 0.01 | 1.56E-06 | 0.0033 | 0.003 | 0.2789 |
| rs12140813 | Low hand grip strength | Fasting insulin | 0.0511 | 0.0094 | 4.76E-08 | -0.0077 | 0.0027 | 0.0004144 |
| rs12520233 | Low hand grip strength | Fasting insulin | 0.036 | 0.0074 | 0.000001096 | 0.0019 | 0.002 | 0.5904 |
| rs12790261 | Low hand grip strength | Fasting insulin | 0.0685 | 0.0134 | 3.42E-07 | 0.0083 | 0.0063 | 0.4757 |
| rs13023281 | Low hand grip strength | Fasting insulin | 0.042 | 0.0089 | 2.09E-06 | 0.0024 | 0.0036 | 0.4169 |
| rs13107325 | Low hand grip strength | Fasting insulin | 0.0897 | 0.0138 | 7.42E-11 | -0.0102 | 0.0044 | 0.00937303 |
| rs143384 | Low hand grip strength | Fasting insulin | -0.0545 | 0.0075 | 4.47E-13 | -0.0029 | 0.0019 | 0.0658294 |
| rs143459567 | Low hand grip strength | Fasting insulin | 0.1185 | 0.0189 | 3.41E-10 | 0.0041 | 0.008 | 0.9949 |
| rs145933237 | Low hand grip strength | Fasting insulin | 0.1344 | 0.0283 | 2.04E-06 | 0.0087 | 0.0109 | 0.3725 |
| rs146169218 | Low hand grip strength | Fasting insulin | -0.0946 | 0.0206 | 4.28E-06 | 0.0022 | 0.0049 | 0.3716 |
| rs151049793 | Low hand grip strength | Fasting insulin | 0.1357 | 0.029 | 3.01E-06 | -0.0099 | 0.0116 | 0.3017 |
| rs1556659 | Low hand grip strength | Fasting insulin | -0.0388 | 0.0076 | 3.48E-07 | -0.0021 | 0.002 | 0.1795 |
| rs1635527 | Low hand grip strength | Fasting insulin | -0.038 | 0.0074 | 2.88E-07 | -0.0006 | 0.002 | 0.749401 |
| rs16892234 | Low hand grip strength | Fasting insulin | -0.0553 | 0.0121 | 4.46E-06 | -0.0015 | 0.0034 | 0.6964 |
| rs17488051 | Low hand grip strength | Fasting insulin | -0.0361 | 0.0079 | 4.71E-06 | -0.0002 | 0.0022 | 0.8957 |
| rs17743567 | Low hand grip strength | Fasting insulin | -0.0965 | 0.0208 | 3.57E-06 | 0.0042 | 0.0062 | 0.7591 |
| rs183231946 | Low hand grip strength | Fasting insulin | 0.1358 | 0.029 | 0.00000274 | -0.0235 | 0.016 | 0.1529 |
| rs185320691 | Low hand grip strength | Fasting insulin | 0.0913 | 0.0146 | 3.84E-10 | -0.0101 | 0.0117 | 0.5924 |
| rs1989803 | Low hand grip strength | Fasting insulin | 0.0386 | 0.0078 | 6.39E-07 | 0.0018 | 0.0019 | 0.8634 |
| rs2293955 | Low hand grip strength | Fasting insulin | 0.0568 | 0.0122 | 2.98E-06 | 0.0036 | 0.0036 | 0.6426 |
| rs2371292 | Low hand grip strength | Fasting insulin | -0.0579 | 0.0122 | 1.96E-06 | -0.0031 | 0.0035 | 0.3615 |
| rs263292 | Low hand grip strength | Fasting insulin | 0.0344 | 0.0075 | 4.84E-06 | 0.0039 | 0.0021 | 0.0335398 |
| rs2723521 | Low hand grip strength | Fasting insulin | -0.0365 | 0.0076 | 1.69E-06 | 0.0023 | 0.0021 | 0.2931 |
| rs2899611 | Low hand grip strength | Fasting insulin | 0.0431 | 0.0074 | 6.01E-09 | -0.0014 | 0.002 | 0.3882 |
| rs3025020 | Low hand grip strength | Fasting insulin | -0.0386 | 0.0084 | 3.76E-06 | 0.0083 | 0.0035 | 0.0151799 |
| rs3118903 | Low hand grip strength | Fasting insulin | 0.0575 | 0.0088 | 6.71E-11 | 0.0055 | 0.0025 | 0.0337699 |
| rs326217 | Low hand grip strength | Fasting insulin | 0.0375 | 0.008 | 2.79E-06 | -0.0035 | 0.002 | 0.1225 |
| rs34415150 | Low hand grip strength | Fasting insulin | 0.0833 | 0.0099 | 4.42E-17 | 0.0066 | 0.004 | 0.2014 |
| rs34464763 | Low hand grip strength | Fasting insulin | 0.0544 | 0.0086 | 3.15E-10 | -0.0035 | 0.0081 | 0.6804 |
| rs35661497 | Low hand grip strength | Fasting insulin | 0.0701 | 0.0147 | 1.89E-06 | 0.0047 | 0.0044 | 0.383 |
| rs4292598 | Low hand grip strength | Fasting insulin | -0.0415 | 0.0086 | 0.00000126 | -0.0001 | 0.0029 | 0.7388 |
| rs4297869 | Low hand grip strength | Fasting insulin | 0.035 | 0.0075 | 2.94E-06 | 0.0013 | 0.0021 | 0.783901 |
| rs4392169 | Low hand grip strength | Fasting insulin | -0.0418 | 0.0089 | 2.64E-06 | 0.0023 | 0.0026 | 0.1082 |
| rs55941591 | Low hand grip strength | Fasting insulin | -0.0543 | 0.0114 | 2.01E-06 | -0.0066 | 0.0031 | 0.0728199 |
| rs577267 | Low hand grip strength | Fasting insulin | -0.039 | 0.0079 | 8.57E-07 | 0.0003 | 0.0025 | 0.7901 |
| rs62102286 | Low hand grip strength | Fasting insulin | -0.0487 | 0.0074 | 5.49E-11 | 0.0011 | 0.002 | 0.5331 |
| rs62333225 | Low hand grip strength | Fasting insulin | 0.0407 | 0.0089 | 4.34E-06 | 0.0001 | 0.0027 | 0.784899 |
| rs6906899 | Low hand grip strength | Fasting insulin | 0.0348 | 0.0074 | 2.65E-06 | 0.0035 | 0.002 | 0.0753494 |
| rs7185040 | Low hand grip strength | Fasting insulin | 0.052 | 0.0096 | 5.57E-08 | 0.0028 | 0.0027 | 0.344 |
| rs72815799 | Low hand grip strength | Fasting insulin | 0.0477 | 0.0098 | 0.000001102 | 0.0024 | 0.0028 | 0.6482 |
| rs7624084 | Low hand grip strength | Fasting insulin | -0.0428 | 0.0074 | 8.51E-09 | -0.0014 | 0.0021 | 0.8883 |
| rs76652726 | Low hand grip strength | Fasting insulin | 0.0361 | 0.0075 | 1.77E-06 | 0.0005 | 0.0021 | 0.5524 |
| rs7740188 | Low hand grip strength | Fasting insulin | 0.0396 | 0.0081 | 9.90E-07 | 0.0085 | 0.0022 | 0.000155901 |
| rs7967622 | Low hand grip strength | Fasting insulin | 0.0392 | 0.0078 | 4.94E-07 | 0.0025 | 0.0021 | 0.1747 |
| rs79723785 | Low hand grip strength | Fasting insulin | 0.1674 | 0.0293 | 1.16E-08 | -0.0219 | 0.0158 | 0.174 |
| rs8061064 | Low hand grip strength | Fasting insulin | 0.0407 | 0.0074 | 3.55E-08 | 0.0039 | 0.0019 | 0.0978791 |
| rs823130 | Low hand grip strength | Fasting insulin | 0.0341 | 0.0074 | 4.26E-06 | -0.0025 | 0.0021 | 0.1435 |
| rs9309884 | Low hand grip strength | Fasting insulin | -0.0388 | 0.0075 | 2.25E-07 | -0.0029 | 0.0021 | 0.2931 |
| rs9579628 | Low hand grip strength | Fasting insulin | -0.0444 | 0.0095 | 2.91E-06 | -0.0004 | 0.0027 | 0.8699 |
| rs958685 | Low hand grip strength | Fasting insulin | -0.0428 | 0.0074 | 6.52E-09 | 0.0035 | 0.002 | 0.2039 |
| rs9830552 | Low hand grip strength | Fasting insulin | -0.0351 | 0.0075 | 2.98E-06 | 0.0015 | 0.0021 | 0.7042 |
| rs9902761 | Low hand grip strength | Fasting insulin | -0.0446 | 0.0085 | 1.64E-07 | 0.0002 | 0.0025 | 0.6093 |
| rs10070428 | Low hand grip strength | HbA1C | 0.0397 | 0.0074 | 7.24E-08 | 0.0039 | 0.0034 | 0.244 |
| rs1017323 | Low hand grip strength | HbA1C | -0.0537 | 0.0117 | 4.21E-06 | 0.0032 | 0.0052 | 0.5383 |
| rs10940166 | Low hand grip strength | HbA1C | 0.0364 | 0.0075 | 0.000001236 | -0.003 | 0.0035 | 0.3963 |
| rs10952289 | Low hand grip strength | HbA1C | -0.0435 | 0.0078 | 2.10E-08 | 0.0013 | 0.0036 | 0.724001 |
| rs112689121 | Low hand grip strength | HbA1C | 0.0479 | 0.01 | 1.56E-06 | -0.0023 | 0.0049 | 0.634699 |
| rs12140813 | Low hand grip strength | HbA1C | 0.0511 | 0.0094 | 4.76E-08 | -0.0041 | 0.0044 | 0.3418 |
| rs12520233 | Low hand grip strength | HbA1C | 0.036 | 0.0074 | 0.000001096 | 0.0002 | 0.0034 | 0.9593 |
| rs13107325 | Low hand grip strength | HbA1C | 0.0897 | 0.0138 | 7.42E-11 | 0.0013 | 0.007 | 0.8555 |
| rs143384 | Low hand grip strength | HbA1C | -0.0545 | 0.0075 | 4.47E-13 | -0.0066 | 0.0037 | 0.0755005 |
| rs146169218 | Low hand grip strength | HbA1C | -0.0946 | 0.0206 | 4.28E-06 | -0.0039 | 0.0081 | 0.6277 |
| rs1556659 | Low hand grip strength | HbA1C | -0.0388 | 0.0076 | 3.48E-07 | -0.003 | 0.0037 | 0.416 |
| rs1635527 | Low hand grip strength | HbA1C | -0.038 | 0.0074 | 2.88E-07 | -0.0076 | 0.0034 | 0.0259203 |
| rs16892234 | Low hand grip strength | HbA1C | -0.0553 | 0.0121 | 4.46E-06 | -0.0034 | 0.0056 | 0.5405 |
| rs17488051 | Low hand grip strength | HbA1C | -0.0361 | 0.0079 | 4.71E-06 | -0.0031 | 0.0038 | 0.4186 |
| rs1989803 | Low hand grip strength | HbA1C | 0.0386 | 0.0078 | 6.39E-07 | -0.0061 | 0.0059 | 0.2987 |
| rs2371292 | Low hand grip strength | HbA1C | -0.0579 | 0.0122 | 1.96E-06 | -0.0079 | 0.0057 | 0.168 |
| rs263292 | Low hand grip strength | HbA1C | 0.0344 | 0.0075 | 4.84E-06 | 0.0015 | 0.0036 | 0.676799 |
| rs2723521 | Low hand grip strength | HbA1C | -0.0365 | 0.0076 | 1.69E-06 | -0.013 | 0.0036 | 0.000346897 |
| rs2899611 | Low hand grip strength | HbA1C | 0.0431 | 0.0074 | 6.01E-09 | -0.0016 | 0.0062 | 0.796 |
| rs3118903 | Low hand grip strength | HbA1C | 0.0575 | 0.0088 | 6.71E-11 | -0.0042 | 0.0041 | 0.3078 |
| rs326217 | Low hand grip strength | HbA1C | 0.0375 | 0.008 | 2.79E-06 | -0.0005 | 0.0038 | 0.9019 |
| rs34464763 | Low hand grip strength | HbA1C | 0.0544 | 0.0086 | 3.15E-10 | -0.001 | 0.0035 | 0.781 |
| rs4297869 | Low hand grip strength | HbA1C | 0.035 | 0.0075 | 2.94E-06 | 0.0009 | 0.0037 | 0.7998 |
| rs4392169 | Low hand grip strength | HbA1C | -0.0418 | 0.0089 | 2.64E-06 | 0.0061 | 0.0044 | 0.1652 |
| rs55941591 | Low hand grip strength | HbA1C | -0.0543 | 0.0114 | 2.01E-06 | 0.0007 | 0.0053 | 0.8968 |
| rs577267 | Low hand grip strength | HbA1C | -0.039 | 0.0079 | 8.57E-07 | -0.0025 | 0.0038 | 0.5041 |
| rs62102286 | Low hand grip strength | HbA1C | -0.0487 | 0.0074 | 5.49E-11 | 0.0023 | 0.0034 | 0.4967 |
| rs6906899 | Low hand grip strength | HbA1C | 0.0348 | 0.0074 | 2.65E-06 | 0.004 | 0.0035 | 0.2501 |
| rs7185040 | Low hand grip strength | HbA1C | 0.052 | 0.0096 | 5.57E-08 | 0.0103 | 0.0067 | 0.1271 |
| rs7624084 | Low hand grip strength | HbA1C | -0.0428 | 0.0074 | 8.51E-09 | -0.0018 | 0.0035 | 0.6124 |
| rs7740188 | Low hand grip strength | HbA1C | 0.0396 | 0.0081 | 9.90E-07 | -0.0007 | 0.0037 | 0.8478 |
| rs7967622 | Low hand grip strength | HbA1C | 0.0392 | 0.0078 | 4.94E-07 | 0.0041 | 0.0037 | 0.2623 |
| rs8061064 | Low hand grip strength | HbA1C | 0.0407 | 0.0074 | 3.55E-08 | -0.0011 | 0.0034 | 0.7365 |
| rs823130 | Low hand grip strength | HbA1C | 0.0341 | 0.0074 | 4.26E-06 | -0.0078 | 0.0034 | 0.0224502 |
| rs9309884 | Low hand grip strength | HbA1C | -0.0388 | 0.0075 | 2.25E-07 | -0.0036 | 0.0035 | 0.3112 |
| rs958685 | Low hand grip strength | HbA1C | -0.0428 | 0.0074 | 6.52E-09 | 0.0055 | 0.0034 | 0.1018 |
| rs10070428 | Low hand grip strength | Two-hour glucose challenge | 0.0397 | 0.0074 | 7.24002E-08 | 0.01 | 0.019 | 0.579499 |
| rs1017323 | Low hand grip strength | Two-hour glucose challenge | -0.0537 | 0.0117 | 4.21105E-06 | 0.011 | 0.029 | 0.7019 |
| rs10940166 | Low hand grip strength | Two-hour glucose challenge | 0.0364 | 0.0075 | 0.000001236 | -0.0029 | 0.019 | 0.878 |
| rs10952289 | Low hand grip strength | Two-hour glucose challenge | -0.0435 | 0.0078 | 2.10402E-08 | -0.0057 | 0.019 | 0.7685 |
| rs112689121 | Low hand grip strength | Two-hour glucose challenge | 0.0479 | 0.01 | 1.56498E-06 | 0.014 | 0.027 | 0.600701 |
| rs12140813 | Low hand grip strength | Two-hour glucose challenge | 0.0511 | 0.0094 | 4.76299E-08 | -0.018 | 0.023 | 0.4518 |
| rs12520233 | Low hand grip strength | Two-hour glucose challenge | 0.036 | 0.0074 | 0.000001096 | 0.0073 | 0.019 | 0.6951 |
| rs13107325 | Low hand grip strength | Two-hour glucose challenge | 0.0897 | 0.0138 | 7.41822E-11 | 0.025 | 0.04 | 0.5374 |
| rs146169218 | Low hand grip strength | Two-hour glucose challenge | -0.0946 | 0.0206 | 4.28302E-06 | 0.018 | 0.045 | 0.6838 |
| rs1556659 | Low hand grip strength | Two-hour glucose challenge | -0.0388 | 0.0076 | 3.47896E-07 | 0.011 | 0.021 | 0.598401 |
| rs1635527 | Low hand grip strength | Two-hour glucose challenge | -0.038 | 0.0074 | 2.87899E-07 | -0.026 | 0.019 | 0.1704 |
| rs16892234 | Low hand grip strength | Two-hour glucose challenge | -0.0553 | 0.0121 | 4.45903E-06 | -0.028 | 0.031 | 0.3675 |
| rs17488051 | Low hand grip strength | Two-hour glucose challenge | -0.0361 | 0.0079 | 4.71498E-06 | -0.038 | 0.021 | 0.0608205 |
| rs2371292 | Low hand grip strength | Two-hour glucose challenge | -0.0579 | 0.0122 | 1.96101E-06 | -0.0004 | 0.032 | 0.9906 |
| rs263292 | Low hand grip strength | Two-hour glucose challenge | 0.0344 | 0.0075 | 4.83905E-06 | -0.019 | 0.02 | 0.352 |
| rs2723521 | Low hand grip strength | Two-hour glucose challenge | -0.0365 | 0.0076 | 1.68702E-06 | -0.03 | 0.019 | 0.113 |
| rs2899611 | Low hand grip strength | Two-hour glucose challenge | 0.0431 | 0.0074 | 6.01395E-09 | 0.032 | 0.019 | 0.088959 |
| rs3118903 | Low hand grip strength | Two-hour glucose challenge | 0.0575 | 0.0088 | 6.71429E-11 | 0.042 | 0.022 | 0.0577006 |
| rs326217 | Low hand grip strength | Two-hour glucose challenge | 0.0375 | 0.008 | 2.78798E-06 | -0.014 | 0.02 | 0.4742 |
| rs34464763 | Low hand grip strength | Two-hour glucose challenge | 0.0544 | 0.0086 | 3.15203E-10 | -0.034 | 0.019 | 0.0788806 |
| rs4297869 | Low hand grip strength | Two-hour glucose challenge | 0.035 | 0.0075 | 2.94002E-06 | 0.014 | 0.019 | 0.4724 |
| rs4392169 | Low hand grip strength | Two-hour glucose challenge | -0.0418 | 0.0089 | 2.63603E-06 | 0.037 | 0.023 | 0.1069 |
| rs55941591 | Low hand grip strength | Two-hour glucose challenge | -0.0543 | 0.0114 | 2.01201E-06 | -0.0028 | 0.028 | 0.9215 |
| rs577267 | Low hand grip strength | Two-hour glucose challenge | -0.039 | 0.0079 | 8.57097E-07 | -0.029 | 0.021 | 0.1769 |
| rs62102286 | Low hand grip strength | Two-hour glucose challenge | -0.0487 | 0.0074 | 5.48909E-11 | -0.017 | 0.019 | 0.384 |
| rs6906899 | Low hand grip strength | Two-hour glucose challenge | 0.0348 | 0.0074 | 2.64899E-06 | -0.0053 | 0.02 | 0.787501 |
| rs7185040 | Low hand grip strength | Two-hour glucose challenge | 0.052 | 0.0096 | 5.57006E-08 | -0.044 | 0.033 | 0.1711 |
| rs72815799 | Low hand grip strength | Two-hour glucose challenge | 0.0477 | 0.0098 | 0.000001102 | -0.044 | 0.024 | 0.0650399 |
| rs7624084 | Low hand grip strength | Two-hour glucose challenge | -0.0428 | 0.0074 | 8.50707E-09 | -0.013 | 0.019 | 0.482 |
| rs7740188 | Low hand grip strength | Two-hour glucose challenge | 0.0396 | 0.0081 | 9.8951E-07 | 0.048 | 0.021 | 0.0235001 |
| rs7967622 | Low hand grip strength | Two-hour glucose challenge | 0.0392 | 0.0078 | 4.94299E-07 | 0.055 | 0.02 | 0.00658203 |
| rs8061064 | Low hand grip strength | Two-hour glucose challenge | 0.0407 | 0.0074 | 3.54601E-08 | 0.0035 | 0.018 | 0.8509 |
| rs823130 | Low hand grip strength | Two-hour glucose challenge | 0.0341 | 0.0074 | 4.25902E-06 | 0.0015 | 0.019 | 0.9368 |
| rs9309884 | Low hand grip strength | Two-hour glucose challenge | -0.0388 | 0.0075 | 2.25102E-07 | -0.012 | 0.02 | 0.5486 |
| rs958685 | Low hand grip strength | Two-hour glucose challenge | -0.0428 | 0.0074 | 6.51793E-09 | -0.045 | 0.018 | 0.0146501 |
| rs10070428 | Low hand grip strength | Type 2 diabetes | 0.0397 | 5 | 7.24002E-08 | -0.0023 | 0.0064 | 0.7188 |
| rs1017323 | Low hand grip strength | Type 2 diabetes | -0.0537 | 2 | 4.21105E-06 | -0.017 | 0.0098 | 0.08412 |
| rs1056074 | Low hand grip strength | Type 2 diabetes | 0.0461 | 2 | 1.57801E-06 | 0.0101 | 0.0086 | 0.2394 |
| rs10940166 | Low hand grip strength | Type 2 diabetes | 0.0364 | 5 | 0.000001236 | 0.0063 | 0.0065 | 0.3318 |
| rs10952289 | Low hand grip strength | Type 2 diabetes | -0.0435 | 7 | 2.10402E-08 | 0.0346 | 0.0067 | 2.424E-07 |
| rs11236213 | Low hand grip strength | Type 2 diabetes | -0.0504 | 11 | 3.01099E-10 | -0.0113 | 0.0069 | 0.102 |
| rs112689121 | Low hand grip strength | Type 2 diabetes | 0.0479 | 18 | 1.56498E-06 | 0.0172 | 0.0089 | 0.05328 |
| rs12140813 | Low hand grip strength | Type 2 diabetes | 0.0511 | 1 | 4.76299E-08 | -0.0163 | 0.0083 | 0.04877 |
| rs12520233 | Low hand grip strength | Type 2 diabetes | 0.036 | 5 | 0.000001096 | -0.0019 | 0.0064 | 0.7661 |
| rs12790261 | Low hand grip strength | Type 2 diabetes | 0.0685 | 11 | 3.41602E-07 | -0.0057 | 0.013 | 0.6606 |
| rs13023281 | Low hand grip strength | Type 2 diabetes | 0.042 | 2 | 2.08502E-06 | -0.0078 | 0.0093 | 0.4026 |
| rs13107325 | Low hand grip strength | Type 2 diabetes | 0.0897 | 4 | 7.41822E-11 | 0.046 | 0.0135 | 0.0006598 |
| rs143384 | Low hand grip strength | Type 2 diabetes | -0.0545 | 20 | 4.47301E-13 | 0.01 | 0.0065 | 0.1234 |
| rs143459567 | Low hand grip strength | Type 2 diabetes | 0.1185 | 16 | 3.40502E-10 | -0.0076 | 0.0166 | 0.648 |
| rs145933237 | Low hand grip strength | Type 2 diabetes | 0.1344 | 3 | 2.03798E-06 | 0.0338 | 0.0266 | 0.2037 |
| rs151049793 | Low hand grip strength | Type 2 diabetes | 0.1357 | 8 | 3.00698E-06 | 0.0186 | 0.0265 | 0.4826 |
| rs1556659 | Low hand grip strength | Type 2 diabetes | -0.0388 | 10 | 3.47896E-07 | -0.0024 | 0.0062 | 0.6976 |
| rs1635527 | Low hand grip strength | Type 2 diabetes | -0.038 | 12 | 2.87899E-07 | -0.0072 | 0.0064 | 0.2596 |
| rs16892234 | Low hand grip strength | Type 2 diabetes | -0.0553 | 8 | 4.45903E-06 | -0.018 | 0.0102 | 0.07634 |
| rs17488051 | Low hand grip strength | Type 2 diabetes | -0.0361 | 11 | 4.71498E-06 | -0.0066 | 0.0069 | 0.3395 |
| rs17743567 | Low hand grip strength | Type 2 diabetes | -0.0965 | 8 | 3.57001E-06 | -0.002 | 0.0168 | 0.905 |
| rs1989803 | Low hand grip strength | Type 2 diabetes | 0.0386 | 17 | 6.38602E-07 | 0.0088 | 0.0067 | 0.1891 |
| rs2293955 | Low hand grip strength | Type 2 diabetes | 0.0568 | 8 | 2.98497E-06 | 0.0117 | 0.0106 | 0.2686 |
| rs2371292 | Low hand grip strength | Type 2 diabetes | -0.0579 | 14 | 1.96101E-06 | 0.0038 | 0.011 | 0.7296 |
| rs263292 | Low hand grip strength | Type 2 diabetes | 0.0344 | 5 | 4.83905E-06 | 0.0183 | 0.0066 | 0.005532 |
| rs2723521 | Low hand grip strength | Type 2 diabetes | -0.0365 | 7 | 1.68702E-06 | 0.0029 | 0.0066 | 0.6602 |
| rs2899611 | Low hand grip strength | Type 2 diabetes | 0.0431 | 15 | 6.01395E-09 | 0.0057 | 0.0064 | 0.3721 |
| rs3025020 | Low hand grip strength | Type 2 diabetes | -0.0386 | 6 | 3.75803E-06 | 0.0133 | 0.0075 | 0.07769 |
| rs3118903 | Low hand grip strength | Type 2 diabetes | 0.0575 | 13 | 6.71429E-11 | 0.0165 | 0.0076 | 0.03087 |
| rs326217 | Low hand grip strength | Type 2 diabetes | 0.0375 | 11 | 2.78798E-06 | 0.0149 | 0.007 | 0.03367 |
| rs34415150 | Low hand grip strength | Type 2 diabetes | 0.0833 | 6 | 4.42181E-17 | 0.0925 | 0.0103 | 1.971E-19 |
| rs35661497 | Low hand grip strength | Type 2 diabetes | 0.0701 | 2 | 1.88899E-06 | 0.0237 | 0.0128 | 0.06354 |
| rs4292598 | Low hand grip strength | Type 2 diabetes | -0.0415 | 7 | 0.00000126 | -0.0049 | 0.0077 | 0.5271 |
| rs4297869 | Low hand grip strength | Type 2 diabetes | 0.035 | 2 | 2.94002E-06 | -0.0003 | 0.0065 | 0.9631 |
| rs4392169 | Low hand grip strength | Type 2 diabetes | -0.0418 | 18 | 2.63603E-06 | 0.0114 | 0.0077 | 0.1412 |
| rs55941591 | Low hand grip strength | Type 2 diabetes | -0.0543 | 1 | 2.01201E-06 | -0.0001 | 0.0096 | 0.9917 |
| rs577267 | Low hand grip strength | Type 2 diabetes | -0.039 | 6 | 8.57097E-07 | -0.0027 | 0.0071 | 0.7045 |
| rs62102286 | Low hand grip strength | Type 2 diabetes | -0.0487 | 18 | 5.48909E-11 | 0.0088 | 0.0065 | 0.1752 |
| rs62333225 | Low hand grip strength | Type 2 diabetes | 0.0407 | 5 | 4.33701E-06 | 0.0068 | 0.0076 | 0.3736 |
| rs6906899 | Low hand grip strength | Type 2 diabetes | 0.0348 | 6 | 2.64899E-06 | 0.0164 | 0.0064 | 0.01024 |
| rs7185040 | Low hand grip strength | Type 2 diabetes | 0.052 | 16 | 5.57006E-08 | -0.0212 | 0.009 | 0.01855 |
| rs72815799 | Low hand grip strength | Type 2 diabetes | 0.0477 | 17 | 0.000001102 | -0.0023 | 0.0087 | 0.7913 |
| rs7624084 | Low hand grip strength | Type 2 diabetes | -0.0428 | 3 | 8.50707E-09 | -0.0274 | 0.0064 | 0.00001786 |
| rs76652726 | Low hand grip strength | Type 2 diabetes | 0.0361 | 9 | 1.76799E-06 | 0.0069 | 0.0065 | 0.2878 |
| rs7740188 | Low hand grip strength | Type 2 diabetes | 0.0396 | 6 | 9.8951E-07 | 0.0192 | 0.007 | 0.006201 |
| rs7967622 | Low hand grip strength | Type 2 diabetes | 0.0392 | 12 | 4.94299E-07 | -0.0101 | 0.0068 | 0.1378 |
| rs79723785 | Low hand grip strength | Type 2 diabetes | 0.1674 | 19 | 1.15899E-08 | -0.0469 | 0.0254 | 0.06528 |
| rs8061064 | Low hand grip strength | Type 2 diabetes | 0.0407 | 16 | 3.54601E-08 | -0.0204 | 0.0064 | 0.001403 |
| rs823130 | Low hand grip strength | Type 2 diabetes | 0.0341 | 1 | 4.25902E-06 | -0.0217 | 0.0065 | 0.0008294 |
| rs9309884 | Low hand grip strength | Type 2 diabetes | -0.0388 | 3 | 2.25102E-07 | -0.0028 | 0.0065 | 0.6662 |
| rs9579628 | Low hand grip strength | Type 2 diabetes | -0.0444 | 13 | 2.90898E-06 | -0.0008 | 0.0085 | 0.9248 |
| rs958685 | Low hand grip strength | Type 2 diabetes | -0.0428 | 2 | 6.51793E-09 | -0.0064 | 0.0064 | 0.3163 |
| rs9830552 | Low hand grip strength | Type 2 diabetes | -0.0351 | 3 | 2.97797E-06 | -0.0071 | 0.0065 | 0.2741 |
| rs9902761 | Low hand grip strength | Type 2 diabetes | -0.0446 | 17 | 1.63999E-07 | -0.0226 | 0.0074 | 0.002365 |

**Supplementary Table 2.** Genome-wide significant SNPs for ALM

| **SNP** | **exposure** | **outcome** | **beta.exposure** | **se.exposure** | **pval.exposure** | **beta.outcome** | **se.outcome** | **pval.outcome** |
| --- | --- | --- | --- | --- | --- | --- | --- | --- |
| rs10005035 | ALM | Fasting glucose | -0.0175 | 12865684 | 0.0021 | 0.0028 | 0.002 | 0.0888894 |
| rs10019221 | ALM | Fasting glucose | -0.0124 | 21785364 | 0.0019 | -0.002 | 0.0019 | 0.2517 |
| rs10036789 | ALM | Fasting glucose | 0.0163 | 71695918 | 0.0019 | 0.0001 | 0.0018 | 0.9127 |
| rs1005723 | ALM | Fasting glucose | 0.0161 | 243646251 | 0.0024 | 0.002 | 0.0024 | 0.4111 |
| rs10068640 | ALM | Fasting glucose | 0.0112 | 123981977 | 0.002 | -0.0006 | 0.0019 | 0.8104 |
| rs10075249 | ALM | Fasting glucose | 0.0143 | 52846505 | 0.0019 | 0.0007 | 0.0018 | 0.9345 |
| rs10107388 | ALM | Fasting glucose | -0.0159 | 145004944 | 0.002 | -0.0026 | 0.002 | 0.0725705 |
| rs10112506 | ALM | Fasting glucose | -0.012 | 13164746 | 0.0019 | -0.0016 | 0.0019 | 0.3373 |
| rs10123619 | ALM | Fasting glucose | -0.0171 | 119353611 | 0.0026 | -0.0001 | 0.0025 | 0.546 |
| rs10128333 | ALM | Fasting glucose | -0.0146 | 64570038 | 0.0025 | -0.0007 | 0.0024 | 0.9665 |
| rs10171272 | ALM | Fasting glucose | 0.0136 | 25946636 | 0.002 | 0.0038 | 0.0073 | 0.5811 |
| rs10202701 | ALM | Fasting glucose | 0.0227 | 232328681 | 0.0019 | 0.0014 | 0.0018 | 0.443 |
| rs10202845 | ALM | Fasting glucose | -0.0288 | 42575820 | 0.003 | 0.0004 | 0.0027 | 0.756201 |
| rs10203320 | ALM | Fasting glucose | 0.0138 | 9771620 | 0.002 | -0.0051 | 0.0021 | 0.009378 |
| rs10203386 | ALM | Fasting glucose | -0.0238 | 25136866 | 0.0019 | -0.0033 | 0.0018 | 0.0517595 |
| rs10205141 | ALM | Fasting glucose | 0.0241 | 11313340 | 0.0044 | -0.0026 | 0.0039 | 0.9058 |
| rs10221831 | ALM | Fasting glucose | 0.03 | 202107829 | 0.0053 | 0.0047 | 0.0048 | 0.497299 |
| rs10225945 | ALM | Fasting glucose | -0.0146 | 28250083 | 0.0026 | 0.0057 | 0.0023 | 0.00702199 |
| rs10242866 | ALM | Fasting glucose | 0.0157 | 17920613 | 0.0019 | 0.003 | 0.0019 | 0.1826 |
| rs10283100 | ALM | Fasting glucose | 0.0575 | 120596023 | 0.0041 | 0.0085 | 0.0046 | 0.2217 |
| rs1035583 | ALM | Fasting glucose | 0.0148 | 207326937 | 0.0019 | 0.0001 | 0.0019 | 0.5052 |
| rs10421750 | ALM | Fasting glucose | -0.0145 | 50290604 | 0.0021 | -0.0025 | 0.0074 | 0.6975 |
| rs10453441 | ALM | Fasting glucose | -0.0139 | 46363739 | 0.002 | -0.0037 | 0.0027 | 0.2642 |
| rs10461725 | ALM | Fasting glucose | 0.0134 | 39437129 | 0.002 | -0.0019 | 0.002 | 0.4481 |
| rs10471339 | ALM | Fasting glucose | -0.011 | 67823773 | 0.0019 | -0.0021 | 0.0019 | 0.0930894 |
| rs1047891 | ALM | Fasting glucose | 0.0233 | 211540507 | 0.002 | 0.0035 | 0.0018 | 0.084351 |
| rs10483727 | ALM | Fasting glucose | -0.0368 | 61072875 | 0.0019 | 0.0011 | 0.0017 | 0.4507 |
| rs1056747 | ALM | Fasting glucose | -0.0155 | 35690102 | 0.0019 | 0.0027 | 0.0019 | 0.4395 |
| rs1063582 | ALM | Fasting glucose | -0.0185 | 23167353 | 0.0022 | 0.0002 | 0.0022 | 0.730801 |
| rs10657263 | ALM | Fasting glucose | -0.013 | 49690460 | 0.0019 | -0.0052 | 0.002 | 0.00423399 |
| rs10748128 | ALM | Fasting glucose | 0.0255 | 69827658 | 0.002 | 0.0025 | 0.0018 | 0.1319 |
| rs10749157 | ALM | Fasting glucose | 0.0113 | 115780129 | 0.002 | -0.0015 | 0.0018 | 0.6027 |
| rs10776560 | ALM | Fasting glucose | -0.0157 | 50542358 | 0.0019 | -0.0009 | 0.0018 | 0.3957 |
| rs10793931 | ALM | Fasting glucose | -0.0132 | 133436478 | 0.002 | 0.0004 | 0.0019 | 0.8558 |
| rs10796828 | ALM | Fasting glucose | 0.0154 | 69490346 | 0.002 | 0.0016 | 0.002 | 0.5608 |
| rs10807137 | ALM | Fasting glucose | -0.0455 | 34183026 | 0.0025 | 0.0026 | 0.0025 | 0.3437 |
| rs10815274 | ALM | Fasting glucose | 0.0124 | 5728968 | 0.0019 | -0.001 | 0.0018 | 0.9326 |
| rs10822117 | ALM | Fasting glucose | -0.0176 | 52786701 | 0.0022 | -0.001 | 0.0021 | 0.9625 |
| rs10824307 | ALM | Fasting glucose | -0.0194 | 77185310 | 0.002 | -0.0028 | 0.002 | 0.1229 |
| rs10829226 | ALM | Fasting glucose | -0.0112 | 27573952 | 0.002 | 0.002 | 0.002 | 0.2658 |
| rs10832963 | ALM | Fasting glucose | -0.0203 | 18664241 | 0.0022 | 0.0011 | 0.002 | 0.5215 |
| rs10845408 | ALM | Fasting glucose | 0.0255 | 11880581 | 0.002 | -0.0008 | 0.0019 | 0.8967 |
| rs10858246 | ALM | Fasting glucose | -0.0188 | 139102831 | 0.002 | 0.0019 | 0.0021 | 0.5006 |
| rs10864899 | ALM | Fasting glucose | -0.0112 | 112929481 | 0.0019 | 0.0019 | 0.0019 | 0.2225 |
| rs10922475 | ALM | Fasting glucose | 0.0159 | 89142142 | 0.0019 | -0.0028 | 0.0018 | 0.1624 |
| rs10948 | ALM | Fasting glucose | -0.0252 | 10754905 | 0.002 | 0.0007 | 0.0019 | 0.3111 |
| rs10962212 | ALM | Fasting glucose | 0.0143 | 15911745 | 0.0019 | -0.0009 | 0.0018 | 0.7013 |
| rs10975935 | ALM | Fasting glucose | -0.0121 | 6954579 | 0.0022 | -0.0036 | 0.0021 | 0.0213801 |
| rs10982888 | ALM | Fasting glucose | -0.0328 | 118468947 | 0.003 | 0.0033 | 0.0031 | 0.5253 |
| rs11009928 | ALM | Fasting glucose | -0.0148 | 35058712 | 0.0022 | -0.0141 | 0.0076 | 0.0777302 |
| rs11014285 | ALM | Fasting glucose | 0.0342 | 25178864 | 0.0026 | 0.0046 | 0.0027 | 0.0710706 |
| rs11042717 | ALM | Fasting glucose | -0.029 | 10303939 | 0.0019 | 0.0008 | 0.0016 | 0.491801 |
| rs11049704 | ALM | Fasting glucose | -0.0183 | 28691701 | 0.0021 | -0.0035 | 0.0065 | 0.5551 |
| rs11060942 | ALM | Fasting glucose | -0.0354 | 123434524 | 0.0052 | 0.0077 | 0.0049 | 0.0616595 |
| rs11068230 | ALM | Fasting glucose | 0.0238 | 117349014 | 0.0028 | -0.0044 | 0.0029 | 0.1212 |
| rs11070842 | ALM | Fasting glucose | -0.0146 | 51624185 | 0.0026 | -0.0008 | 0.0025 | 0.7115 |
| rs11098677 | ALM | Fasting glucose | -0.0263 | 123833516 | 0.0023 | 0.0029 | 0.0022 | 0.1688 |
| rs11121615 | ALM | Fasting glucose | -0.0202 | 10825577 | 0.002 | -0.0008 | 0.0021 | 0.8326 |
| rs111365325 | ALM | Fasting glucose | -0.0271 | 170865229 | 0.0022 | 0.001 | 0.0023 | 0.8265 |
| rs111622870 | ALM | Fasting glucose | -0.0282 | 2613109 | 0.0044 | -0.0099 | 0.0049 | 0.0535402 |
| rs11175919 | ALM | Fasting glucose | 0.0349 | 66180277 | 0.0059 | -0.0009 | 0.0057 | 1 |
| rs11178643 | ALM | Fasting glucose | 0.0109 | 71522437 | 0.002 | -0.0063 | 0.0071 | 0.4752 |
| rs11187838 | ALM | Fasting glucose | 0.0394 | 96038686 | 0.0019 | 0.0012 | 0.0018 | 0.9775 |
| rs111901094 | ALM | Fasting glucose | -0.0253 | 19513570 | 0.0025 | 0.0132 | 0.0101 | 0.2184 |
| rs11191208 | ALM | Fasting glucose | 0.0147 | 103838497 | 0.0024 | -0.0003 | 0.0023 | 0.797 |
| rs11198591 | ALM | Fasting glucose | 0.0148 | 120515892 | 0.002 | -0.0009 | 0.0017 | 0.573 |
| rs112021215 | ALM | Fasting glucose | -0.0147 | 62867186 | 0.0025 | 0.0039 | 0.0029 | 0.5611 |
| rs11210892 | ALM | Fasting glucose | 0.0118 | 44100084 | 0.002 | 0.0014 | 0.0019 | 0.2757 |
| rs112153300 | ALM | Fasting glucose | 0.0261 | 47547474 | 0.0034 | -0.0028 | 0.0035 | 0.556 |
| rs11217863 | ALM | Fasting glucose | -0.0268 | 120293138 | 0.003 | 0.0066 | 0.0031 | 0.0504905 |
| rs11221657 | ALM | Fasting glucose | 0.0179 | 129181358 | 0.0028 | 0.0041 | 0.0027 | 0.0507096 |
| rs11233117 | ALM | Fasting glucose | -0.0176 | 69924352 | 0.0019 | -0.0009 | 0.0019 | 0.709 |
| rs11243202 | ALM | Fasting glucose | 0.0302 | 7719065 | 0.0019 | -0.0014 | 0.0018 | 0.3824 |
| rs112537273 | ALM | Fasting glucose | -0.0212 | 38248306 | 0.0022 | 0.0036 | 0.0022 | 0.0413704 |
| rs11260035 | ALM | Fasting glucose | 0.015 | 7898957 | 0.0021 | 0.0046 | 0.0021 | 0.01661 |
| rs11260623 | ALM | Fasting glucose | 0.0117 | 1781456 | 0.0019 | -0.0005 | 0.0019 | 0.3937 |
| rs112873218 | ALM | Fasting glucose | 0.0216 | 1960119 | 0.0031 | 0.0083 | 0.0035 | 0.0316199 |
| rs113107560 | ALM | Fasting glucose | -0.019 | 36747842 | 0.0019 | -0.0015 | 0.007 | 0.9852 |
| rs113146332 | ALM | Fasting glucose | 0.0311 | 42565977 | 0.0049 | -0.005 | 0.0054 | 0.5048 |
| rs113232639 | ALM | Fasting glucose | 0.0327 | 20715656 | 0.0019 | -0.0002 | 0.002 | 0.8244 |
| rs113289555 | ALM | Fasting glucose | -0.0206 | 996998 | 0.0023 | 0.0054 | 0.0029 | 0.1322 |
| rs113671109 | ALM | Fasting glucose | -0.015 | 12620885 | 0.0023 | -0.0003 | 0.0021 | 0.8143 |
| rs113827862 | ALM | Fasting glucose | -0.0235 | 89849527 | 0.004 | -0.0092 | 0.0038 | 0.00436204 |
| rs113898003 | ALM | Fasting glucose | -0.036 | 130341235 | 0.0021 | -0.005 | 0.0023 | 0.08337 |
| rs115010283 | ALM | Fasting glucose | 0.034 | 172162393 | 0.002 | -0.0054 | 0.0072 | 0.4806 |
| rs115105539 | ALM | Fasting glucose | 0.0231 | 49409496 | 0.0025 | -0.0002 | 0.009 | 0.9846 |
| rs11562101 | ALM | Fasting glucose | 0.0115 | 56059458 | 0.002 | -0.002 | 0.0029 | 0.5033 |
| rs11580040 | ALM | Fasting glucose | 0.0325 | 155198222 | 0.0035 | 0.0076 | 0.0038 | 0.0585599 |
| rs11590254 | ALM | Fasting glucose | 0.0186 | 92316573 | 0.002 | 0.0018 | 0.0019 | 0.169 |
| rs115912456 | ALM | Fasting glucose | 0.0577 | 82815158 | 0.0047 | 0.0003 | 0.0048 | 0.8403 |
| rs116008080 | ALM | Fasting glucose | -0.0415 | 67254841 | 0.0063 | -0.0047 | 0.0074 | 0.8196 |
| rs116052377 | ALM | Fasting glucose | 0.0225 | 124787756 | 0.0035 | -0.0025 | 0.0035 | 0.712901 |
| rs11605297 | ALM | Fasting glucose | 0.0146 | 58296806 | 0.0022 | -0.001 | 0.0021 | 0.573 |
| rs116092985 | ALM | Fasting glucose | -0.0401 | 2160973 | 0.0033 | 0.0011 | 0.0046 | 0.662 |
| rs11612462 | ALM | Fasting glucose | 0.015 | 104411368 | 0.0025 | -0.002 | 0.0024 | 0.2771 |
| rs11629593 | ALM | Fasting glucose | -0.0109 | 96033696 | 0.002 | -0.0016 | 0.0021 | 0.3359 |
| rs11633371 | ALM | Fasting glucose | 0.0216 | 89356832 | 0.0019 | 0.0008 | 0.0018 | 0.6027 |
| rs116339650 | ALM | Fasting glucose | -0.0175 | 26200972 | 0.0029 | 0.0055 | 0.0027 | 0.02467 |
| rs116493405 | ALM | Fasting glucose | 0.0287 | 114733556 | 0.0042 | 0.0034 | 0.0042 | 0.6531 |
| rs11672848 | ALM | Fasting glucose | -0.0171 | 37570704 | 0.0019 | -0.0008 | 0.0019 | 0.6893 |
| rs11684531 | ALM | Fasting glucose | -0.0172 | 219835489 | 0.0028 | 0.0011 | 0.0062 | 0.9556 |
| rs1168768 | ALM | Fasting glucose | 0.0332 | 66509650 | 0.006 | 0.002 | 0.0074 | 0.7185 |
| rs116919274 | ALM | Fasting glucose | 0.0271 | 17359808 | 0.0046 | 0.0033 | 0.0056 | 0.59 |
| rs117068593 | ALM | Fasting glucose | 0.0403 | 93118229 | 0.0024 | 0.0035 | 0.0025 | 0.184 |
| rs117203652 | ALM | Fasting glucose | -0.0346 | 49857801 | 0.0055 | 0.006 | 0.0073 | 0.2203 |
| rs11720869 | ALM | Fasting glucose | 0.0141 | 185619716 | 0.002 | 0.0013 | 0.002 | 0.533301 |
| rs11721522 | ALM | Fasting glucose | 0.0106 | 156976051 | 0.0019 | 0.0038 | 0.0019 | 0.0549604 |
| rs11727162 | ALM | Fasting glucose | -0.017 | 88606761 | 0.0019 | -0.0021 | 0.0018 | 0.3979 |
| rs117335233 | ALM | Fasting glucose | -0.0236 | 79914330 | 0.0042 | -0.0037 | 0.0052 | 0.7718 |
| rs1177765 | ALM | Fasting glucose | -0.0232 | 32829929 | 0.0019 | 0.0018 | 0.0017 | 0.2955 |
| rs11778491 | ALM | Fasting glucose | -0.0247 | 120451362 | 0.0022 | 0.0018 | 0.0021 | 0.2801 |
| rs117818446 | ALM | Fasting glucose | 0.0423 | 67223589 | 0.0068 | 0.0022 | 0.0083 | 0.512201 |
| rs117972846 | ALM | Fasting glucose | 0.0335 | 26947476 | 0.0057 | -0.0015 | 0.0061 | 0.8824 |
| rs11867855 | ALM | Fasting glucose | -0.0132 | 18262584 | 0.0022 | 0.001 | 0.0021 | 0.281 |
| rs1190540 | ALM | Fasting glucose | 0.0125 | 102897009 | 0.0021 | -0.0023 | 0.002 | 0.3761 |
| rs11959466 | ALM | Fasting glucose | 0.038 | 42803824 | 0.0042 | -0.004 | 0.0047 | 0.3188 |
| rs1202186 | ALM | Fasting glucose | -0.012 | 87213258 | 0.002 | 0.0015 | 0.0019 | 0.4362 |
| rs12051245 | ALM | Fasting glucose | 0.0299 | 783865 | 0.0022 | 0.0011 | 0.0023 | 0.2383 |
| rs12074850 | ALM | Fasting glucose | 0.0393 | 51248316 | 0.0033 | -0.0099 | 0.0033 | 0.000524699 |
| rs12099669 | ALM | Fasting glucose | 0.0331 | 46783653 | 0.002 | -0.0031 | 0.0019 | 0.0967008 |
| rs12150907 | ALM | Fasting glucose | -0.0219 | 4940630 | 0.0024 | 0.0033 | 0.0024 | 0.1536 |
| rs12185775 | ALM | Fasting glucose | -0.0167 | 20293769 | 0.003 | -0.0004 | 0.0033 | 0.9252 |
| rs12188208 | ALM | Fasting glucose | -0.0195 | 77442791 | 0.0022 | -0.0068 | 0.0021 | 0.002452 |
| rs12230946 | ALM | Fasting glucose | 0.0271 | 53498725 | 0.0033 | 0.0001 | 0.003 | 0.9448 |
| rs12334478 | ALM | Fasting glucose | -0.0161 | 141998765 | 0.0019 | 0.0002 | 0.0018 | 0.742299 |
| rs12340775 | ALM | Fasting glucose | -0.0287 | 13226945 | 0.0043 | -0.0009 | 0.0039 | 0.5365 |
| rs12344515 | ALM | Fasting glucose | -0.0163 | 113801231 | 0.0022 | 0.0034 | 0.0022 | 0.1421 |
| rs12347137 | ALM | Fasting glucose | -0.046 | 119122721 | 0.0024 | 0.0051 | 0.0022 | 0.01034 |
| rs12351226 | ALM | Fasting glucose | 0.0218 | 98405230 | 0.0025 | 0.0036 | 0.0025 | 0.1711 |
| rs12423821 | ALM | Fasting glucose | 0.0161 | 132650284 | 0.0027 | -0.0019 | 0.0036 | 0.8768 |
| rs12461874 | ALM | Fasting glucose | -0.0181 | 17180358 | 0.0021 | -0.0001 | 0.0021 | 0.9808 |
| rs12483401 | ALM | Fasting glucose | -0.0387 | 35443829 | 0.0067 | -0.002 | 0.0065 | 0.8705 |
| rs12512942 | ALM | Fasting glucose | -0.0162 | 177766307 | 0.002 | 0.0017 | 0.0019 | 0.9363 |
| rs12517711 | ALM | Fasting glucose | -0.0147 | 60754661 | 0.0019 | 0.003 | 0.0019 | 0.0984011 |
| rs12519407 | ALM | Fasting glucose | 0.0181 | 137651012 | 0.0022 | 0.0018 | 0.002 | 0.729899 |
| rs12533452 | ALM | Fasting glucose | 0.0237 | 19016871 | 0.0026 | 0.0021 | 0.0025 | 0.4301 |
| rs12536902 | ALM | Fasting glucose | 0.0479 | 33213009 | 0.0081 | -0.014 | 0.0083 | 0.2117 |
| rs12541381 | ALM | Fasting glucose | -0.0319 | 135649848 | 0.0022 | 0.0052 | 0.0023 | 0.0217801 |
| rs12563442 | ALM | Fasting glucose | 0.0122 | 19786695 | 0.0021 | 0.0026 | 0.002 | 0.2086 |
| rs1260326 | ALM | Fasting glucose | 0.0323 | 27730940 | 0.0019 | 0.0282 | 0.0017 | 4.48126E-65 |
| rs12616192 | ALM | Fasting glucose | -0.0261 | 121568931 | 0.0038 | -0.0036 | 0.0041 | 0.1857 |
| rs12655296 | ALM | Fasting glucose | -0.011 | 15890643 | 0.002 | 0.0002 | 0.0019 | 0.709 |
| rs12672217 | ALM | Fasting glucose | 0.0139 | 156310948 | 0.002 | -0.0004 | 0.0019 | 0.9379 |
| rs12700901 | ALM | Fasting glucose | -0.0184 | 28783171 | 0.0019 | 0.0012 | 0.0019 | 0.6125 |
| rs12702693 | ALM | Fasting glucose | 0.0173 | 8101039 | 0.0019 | -0.0008 | 0.0018 | 0.3807 |
| rs12713004 | ALM | Fasting glucose | 0.0367 | 23896049 | 0.0021 | -0.0018 | 0.0021 | 0.5006 |
| rs12724708 | ALM | Fasting glucose | 0.0243 | 219620569 | 0.002 | 0.0021 | 0.0018 | 0.1624 |
| rs12773500 | ALM | Fasting glucose | 0.0171 | 81232632 | 0.0028 | 0.0003 | 0.0025 | 0.8467 |
| rs12831751 | ALM | Fasting glucose | 0.0172 | 29520017 | 0.0021 | -0.0007 | 0.002 | 0.4331 |
| rs12882130 | ALM | Fasting glucose | -0.0202 | 103878774 | 0.002 | -0.0028 | 0.002 | 0.1495 |
| rs12907139 | ALM | Fasting glucose | -0.0149 | 73521566 | 0.0019 | -0.0034 | 0.0018 | 0.2718 |
| rs1290786 | ALM | Fasting glucose | -0.0143 | 169097381 | 0.0019 | 0.0015 | 0.0016 | 0.4732 |
| rs12909863 | ALM | Fasting glucose | 0.0189 | 75825822 | 0.0022 | -0.0048 | 0.002 | 0.0204602 |
| rs1291114 | ALM | Fasting glucose | 0.0173 | 35500850 | 0.0031 | -0.0017 | 0.0035 | 0.674 |
| rs12926103 | ALM | Fasting glucose | 0.0272 | 86371775 | 0.0038 | 0.0007 | 0.0038 | 0.6991 |
| rs12943867 | ALM | Fasting glucose | 0.0184 | 79409710 | 0.002 | -0.002 | 0.0025 | 0.4325 |
| rs12962050 | ALM | Fasting glucose | 0.0153 | 35179808 | 0.002 | -0.0008 | 0.0019 | 0.8153 |
| rs12997625 | ALM | Fasting glucose | -0.017 | 202970250 | 0.0019 | 0.0053 | 0.0018 | 0.0153801 |
| rs13037813 | ALM | Fasting glucose | 0.0292 | 47750588 | 0.0022 | -0.005 | 0.0021 | 0.1034 |
| rs13103161 | ALM | Fasting glucose | -0.0284 | 106216459 | 0.0019 | 0.0003 | 0.0019 | 0.9787 |
| rs13109280 | ALM | Fasting glucose | 0.0131 | 54380513 | 0.002 | -0.0004 | 0.002 | 0.8994 |
| rs13123591 | ALM | Fasting glucose | 0.0185 | 120105990 | 0.002 | -0.0026 | 0.0019 | 0.1771 |
| rs13127468 | ALM | Fasting glucose | -0.0123 | 8599658 | 0.0019 | 0.0002 | 0.0021 | 0.8099 |
| rs13170063 | ALM | Fasting glucose | -0.0152 | 157895013 | 0.0019 | -0.0015 | 0.0017 | 0.3389 |
| rs1319012 | ALM | Fasting glucose | -0.052 | 41852616 | 0.0037 | 0.0059 | 0.0037 | 0.2176 |
| rs13209685 | ALM | Fasting glucose | 0.0277 | 7779729 | 0.0026 | -0.002 | 0.0024 | 0.651199 |
| rs1324538 | ALM | Fasting glucose | 0.0237 | 45080144 | 0.0019 | 0.0001 | 0.002 | 0.5439 |
| rs1325596 | ALM | Fasting glucose | 0.0287 | 176794066 | 0.0019 | -0.0021 | 0.0016 | 0.1514 |
| rs1330826 | ALM | Fasting glucose | 0.0162 | 85129970 | 0.0023 | 0.0012 | 0.0023 | 0.5772 |
| rs13316 | ALM | Fasting glucose | 0.0115 | 93407301 | 0.0019 | 0.0016 | 0.002 | 0.404 |
| rs13391980 | ALM | Fasting glucose | -0.0225 | 165504841 | 0.0029 | -0.004 | 0.0025 | 0.0665902 |
| rs1340022 | ALM | Fasting glucose | 0.0118 | 131334465 | 0.0019 | 0.0019 | 0.0018 | 0.2176 |
| rs1341215 | ALM | Fasting glucose | 0.0229 | 111662350 | 0.0027 | -0.0067 | 0.0025 | 0.00922104 |
| rs13430869 | ALM | Fasting glucose | 0.0272 | 218146818 | 0.0021 | 0.0031 | 0.002 | 0.1998 |
| rs139163241 | ALM | Fasting glucose | -0.0164 | 76709213 | 0.0027 | 0.001 | 0.0033 | 0.5109 |
| rs139921635 | ALM | Fasting glucose | 0.0385 | 73181637 | 0.0062 | 0.0022 | 0.0103 | 0.6982 |
| rs140440099 | ALM | Fasting glucose | 0.0613 | 50632595 | 0.0063 | 0.0075 | 0.0074 | 0.1525 |
| rs1405227 | ALM | Fasting glucose | 0.0129 | 98873390 | 0.002 | 0.0022 | 0.002 | 0.3494 |
| rs143076454 | ALM | Fasting glucose | -0.0499 | 921179 | 0.007 | -0.0144 | 0.0113 | 0.1885 |
| rs143384 | ALM | Fasting glucose | 0.0725 | 34025756 | 0.0019 | 0.0028 | 0.0017 | 0.0454203 |
| rs143554698 | ALM | Fasting glucose | -0.0257 | 95538573 | 0.0027 | 0.0057 | 0.0029 | 0.0393396 |
| rs144109601 | ALM | Fasting glucose | -0.0278 | 50455500 | 0.0048 | 0.0004 | 0.0047 | 0.9741 |
| rs1443536 | ALM | Fasting glucose | 0.0218 | 82174165 | 0.0021 | 0.0023 | 0.0019 | 0.2744 |
| rs1444628 | ALM | Fasting glucose | 0.024 | 20563643 | 0.002 | -0.0005 | 0.002 | 0.649 |
| rs144627572 | ALM | Fasting glucose | 0.0439 | 20583907 | 0.0053 | -0.0031 | 0.0058 | 0.8949 |
| rs147110934 | ALM | Fasting glucose | -0.0722 | 55993436 | 0.0062 | 0.0016 | 0.0071 | 0.9056 |
| rs147233090 | ALM | Fasting glucose | -0.0446 | 44028047 | 0.0061 | 0.0103 | 0.0059 | 0.0437401 |
| rs1472852 | ALM | Fasting glucose | -0.0638 | 17910236 | 0.0026 | 0.0067 | 0.0026 | 0.0442996 |
| rs1478575 | ALM | Fasting glucose | 0.0312 | 218278555 | 0.002 | -0.0025 | 0.0019 | 0.1194 |
| rs14976 | ALM | Fasting glucose | 0.0144 | 85818886 | 0.002 | -0.0023 | 0.002 | 0.0431897 |
| rs1514134 | ALM | Fasting glucose | -0.0114 | 56116513 | 0.0019 | 0.0026 | 0.0018 | 0.2388 |
| rs1556659 | ALM | Fasting glucose | 0.0163 | 130834698 | 0.002 | -0.0055 | 0.0017 | 0.008077 |
| rs1584011 | ALM | Fasting glucose | 0.0159 | 27080527 | 0.002 | -0.0003 | 0.0019 | 0.7694 |
| rs165849 | ALM | Fasting glucose | 0.0157 | 19958669 | 0.0021 | 0.0054 | 0.002 | 0.01629 |
| rs16989695 | ALM | Fasting glucose | -0.0139 | 4505445 | 0.0019 | 0.0056 | 0.002 | 0.00895901 |
| rs17197114 | ALM | Fasting glucose | 0.0177 | 21894526 | 0.0025 | 0.0014 | 0.0027 | 0.5269 |
| rs17205463 | ALM | Fasting glucose | -0.0263 | 62381413 | 0.0019 | -0.0094 | 0.0017 | 8.03101E-08 |
| rs17246129 | ALM | Fasting glucose | 0.0254 | 227259964 | 0.002 | 0.003 | 0.0019 | 0.0203901 |
| rs17278379 | ALM | Fasting glucose | 0.0226 | 172381284 | 0.0029 | 0.0028 | 0.0026 | 0.2381 |
| rs1730028 | ALM | Fasting glucose | 0.0131 | 157900789 | 0.0019 | 0.0004 | 0.0018 | 0.8265 |
| rs173135 | ALM | Fasting glucose | -0.0341 | 68172326 | 0.003 | -0.006 | 0.003 | 0.0769804 |
| rs17400325 | ALM | Fasting glucose | 0.0345 | 178565913 | 0.0047 | 0.0055 | 0.0046 | 0.1986 |
| rs17408561 | ALM | Fasting glucose | 0.0123 | 225474277 | 0.002 | -0.0005 | 0.0064 | 0.9133 |
| rs17478946 | ALM | Fasting glucose | -0.0192 | 24093062 | 0.0021 | -0.0019 | 0.0021 | 0.4705 |
| rs17681189 | ALM | Fasting glucose | -0.0131 | 65976175 | 0.0019 | -0.0062 | 0.0018 | 0.000453701 |
| rs17718736 | ALM | Fasting glucose | 0.0115 | 71555205 | 0.002 | -0.0028 | 0.0019 | 0.0456604 |
| rs177591 | ALM | Fasting glucose | -0.0191 | 28556199 | 0.0027 | 0.0012 | 0.0029 | 0.9286 |
| rs17773965 | ALM | Fasting glucose | -0.0163 | 217631338 | 0.0027 | -0.0004 | 0.0025 | 0.8129 |
| rs17818592 | ALM | Fasting glucose | -0.0129 | 86088594 | 0.0019 | 0.0016 | 0.0018 | 0.2176 |
| rs1786263 | ALM | Fasting glucose | -0.019 | 13116432 | 0.0019 | 0.0027 | 0.0019 | 0.2201 |
| rs1797070 | ALM | Fasting glucose | 0.0219 | 218630201 | 0.0021 | 0.0035 | 0.002 | 0.1002 |
| rs1823217 | ALM | Fasting glucose | -0.0181 | 134380959 | 0.002 | 0.0004 | 0.0019 | 0.9586 |
| rs182798714 | ALM | Fasting glucose | 0.0376 | 128960869 | 0.0062 | -0.0138 | 0.0069 | 0.0659098 |
| rs1880318 | ALM | Fasting glucose | 0.0147 | 46028167 | 0.0024 | 0.0002 | 0.0023 | 0.8954 |
| rs188617336 | ALM | Fasting glucose | 0.0138 | 20610730 | 0.0021 | -0.009 | 0.0062 | 0.1595 |
| rs1899040 | ALM | Fasting glucose | 0.0152 | 223901896 | 0.0023 | 0.0024 | 0.0022 | 0.4197 |
| rs190823861 | ALM | Fasting glucose | -0.0345 | 73505911 | 0.0045 | 0.0101 | 0.0051 | 0.0364301 |
| rs1933081 | ALM | Fasting glucose | 0.0267 | 151651505 | 0.0034 | -0.004 | 0.0034 | 0.1673 |
| rs199647708 | ALM | Fasting glucose | 0.0114 | 125352195 | 0.0019 | -0.001 | 0.0069 | 0.876 |
| rs200439 | ALM | Fasting glucose | -0.0128 | 6716083 | 0.0023 | -0.0007 | 0.0022 | 0.8365 |
| rs2005172 | ALM | Fasting glucose | 0.048 | 61996255 | 0.002 | 0.0004 | 0.002 | 0.8794 |
| rs200739311 | ALM | Fasting glucose | -0.0128 | 85650323 | 0.002 | -0.0061 | 0.0072 | 0.5083 |
| rs201570119 | ALM | Fasting glucose | 0.0194 | 112256320 | 0.0023 | -0.0187 | 0.0082 | 0.02399 |
| rs2019203 | ALM | Fasting glucose | 0.0189 | 36908672 | 0.0019 | -0.004 | 0.0018 | 0.0705797 |
| rs2025609 | ALM | Fasting glucose | 0.0186 | 67422990 | 0.0026 | 0.0089 | 0.0025 | 0.000414295 |
| rs2025808 | ALM | Fasting glucose | 0.0122 | 184161757 | 0.0022 | -0.002 | 0.0021 | 0.665001 |
| rs2035901 | ALM | Fasting glucose | 0.024 | 145521867 | 0.0019 | 0.001 | 0.0018 | 0.8479 |
| rs2070598 | ALM | Fasting glucose | 0.0204 | 75360906 | 0.0019 | -0.0006 | 0.0018 | 0.742299 |
| rs2071450 | ALM | Fasting glucose | -0.0174 | 54428532 | 0.002 | -0.0033 | 0.0019 | 0.2103 |
| rs2089111 | ALM | Fasting glucose | -0.0172 | 91180019 | 0.0022 | -0.0043 | 0.0022 | 0.0485501 |
| rs2101017 | ALM | Fasting glucose | -0.0223 | 122306857 | 0.0028 | -0.001 | 0.0025 | 0.8282 |
| rs2105333 | ALM | Fasting glucose | -0.019 | 158755437 | 0.002 | -0.0016 | 0.0019 | 0.2985 |
| rs2112617 | ALM | Fasting glucose | -0.0167 | 46977125 | 0.0019 | 0.0044 | 0.0018 | 0.0795408 |
| rs212526 | ALM | Fasting glucose | 0.0214 | 21584941 | 0.0019 | 0.0009 | 0.0018 | 0.2176 |
| rs2138374 | ALM | Fasting glucose | -0.0149 | 190014317 | 0.002 | -0.0048 | 0.002 | 0.0260501 |
| rs2140619 | ALM | Fasting glucose | 0.0113 | 114007270 | 0.0019 | 0.0009 | 0.0018 | 0.5109 |
| rs2142331 | ALM | Fasting glucose | -0.0165 | 116636719 | 0.0019 | -0.0084 | 0.0018 | 3.20302E-06 |
| rs2142644 | ALM | Fasting glucose | -0.0181 | 19053843 | 0.002 | -0.0009 | 0.0019 | 0.6037 |
| rs2174008 | ALM | Fasting glucose | -0.0192 | 38510456 | 0.0019 | -0.0016 | 0.0019 | 0.4717 |
| rs2181834 | ALM | Fasting glucose | 0.0254 | 102661251 | 0.0019 | 0.0023 | 0.0018 | 0.4763 |
| rs2194411 | ALM | Fasting glucose | 0.0443 | 185548663 | 0.0029 | -0.0046 | 0.0026 | 0.1029 |
| rs2209098 | ALM | Fasting glucose | 0.024 | 172167226 | 0.002 | -0.0018 | 0.0019 | 0.3648 |
| rs2212926 | ALM | Fasting glucose | -0.022 | 38066883 | 0.0023 | 0.0024 | 0.003 | 0.3154 |
| rs2229840 | ALM | Fasting glucose | 0.0341 | 124826462 | 0.0026 | 0.0017 | 0.0023 | 0.453 |
| rs2230033 | ALM | Fasting glucose | -0.0265 | 39671476 | 0.0019 | 0.0045 | 0.0016 | 0.0197401 |
| rs2236096 | ALM | Fasting glucose | 0.018 | 3266319 | 0.0023 | -0.0008 | 0.0022 | 0.5303 |
| rs2236406 | ALM | Fasting glucose | 0.0394 | 98221861 | 0.002 | 0.0037 | 0.0019 | 0.091601 |
| rs2237485 | ALM | Fasting glucose | 0.0191 | 50749870 | 0.0023 | -0.0093 | 0.0021 | 1.92199E-05 |
| rs2240735 | ALM | Fasting glucose | 0.0189 | 4027605 | 0.0022 | -0.0057 | 0.0021 | 0.00914597 |
| rs2268718 | ALM | Fasting glucose | 0.0141 | 52415023 | 0.0021 | -0.0019 | 0.0019 | 0.5505 |
| rs2270894 | ALM | Fasting glucose | -0.0332 | 9975386 | 0.0024 | 0.0033 | 0.0033 | 0.324 |
| rs2274351 | ALM | Fasting glucose | 0.017 | 104264107 | 0.0019 | 0.0036 | 0.0017 | 0.1275 |
| rs2283200 | ALM | Fasting glucose | -0.0281 | 2729340 | 0.0042 | -0.0024 | 0.0037 | 0.3807 |
| rs2287821 | ALM | Fasting glucose | -0.0153 | 33935102 | 0.0019 | -0.0042 | 0.0017 | 0.0454203 |
| rs2289629 | ALM | Fasting glucose | -0.0148 | 27959903 | 0.002 | -0.0007 | 0.0017 | 0.4638 |
| rs2296316 | ALM | Fasting glucose | -0.0192 | 65520246 | 0.0019 | -0.0006 | 0.0018 | 0.4934 |
| rs2303423 | ALM | Fasting glucose | 0.0168 | 38120029 | 0.003 | 0.0013 | 0.003 | 0.755499 |
| rs2305141 | ALM | Fasting glucose | 0.0183 | 233684402 | 0.0019 | -0.0043 | 0.0019 | 0.0831591 |
| rs2324154 | ALM | Fasting glucose | 0.015 | 24027226 | 0.0019 | 0.0033 | 0.0018 | 0.0136801 |
| rs234640 | ALM | Fasting glucose | -0.0131 | 184867830 | 0.0019 | 0.0015 | 0.0018 | 0.891 |
| rs2347603 | ALM | Fasting glucose | -0.0181 | 47297426 | 0.0022 | 0.0001 | 0.0021 | 0.7545 |
| rs2347808 | ALM | Fasting glucose | -0.0125 | 2750856 | 0.0019 | 0.0023 | 0.0018 | 0.139 |
| rs2362487 | ALM | Fasting glucose | 0.0154 | 126208402 | 0.0022 | -0.001 | 0.0022 | 0.730801 |
| rs2390669 | ALM | Fasting glucose | 0.0174 | 169091942 | 0.0028 | 0.0024 | 0.0027 | 0.2352 |
| rs244711 | ALM | Fasting glucose | 0.0279 | 176509193 | 0.0022 | -0.0016 | 0.0026 | 0.6151 |
| rs2454390 | ALM | Fasting glucose | -0.0176 | 103255613 | 0.0026 | -0.0014 | 0.0026 | 0.3963 |
| rs246177 | ALM | Fasting glucose | 0.0214 | 14380768 | 0.002 | -0.0044 | 0.0019 | 0.0378599 |
| rs2490302 | ALM | Fasting glucose | 0.0221 | 37702435 | 0.0034 | 0.0004 | 0.0033 | 0.8114 |
| rs249677 | ALM | Fasting glucose | -0.0109 | 141539339 | 0.002 | 0.0009 | 0.0019 | 0.8762 |
| rs2521349 | ALM | Fasting glucose | 0.0155 | 67503501 | 0.0019 | 0.002 | 0.0019 | 0.3508 |
| rs2529090 | ALM | Fasting glucose | 0.0136 | 24662280 | 0.0025 | 0.0004 | 0.0024 | 0.6075 |
| rs2545339 | ALM | Fasting glucose | 0.0115 | 149911219 | 0.002 | 0.0052 | 0.0019 | 0.000505406 |
| rs2569888 | ALM | Fasting glucose | 0.0133 | 1625803 | 0.0022 | -0.0009 | 0.0023 | 0.6771 |
| rs2578565 | ALM | Fasting glucose | -0.0141 | 5460569 | 0.002 | 0.002 | 0.0019 | 0.3937 |
| rs258794 | ALM | Fasting glucose | 0.0147 | 142540040 | 0.0021 | -0.0033 | 0.0076 | 0.5404 |
| rs2592208 | ALM | Fasting glucose | -0.0124 | 67408873 | 0.0019 | -0.0016 | 0.0018 | 0.2843 |
| rs2607234 | ALM | Fasting glucose | -0.0302 | 35563834 | 0.0043 | 0.0049 | 0.0043 | 0.4123 |
| rs261223 | ALM | Fasting glucose | 0.0175 | 95901046 | 0.0019 | -0.0003 | 0.0019 | 0.6893 |
| rs2648725 | ALM | Fasting glucose | 0.0165 | 93015079 | 0.0023 | -0.0002 | 0.0022 | 0.8401 |
| rs2663126 | ALM | Fasting glucose | -0.0139 | 99563857 | 0.0021 | -0.0058 | 0.002 | 0.00962698 |
| rs2676298 | ALM | Fasting glucose | -0.0269 | 62726707 | 0.0027 | 0.0008 | 0.0034 | 0.769699 |
| rs2717008 | ALM | Fasting glucose | -0.0127 | 58149158 | 0.0019 | -0.0003 | 0.0019 | 0.8311 |
| rs2748501 | ALM | Fasting glucose | -0.0195 | 146312258 | 0.0019 | 0.0014 | 0.0018 | 0.366 |
| rs2754255 | ALM | Fasting glucose | -0.0153 | 88393572 | 0.0023 | 0.0064 | 0.0022 | 0.001153 |
| rs2763263 | ALM | Fasting glucose | -0.017 | 168814392 | 0.0022 | -0.0002 | 0.0021 | 0.6897 |
| rs2764264 | ALM | Fasting glucose | 0.0203 | 108934461 | 0.0021 | 0.0014 | 0.002 | 0.5109 |
| rs2788213 | ALM | Fasting glucose | 0.0123 | 703249 | 0.0021 | 0.0009 | 0.0019 | 0.755499 |
| rs2789365 | ALM | Fasting glucose | -0.0145 | 235515534 | 0.0019 | 0.0006 | 0.0018 | 0.7217 |
| rs2791654 | ALM | Fasting glucose | -0.0239 | 11129317 | 0.0022 | -0.0068 | 0.0076 | 0.3889 |
| rs2807339 | ALM | Fasting glucose | 0.0162 | 22578063 | 0.0022 | -0.0001 | 0.0021 | 1 |
| rs2812208 | ALM | Fasting glucose | 0.1156 | 50707087 | 0.0066 | -0.0017 | 0.0061 | 0.3887 |
| rs28379706 | ALM | Fasting glucose | 0.0114 | 50728062 | 0.002 | -0.0018 | 0.0024 | 0.666099 |
| rs28485212 | ALM | Fasting glucose | -0.0188 | 63550026 | 0.0027 | 0.0004 | 0.0033 | 1 |
| rs28529055 | ALM | Fasting glucose | -0.0147 | 92428216 | 0.0019 | -0.0032 | 0.0019 | 0.0984011 |
| rs28529426 | ALM | Fasting glucose | -0.0168 | 4678264 | 0.0026 | 0.0044 | 0.003 | 0.2083 |
| rs28592876 | ALM | Fasting glucose | 0.03 | 123866429 | 0.0023 | 0.0045 | 0.0024 | 0.1885 |
| rs28678024 | ALM | Fasting glucose | -0.0119 | 25937161 | 0.0021 | -0.0019 | 0.002 | 0.3616 |
| rs2871865 | ALM | Fasting glucose | -0.0493 | 99194896 | 0.003 | 0.0058 | 0.0032 | 0.2089 |
| rs2871960 | ALM | Fasting glucose | 0.0469 | 141121814 | 0.0019 | -0.0069 | 0.0017 | 0.000526599 |
| rs28736838 | ALM | Fasting glucose | -0.0117 | 120148713 | 0.002 | 0.0019 | 0.0021 | 0.3476 |
| rs2885697 | ALM | Fasting glucose | -0.0323 | 41544279 | 0.002 | 0.0013 | 0.0019 | 0.677901 |
| rs291979 | ALM | Fasting glucose | 0.0242 | 121129797 | 0.0023 | 0.0015 | 0.0022 | 0.463 |
| rs2923411 | ALM | Fasting glucose | 0.0127 | 42455206 | 0.0019 | 0.0022 | 0.0019 | 0.2428 |
| rs2925155 | ALM | Fasting glucose | -0.015 | 75886297 | 0.0022 | 0.0003 | 0.0019 | 0.8967 |
| rs293517 | ALM | Fasting glucose | -0.013 | 83662455 | 0.0021 | 0.0026 | 0.002 | 0.0680597 |
| rs2971857 | ALM | Fasting glucose | -0.0119 | 234369487 | 0.0019 | -0.002 | 0.0019 | 0.593999 |
| rs2978362 | ALM | Fasting glucose | 0.0106 | 32959397 | 0.0019 | -0.0024 | 0.0018 | 0.1707 |
| rs301807 | ALM | Fasting glucose | -0.0144 | 8484823 | 0.0019 | -0.0012 | 0.0019 | 0.4554 |
| rs3103223 | ALM | Fasting glucose | 0.0126 | 42402721 | 0.0022 | 0.0021 | 0.0021 | 0.6555 |
| rs310796 | ALM | Fasting glucose | 0.0142 | 77453226 | 0.002 | -0.0003 | 0.0018 | 0.784099 |
| rs3116194 | ALM | Fasting glucose | -0.0295 | 233061266 | 0.0032 | -0.0023 | 0.0033 | 0.3394 |
| rs3116602 | ALM | Fasting glucose | -0.0612 | 51111355 | 0.0023 | 0.001 | 0.002 | 0.3118 |
| rs31196 | ALM | Fasting glucose | -0.0107 | 158300798 | 0.0019 | -0.0049 | 0.0018 | 0.00626902 |
| rs3184504 | ALM | Fasting glucose | 0.0183 | 111884608 | 0.0019 | 0.0004 | 0.0017 | 0.9076 |
| rs3205136 | ALM | Fasting glucose | -0.0184 | 136126631 | 0.0033 | -0.0049 | 0.0027 | 0.0464997 |
| rs331917 | ALM | Fasting glucose | -0.0127 | 98158524 | 0.0019 | -0.0036 | 0.002 | 0.1111 |
| rs332116 | ALM | Fasting glucose | -0.0206 | 28926099 | 0.0021 | -0.0047 | 0.002 | 0.03002 |
| rs336630 | ALM | Fasting glucose | -0.0106 | 18607538 | 0.0019 | 0.0013 | 0.002 | 0.5439 |
| rs33973388 | ALM | Fasting glucose | 0.0249 | 46611842 | 0.0019 | 0.002 | 0.0018 | 0.7013 |
| rs34287 | ALM | Fasting glucose | 0.0187 | 67585143 | 0.002 | -0.0041 | 0.0019 | 0.0429299 |
| rs34312629 | ALM | Fasting glucose | -0.017 | 24079795 | 0.0021 | 0.003 | 0.0021 | 0.178 |
| rs34338597 | ALM | Fasting glucose | -0.0112 | 106301580 | 0.0019 | -0.0003 | 0.0019 | 1 |
| rs34345560 | ALM | Fasting glucose | 0.0219 | 69081998 | 0.0024 | 0.005 | 0.0024 | 0.0462498 |
| rs34390533 | ALM | Fasting glucose | -0.0257 | 184030838 | 0.0022 | 0.0009 | 0.0023 | 0.9464 |
| rs34517439 | ALM | Fasting glucose | 0.0421 | 78450517 | 0.0029 | -0.0108 | 0.0033 | 0.00102901 |
| rs34522021 | ALM | Fasting glucose | 0.0126 | 23350420 | 0.0019 | -0.0019 | 0.0018 | 0.1885 |
| rs34776209 | ALM | Fasting glucose | -0.0317 | 23513093 | 0.0022 | 0.0017 | 0.0022 | 0.409 |
| rs34879158 | ALM | Fasting glucose | -0.0363 | 32300634 | 0.0022 | 0.0018 | 0.0021 | 0.281 |
| rs35073631 | ALM | Fasting glucose | 0.0112 | 22696964 | 0.0019 | 0.0025 | 0.002 | 0.3121 |
| rs350832 | ALM | Fasting glucose | -0.0165 | 4069426 | 0.0023 | 0.0012 | 0.0024 | 0.5019 |
| rs35268848 | ALM | Fasting glucose | 0.0737 | 67927240 | 0.0101 | 0.0034 | 0.0069 | 0.443 |
| rs35288270 | ALM | Fasting glucose | -0.0328 | 4961278 | 0.0028 | -0.0033 | 0.0027 | 0.1628 |
| rs35732917 | ALM | Fasting glucose | 0.0204 | 73013269 | 0.0021 | 0.0061 | 0.0019 | 0.00308802 |
| rs35756741 | ALM | Fasting glucose | -0.0378 | 12868701 | 0.0033 | -0.0012 | 0.0032 | 0.686099 |
| rs35811052 | ALM | Fasting glucose | -0.0148 | 15128416 | 0.0022 | -0.0043 | 0.0023 | 0.05377 |
| rs35816944 | ALM | Fasting glucose | -0.1088 | 1828030 | 0.0117 | 0.0211 | 0.0149 | 0.3898 |
| rs35963161 | ALM | Fasting glucose | -0.0157 | 49210635 | 0.0019 | -0.0005 | 0.002 | 0.49 |
| rs36000545 | ALM | Fasting glucose | -0.022 | 79093822 | 0.002 | 0.0028 | 0.0022 | 0.1331 |
| rs36012032 | ALM | Fasting glucose | 0.0298 | 52814709 | 0.0033 | -0.0041 | 0.003 | 0.1076 |
| rs36048468 | ALM | Fasting glucose | 0.0254 | 122879901 | 0.0023 | -0.0013 | 0.0023 | 0.5989 |
| rs36226649 | ALM | Fasting glucose | 0.0485 | 24835500 | 0.0038 | 0.003 | 0.0039 | 0.533301 |
| rs373736365 | ALM | Fasting glucose | 0.0189 | 51360867 | 0.0022 | -0.0009 | 0.0078 | 0.9514 |
| rs3764002 | ALM | Fasting glucose | 0.028 | 108618630 | 0.0021 | 0.0031 | 0.0019 | 0.1944 |
| rs3768495 | ALM | Fasting glucose | -0.0178 | 109935325 | 0.0021 | 0.0023 | 0.0018 | 0.4111 |
| rs3769598 | ALM | Fasting glucose | 0.0171 | 32679732 | 0.0027 | -0.0035 | 0.0027 | 0.0624698 |
| rs377599 | ALM | Fasting glucose | 0.0217 | 2164699 | 0.0019 | -0.0032 | 0.0019 | 0.102 |
| rs3778858 | ALM | Fasting glucose | 0.0108 | 129963356 | 0.002 | -0.0005 | 0.0019 | 0.6591 |
| rs3782232 | ALM | Fasting glucose | -0.0339 | 57116249 | 0.0037 | 0.0063 | 0.0044 | 0.195 |
| rs3782811 | ALM | Fasting glucose | -0.0165 | 3339927 | 0.0022 | 0.0026 | 0.0021 | 0.3015 |
| rs3792819 | ALM | Fasting glucose | 0.021 | 172576296 | 0.0034 | -0.0012 | 0.0033 | 0.4067 |
| rs3818416 | ALM | Fasting glucose | 0.0279 | 78474468 | 0.0022 | 0.003 | 0.0022 | 0.176 |
| rs3822742 | ALM | Fasting glucose | 0.0162 | 139059017 | 0.002 | 0.0011 | 0.0019 | 0.4674 |
| rs3828729 | ALM | Fasting glucose | -0.016 | 155554707 | 0.002 | -0.0004 | 0.002 | 0.723299 |
| rs3901421 | ALM | Fasting glucose | 0.0215 | 96204538 | 0.0019 | 0.0001 | 0.0018 | 0.9563 |
| rs395980 | ALM | Fasting glucose | -0.0184 | 177430072 | 0.0021 | 0.0021 | 0.0019 | 0.2992 |
| rs40270 | ALM | Fasting glucose | 0.0151 | 55804552 | 0.0022 | 0.0112 | 0.0021 | 1.378E-07 |
| rs4073154 | ALM | Fasting glucose | 0.0274 | 129035485 | 0.0023 | -0.0019 | 0.0024 | 0.3038 |
| rs4076108 | ALM | Fasting glucose | 0.0174 | 13736088 | 0.0022 | -0.0004 | 0.0021 | 0.7831 |
| rs4077103 | ALM | Fasting glucose | -0.0143 | 49557732 | 0.0026 | 0.0008 | 0.0024 | 0.9679 |
| rs4121583 | ALM | Fasting glucose | 0.0118 | 125075 | 0.002 | 0.004 | 0.0032 | 0.2859 |
| rs41271299 | ALM | Fasting glucose | 0.0616 | 19839415 | 0.0043 | 0.0099 | 0.0066 | 0.06223 |
| rs41311445 | ALM | Fasting glucose | -0.0328 | 42070374 | 0.0032 | -0.0013 | 0.0036 | 0.6785 |
| rs42039 | ALM | Fasting glucose | 0.0481 | 92244422 | 0.0022 | -0.0028 | 0.0021 | 0.1329 |
| rs4244809 | ALM | Fasting glucose | -0.0262 | 2164333 | 0.0023 | -0.0008 | 0.0023 | 0.8638 |
| rs4252548 | ALM | Fasting glucose | -0.0753 | 55879672 | 0.0065 | 0.0113 | 0.0093 | 0.1427 |
| rs4274112 | ALM | Fasting glucose | -0.0217 | 26746199 | 0.002 | 0.005 | 0.0019 | 0.0239398 |
| rs4282339 | ALM | Fasting glucose | -0.0311 | 168256240 | 0.0023 | -0.0047 | 0.002 | 0.0322797 |
| rs4287835 | ALM | Fasting glucose | 0.0147 | 31457337 | 0.0019 | 0.0041 | 0.0018 | 0.0326099 |
| rs4360494 | ALM | Fasting glucose | -0.0198 | 38455891 | 0.0019 | -0.0022 | 0.002 | 0.4018 |
| rs4380799 | ALM | Fasting glucose | -0.0255 | 32571864 | 0.0021 | -0.0032 | 0.0038 | 0.756 |
| rs4383083 | ALM | Fasting glucose | 0.0111 | 63080442 | 0.002 | -0.001 | 0.002 | 0.5439 |
| rs447352 | ALM | Fasting glucose | -0.0181 | 678750 | 0.0029 | -0.002 | 0.0037 | 0.5772 |
| rs4504126 | ALM | Fasting glucose | 0.046 | 33600582 | 0.0058 | -0.0032 | 0.0061 | 0.4519 |
| rs45474992 | ALM | Fasting glucose | -0.0617 | 47724564 | 0.0051 | -0.0068 | 0.0069 | 0.611699 |
| rs45528934 | ALM | Fasting glucose | 0.0262 | 23793305 | 0.0026 | -0.0018 | 0.0029 | 0.549301 |
| rs4602848 | ALM | Fasting glucose | 0.016 | 92186933 | 0.002 | -0.0004 | 0.0019 | 0.9586 |
| rs4622329 | ALM | Fasting glucose | 0.0149 | 102321935 | 0.002 | -0.0035 | 0.0019 | 0.0616595 |
| rs4640244 | ALM | Fasting glucose | -0.02 | 21284223 | 0.0019 | 0.005 | 0.0018 | 0.00565705 |
| rs4644481 | ALM | Fasting glucose | -0.0112 | 155130900 | 0.0019 | -0.0021 | 0.0019 | 0.755499 |
| rs4655345 | ALM | Fasting glucose | -0.0246 | 214608704 | 0.0019 | 0.0035 | 0.0019 | 0.1577 |
| rs4682483 | ALM | Fasting glucose | -0.0165 | 112993982 | 0.0026 | 0.006 | 0.0024 | 0.0960506 |
| rs4683435 | ALM | Fasting glucose | 0.0144 | 142624732 | 0.0022 | -0.0027 | 0.0022 | 0.324 |
| rs4735761 | ALM | Fasting glucose | 0.0331 | 78097161 | 0.0021 | 0.0037 | 0.002 | 0.0576196 |
| rs4748008 | ALM | Fasting glucose | -0.0125 | 12935125 | 0.0019 | -0.0012 | 0.0018 | 0.366 |
| rs4752689 | ALM | Fasting glucose | 0.0205 | 124131176 | 0.0019 | 0.0061 | 0.0019 | 0.000260399 |
| rs4752829 | ALM | Fasting glucose | 0.0262 | 47396654 | 0.0021 | 0.0129 | 0.0018 | 7.43361E-12 |
| rs4788218 | ALM | Fasting glucose | 0.0275 | 30055750 | 0.0019 | -0.0029 | 0.0019 | 0.2325 |
| rs4807472 | ALM | Fasting glucose | -0.0158 | 3448842 | 0.002 | -0.0019 | 0.002 | 0.5439 |
| rs4815952 | ALM | Fasting glucose | -0.0161 | 6934897 | 0.0019 | -0.0006 | 0.0018 | 0.7694 |
| rs4818280 | ALM | Fasting glucose | -0.0124 | 18114472 | 0.002 | -0.0001 | 0.0019 | 0.755499 |
| rs4847378 | ALM | Fasting glucose | 0.0136 | 93324634 | 0.0019 | -0.0061 | 0.0019 | 0.00875709 |
| rs4852257 | ALM | Fasting glucose | -0.0231 | 71678520 | 0.0019 | -0.0061 | 0.0019 | 0.000862303 |
| rs4865956 | ALM | Fasting glucose | -0.0258 | 54882505 | 0.0021 | -0.0014 | 0.0019 | 0.3775 |
| rs4870941 | ALM | Fasting glucose | -0.0297 | 126498828 | 0.0023 | -0.0007 | 0.002 | 0.9798 |
| rs488621 | ALM | Fasting glucose | 0.0191 | 169707552 | 0.0019 | 0.0117 | 0.0017 | 3.30202E-10 |
| rs4900578 | ALM | Fasting glucose | -0.0177 | 103926010 | 0.002 | -0.0089 | 0.007 | 0.2064 |
| rs4932439 | ALM | Fasting glucose | -0.0151 | 89401109 | 0.0025 | -0.0023 | 0.0022 | 0.2731 |
| rs4938359 | ALM | Fasting glucose | -0.0156 | 117093560 | 0.0024 | 0.0006 | 0.0022 | 0.8008 |
| rs4940874 | ALM | Fasting glucose | 0.0148 | 57105638 | 0.0024 | 0.0023 | 0.0023 | 0.4562 |
| rs4965298 | ALM | Fasting glucose | -0.0119 | 100802766 | 0.0021 | -0.0036 | 0.002 | 0.1179 |
| rs496783 | ALM | Fasting glucose | -0.0124 | 116137961 | 0.0019 | -0.0012 | 0.0019 | 0.5052 |
| rs4976262 | ALM | Fasting glucose | -0.0245 | 134379531 | 0.002 | -0.0012 | 0.002 | 0.4947 |
| rs4985445 | ALM | Fasting glucose | -0.0175 | 69867835 | 0.0019 | 0.0031 | 0.0018 | 0.1762 |
| rs532499 | ALM | Fasting glucose | -0.0127 | 30165465 | 0.0022 | -0.0048 | 0.0022 | 0.1096 |
| rs543650 | ALM | Fasting glucose | 0.025 | 152110943 | 0.002 | 0.0007 | 0.0018 | 0.7141 |
| rs544136 | ALM | Fasting glucose | 0.0121 | 101041229 | 0.0022 | -0.0006 | 0.0021 | 0.5891 |
| rs545104 | ALM | Fasting glucose | 0.0127 | 118591352 | 0.002 | 0.0032 | 0.0019 | 0.0658795 |
| rs55717234 | ALM | Fasting glucose | 0.0122 | 150999863 | 0.0019 | 0.0032 | 0.0024 | 0.2317 |
| rs55758152 | ALM | Fasting glucose | 0.0145 | 171317318 | 0.002 | -0.0018 | 0.0024 | 0.3558 |
| rs55852614 | ALM | Fasting glucose | -0.0393 | 172416869 | 0.0022 | -0.0026 | 0.0021 | 0.2906 |
| rs55872725 | ALM | Fasting glucose | 0.0222 | 53809123 | 0.0019 | -0.0019 | 0.0017 | 0.0520595 |
| rs55980611 | ALM | Fasting glucose | 0.0164 | 74771027 | 0.0028 | 0.0157 | 0.0102 | 0.0820805 |
| rs56112295 | ALM | Fasting glucose | 0.0154 | 105877057 | 0.0024 | -0.0008 | 0.0039 | 0.697 |
| rs56207600 | ALM | Fasting glucose | 0.0192 | 126196537 | 0.003 | 0.0027 | 0.0029 | 0.3506 |
| rs56239180 | ALM | Fasting glucose | -0.0459 | 32937951 | 0.0062 | 0.004 | 0.0073 | 0.2755 |
| rs56363908 | ALM | Fasting glucose | -0.0382 | 96611052 | 0.0047 | -0.0023 | 0.0066 | 0.9754 |
| rs568267 | ALM | Fasting glucose | 0.0122 | 8799828 | 0.0022 | -0.0011 | 0.0021 | 0.573 |
| rs57059662 | ALM | Fasting glucose | 0.0118 | 33217275 | 0.002 | 0.0004 | 0.0019 | 0.8311 |
| rs5742915 | ALM | Fasting glucose | 0.0248 | 74336633 | 0.0019 | 0.0014 | 0.0017 | 0.2459 |
| rs57513571 | ALM | Fasting glucose | -0.0191 | 2309130 | 0.0024 | 0.0108 | 0.0024 | 2.52901E-05 |
| rs5753518 | ALM | Fasting glucose | 0.0242 | 31631314 | 0.0033 | 0.0007 | 0.0031 | 0.8209 |
| rs5763821 | ALM | Fasting glucose | 0.0191 | 30549071 | 0.002 | 0.0026 | 0.0068 | 0.5329 |
| rs57696574 | ALM | Fasting glucose | 0.0173 | 54884826 | 0.002 | 0.0009 | 0.0019 | 1 |
| rs577289 | ALM | Fasting glucose | -0.0125 | 40208911 | 0.0021 | 0.0019 | 0.0021 | 0.547599 |
| rs57791149 | ALM | Fasting glucose | -0.0173 | 54222307 | 0.0019 | 0.0017 | 0.0019 | 0.6697 |
| rs591668 | ALM | Fasting glucose | -0.0174 | 27535931 | 0.0019 | -0.0014 | 0.0018 | 0.7491 |
| rs599004 | ALM | Fasting glucose | -0.0157 | 140439740 | 0.0021 | 0.0017 | 0.002 | 0.2086 |
| rs59950280 | ALM | Fasting glucose | -0.0254 | 3452345 | 0.002 | -0.0017 | 0.0022 | 0.663001 |
| rs59985551 | ALM | Fasting glucose | -0.0313 | 56106928 | 0.0022 | -0.0035 | 0.0021 | 0.0571597 |
| rs6000886 | ALM | Fasting glucose | 0.0131 | 38176670 | 0.002 | -0.0001 | 0.002 | 0.8994 |
| rs6028716 | ALM | Fasting glucose | -0.021 | 38547459 | 0.0022 | -0.0003 | 0.0021 | 0.9808 |
| rs60389750 | ALM | Fasting glucose | -0.0175 | 77182836 | 0.0021 | -0.002 | 0.0022 | 0.3129 |
| rs60408354 | ALM | Fasting glucose | 0.0259 | 70158495 | 0.0036 | -0.0001 | 0.0037 | 0.8183 |
| rs604723 | ALM | Fasting glucose | -0.0166 | 100610546 | 0.0021 | 0.0013 | 0.0018 | 0.4111 |
| rs6054390 | ALM | Fasting glucose | -0.0188 | 6592094 | 0.002 | -0.0016 | 0.0019 | 0.4554 |
| rs6054491 | ALM | Fasting glucose | -0.0142 | 6709535 | 0.0022 | 0.002 | 0.0021 | 0.2906 |
| rs6066122 | ALM | Fasting glucose | 0.0127 | 45558573 | 0.0023 | 0.0019 | 0.0021 | 0.2311 |
| rs60804050 | ALM | Fasting glucose | -0.0217 | 118870373 | 0.0021 | 0.0023 | 0.002 | 0.2482 |
| rs6082354 | ALM | Fasting glucose | -0.024 | 21217976 | 0.002 | -0.0011 | 0.002 | 0.685799 |
| rs610694 | ALM | Fasting glucose | 0.0136 | 121304826 | 0.0019 | -0.0028 | 0.0016 | 0.0729407 |
| rs61397287 | ALM | Fasting glucose | 0.0235 | 144223279 | 0.0036 | -0.006 | 0.0128 | 0.752501 |
| rs6142059 | ALM | Fasting glucose | 0.0116 | 32544327 | 0.0019 | 0.0018 | 0.0018 | 0.4111 |
| rs61528919 | ALM | Fasting glucose | 0.014 | 1004909 | 0.002 | 0.0062 | 0.0073 | 0.5928 |
| rs61729527 | ALM | Fasting glucose | -0.0346 | 77761919 | 0.0043 | 0.0035 | 0.0048 | 0.2325 |
| rs61732778 | ALM | Fasting glucose | 0.023 | 187443314 | 0.0037 | -0.0028 | 0.0037 | 0.4204 |
| rs61827272 | ALM | Fasting glucose | 0.0144 | 203810763 | 0.0021 | 0.002 | 0.0021 | 0.1124 |
| rs61878760 | ALM | Fasting glucose | 0.019 | 12807189 | 0.0034 | -0.0034 | 0.0036 | 0.459501 |
| rs61919240 | ALM | Fasting glucose | 0.0137 | 8831954 | 0.002 | -0.0011 | 0.002 | 0.6673 |
| rs61944841 | ALM | Fasting glucose | 0.0253 | 27049616 | 0.002 | 0.0023 | 0.0022 | 0.3351 |
| rs62033029 | ALM | Fasting glucose | -0.0141 | 50107273 | 0.0023 | -0.0034 | 0.0022 | 0.2921 |
| rs62070319 | ALM | Fasting glucose | -0.018 | 89573216 | 0.002 | 0.0012 | 0.0069 | 0.985 |
| rs62103240 | ALM | Fasting glucose | 0.0212 | 77650637 | 0.0037 | -0.0042 | 0.0048 | 0.2603 |
| rs62106258 | ALM | Fasting glucose | -0.0504 | 417167 | 0.0044 | 0.01 | 0.0067 | 0.0830195 |
| rs62143873 | ALM | Fasting glucose | -0.0115 | 72035050 | 0.0019 | -0.0012 | 0.0019 | 0.6125 |
| rs62370472 | ALM | Fasting glucose | -0.0253 | 52767109 | 0.0023 | -0.0016 | 0.0022 | 0.590599 |
| rs62466110 | ALM | Fasting glucose | -0.0371 | 92623541 | 0.0041 | -0.0012 | 0.0052 | 0.729899 |
| rs62501195 | ALM | Fasting glucose | -0.0198 | 24041988 | 0.0025 | -0.0002 | 0.0026 | 0.536 |
| rs62515437 | ALM | Fasting glucose | 0.0369 | 57160328 | 0.0023 | -0.0006 | 0.0023 | 0.6142 |
| rs62621812 | ALM | Fasting glucose | 0.0743 | 127015083 | 0.0069 | 0.0015 | 0.0068 | 0.9409 |
| rs6425817 | ALM | Fasting glucose | 0.0157 | 33873034 | 0.002 | 0.0036 | 0.0019 | 0.0378599 |
| rs6470771 | ALM | Fasting glucose | -0.0268 | 130743726 | 0.0025 | 0.0012 | 0.0025 | 0.6148 |
| rs6502935 | ALM | Fasting glucose | -0.0125 | 1650168 | 0.0022 | -0.0005 | 0.0023 | 0.9825 |
| rs650508 | ALM | Fasting glucose | -0.013 | 45880122 | 0.002 | 0.0018 | 0.002 | 0.4158 |
| rs6543146 | ALM | Fasting glucose | 0.0154 | 103096695 | 0.0019 | 0.0037 | 0.0019 | 0.1355 |
| rs655113 | ALM | Fasting glucose | 0.0188 | 52269151 | 0.0021 | -0.0022 | 0.002 | 0.4947 |
| rs6570509 | ALM | Fasting glucose | -0.0244 | 142716286 | 0.0021 | -0.0041 | 0.0018 | 0.04559 |
| rs6582398 | ALM | Fasting glucose | 0.014 | 42870444 | 0.002 | -0.0005 | 0.0019 | 0.5164 |
| rs6593210 | ALM | Fasting glucose | 0.0146 | 55254186 | 0.0024 | 0.0005 | 0.0021 | 0.8474 |
| rs664317 | ALM | Fasting glucose | -0.0177 | 89812230 | 0.0026 | 0.0024 | 0.0025 | 0.2762 |
| rs6675858 | ALM | Fasting glucose | -0.0137 | 224559936 | 0.0023 | 0.0049 | 0.0022 | 0.0113099 |
| rs6693481 | ALM | Fasting glucose | -0.0143 | 203766395 | 0.002 | -0.0015 | 0.002 | 0.1292 |
| rs670318 | ALM | Fasting glucose | 0.0413 | 63727542 | 0.0044 | 0.0032 | 0.0047 | 0.6029 |
| rs6721191 | ALM | Fasting glucose | -0.0144 | 10190115 | 0.0019 | 0.0015 | 0.0019 | 0.15 |
| rs6738207 | ALM | Fasting glucose | 0.0127 | 105989716 | 0.0019 | -0.0008 | 0.0019 | 0.5223 |
| rs6739278 | ALM | Fasting glucose | -0.021 | 44401055 | 0.0024 | 0.0036 | 0.0022 | 0.1911 |
| rs67527161 | ALM | Fasting glucose | -0.0182 | 63781824 | 0.0023 | -0.0004 | 0.0022 | 0.621901 |
| rs67551338 | ALM | Fasting glucose | 0.0576 | 3393100 | 0.004 | 0.0042 | 0.004 | 0.7391 |
| rs6762851 | ALM | Fasting glucose | -0.0218 | 56686329 | 0.002 | -0.0014 | 0.0019 | 0.5052 |
| rs67716382 | ALM | Fasting glucose | 0.0226 | 46890317 | 0.0023 | -0.0001 | 0.0022 | 0.6866 |
| rs6789000 | ALM | Fasting glucose | 0.0121 | 25188002 | 0.002 | 0.0044 | 0.0072 | 0.516 |
| rs68049170 | ALM | Fasting glucose | -0.0259 | 72432047 | 0.0021 | -0.0046 | 0.002 | 0.0680597 |
| rs680882 | ALM | Fasting glucose | 0.0133 | 18325278 | 0.0022 | -0.0029 | 0.0021 | 0.3126 |
| rs6821305 | ALM | Fasting glucose | 0.0204 | 122713863 | 0.0019 | 0.0026 | 0.0018 | 0.1795 |
| rs684905 | ALM | Fasting glucose | -0.0118 | 10472790 | 0.0019 | 0.0009 | 0.0018 | 0.9563 |
| rs6849302 | ALM | Fasting glucose | 0.0155 | 156665074 | 0.0024 | -0.0011 | 0.0023 | 0.7757 |
| rs6860245 | ALM | Fasting glucose | 0.0589 | 127367998 | 0.0022 | 0.0003 | 0.0022 | 1 |
| rs6874142 | ALM | Fasting glucose | 0.0288 | 172753555 | 0.0031 | -0.0012 | 0.0047 | 0.580399 |
| rs6902109 | ALM | Fasting glucose | -0.0167 | 130316559 | 0.0019 | -0.0013 | 0.0018 | 0.2978 |
| rs6931421 | ALM | Fasting glucose | -0.0279 | 80880138 | 0.002 | 0.0003 | 0.002 | 0.9597 |
| rs6977416 | ALM | Fasting glucose | 0.0457 | 150542711 | 0.002 | 0.0033 | 0.0018 | 0.0426305 |
| rs700677 | ALM | Fasting glucose | 0.0173 | 198702424 | 0.002 | -0.0011 | 0.0019 | 0.4998 |
| rs7007389 | ALM | Fasting glucose | -0.0134 | 25355022 | 0.002 | -0.0026 | 0.0071 | 0.5516 |
| rs7014590 | ALM | Fasting glucose | -0.0228 | 89335647 | 0.0022 | 0.0001 | 0.0021 | 0.7728 |
| rs7020491 | ALM | Fasting glucose | -0.0178 | 128144477 | 0.0019 | 0.0014 | 0.0019 | 0.3508 |
| rs702886 | ALM | Fasting glucose | 0.012 | 65753310 | 0.002 | -0.0014 | 0.0019 | 0.4554 |
| rs704660 | ALM | Fasting glucose | 0.0153 | 30447998 | 0.0019 | 0.0006 | 0.0018 | 0.7013 |
| rs7082659 | ALM | Fasting glucose | 0.0156 | 12017584 | 0.0028 | -0.0011 | 0.0029 | 0.6349 |
| rs7095472 | ALM | Fasting glucose | 0.0267 | 70399109 | 0.0019 | -0.0015 | 0.0019 | 0.3775 |
| rs7107356 | ALM | Fasting glucose | 0.0133 | 47676170 | 0.0019 | -0.0061 | 0.0017 | 0.000147299 |
| rs7129320 | ALM | Fasting glucose | -0.0389 | 68388220 | 0.0025 | -0.0019 | 0.0026 | 0.324 |
| rs713467 | ALM | Fasting glucose | 0.0146 | 84646473 | 0.0019 | -0.0008 | 0.0016 | 0.675601 |
| rs7137546 | ALM | Fasting glucose | 0.0142 | 577237 | 0.0019 | 0.0046 | 0.0019 | 0.0131801 |
| rs71414738 | ALM | Fasting glucose | 0.015 | 127876242 | 0.0025 | -0.0004 | 0.0025 | 0.9843 |
| rs7144307 | ALM | Fasting glucose | -0.0122 | 69533837 | 0.002 | 0.0002 | 0.0019 | 0.7358 |
| rs71635721 | ALM | Fasting glucose | 0.0316 | 171960170 | 0.0039 | 0.0174 | 0.0144 | 0.2507 |
| rs7185244 | ALM | Fasting glucose | -0.0148 | 86546887 | 0.0023 | 0.0011 | 0.0023 | 0.3911 |
| rs718603 | ALM | Fasting glucose | 0.0131 | 2644245 | 0.0021 | -0.0012 | 0.002 | 0.5376 |
| rs7220127 | ALM | Fasting glucose | -0.0105 | 64545922 | 0.0019 | 0.0005 | 0.0018 | 0.763099 |
| rs7228151 | ALM | Fasting glucose | -0.0185 | 57181694 | 0.0023 | 0.0038 | 0.0022 | 0.1451 |
| rs7229520 | ALM | Fasting glucose | -0.0224 | 46516468 | 0.002 | 0.0007 | 0.0019 | 0.6037 |
| rs723149 | ALM | Fasting glucose | -0.0276 | 46577056 | 0.0019 | -0.003 | 0.0019 | 0.2517 |
| rs72656010 | ALM | Fasting glucose | -0.0668 | 57122215 | 0.0028 | 0.0015 | 0.0027 | 0.4992 |
| rs72657800 | ALM | Fasting glucose | -0.0219 | 90822051 | 0.0035 | 0.0005 | 0.0035 | 0.779701 |
| rs72695791 | ALM | Fasting glucose | -0.0297 | 184059452 | 0.0051 | 0.0035 | 0.0052 | 0.6643 |
| rs72721979 | ALM | Fasting glucose | -0.0229 | 135827942 | 0.0027 | -0.0015 | 0.0029 | 0.9143 |
| rs72726050 | ALM | Fasting glucose | -0.0192 | 42270059 | 0.0034 | 0.0027 | 0.0041 | 0.2153 |
| rs72771070 | ALM | Fasting glucose | 0.015 | 19993750 | 0.0021 | -0.0005 | 0.0019 | 0.9363 |
| rs72801843 | ALM | Fasting glucose | 0.0313 | 53508802 | 0.0021 | -0.0068 | 0.002 | 8.83995E-05 |
| rs72809820 | ALM | Fasting glucose | -0.0111 | 97360079 | 0.002 | 0.0013 | 0.0019 | 0.4211 |
| rs72829852 | ALM | Fasting glucose | 0.0309 | 46633974 | 0.0039 | 0.0041 | 0.0039 | 0.2081 |
| rs72841270 | ALM | Fasting glucose | 0.0294 | 104642237 | 0.0028 | 0.0019 | 0.0024 | 0.3138 |
| rs7286917 | ALM | Fasting glucose | 0.0171 | 39860868 | 0.0023 | -0.0012 | 0.0021 | 0.5664 |
| rs72894003 | ALM | Fasting glucose | -0.0423 | 34775096 | 0.0038 | -0.0015 | 0.0048 | 0.7792 |
| rs73006226 | ALM | Fasting glucose | -0.0182 | 108072728 | 0.0029 | 0.0029 | 0.0029 | 0.1322 |
| rs7301341 | ALM | Fasting glucose | -0.0255 | 94083105 | 0.002 | 0.0019 | 0.0019 | 0.4516 |
| rs73052033 | ALM | Fasting glucose | -0.0151 | 185828465 | 0.0024 | 0.0001 | 0.0022 | 0.8577 |
| rs73125634 | ALM | Fasting glucose | -0.0195 | 20069826 | 0.0021 | -0.0073 | 0.002 | 0.000609705 |
| rs73197345 | ALM | Fasting glucose | 0.0211 | 36770120 | 0.0028 | 0.0008 | 0.0025 | 0.7523 |
| rs7320878 | ALM | Fasting glucose | -0.015 | 91994132 | 0.0019 | 0.002 | 0.0018 | 0.1543 |
| rs7321635 | ALM | Fasting glucose | -0.0132 | 21472055 | 0.002 | 0.0001 | 0.0019 | 0.9793 |
| rs7328187 | ALM | Fasting glucose | 0.0116 | 74189974 | 0.0019 | -0.0071 | 0.0018 | 0.000222998 |
| rs73384223 | ALM | Fasting glucose | -0.0205 | 3869315 | 0.0024 | 0.011 | 0.0023 | 1.10601E-05 |
| rs73413540 | ALM | Fasting glucose | -0.0124 | 3090976 | 0.0023 | -0.0002 | 0.0024 | 0.9164 |
| rs7367519 | ALM | Fasting glucose | 0.0164 | 204479176 | 0.002 | -0.0042 | 0.002 | 0.0725705 |
| rs73696333 | ALM | Fasting glucose | 0.0191 | 46669400 | 0.0024 | 0.0005 | 0.0024 | 0.752899 |
| rs73856768 | ALM | Fasting glucose | -0.0247 | 157788804 | 0.0035 | 0.0003 | 0.0033 | 0.8775 |
| rs7418410 | ALM | Fasting glucose | 0.0155 | 10236402 | 0.0019 | -0.0015 | 0.0018 | 0.106 |
| rs74379684 | ALM | Fasting glucose | -0.0272 | 94050205 | 0.0036 | -0.0026 | 0.0034 | 0.491801 |
| rs74458759 | ALM | Fasting glucose | 0.0171 | 136940614 | 0.0022 | 0.0031 | 0.0039 | 0.3177 |
| rs7448554 | ALM | Fasting glucose | -0.0132 | 95711603 | 0.002 | 0.0179 | 0.0022 | 5.01649E-14 |
| rs74494415 | ALM | Fasting glucose | -0.0417 | 74972138 | 0.0049 | -0.0099 | 0.0044 | 0.0215998 |
| rs7485647 | ALM | Fasting glucose | -0.0261 | 131631133 | 0.0026 | 0.0011 | 0.0024 | 0.8372 |
| rs75022676 | ALM | Fasting glucose | -0.0163 | 60293216 | 0.0023 | -0.0008 | 0.0022 | 0.5989 |
| rs7522400 | ALM | Fasting glucose | 0.0129 | 36613380 | 0.0022 | -0.0006 | 0.0021 | 0.9625 |
| rs7543136 | ALM | Fasting glucose | -0.021 | 22472451 | 0.0021 | 0.0004 | 0.002 | 0.9803 |
| rs7543202 | ALM | Fasting glucose | 0.0129 | 73872885 | 0.0019 | -0.0016 | 0.0019 | 0.4674 |
| rs75508358 | ALM | Fasting glucose | 0.0266 | 96926382 | 0.0045 | -0.0024 | 0.0049 | 0.8452 |
| rs7563362 | ALM | Fasting glucose | 0.0352 | 620297 | 0.0027 | -0.0023 | 0.0023 | 0.3679 |
| rs7570235 | ALM | Fasting glucose | -0.0168 | 242491353 | 0.0019 | 0.0018 | 0.0021 | 0.4414 |
| rs75702986 | ALM | Fasting glucose | -0.0163 | 35566151 | 0.0025 | 0.0025 | 0.0025 | 0.3142 |
| rs757834 | ALM | Fasting glucose | 0.0256 | 139717200 | 0.0024 | 0.005 | 0.0023 | 0.00836007 |
| rs7598430 | ALM | Fasting glucose | -0.016 | 219193963 | 0.0019 | 0.0014 | 0.0019 | 0.7694 |
| rs7610055 | ALM | Fasting glucose | -0.0373 | 12388409 | 0.0029 | -0.0073 | 0.0024 | 0.001448 |
| rs7633464 | ALM | Fasting glucose | 0.0175 | 98715823 | 0.0019 | 0.0041 | 0.0017 | 0.00340197 |
| rs76364830 | ALM | Fasting glucose | -0.0471 | 13372120 | 0.0039 | 0.0025 | 0.0048 | 0.8653 |
| rs76517946 | ALM | Fasting glucose | -0.0368 | 68354936 | 0.0035 | -0.0018 | 0.0037 | 0.4046 |
| rs7679276 | ALM | Fasting glucose | -0.033 | 146860186 | 0.0048 | 0.0002 | 0.0056 | 0.5369 |
| rs7689420 | ALM | Fasting glucose | 0.0466 | 145568352 | 0.0025 | 0.0028 | 0.0022 | 0.3465 |
| rs76895963 | ALM | Fasting glucose | 0.1639 | 4384844 | 0.0073 | -0.0332 | 0.0113 | 0.001789 |
| rs7701233 | ALM | Fasting glucose | -0.0179 | 171218388 | 0.0019 | 0.0024 | 0.0018 | 0.2388 |
| rs77013652 | ALM | Fasting glucose | 0.049 | 51142279 | 0.0081 | 0.0054 | 0.0071 | 0.2808 |
| rs772222 | ALM | Fasting glucose | 0.0121 | 52356892 | 0.0021 | 0.0004 | 0.0021 | 1 |
| rs7731023 | ALM | Fasting glucose | 0.0166 | 36181627 | 0.0019 | -0.0027 | 0.0017 | 0.1242 |
| rs7735891 | ALM | Fasting glucose | 0.0259 | 131597005 | 0.0019 | 0.0046 | 0.0019 | 0.0880096 |
| rs77364196 | ALM | Fasting glucose | -0.033 | 88353016 | 0.0043 | 0.0049 | 0.005 | 0.1222 |
| rs77447813 | ALM | Fasting glucose | 0.0224 | 50827041 | 0.0034 | -0.0023 | 0.0033 | 0.4053 |
| rs7768382 | ALM | Fasting glucose | -0.0201 | 166341870 | 0.0019 | -0.0018 | 0.0018 | 0.5286 |
| rs7768973 | ALM | Fasting glucose | -0.024 | 109745325 | 0.0019 | -0.0045 | 0.0018 | 0.00925103 |
| rs77809369 | ALM | Fasting glucose | 0.0237 | 9052448 | 0.0039 | -0.0057 | 0.004 | 0.1066 |
| rs78000963 | ALM | Fasting glucose | 0.0169 | 30502802 | 0.0031 | -0.0017 | 0.0039 | 0.5592 |
| rs78051210 | ALM | Fasting glucose | 0.0263 | 131379491 | 0.0036 | 0.0012 | 0.0037 | 0.4449 |
| rs7816345 | ALM | Fasting glucose | 0.0255 | 36846109 | 0.0025 | 0.0059 | 0.0022 | 0.00668298 |
| rs781669 | ALM | Fasting glucose | 0.0164 | 57819794 | 0.0019 | -0.0022 | 0.0018 | 0.1979 |
| rs7826059 | ALM | Fasting glucose | 0.0114 | 22512068 | 0.002 | 0.0004 | 0.0019 | 0.697 |
| rs7828086 | ALM | Fasting glucose | 0.0135 | 120843775 | 0.0022 | 0.0006 | 0.0021 | 0.9813 |
| rs78378222 | ALM | Fasting glucose | 0.138 | 7571752 | 0.0087 | -0.0246 | 0.0081 | 0.00482103 |
| rs78457529 | ALM | Fasting glucose | -0.0904 | 24950880 | 0.0088 | -0.0041 | 0.0102 | 0.574 |
| rs78525785 | ALM | Fasting glucose | -0.0169 | 111038331 | 0.002 | -0.0005 | 0.0069 | 0.8908 |
| rs7858712 | ALM | Fasting glucose | 0.0347 | 16738312 | 0.0034 | -0.0042 | 0.0033 | 0.1938 |
| rs7863102 | ALM | Fasting glucose | -0.011 | 73963468 | 0.0019 | -0.0038 | 0.0019 | 0.1287 |
| rs78766798 | ALM | Fasting glucose | 0.0319 | 7517075 | 0.0035 | 0.0002 | 0.0042 | 0.9508 |
| rs7893378 | ALM | Fasting glucose | 0.0175 | 93634095 | 0.0031 | 0.0046 | 0.0027 | 0.144 |
| rs7902 | ALM | Fasting glucose | 0.0149 | 95565288 | 0.0019 | -0.0011 | 0.0019 | 0.3937 |
| rs79066296 | ALM | Fasting glucose | -0.0169 | 76391462 | 0.0022 | -0.001 | 0.0079 | 0.9223 |
| rs7941305 | ALM | Fasting glucose | -0.0129 | 28652116 | 0.0021 | 0.003 | 0.0075 | 0.9173 |
| rs79441499 | ALM | Fasting glucose | -0.0138 | 7201704 | 0.0019 | 0.0006 | 0.002 | 0.9798 |
| rs7952436 | ALM | Fasting glucose | -0.0453 | 67024534 | 0.0034 | 0.0133 | 0.0048 | 0.00404203 |
| rs7971536 | ALM | Fasting glucose | -0.0194 | 102373788 | 0.0019 | 0.0019 | 0.0018 | 0.3104 |
| rs798548 | ALM | Fasting glucose | -0.0359 | 2760935 | 0.0021 | -0.0023 | 0.002 | 0.3761 |
| rs8000973 | ALM | Fasting glucose | 0.0134 | 100691367 | 0.0019 | -0.0014 | 0.0018 | 0.4111 |
| rs80132799 | ALM | Fasting glucose | 0.0231 | 62322896 | 0.0038 | -0.0016 | 0.0041 | 0.9019 |
| rs8017006 | ALM | Fasting glucose | 0.0122 | 42745052 | 0.002 | 0.0022 | 0.0021 | 0.4811 |
| rs8018486 | ALM | Fasting glucose | -0.0138 | 39818616 | 0.0024 | 0.0004 | 0.0023 | 1 |
| rs8019890 | ALM | Fasting glucose | 0.025 | 21538067 | 0.0019 | -0.0017 | 0.0021 | 0.3359 |
| rs8020095 | ALM | Fasting glucose | -0.0145 | 67453858 | 0.0027 | 0.0041 | 0.0025 | 0.1192 |
| rs80280630 | ALM | Fasting glucose | -0.0168 | 117030861 | 0.003 | -0.0023 | 0.0032 | 0.4768 |
| rs80295797 | ALM | Fasting glucose | -0.0198 | 23341690 | 0.002 | 0.0008 | 0.002 | 0.6134 |
| rs8042578 | ALM | Fasting glucose | 0.0287 | 66992964 | 0.0022 | -0.0014 | 0.002 | 0.5215 |
| rs8054549 | ALM | Fasting glucose | -0.0251 | 86417234 | 0.0019 | 0.0025 | 0.0019 | 0.2744 |
| rs8112948 | ALM | Fasting glucose | -0.0297 | 2175005 | 0.0022 | 0.0102 | 0.0078 | 0.1753 |
| rs8136517 | ALM | Fasting glucose | 0.0267 | 46439433 | 0.0039 | -0.0026 | 0.0043 | 0.5719 |
| rs822530 | ALM | Fasting glucose | 0.0255 | 148631555 | 0.0024 | -0.0029 | 0.0023 | 0.3911 |
| rs839255 | ALM | Fasting glucose | -0.0126 | 57974580 | 0.0021 | 0.0014 | 0.002 | 0.5954 |
| rs861674 | ALM | Fasting glucose | 0.0128 | 112064475 | 0.0019 | -0.0001 | 0.0018 | 0.6811 |
| rs867529 | ALM | Fasting glucose | 0.0184 | 88913273 | 0.0021 | 0.0011 | 0.002 | 0.578 |
| rs876122 | ALM | Fasting glucose | 0.0162 | 6886297 | 0.0029 | -0.0009 | 0.0027 | 0.9563 |
| rs8904 | ALM | Fasting glucose | -0.0157 | 35871217 | 0.002 | -0.0009 | 0.002 | 0.723299 |
| rs900399 | ALM | Fasting glucose | 0.0164 | 156798732 | 0.0019 | 0.0026 | 0.0017 | 0.1242 |
| rs905938 | ALM | Fasting glucose | 0.0394 | 154991389 | 0.0021 | -0.0002 | 0.002 | 0.9597 |
| rs909220 | ALM | Fasting glucose | -0.015 | 75908780 | 0.0019 | -0.0008 | 0.0018 | 0.8052 |
| rs9266244 | ALM | Fasting glucose | -0.0427 | 31325692 | 0.0021 | -0.0011 | 0.0025 | 0.6579 |
| rs9343327 | ALM | Fasting glucose | 0.014 | 76606296 | 0.0019 | -0.0015 | 0.0018 | 0.4763 |
| rs9344126 | ALM | Fasting glucose | -0.0185 | 81907559 | 0.0019 | -0.0015 | 0.0017 | 0.2955 |
| rs9375188 | ALM | Fasting glucose | 0.0136 | 98555272 | 0.0019 | -0.0062 | 0.0018 | 0.000368697 |
| rs9385002 | ALM | Fasting glucose | -0.0147 | 117552469 | 0.0022 | 0.0031 | 0.0021 | 0.152 |
| rs9388490 | ALM | Fasting glucose | 0.0462 | 126704795 | 0.0019 | 0.0056 | 0.0018 | 0.00405005 |
| rs9391254 | ALM | Fasting glucose | 0.0166 | 105377347 | 0.002 | 0.0065 | 0.0019 | 0.00144099 |
| rs947099 | ALM | Fasting glucose | 0.0117 | 31129883 | 0.002 | 0.0022 | 0.0019 | 0.0785308 |
| rs951366 | ALM | Fasting glucose | 0.0205 | 205685352 | 0.0019 | 0.0026 | 0.0019 | 0.1039 |
| rs9517483 | ALM | Fasting glucose | -0.0181 | 99572712 | 0.0021 | -0.0008 | 0.0019 | 0.697 |
| rs9525326 | ALM | Fasting glucose | -0.0184 | 115075715 | 0.0024 | -0.0009 | 0.0026 | 0.8052 |
| rs9568031 | ALM | Fasting glucose | -0.0115 | 48897520 | 0.0021 | 0.0008 | 0.002 | 0.8244 |
| rs9590328 | ALM | Fasting glucose | 0.0153 | 96448383 | 0.0027 | -0.0031 | 0.0026 | 0.2539 |
| rs9594714 | ALM | Fasting glucose | 0.0144 | 42800481 | 0.0021 | 0.0002 | 0.002 | 0.9798 |
| rs9610447 | ALM | Fasting glucose | 0.0152 | 20768891 | 0.0022 | 0.0007 | 0.0021 | 0.6555 |
| rs963317 | ALM | Fasting glucose | -0.0136 | 45129970 | 0.002 | 0.0051 | 0.0017 | 0.01034 |
| rs9634212 | ALM | Fasting glucose | 0.0471 | 93993266 | 0.0023 | 0.0028 | 0.0022 | 0.382 |
| rs9636364 | ALM | Fasting glucose | 0.011 | 111992435 | 0.0019 | -0.001 | 0.0018 | 0.7013 |
| rs9640283 | ALM | Fasting glucose | -0.0119 | 150485659 | 0.0019 | -0.0013 | 0.0019 | 0.4554 |
| rs9647379 | ALM | Fasting glucose | 0.0215 | 171785168 | 0.0019 | 0.0032 | 0.002 | 0.0336504 |
| rs9669278 | ALM | Fasting glucose | -0.0496 | 66374587 | 0.0019 | 0.0069 | 0.0018 | 0.000178398 |
| rs9809116 | ALM | Fasting glucose | -0.016 | 72397279 | 0.0019 | -0.0009 | 0.0019 | 0.2303 |
| rs9828525 | ALM | Fasting glucose | 0.0121 | 61552810 | 0.0019 | -0.0002 | 0.0019 | 0.9363 |
| rs9832919 | ALM | Fasting glucose | -0.0179 | 132184526 | 0.002 | 0.0002 | 0.0019 | 0.697 |
| rs9838614 | ALM | Fasting glucose | -0.0185 | 38537671 | 0.0019 | -0.0023 | 0.0017 | 0.3861 |
| rs985136 | ALM | Fasting glucose | 0.0138 | 17497794 | 0.002 | -0.0007 | 0.0022 | 0.590599 |
| rs987666 | ALM | Fasting glucose | 0.0185 | 116267938 | 0.0029 | 0.0011 | 0.0025 | 0.7523 |
| rs9890062 | ALM | Fasting glucose | 0.0267 | 17434352 | 0.0039 | -0.0018 | 0.004 | 0.9597 |
| rs9894577 | ALM | Fasting glucose | -0.031 | 43223292 | 0.002 | 0.0045 | 0.0018 | 0.02246 |
| rs9898189 | ALM | Fasting glucose | -0.0163 | 80480516 | 0.0021 | 0.0035 | 0.002 | 0.0725705 |
| rs990315 | ALM | Fasting glucose | -0.0115 | 69578811 | 0.002 | 0.0024 | 0.0019 | 0.0785308 |
| rs9905385 | ALM | Fasting glucose | -0.0339 | 59498250 | 0.002 | -0.0047 | 0.002 | 0.0228502 |
| rs9957318 | ALM | Fasting glucose | 0.0187 | 33039106 | 0.002 | 0.0023 | 0.0018 | 0.621901 |
| rs10005035 | ALM | Fasting insulin | -0.0175 | 0.0021 | 6.51328E-17 | -0.0036 | 0.0023 | 0.0985598 |
| rs10019221 | ALM | Fasting insulin | -0.0124 | 0.0019 | 1.22101E-10 | -0.0013 | 0.0021 | 0.766399 |
| rs1005723 | ALM | Fasting insulin | 0.0161 | 0.0024 | 1.7869E-11 | 0.0032 | 0.0028 | 0.3898 |
| rs10068640 | ALM | Fasting insulin | 0.0112 | 0.002 | 1.28201E-08 | 0.0056 | 0.0021 | 0.00296702 |
| rs10075249 | ALM | Fasting insulin | 0.0143 | 0.0019 | 4.55827E-14 | -0.0008 | 0.002 | 0.6067 |
| rs10107388 | ALM | Fasting insulin | -0.0159 | 0.002 | 6.95344E-16 | 0.0022 | 0.0022 | 0.4047 |
| rs10112506 | ALM | Fasting insulin | -0.012 | 0.0019 | 5.75705E-10 | -0.0001 | 0.0021 | 0.783901 |
| rs10123619 | ALM | Fasting insulin | -0.0171 | 0.0026 | 4.05042E-11 | 0.0065 | 0.0029 | 0.0556404 |
| rs10202845 | ALM | Fasting insulin | -0.0288 | 0.003 | 5.34564E-22 | 0.0041 | 0.0031 | 0.1362 |
| rs10203320 | ALM | Fasting insulin | 0.0138 | 0.002 | 7.85597E-12 | 0.003 | 0.0023 | 0.2678 |
| rs10205141 | ALM | Fasting insulin | 0.0241 | 0.0044 | 4.70999E-08 | -0.0021 | 0.0042 | 0.6514 |
| rs10221831 | ALM | Fasting insulin | 0.03 | 0.0053 | 1.80302E-08 | 0.0052 | 0.0054 | 0.2361 |
| rs1035583 | ALM | Fasting insulin | 0.0148 | 0.0019 | 1.99205E-14 | 0.0007 | 0.0021 | 0.909 |
| rs10421750 | ALM | Fasting insulin | -0.0145 | 0.0021 | 5.72796E-12 | 0.0028 | 0.0089 | 0.7405 |
| rs10461725 | ALM | Fasting insulin | 0.0134 | 0.002 | 1.9829E-11 | -0.0023 | 0.0022 | 0.1375 |
| rs10471339 | ALM | Fasting insulin | -0.011 | 0.0019 | 1.448E-08 | -0.0015 | 0.0021 | 0.1952 |
| rs1056747 | ALM | Fasting insulin | -0.0155 | 0.0019 | 8.04822E-16 | -0.0018 | 0.0021 | 0.3257 |
| rs1063582 | ALM | Fasting insulin | -0.0185 | 0.0022 | 1.12409E-16 | -0.0012 | 0.0026 | 0.4523 |
| rs10657263 | ALM | Fasting insulin | -0.013 | 0.0019 | 7.97995E-12 | -0.0008 | 0.0023 | 0.564 |
| rs10748128 | ALM | Fasting insulin | 0.0255 | 0.002 | 6.77174E-38 | 0.0049 | 0.002 | 0.00726106 |
| rs10749157 | ALM | Fasting insulin | 0.0113 | 0.002 | 1.239E-08 | -0.0014 | 0.002 | 0.454 |
| rs10776560 | ALM | Fasting insulin | -0.0157 | 0.0019 | 7.87952E-17 | 0.0034 | 0.0021 | 0.1342 |
| rs10793931 | ALM | Fasting insulin | -0.0132 | 0.002 | 3.23221E-11 | -0.002 | 0.0021 | 0.639 |
| rs10796828 | ALM | Fasting insulin | 0.0154 | 0.002 | 5.77431E-15 | -0.0036 | 0.0022 | 0.1594 |
| rs10822117 | ALM | Fasting insulin | -0.0176 | 0.0022 | 4.24033E-15 | -0.0015 | 0.0025 | 0.2797 |
| rs10824307 | ALM | Fasting insulin | -0.0194 | 0.002 | 1.78896E-22 | -0.0054 | 0.0022 | 0.0259102 |
| rs10829226 | ALM | Fasting insulin | -0.0112 | 0.002 | 1.32999E-08 | 0.0023 | 0.0021 | 0.4344 |
| rs10832963 | ALM | Fasting insulin | -0.0203 | 0.0022 | 9.22571E-21 | 0.0022 | 0.0022 | 0.3153 |
| rs10845408 | ALM | Fasting insulin | 0.0255 | 0.002 | 3.25387E-38 | 0.0032 | 0.0022 | 0.1802 |
| rs10864899 | ALM | Fasting insulin | -0.0112 | 0.0019 | 3.84503E-09 | 0.0035 | 0.0022 | 0.1594 |
| rs10975935 | ALM | Fasting insulin | -0.0121 | 0.0022 | 4.15002E-08 | -0.0031 | 0.0023 | 0.1014 |
| rs10982888 | ALM | Fasting insulin | -0.0328 | 0.003 | 3.96643E-28 | 0.002 | 0.0034 | 0.5438 |
| rs11009928 | ALM | Fasting insulin | -0.0148 | 0.0022 | 1.03801E-11 | 0.006 | 0.0091 | 0.516799 |
| rs11042717 | ALM | Fasting insulin | -0.029 | 0.0019 | 4.04297E-53 | 0.001 | 0.0018 | 0.485 |
| rs11049704 | ALM | Fasting insulin | -0.0183 | 0.0021 | 1.10306E-18 | -0.0044 | 0.0069 | 0.2894 |
| rs11060942 | ALM | Fasting insulin | -0.0354 | 0.0052 | 7.49031E-12 | 0.0046 | 0.0054 | 0.1581 |
| rs11068230 | ALM | Fasting insulin | 0.0238 | 0.0028 | 9.67386E-18 | -0.0005 | 0.0033 | 0.856 |
| rs11070842 | ALM | Fasting insulin | -0.0146 | 0.0026 | 1.30101E-08 | -0.0053 | 0.0029 | 0.0557596 |
| rs11098677 | ALM | Fasting insulin | -0.0263 | 0.0023 | 3.93731E-30 | 0.0102 | 0.0025 | 3.50598E-06 |
| rs11121615 | ALM | Fasting insulin | -0.0202 | 0.002 | 3.31894E-23 | -0.0011 | 0.0023 | 0.4863 |
| rs111622870 | ALM | Fasting insulin | -0.0282 | 0.0044 | 1.85999E-10 | -0.0063 | 0.0057 | 0.1852 |
| rs11175919 | ALM | Fasting insulin | 0.0349 | 0.0059 | 3.159E-09 | -0.0003 | 0.0063 | 0.9205 |
| rs11178643 | ALM | Fasting insulin | 0.0109 | 0.002 | 4.43997E-08 | 0.0141 | 0.0085 | 0.0741208 |
| rs11191208 | ALM | Fasting insulin | 0.0147 | 0.0024 | 3.68901E-10 | 0.0045 | 0.0027 | 0.138 |
| rs11198591 | ALM | Fasting insulin | 0.0148 | 0.002 | 4.90795E-14 | 0.0023 | 0.0019 | 0.5888 |
| rs112021215 | ALM | Fasting insulin | -0.0147 | 0.0025 | 5.99198E-09 | 0.0052 | 0.0032 | 0.1866 |
| rs112153300 | ALM | Fasting insulin | 0.0261 | 0.0034 | 7.05342E-15 | -0.0003 | 0.004 | 0.758799 |
| rs11217863 | ALM | Fasting insulin | -0.0268 | 0.003 | 1.06292E-19 | 0.0022 | 0.0034 | 0.4418 |
| rs11221657 | ALM | Fasting insulin | 0.0179 | 0.0028 | 1.20501E-10 | -0.0005 | 0.0031 | 0.428 |
| rs11233117 | ALM | Fasting insulin | -0.0176 | 0.0019 | 1.74181E-20 | 0.0015 | 0.0021 | 0.3727 |
| rs112537273 | ALM | Fasting insulin | -0.0212 | 0.0022 | 3.34426E-21 | 0.002 | 0.0026 | 0.3859 |
| rs11260035 | ALM | Fasting insulin | 0.015 | 0.0021 | 1.85609E-12 | 0.0096 | 0.0023 | 0.000210698 |
| rs11260623 | ALM | Fasting insulin | 0.0117 | 0.0019 | 4.85601E-10 | -0.0063 | 0.0021 | 0.00427799 |
| rs113146332 | ALM | Fasting insulin | 0.0311 | 0.0049 | 2.98696E-10 | -0.0115 | 0.0063 | 0.0581206 |
| rs113289555 | ALM | Fasting insulin | -0.0206 | 0.0023 | 7.32656E-20 | -0.0005 | 0.0034 | 0.9766 |
| rs113827862 | ALM | Fasting insulin | -0.0235 | 0.004 | 4.66702E-09 | -0.0049 | 0.0043 | 0.1953 |
| rs115010283 | ALM | Fasting insulin | 0.034 | 0.002 | 2.3518E-63 | 0.0058 | 0.0086 | 0.3952 |
| rs115105539 | ALM | Fasting insulin | 0.0231 | 0.0025 | 7.82528E-20 | -0.0115 | 0.0106 | 0.2699 |
| rs11562101 | ALM | Fasting insulin | 0.0115 | 0.002 | 9.38599E-09 | -0.0041 | 0.0033 | 0.1215 |
| rs11580040 | ALM | Fasting insulin | 0.0325 | 0.0035 | 6.76083E-21 | 0.0068 | 0.0043 | 0.2657 |
| rs115912456 | ALM | Fasting insulin | 0.0577 | 0.0047 | 3.69233E-34 | -0.0101 | 0.0054 | 0.0404604 |
| rs116008080 | ALM | Fasting insulin | -0.0415 | 0.0063 | 4.05695E-11 | -0.0046 | 0.0081 | 0.542 |
| rs116052377 | ALM | Fasting insulin | 0.0225 | 0.0035 | 7.89042E-11 | -0.0019 | 0.0039 | 0.696101 |
| rs116092985 | ALM | Fasting insulin | -0.0401 | 0.0033 | 1.16788E-34 | -0.004 | 0.0052 | 0.4137 |
| rs11612462 | ALM | Fasting insulin | 0.015 | 0.0025 | 2.54701E-09 | 0.0048 | 0.0028 | 0.1183 |
| rs11629593 | ALM | Fasting insulin | -0.0109 | 0.002 | 4.42201E-08 | -0.0058 | 0.0023 | 0.02666 |
| rs116339650 | ALM | Fasting insulin | -0.0175 | 0.0029 | 1.05201E-09 | 0.0055 | 0.0032 | 0.0409996 |
| rs116493405 | ALM | Fasting insulin | 0.0287 | 0.0042 | 9.52138E-12 | -0.0001 | 0.0047 | 0.898 |
| rs11672848 | ALM | Fasting insulin | -0.0171 | 0.0019 | 7.73393E-19 | 0.0039 | 0.0021 | 0.0419402 |
| rs11684531 | ALM | Fasting insulin | -0.0172 | 0.0028 | 4.16697E-10 | -0.0035 | 0.0078 | 0.4248 |
| rs1168768 | ALM | Fasting insulin | 0.0332 | 0.006 | 3.60903E-08 | -0.0058 | 0.0084 | 0.4926 |
| rs116919274 | ALM | Fasting insulin | 0.0271 | 0.0046 | 3.52298E-09 | 0.0035 | 0.0068 | 0.9641 |
| rs117203652 | ALM | Fasting insulin | -0.0346 | 0.0055 | 4.31599E-10 | 0.003 | 0.0088 | 0.8366 |
| rs11720869 | ALM | Fasting insulin | 0.0141 | 0.002 | 2.53805E-12 | 0.0015 | 0.0022 | 0.623199 |
| rs11721522 | ALM | Fasting insulin | 0.0106 | 0.0019 | 4.03005E-08 | 0.0035 | 0.0021 | 0.1775 |
| rs11727162 | ALM | Fasting insulin | -0.017 | 0.0019 | 2.15477E-19 | -0.002 | 0.002 | 0.3624 |
| rs117335233 | ALM | Fasting insulin | -0.0236 | 0.0042 | 2.561E-08 | 0.0044 | 0.006 | 0.3764 |
| rs117818446 | ALM | Fasting insulin | 0.0423 | 0.0068 | 5.28896E-10 | -0.0164 | 0.0093 | 0.1569 |
| rs117972846 | ALM | Fasting insulin | 0.0335 | 0.0057 | 5.47293E-09 | -0.0016 | 0.0071 | 0.6772 |
| rs11867855 | ALM | Fasting insulin | -0.0132 | 0.0022 | 2.93103E-09 | 0.0069 | 0.0025 | 0.003404 |
| rs1202186 | ALM | Fasting insulin | -0.012 | 0.002 | 1.65299E-09 | 0.0038 | 0.0022 | 0.1061 |
| rs12185775 | ALM | Fasting insulin | -0.0167 | 0.003 | 3.661E-08 | -0.0005 | 0.0036 | 0.7671 |
| rs12230946 | ALM | Fasting insulin | 0.0271 | 0.0033 | 1.45011E-16 | 0.0017 | 0.0033 | 0.672 |
| rs12334478 | ALM | Fasting insulin | -0.0161 | 0.0019 | 2.26621E-17 | -0.0012 | 0.0021 | 0.4974 |
| rs12340775 | ALM | Fasting insulin | -0.0287 | 0.0043 | 1.73181E-11 | -0.0046 | 0.0044 | 0.1926 |
| rs12423821 | ALM | Fasting insulin | 0.0161 | 0.0027 | 1.17201E-09 | -0.0065 | 0.0042 | 0.1696 |
| rs12483401 | ALM | Fasting insulin | -0.0387 | 0.0067 | 9.22104E-09 | -0.0059 | 0.0075 | 0.5309 |
| rs12512942 | ALM | Fasting insulin | -0.0162 | 0.002 | 1.58016E-16 | 0.0021 | 0.0021 | 0.5075 |
| rs12517711 | ALM | Fasting insulin | -0.0147 | 0.0019 | 2.78869E-14 | 0.0021 | 0.0021 | 0.1775 |
| rs12519407 | ALM | Fasting insulin | 0.0181 | 0.0022 | 3.3752E-17 | 0.002 | 0.0022 | 0.2952 |
| rs12533452 | ALM | Fasting insulin | 0.0237 | 0.0026 | 1.31401E-19 | -0.0018 | 0.003 | 0.4872 |
| rs12536902 | ALM | Fasting insulin | 0.0479 | 0.0081 | 3.65603E-09 | 0.0001 | 0.0095 | 0.5547 |
| rs12563442 | ALM | Fasting insulin | 0.0122 | 0.0021 | 9.93299E-09 | 0.0017 | 0.0023 | 0.5497 |
| rs12616192 | ALM | Fasting insulin | -0.0261 | 0.0038 | 6.82967E-12 | -0.0007 | 0.0044 | 0.8206 |
| rs12655296 | ALM | Fasting insulin | -0.011 | 0.002 | 1.62002E-08 | -0.0005 | 0.0021 | 0.9453 |
| rs12702693 | ALM | Fasting insulin | 0.0173 | 0.0019 | 6.56145E-20 | -0.0008 | 0.002 | 0.9813 |
| rs12724708 | ALM | Fasting insulin | 0.0243 | 0.002 | 1.65615E-35 | 0.007 | 0.002 | 0.001242 |
| rs12907139 | ALM | Fasting insulin | -0.0149 | 0.0019 | 4.89328E-15 | 0.001 | 0.0019 | 0.5071 |
| rs1291114 | ALM | Fasting insulin | 0.0173 | 0.0031 | 1.94599E-08 | -0.0039 | 0.0038 | 0.1974 |
| rs12926103 | ALM | Fasting insulin | 0.0272 | 0.0038 | 9.59843E-13 | -0.002 | 0.0044 | 0.1739 |
| rs12943867 | ALM | Fasting insulin | 0.0184 | 0.002 | 7.56833E-20 | -0.0023 | 0.003 | 0.3956 |
| rs13109280 | ALM | Fasting insulin | 0.0131 | 0.002 | 9.14534E-11 | -0.0024 | 0.0022 | 0.3257 |
| rs13127468 | ALM | Fasting insulin | -0.0123 | 0.0019 | 9.85825E-11 | -0.0025 | 0.0025 | 0.1923 |
| rs13170063 | ALM | Fasting insulin | -0.0152 | 0.0019 | 4.10771E-15 | -0.0042 | 0.0019 | 0.0190999 |
| rs1324538 | ALM | Fasting insulin | 0.0237 | 0.0019 | 1.73301E-34 | 0.0019 | 0.0022 | 0.5554 |
| rs13391980 | ALM | Fasting insulin | -0.0225 | 0.0029 | 7.59801E-15 | -0.0287 | 0.003 | 3.95367E-24 |
| rs13430869 | ALM | Fasting insulin | 0.0272 | 0.0021 | 6.36649E-37 | -0.0025 | 0.0022 | 0.1783 |
| rs139163241 | ALM | Fasting insulin | -0.0164 | 0.0027 | 1.78599E-09 | 0.003 | 0.0037 | 0.3191 |
| rs139921635 | ALM | Fasting insulin | 0.0385 | 0.0062 | 6.15602E-10 | 0.0054 | 0.0124 | 0.7854 |
| rs1405227 | ALM | Fasting insulin | 0.0129 | 0.002 | 1.573E-10 | -0.0011 | 0.0022 | 0.8643 |
| rs143076454 | ALM | Fasting insulin | -0.0499 | 0.007 | 1.05512E-12 | -0.0338 | 0.0133 | 0.0162499 |
| rs143554698 | ALM | Fasting insulin | -0.0257 | 0.0027 | 3.39391E-21 | 0.008 | 0.0033 | 0.0131501 |
| rs144109601 | ALM | Fasting insulin | -0.0278 | 0.0048 | 5.15003E-09 | 0.0105 | 0.0051 | 0.0209402 |
| rs1444628 | ALM | Fasting insulin | 0.024 | 0.002 | 6.84542E-32 | 0.0011 | 0.0022 | 0.6081 |
| rs144627572 | ALM | Fasting insulin | 0.0439 | 0.0053 | 1.30197E-16 | -0.0024 | 0.0064 | 0.9069 |
| rs1478575 | ALM | Fasting insulin | 0.0312 | 0.002 | 5.20236E-54 | -0.0046 | 0.0021 | 0.0576196 |
| rs14976 | ALM | Fasting insulin | 0.0144 | 0.002 | 1.42692E-12 | -0.0033 | 0.0023 | 0.0191801 |
| rs1514134 | ALM | Fasting insulin | -0.0114 | 0.0019 | 3.57396E-09 | -0.0012 | 0.002 | 0.4682 |
| rs1556659 | ALM | Fasting insulin | 0.0163 | 0.002 | 7.18952E-17 | -0.0021 | 0.002 | 0.1795 |
| rs1584011 | ALM | Fasting insulin | 0.0159 | 0.002 | 9.77687E-16 | 0.0007 | 0.0021 | 0.5372 |
| rs165849 | ALM | Fasting insulin | 0.0157 | 0.0021 | 4.56983E-14 | 0.0012 | 0.0022 | 0.4292 |
| rs17197114 | ALM | Fasting insulin | 0.0177 | 0.0025 | 1.54383E-12 | -0.0034 | 0.0032 | 0.5638 |
| rs17205463 | ALM | Fasting insulin | -0.0263 | 0.0019 | 4.2092E-43 | 0.0022 | 0.002 | 0.1542 |
| rs17246129 | ALM | Fasting insulin | 0.0254 | 0.002 | 1.26911E-35 | 0.0053 | 0.0021 | 0.0250998 |
| rs17278379 | ALM | Fasting insulin | 0.0226 | 0.0029 | 2.39773E-15 | 0.0043 | 0.003 | 0.1427 |
| rs173135 | ALM | Fasting insulin | -0.0341 | 0.003 | 3.24564E-30 | 0.0049 | 0.0034 | 0.1165 |
| rs17408561 | ALM | Fasting insulin | 0.0123 | 0.002 | 5.99998E-10 | -0.0005 | 0.0082 | 0.8221 |
| rs17478946 | ALM | Fasting insulin | -0.0192 | 0.0021 | 9.98389E-21 | -0.0026 | 0.0023 | 0.0493503 |
| rs17681189 | ALM | Fasting insulin | -0.0131 | 0.0019 | 5.81969E-12 | -0.0055 | 0.002 | 0.00575997 |
| rs177591 | ALM | Fasting insulin | -0.0191 | 0.0027 | 1.51286E-12 | 0.002 | 0.0032 | 0.3823 |
| rs17773965 | ALM | Fasting insulin | -0.0163 | 0.0027 | 1.50699E-09 | 0.0032 | 0.0029 | 0.3341 |
| rs1786263 | ALM | Fasting insulin | -0.019 | 0.0019 | 1.02589E-22 | 0.0023 | 0.0021 | 0.2531 |
| rs182798714 | ALM | Fasting insulin | 0.0376 | 0.0062 | 1.45101E-09 | -0.0158 | 0.0078 | 0.0194599 |
| rs1880318 | ALM | Fasting insulin | 0.0147 | 0.0024 | 6.85899E-10 | -0.0052 | 0.0027 | 0.0854594 |
| rs188617336 | ALM | Fasting insulin | 0.0138 | 0.0021 | 6.6558E-11 | -0.0054 | 0.0078 | 0.4938 |
| rs1899040 | ALM | Fasting insulin | 0.0152 | 0.0023 | 9.04066E-11 | 0.0025 | 0.0026 | 0.2054 |
| rs190823861 | ALM | Fasting insulin | -0.0345 | 0.0045 | 2.08882E-14 | 0.0108 | 0.0058 | 0.1715 |
| rs1933081 | ALM | Fasting insulin | 0.0267 | 0.0034 | 5.32844E-15 | -0.0013 | 0.0038 | 0.8544 |
| rs199647708 | ALM | Fasting insulin | 0.0114 | 0.0019 | 3.43297E-09 | -0.0135 | 0.0082 | 0.1018 |
| rs200439 | ALM | Fasting insulin | -0.0128 | 0.0023 | 1.51001E-08 | 0.0006 | 0.0025 | 0.953 |
| rs200739311 | ALM | Fasting insulin | -0.0128 | 0.002 | 1.55299E-10 | 0.013 | 0.0085 | 0.0824594 |
| rs2019203 | ALM | Fasting insulin | 0.0189 | 0.0019 | 1.8412E-23 | 0.0014 | 0.0021 | 0.837 |
| rs2025808 | ALM | Fasting insulin | 0.0122 | 0.0022 | 1.71601E-08 | 0.004 | 0.0023 | 0.0854594 |
| rs2070598 | ALM | Fasting insulin | 0.0204 | 0.0019 | 6.35624E-27 | -0.0001 | 0.002 | 0.8699 |
| rs2089111 | ALM | Fasting insulin | -0.0172 | 0.0022 | 1.72584E-15 | -0.008 | 0.0026 | 0.00149799 |
| rs2101017 | ALM | Fasting insulin | -0.0223 | 0.0028 | 1.44411E-15 | -0.0003 | 0.0029 | 0.8521 |
| rs212526 | ALM | Fasting insulin | 0.0214 | 0.0019 | 3.84326E-29 | 0.0031 | 0.0021 | 0.0920407 |
| rs2138374 | ALM | Fasting insulin | -0.0149 | 0.002 | 2.78997E-13 | 0.0005 | 0.0022 | 0.9826 |
| rs2142331 | ALM | Fasting insulin | -0.0165 | 0.0019 | 1.37784E-17 | -0.0098 | 0.002 | 0.00001434 |
| rs2142644 | ALM | Fasting insulin | -0.0181 | 0.002 | 3.37287E-19 | 0.0048 | 0.0022 | 0.1078 |
| rs2174008 | ALM | Fasting insulin | -0.0192 | 0.0019 | 5.89251E-24 | -0.0027 | 0.0021 | 0.1927 |
| rs2181834 | ALM | Fasting insulin | 0.0254 | 0.0019 | 7.69485E-41 | 0.0028 | 0.002 | 0.1644 |
| rs2188805 | ALM | Fasting insulin | 0.0114 | 0.002 | 2.003E-08 | 0.0041 | 0.0023 | 0.227 |
| rs2212926 | ALM | Fasting insulin | -0.022 | 0.0023 | 7.74819E-21 | -0.0005 | 0.0033 | 0.9877 |
| rs2230033 | ALM | Fasting insulin | -0.0265 | 0.0019 | 3.48819E-43 | 0.0066 | 0.0019 | 0.000258297 |
| rs2236096 | ALM | Fasting insulin | 0.018 | 0.0023 | 1.28795E-15 | -0.0012 | 0.0026 | 0.3163 |
| rs2237485 | ALM | Fasting insulin | 0.0191 | 0.0023 | 3.73422E-17 | -0.0111 | 0.0025 | 1.53102E-05 |
| rs2240735 | ALM | Fasting insulin | 0.0189 | 0.0022 | 3.98566E-18 | -0.001 | 0.0025 | 0.7181 |
| rs2283200 | ALM | Fasting insulin | -0.0281 | 0.0042 | 1.48115E-11 | 0.0014 | 0.0041 | 0.7969 |
| rs2287821 | ALM | Fasting insulin | -0.0153 | 0.0019 | 6.95344E-16 | -0.0066 | 0.0019 | 0.000485099 |
| rs2303423 | ALM | Fasting insulin | 0.0168 | 0.003 | 2.64399E-08 | 0.0016 | 0.0034 | 0.8233 |
| rs2305141 | ALM | Fasting insulin | 0.0183 | 0.0019 | 1.07498E-21 | -0.0023 | 0.0021 | 0.5222 |
| rs2324154 | ALM | Fasting insulin | 0.015 | 0.0019 | 1.91602E-15 | 0.0015 | 0.002 | 0.3377 |
| rs234640 | ALM | Fasting insulin | -0.0131 | 0.0019 | 3.86812E-12 | 0.0001 | 0.002 | 0.8699 |
| rs2347603 | ALM | Fasting insulin | -0.0181 | 0.0022 | 5.64547E-17 | -0.0013 | 0.0023 | 0.9022 |
| rs2347808 | ALM | Fasting insulin | -0.0125 | 0.0019 | 5.78762E-11 | 0.0005 | 0.002 | 0.8856 |
| rs2362487 | ALM | Fasting insulin | 0.0154 | 0.0022 | 3.57026E-12 | -0.0076 | 0.0026 | 0.00247002 |
| rs2454390 | ALM | Fasting insulin | -0.0176 | 0.0026 | 1.7398E-11 | 0.0015 | 0.003 | 0.7475 |
| rs246177 | ALM | Fasting insulin | 0.0214 | 0.002 | 2.03517E-27 | -0.0038 | 0.0021 | 0.0983603 |
| rs2490302 | ALM | Fasting insulin | 0.0221 | 0.0034 | 6.23735E-11 | -0.0001 | 0.0036 | 0.5301 |
| rs2521349 | ALM | Fasting insulin | 0.0155 | 0.0019 | 2.01419E-15 | -0.0009 | 0.0021 | 0.5833 |
| rs2545339 | ALM | Fasting insulin | 0.0115 | 0.002 | 3.48001E-09 | 0.0019 | 0.0021 | 0.2193 |
| rs2569888 | ALM | Fasting insulin | 0.0133 | 0.0022 | 2.29298E-09 | 0.0044 | 0.0026 | 0.0820805 |
| rs2578565 | ALM | Fasting insulin | -0.0141 | 0.002 | 1.37404E-12 | 0.0044 | 0.0021 | 0.0709905 |
| rs258794 | ALM | Fasting insulin | 0.0147 | 0.0021 | 6.23448E-12 | 0.0143 | 0.0091 | 0.1108 |
| rs2592208 | ALM | Fasting insulin | -0.0124 | 0.0019 | 5.51696E-11 | 0.0048 | 0.002 | 0.0227798 |
| rs2607234 | ALM | Fasting insulin | -0.0302 | 0.0043 | 1.99618E-12 | 0.0067 | 0.0047 | 0.1721 |
| rs261223 | ALM | Fasting insulin | 0.0175 | 0.0019 | 2.29985E-19 | -0.0033 | 0.0021 | 0.0952292 |
| rs2663126 | ALM | Fasting insulin | -0.0139 | 0.0021 | 1.35613E-11 | -0.0029 | 0.0023 | 0.3467 |
| rs2717008 | ALM | Fasting insulin | -0.0127 | 0.0019 | 4.9877E-11 | -0.0012 | 0.0021 | 0.4786 |
| rs2748501 | ALM | Fasting insulin | -0.0195 | 0.0019 | 1.31099E-24 | -0.0019 | 0.002 | 0.778901 |
| rs2754255 | ALM | Fasting insulin | -0.0153 | 0.0023 | 1.04689E-11 | 0.0028 | 0.0026 | 0.2034 |
| rs2763263 | ALM | Fasting insulin | -0.017 | 0.0022 | 1.37088E-14 | 0.0014 | 0.0025 | 0.8254 |
| rs2764264 | ALM | Fasting insulin | 0.0203 | 0.0021 | 5.07341E-23 | -0.001 | 0.0022 | 0.8142 |
| rs2788213 | ALM | Fasting insulin | 0.0123 | 0.0021 | 3.79603E-09 | 0.0019 | 0.0022 | 0.913 |
| rs2791654 | ALM | Fasting insulin | -0.0239 | 0.0022 | 1.20587E-28 | -0.0001 | 0.0091 | 0.979 |
| rs28379706 | ALM | Fasting insulin | 0.0114 | 0.002 | 4.49004E-09 | 0.0005 | 0.0029 | 0.8745 |
| rs28485212 | ALM | Fasting insulin | -0.0188 | 0.0027 | 1.24194E-12 | 0.0027 | 0.0037 | 0.2092 |
| rs28529055 | ALM | Fasting insulin | -0.0147 | 0.0019 | 1.87888E-14 | -0.0038 | 0.0021 | 0.0299102 |
| rs28529426 | ALM | Fasting insulin | -0.0168 | 0.0026 | 5.82371E-11 | -0.0011 | 0.0034 | 0.5928 |
| rs28678024 | ALM | Fasting insulin | -0.0119 | 0.0021 | 1.89898E-08 | -0.0003 | 0.0022 | 0.898 |
| rs28736838 | ALM | Fasting insulin | -0.0117 | 0.002 | 1.06999E-08 | 0.0027 | 0.0025 | 0.457999 |
| rs291979 | ALM | Fasting insulin | 0.0242 | 0.0023 | 6.75616E-27 | 0.0038 | 0.0026 | 0.2975 |
| rs2925155 | ALM | Fasting insulin | -0.015 | 0.0022 | 5.46638E-12 | -0.0044 | 0.0021 | 0.0357701 |
| rs293517 | ALM | Fasting insulin | -0.013 | 0.0021 | 2.80401E-10 | 0.0044 | 0.0023 | 0.0545004 |
| rs2978362 | ALM | Fasting insulin | 0.0106 | 0.0019 | 2.848E-08 | 0.0021 | 0.002 | 0.3615 |
| rs310796 | ALM | Fasting insulin | 0.0142 | 0.002 | 2.53396E-12 | -0.0073 | 0.002 | 0.000753009 |
| rs331917 | ALM | Fasting insulin | -0.0127 | 0.0019 | 3.5416E-11 | 0.0026 | 0.0023 | 0.3472 |
| rs336630 | ALM | Fasting insulin | -0.0106 | 0.0019 | 2.89701E-08 | 0.0056 | 0.0023 | 0.0277703 |
| rs33973388 | ALM | Fasting insulin | 0.0249 | 0.0019 | 1.44611E-38 | 0.0008 | 0.002 | 0.6488 |
| rs34287 | ALM | Fasting insulin | 0.0187 | 0.002 | 1.17193E-20 | -0.0013 | 0.0021 | 0.7208 |
| rs34312629 | ALM | Fasting insulin | -0.017 | 0.0021 | 2.12324E-15 | -0.0009 | 0.0023 | 0.6087 |
| rs34338597 | ALM | Fasting insulin | -0.0112 | 0.0019 | 7.88007E-09 | -0.0003 | 0.0021 | 0.8015 |
| rs34345560 | ALM | Fasting insulin | 0.0219 | 0.0024 | 7.09741E-20 | 0.008 | 0.0029 | 0.000343503 |
| rs34390533 | ALM | Fasting insulin | -0.0257 | 0.0022 | 6.69114E-32 | -0.0024 | 0.0025 | 0.1274 |
| rs34522021 | ALM | Fasting insulin | 0.0126 | 0.0019 | 3.37598E-11 | -0.004 | 0.002 | 0.0440595 |
| rs35073631 | ALM | Fasting insulin | 0.0112 | 0.0019 | 5.92298E-09 | -0.0007 | 0.0023 | 0.9833 |
| rs35268848 | ALM | Fasting insulin | 0.0737 | 0.0101 | 2.83204E-13 | -0.0006 | 0.008 | 0.9742 |
| rs35288270 | ALM | Fasting insulin | -0.0328 | 0.0028 | 3.43479E-32 | -0.0041 | 0.0031 | 0.2408 |
| rs35756741 | ALM | Fasting insulin | -0.0378 | 0.0033 | 5.80096E-31 | 0.0035 | 0.0036 | 0.158 |
| rs35811052 | ALM | Fasting insulin | -0.0148 | 0.0022 | 8.84097E-12 | 0.0029 | 0.0027 | 0.249 |
| rs35816944 | ALM | Fasting insulin | -0.1088 | 0.0117 | 1.2659E-20 | 0.0125 | 0.0175 | 0.5948 |
| rs36000545 | ALM | Fasting insulin | -0.022 | 0.002 | 2.558E-29 | -0.0062 | 0.0027 | 0.01073 |
| rs36012032 | ALM | Fasting insulin | 0.0298 | 0.0033 | 9.93116E-20 | -0.0117 | 0.0033 | 0.000209098 |
| rs36048468 | ALM | Fasting insulin | 0.0254 | 0.0023 | 9.33899E-28 | 0.0043 | 0.0026 | 0.1456 |
| rs373736365 | ALM | Fasting insulin | 0.0189 | 0.0022 | 4.53419E-18 | -0.0176 | 0.0093 | 0.0487596 |
| rs3764002 | ALM | Fasting insulin | 0.028 | 0.0021 | 4.47095E-39 | -0.0049 | 0.0021 | 0.0116001 |
| rs3768495 | ALM | Fasting insulin | -0.0178 | 0.0021 | 1.06611E-17 | -0.0017 | 0.002 | 0.3374 |
| rs3778858 | ALM | Fasting insulin | 0.0108 | 0.002 | 4.26197E-08 | 0.0052 | 0.0022 | 0.0172898 |
| rs3782232 | ALM | Fasting insulin | -0.0339 | 0.0037 | 2.40602E-20 | 0.0082 | 0.0048 | 0.1446 |
| rs3782811 | ALM | Fasting insulin | -0.0165 | 0.0022 | 3.95913E-14 | -0.0005 | 0.0025 | 0.794301 |
| rs3792819 | ALM | Fasting insulin | 0.021 | 0.0034 | 4.42201E-10 | -0.002 | 0.0037 | 0.5627 |
| rs3822742 | ALM | Fasting insulin | 0.0162 | 0.002 | 1.02707E-16 | 0.0021 | 0.0022 | 0.2846 |
| rs3828729 | ALM | Fasting insulin | -0.016 | 0.002 | 4.66874E-15 | -0.0049 | 0.0022 | 0.0144401 |
| rs3901421 | ALM | Fasting insulin | 0.0215 | 0.0019 | 7.54745E-30 | -0.0026 | 0.002 | 0.1874 |
| rs395980 | ALM | Fasting insulin | -0.0184 | 0.0021 | 1.02094E-17 | 0.0038 | 0.0021 | 0.05199 |
| rs40270 | ALM | Fasting insulin | 0.0151 | 0.0022 | 1.89802E-11 | 0.0185 | 0.0023 | 4.15336E-16 |
| rs4077103 | ALM | Fasting insulin | -0.0143 | 0.0026 | 4.17503E-08 | -0.0042 | 0.0028 | 0.1268 |
| rs4252548 | ALM | Fasting insulin | -0.0753 | 0.0065 | 2.95597E-31 | 0.0066 | 0.0108 | 0.609101 |
| rs4282339 | ALM | Fasting insulin | -0.0311 | 0.0023 | 6.16453E-41 | -0.0029 | 0.0023 | 0.1901 |
| rs4287835 | ALM | Fasting insulin | 0.0147 | 0.0019 | 9.94489E-15 | 0.0008 | 0.002 | 0.7256 |
| rs447352 | ALM | Fasting insulin | -0.0181 | 0.0029 | 6.62293E-10 | -0.004 | 0.0042 | 0.572 |
| rs4504126 | ALM | Fasting insulin | 0.046 | 0.0058 | 1.61585E-15 | -0.0038 | 0.0069 | 0.6721 |
| rs45528934 | ALM | Fasting insulin | 0.0262 | 0.0026 | 1.96517E-24 | -0.006 | 0.0033 | 0.0476497 |
| rs4602848 | ALM | Fasting insulin | 0.016 | 0.002 | 3.14485E-15 | 0.0035 | 0.0021 | 0.173 |
| rs4622329 | ALM | Fasting insulin | 0.0149 | 0.002 | 8.61986E-14 | -0.005 | 0.0021 | 0.0338501 |
| rs4640244 | ALM | Fasting insulin | -0.02 | 0.0019 | 3.80978E-25 | 0.0037 | 0.0021 | 0.1169 |
| rs4644481 | ALM | Fasting insulin | -0.0112 | 0.0019 | 3.54201E-09 | 0.0026 | 0.0021 | 0.1594 |
| rs4655345 | ALM | Fasting insulin | -0.0246 | 0.0019 | 5.81032E-38 | 0.0042 | 0.0021 | 0.0250998 |
| rs4682483 | ALM | Fasting insulin | -0.0165 | 0.0026 | 2.651E-10 | 0.0056 | 0.0028 | 0.0178299 |
| rs4683435 | ALM | Fasting insulin | 0.0144 | 0.0022 | 1.60498E-10 | -0.0023 | 0.0026 | 0.355 |
| rs4748008 | ALM | Fasting insulin | -0.0125 | 0.0019 | 8.94953E-11 | -0.0014 | 0.002 | 0.2807 |
| rs4752689 | ALM | Fasting insulin | 0.0205 | 0.0019 | 1.36301E-26 | -0.0054 | 0.0021 | 0.0493503 |
| rs4807472 | ALM | Fasting insulin | -0.0158 | 0.002 | 8.18276E-15 | 0.0029 | 0.0022 | 0.0761798 |
| rs4815952 | ALM | Fasting insulin | -0.0161 | 0.0019 | 1.23595E-16 | 0.0006 | 0.0021 | 0.8758 |
| rs4818280 | ALM | Fasting insulin | -0.0124 | 0.002 | 2.84302E-10 | 0.0008 | 0.0021 | 0.7376 |
| rs4865956 | ALM | Fasting insulin | -0.0258 | 0.0021 | 3.84769E-36 | -0.0092 | 0.0022 | 7.70407E-05 |
| rs4938359 | ALM | Fasting insulin | -0.0156 | 0.0024 | 3.23966E-11 | 0.0022 | 0.0025 | 0.1603 |
| rs4940874 | ALM | Fasting insulin | 0.0148 | 0.0024 | 1.244E-09 | -0.0006 | 0.0026 | 0.738599 |
| rs496783 | ALM | Fasting insulin | -0.0124 | 0.0019 | 8.12643E-11 | -0.0005 | 0.0021 | 0.5991 |
| rs532499 | ALM | Fasting insulin | -0.0127 | 0.0022 | 4.90004E-09 | 0.0031 | 0.0026 | 0.0731105 |
| rs544136 | ALM | Fasting insulin | 0.0121 | 0.0022 | 2.50098E-08 | -0.0044 | 0.0025 | 0.0710608 |
| rs545104 | ALM | Fasting insulin | 0.0127 | 0.002 | 8.08351E-11 | 0.0013 | 0.0021 | 0.2818 |
| rs55717234 | ALM | Fasting insulin | 0.0122 | 0.0019 | 1.341E-10 | 0.0011 | 0.0028 | 0.9715 |
| rs55758152 | ALM | Fasting insulin | 0.0145 | 0.002 | 1.05293E-12 | -0.0045 | 0.0028 | 0.2092 |
| rs55980611 | ALM | Fasting insulin | 0.0164 | 0.0028 | 4.21697E-09 | -0.001 | 0.0121 | 0.8817 |
| rs56112295 | ALM | Fasting insulin | 0.0154 | 0.0024 | 1.118E-10 | -0.0041 | 0.0044 | 0.6514 |
| rs56207600 | ALM | Fasting insulin | 0.0192 | 0.003 | 2.50098E-10 | 0.004 | 0.0032 | 0.3643 |
| rs56239180 | ALM | Fasting insulin | -0.0459 | 0.0062 | 1.85097E-13 | -0.001 | 0.0082 | 0.784899 |
| rs56363908 | ALM | Fasting insulin | -0.0382 | 0.0047 | 3.87793E-16 | -0.0039 | 0.0076 | 0.7568 |
| rs57059662 | ALM | Fasting insulin | 0.0118 | 0.002 | 5.63106E-09 | 0.0024 | 0.0021 | 0.1927 |
| rs57513571 | ALM | Fasting insulin | -0.0191 | 0.0024 | 7.02102E-16 | -0.0009 | 0.0028 | 0.986 |
| rs5753518 | ALM | Fasting insulin | 0.0242 | 0.0033 | 5.03849E-13 | 0.0026 | 0.0034 | 0.3189 |
| rs57696574 | ALM | Fasting insulin | 0.0173 | 0.002 | 4.50194E-18 | -0.0016 | 0.0022 | 0.6877 |
| rs577289 | ALM | Fasting insulin | -0.0125 | 0.0021 | 5.39399E-09 | 0.0043 | 0.0023 | 0.1832 |
| rs6000886 | ALM | Fasting insulin | 0.0131 | 0.002 | 5.88166E-11 | 0.0001 | 0.0022 | 0.9304 |
| rs6028716 | ALM | Fasting insulin | -0.021 | 0.0022 | 4.58247E-22 | -0.0003 | 0.0023 | 0.8507 |
| rs60389750 | ALM | Fasting insulin | -0.0175 | 0.0021 | 1.05706E-16 | -0.0015 | 0.0025 | 0.2981 |
| rs60408354 | ALM | Fasting insulin | 0.0259 | 0.0036 | 1.15213E-12 | 0.0054 | 0.0043 | 0.3091 |
| rs6082354 | ALM | Fasting insulin | -0.024 | 0.002 | 1.18987E-32 | -0.0033 | 0.0022 | 0.3366 |
| rs61397287 | ALM | Fasting insulin | 0.0235 | 0.0036 | 4.45349E-11 | -0.0108 | 0.0153 | 0.4946 |
| rs6142059 | ALM | Fasting insulin | 0.0116 | 0.0019 | 1.19001E-09 | 0.0036 | 0.002 | 0.0833508 |
| rs61528919 | ALM | Fasting insulin | 0.014 | 0.002 | 3.32889E-12 | 0.0162 | 0.0087 | 0.0596705 |
| rs61732778 | ALM | Fasting insulin | 0.023 | 0.0037 | 3.13199E-10 | 0.0104 | 0.0041 | 0.00559294 |
| rs61827272 | ALM | Fasting insulin | 0.0144 | 0.0021 | 7.89042E-12 | -0.001 | 0.0023 | 0.3257 |
| rs61878760 | ALM | Fasting insulin | 0.019 | 0.0034 | 3.73603E-08 | 0.0029 | 0.0041 | 0.5927 |
| rs61944841 | ALM | Fasting insulin | 0.0253 | 0.002 | 3.54079E-37 | -0.007 | 0.0026 | 0.007085 |
| rs62033029 | ALM | Fasting insulin | -0.0141 | 0.0023 | 1.726E-09 | -0.0004 | 0.0026 | 0.576301 |
| rs62070319 | ALM | Fasting insulin | -0.018 | 0.002 | 2.54097E-20 | 0.0048 | 0.0082 | 0.6048 |
| rs62103240 | ALM | Fasting insulin | 0.0212 | 0.0037 | 1.39701E-08 | -0.0075 | 0.0053 | 0.1269 |
| rs62106258 | ALM | Fasting insulin | -0.0504 | 0.0044 | 6.44763E-31 | 0.0181 | 0.0079 | 0.0159302 |
| rs62143873 | ALM | Fasting insulin | -0.0115 | 0.0019 | 1.193E-09 | -0.004 | 0.0021 | 0.1148 |
| rs62466110 | ALM | Fasting insulin | -0.0371 | 0.0041 | 5.74116E-20 | 0.003 | 0.0055 | 0.3654 |
| rs6502935 | ALM | Fasting insulin | -0.0125 | 0.0022 | 6.31495E-09 | -0.0011 | 0.0026 | 0.9693 |
| rs655113 | ALM | Fasting insulin | 0.0188 | 0.0021 | 7.41822E-20 | 0.0029 | 0.0022 | 0.1557 |
| rs6582398 | ALM | Fasting insulin | 0.014 | 0.002 | 1.13501E-12 | -0.0036 | 0.0022 | 0.0493503 |
| rs6593210 | ALM | Fasting insulin | 0.0146 | 0.0024 | 4.88203E-10 | 0.0001 | 0.0023 | 0.5391 |
| rs664317 | ALM | Fasting insulin | -0.0177 | 0.0026 | 4.49573E-12 | 0.0028 | 0.0029 | 0.4378 |
| rs6693481 | ALM | Fasting insulin | -0.0143 | 0.002 | 2.208E-12 | -0.0007 | 0.0022 | 0.7812 |
| rs670318 | ALM | Fasting insulin | 0.0413 | 0.0044 | 2.52E-21 | 0.0048 | 0.0054 | 0.8842 |
| rs67527161 | ALM | Fasting insulin | -0.0182 | 0.0023 | 5.79029E-15 | 0.0021 | 0.0026 | 0.630001 |
| rs6789000 | ALM | Fasting insulin | 0.0121 | 0.002 | 1.61102E-09 | -0.0072 | 0.0086 | 0.424 |
| rs68049170 | ALM | Fasting insulin | -0.0259 | 0.0021 | 2.6903E-34 | -0.0005 | 0.0022 | 0.521601 |
| rs680882 | ALM | Fasting insulin | 0.0133 | 0.0022 | 1.98399E-09 | -0.0006 | 0.0023 | 0.5945 |
| rs6821305 | ALM | Fasting insulin | 0.0204 | 0.0019 | 3.1463E-26 | -0.0033 | 0.002 | 0.1603 |
| rs684905 | ALM | Fasting insulin | -0.0118 | 0.0019 | 7.10003E-10 | -0.0008 | 0.0021 | 0.778901 |
| rs6902109 | ALM | Fasting insulin | -0.0167 | 0.0019 | 1.03395E-18 | 0.0023 | 0.0021 | 0.2171 |
| rs6931421 | ALM | Fasting insulin | -0.0279 | 0.002 | 2.30515E-43 | -0.0014 | 0.0022 | 0.4047 |
| rs700677 | ALM | Fasting insulin | 0.0173 | 0.002 | 1.13006E-18 | 0.0002 | 0.0022 | 0.913 |
| rs7007389 | ALM | Fasting insulin | -0.0134 | 0.002 | 2.94374E-11 | -0.0071 | 0.0085 | 0.3686 |
| rs7014590 | ALM | Fasting insulin | -0.0228 | 0.0022 | 4.47713E-26 | -0.0018 | 0.0023 | 0.4902 |
| rs7020491 | ALM | Fasting insulin | -0.0178 | 0.0019 | 1.21899E-20 | -0.0024 | 0.0021 | 0.3606 |
| rs702886 | ALM | Fasting insulin | 0.012 | 0.002 | 1.11199E-09 | -0.0033 | 0.0021 | 0.0865008 |
| rs704660 | ALM | Fasting insulin | 0.0153 | 0.0019 | 2.27719E-15 | 0.0027 | 0.0021 | 0.1565 |
| rs7082659 | ALM | Fasting insulin | 0.0156 | 0.0028 | 2.273E-08 | 0.0014 | 0.0032 | 0.913 |
| rs7095472 | ALM | Fasting insulin | 0.0267 | 0.0019 | 7.65597E-45 | -0.0061 | 0.0022 | 0.00771205 |
| rs713467 | ALM | Fasting insulin | 0.0146 | 0.0019 | 3.09386E-14 | -0.0028 | 0.0019 | 0.2464 |
| rs7137546 | ALM | Fasting insulin | 0.0142 | 0.0019 | 9.70733E-14 | -0.0023 | 0.0021 | 0.3487 |
| rs71414738 | ALM | Fasting insulin | 0.015 | 0.0025 | 1.00201E-09 | -0.0009 | 0.003 | 0.9204 |
| rs71635721 | ALM | Fasting insulin | 0.0316 | 0.0039 | 3.46418E-16 | 0.002 | 0.0172 | 0.936 |
| rs7185244 | ALM | Fasting insulin | -0.0148 | 0.0023 | 1.39601E-10 | -0.0039 | 0.0027 | 0.221 |
| rs718603 | ALM | Fasting insulin | 0.0131 | 0.0021 | 6.67898E-10 | -0.0012 | 0.0023 | 0.6848 |
| rs7220127 | ALM | Fasting insulin | -0.0105 | 0.0019 | 4.60299E-08 | 0.0005 | 0.002 | 0.9813 |
| rs7228151 | ALM | Fasting insulin | -0.0185 | 0.0023 | 3.18933E-15 | 0.0027 | 0.0025 | 0.4879 |
| rs7229520 | ALM | Fasting insulin | -0.0224 | 0.002 | 9.20874E-29 | 0.0096 | 0.0021 | 7.01504E-05 |
| rs72657800 | ALM | Fasting insulin | -0.0219 | 0.0035 | 6.39102E-10 | -0.0061 | 0.004 | 0.1455 |
| rs72695791 | ALM | Fasting insulin | -0.0297 | 0.0051 | 4.57604E-09 | 0.0017 | 0.0059 | 0.745399 |
| rs72771070 | ALM | Fasting insulin | 0.015 | 0.0021 | 1.29688E-12 | -0.0024 | 0.0021 | 0.1374 |
| rs72829852 | ALM | Fasting insulin | 0.0309 | 0.0039 | 3.74024E-15 | -0.0017 | 0.0044 | 0.7545 |
| rs73006226 | ALM | Fasting insulin | -0.0182 | 0.0029 | 2.22998E-10 | 0.0124 | 0.0032 | 0.0001228 |
| rs7301341 | ALM | Fasting insulin | -0.0255 | 0.002 | 9.2747E-37 | -0.0019 | 0.0022 | 0.5317 |
| rs73052033 | ALM | Fasting insulin | -0.0151 | 0.0024 | 4.79005E-10 | 0.0067 | 0.0025 | 0.00629303 |
| rs73197345 | ALM | Fasting insulin | 0.0211 | 0.0028 | 3.54568E-14 | 0.0054 | 0.0029 | 0.03252 |
| rs7320878 | ALM | Fasting insulin | -0.015 | 0.0019 | 1.344E-14 | 0.0019 | 0.002 | 0.2714 |
| rs7321635 | ALM | Fasting insulin | -0.0132 | 0.002 | 2.5781E-11 | 0.001 | 0.0021 | 0.5028 |
| rs7328187 | ALM | Fasting insulin | 0.0116 | 0.0019 | 1.185E-09 | -0.0075 | 0.002 | 0.000295202 |
| rs73413540 | ALM | Fasting insulin | -0.0124 | 0.0023 | 4.42099E-08 | 0.0066 | 0.0028 | 0.00707001 |
| rs73856768 | ALM | Fasting insulin | -0.0247 | 0.0035 | 1.55418E-12 | -0.0045 | 0.0037 | 0.346 |
| rs74379684 | ALM | Fasting insulin | -0.0272 | 0.0036 | 4.39137E-14 | -0.0045 | 0.0037 | 0.1238 |
| rs7448554 | ALM | Fasting insulin | -0.0132 | 0.002 | 1.77296E-11 | 0.0044 | 0.0026 | 0.1774 |
| rs74494415 | ALM | Fasting insulin | -0.0417 | 0.0049 | 1.8218E-17 | -0.0074 | 0.0048 | 0.1418 |
| rs75022676 | ALM | Fasting insulin | -0.0163 | 0.0023 | 2.84315E-12 | 0.0002 | 0.0026 | 0.9693 |
| rs7522400 | ALM | Fasting insulin | 0.0129 | 0.0022 | 5.62497E-09 | 0.0017 | 0.0025 | 1 |
| rs7543136 | ALM | Fasting insulin | -0.021 | 0.0021 | 9.95864E-24 | -0.003 | 0.0022 | 0.4047 |
| rs7543202 | ALM | Fasting insulin | 0.0129 | 0.0019 | 2.86484E-11 | -0.0037 | 0.0022 | 0.0983603 |
| rs757834 | ALM | Fasting insulin | 0.0256 | 0.0024 | 1.24796E-25 | 0.0001 | 0.0026 | 0.8501 |
| rs7633464 | ALM | Fasting insulin | 0.0175 | 0.0019 | 1.27497E-20 | 0.0014 | 0.0018 | 0.4227 |
| rs7679276 | ALM | Fasting insulin | -0.033 | 0.0048 | 5.92652E-12 | 0.0013 | 0.0062 | 0.4685 |
| rs7701233 | ALM | Fasting insulin | -0.0179 | 0.0019 | 5.12035E-21 | 0.0028 | 0.002 | 0.6736 |
| rs77013652 | ALM | Fasting insulin | 0.049 | 0.0081 | 1.502E-09 | 0.0044 | 0.0081 | 0.4261 |
| rs7731023 | ALM | Fasting insulin | 0.0166 | 0.0019 | 3.48177E-18 | -0.0037 | 0.0019 | 0.0810905 |
| rs77364196 | ALM | Fasting insulin | -0.033 | 0.0043 | 8.34834E-15 | 0.0159 | 0.0058 | 0.00602296 |
| rs77447813 | ALM | Fasting insulin | 0.0224 | 0.0034 | 3.20775E-11 | -0.0117 | 0.0038 | 0.000324496 |
| rs7768382 | ALM | Fasting insulin | -0.0201 | 0.0019 | 1.57109E-26 | -0.0017 | 0.002 | 0.2807 |
| rs7816345 | ALM | Fasting insulin | 0.0255 | 0.0025 | 6.22444E-24 | 0.0091 | 0.0026 | 0.000329503 |
| rs7826059 | ALM | Fasting insulin | 0.0114 | 0.002 | 7.61395E-09 | -0.0019 | 0.0021 | 0.2726 |
| rs7828086 | ALM | Fasting insulin | 0.0135 | 0.0022 | 1.11099E-09 | -0.002 | 0.0023 | 0.4366 |
| rs78457529 | ALM | Fasting insulin | -0.0904 | 0.0088 | 1.22096E-24 | 0.0152 | 0.0111 | 0.24 |
| rs7858712 | ALM | Fasting insulin | 0.0347 | 0.0034 | 1.04304E-24 | -0.0086 | 0.0036 | 0.0118201 |
| rs7863102 | ALM | Fasting insulin | -0.011 | 0.0019 | 9.97401E-09 | -0.0009 | 0.0021 | 0.8909 |
| rs7893378 | ALM | Fasting insulin | 0.0175 | 0.0031 | 2.35999E-08 | 0.0057 | 0.0031 | 0.0840292 |
| rs7902 | ALM | Fasting insulin | 0.0149 | 0.0019 | 5.21915E-15 | -0.0046 | 0.0021 | 0.0493503 |
| rs79066296 | ALM | Fasting insulin | -0.0169 | 0.0022 | 6.10661E-14 | 0.0084 | 0.0094 | 0.3912 |
| rs7941305 | ALM | Fasting insulin | -0.0129 | 0.0021 | 6.01201E-10 | 0.011 | 0.009 | 0.232 |
| rs79441499 | ALM | Fasting insulin | -0.0138 | 0.0019 | 9.66941E-13 | -0.0024 | 0.0022 | 0.3706 |
| rs7971536 | ALM | Fasting insulin | -0.0194 | 0.0019 | 1.06488E-24 | 0.0014 | 0.002 | 0.7309 |
| rs80132799 | ALM | Fasting insulin | 0.0231 | 0.0038 | 1.31199E-09 | -0.0033 | 0.0047 | 0.6046 |
| rs8017006 | ALM | Fasting insulin | 0.0122 | 0.002 | 2.21998E-09 | 0.0006 | 0.0025 | 0.6883 |
| rs8018486 | ALM | Fasting insulin | -0.0138 | 0.0024 | 1.17801E-08 | -0.0022 | 0.0026 | 0.4497 |
| rs8019890 | ALM | Fasting insulin | 0.025 | 0.0019 | 1.95884E-38 | 0.0007 | 0.0025 | 0.637699 |
| rs80280630 | ALM | Fasting insulin | -0.0168 | 0.003 | 2.27898E-08 | 0.0037 | 0.0036 | 0.5093 |
| rs80295797 | ALM | Fasting insulin | -0.0198 | 0.002 | 3.8256E-23 | 0.0004 | 0.0022 | 0.9478 |
| rs8042578 | ALM | Fasting insulin | 0.0287 | 0.0022 | 2.28507E-38 | -0.0033 | 0.0022 | 0.1783 |
| rs8054549 | ALM | Fasting insulin | -0.0251 | 0.0019 | 3.37287E-39 | 0.0071 | 0.0021 | 0.00917403 |
| rs8084413 | ALM | Fasting insulin | -0.0127 | 0.0019 | 3.24713E-11 | 0.0007 | 0.0022 | 0.793299 |
| rs8112948 | ALM | Fasting insulin | -0.0297 | 0.0022 | 4.24131E-42 | 0.0049 | 0.0093 | 0.6414 |
| rs8136517 | ALM | Fasting insulin | 0.0267 | 0.0039 | 6.58264E-12 | 0.0023 | 0.0048 | 0.2241 |
| rs839255 | ALM | Fasting insulin | -0.0126 | 0.0021 | 8.38205E-10 | 0.0007 | 0.0023 | 0.9489 |
| rs861674 | ALM | Fasting insulin | 0.0128 | 0.0019 | 1.3649E-11 | 0.0023 | 0.002 | 0.3143 |
| rs867529 | ALM | Fasting insulin | 0.0184 | 0.0021 | 1.00092E-18 | -0.0021 | 0.0022 | 0.6383 |
| rs876122 | ALM | Fasting insulin | 0.0162 | 0.0029 | 2.186E-08 | 0.0006 | 0.0031 | 0.9102 |
| rs900399 | ALM | Fasting insulin | 0.0164 | 0.0019 | 1.35394E-17 | 0.007 | 0.0019 | 0.0002073 |
| rs905938 | ALM | Fasting insulin | 0.0394 | 0.0021 | 8.42558E-77 | -0.0016 | 0.0022 | 0.5078 |
| rs909220 | ALM | Fasting insulin | -0.015 | 0.0019 | 3.24489E-15 | -0.0021 | 0.002 | 0.1747 |
| rs9343327 | ALM | Fasting insulin | 0.014 | 0.0019 | 1.09094E-13 | 0.0022 | 0.002 | 0.2307 |
| rs9385002 | ALM | Fasting insulin | -0.0147 | 0.0022 | 3.26287E-11 | -0.0018 | 0.0023 | 0.3569 |
| rs947099 | ALM | Fasting insulin | 0.0117 | 0.002 | 2.70701E-09 | 0.0016 | 0.0021 | 0.396 |
| rs951366 | ALM | Fasting insulin | 0.0205 | 0.0019 | 9.15377E-27 | -0.0009 | 0.0021 | 0.9272 |
| rs9517483 | ALM | Fasting insulin | -0.0181 | 0.0021 | 2.25996E-18 | -0.0009 | 0.0022 | 0.5265 |
| rs9590328 | ALM | Fasting insulin | 0.0153 | 0.0027 | 2.01999E-08 | -0.0017 | 0.003 | 0.5377 |
| rs9594714 | ALM | Fasting insulin | 0.0144 | 0.0021 | 2.64789E-12 | 0.0036 | 0.0022 | 0.0468803 |
| rs9610447 | ALM | Fasting insulin | 0.0152 | 0.0022 | 5.28567E-12 | -0.0022 | 0.0025 | 0.1086 |
| rs963317 | ALM | Fasting insulin | -0.0136 | 0.002 | 1.0311E-11 | 0.0011 | 0.002 | 0.6583 |
| rs9636364 | ALM | Fasting insulin | 0.011 | 0.0019 | 5.09296E-09 | -0.0031 | 0.002 | 0.1674 |
| rs9640283 | ALM | Fasting insulin | -0.0119 | 0.0019 | 4.53398E-10 | -0.0028 | 0.0021 | 0.1703 |
| rs9828525 | ALM | Fasting insulin | 0.0121 | 0.0019 | 2.50801E-10 | -0.0028 | 0.0021 | 0.2726 |
| rs9832919 | ALM | Fasting insulin | -0.0179 | 0.002 | 7.89587E-20 | -0.01 | 0.0083 | 0.1843 |
| rs9838614 | ALM | Fasting insulin | -0.0185 | 0.0019 | 1.21088E-21 | 0.0001 | 0.0019 | 0.9799 |
| rs985136 | ALM | Fasting insulin | 0.0138 | 0.002 | 2.9621E-12 | 0.0004 | 0.0026 | 0.4758 |
| rs9890062 | ALM | Fasting insulin | 0.0267 | 0.0039 | 1.20005E-11 | 0.0049 | 0.0045 | 0.3204 |
| rs990315 | ALM | Fasting insulin | -0.0115 | 0.002 | 5.07002E-09 | -0.0001 | 0.0021 | 0.697599 |
| rs9957318 | ALM | Fasting insulin | 0.0187 | 0.002 | 1.02E-20 | -0.0026 | 0.002 | 0.2307 |
| rs10005035 | ALM | HbA1C | -0.0175 | 12865684 | 0.0021 | 0.0016 | 0.0038 | 0.668299 |
| rs10019221 | ALM | HbA1C | -0.0124 | 21785364 | 0.0019 | 0.0048 | 0.0035 | 0.1676 |
| rs10036789 | ALM | HbA1C | 0.0163 | 71695918 | 0.0019 | 0.001 | 0.0034 | 0.7689 |
| rs1005723 | ALM | HbA1C | 0.0161 | 243646251 | 0.0024 | -0.01 | 0.0044 | 0.0227997 |
| rs10068640 | ALM | HbA1C | 0.0112 | 123981977 | 0.002 | -0.0053 | 0.006 | 0.3793 |
| rs10075249 | ALM | HbA1C | 0.0143 | 52846505 | 0.0019 | 0.0037 | 0.0034 | 0.2721 |
| rs10107388 | ALM | HbA1C | -0.0159 | 145004944 | 0.002 | -0.0014 | 0.0036 | 0.6917 |
| rs10112506 | ALM | HbA1C | -0.012 | 13164746 | 0.0019 | -0.018 | 0.0078 | 0.02073 |
| rs10123619 | ALM | HbA1C | -0.0171 | 119353611 | 0.0026 | 0.0046 | 0.0045 | 0.3052 |
| rs10128333 | ALM | HbA1C | -0.0146 | 64570038 | 0.0025 | -0.0078 | 0.0104 | 0.4545 |
| rs10171272 | ALM | HbA1C | 0.0136 | 25946636 | 0.002 | -0.0014 | 0.0038 | 0.721 |
| rs10202701 | ALM | HbA1C | 0.0227 | 232328681 | 0.0019 | -0.004 | 0.0034 | 0.2438 |
| rs10202845 | ALM | HbA1C | -0.0288 | 42575820 | 0.003 | 0.0118 | 0.005 | 0.01736 |
| rs10203320 | ALM | HbA1C | 0.0138 | 9771620 | 0.002 | 0.0017 | 0.0036 | 0.632799 |
| rs10203386 | ALM | HbA1C | -0.0238 | 25136866 | 0.0019 | 0.0023 | 0.0034 | 0.4979 |
| rs10205141 | ALM | HbA1C | 0.0241 | 11313340 | 0.0044 | -0.0113 | 0.0146 | 0.4393 |
| rs10221831 | ALM | HbA1C | 0.03 | 202107829 | 0.0053 | 0.0072 | 0.0085 | 0.4004 |
| rs10225945 | ALM | HbA1C | -0.0146 | 28250083 | 0.0026 | 0.0008 | 0.0049 | 0.8733 |
| rs10242866 | ALM | HbA1C | 0.0157 | 17920613 | 0.0019 | -0.0122 | 0.0035 | 0.000468598 |
| rs10283100 | ALM | HbA1C | 0.0575 | 120596023 | 0.0041 | 0.0085 | 0.0106 | 0.4226 |
| rs1035583 | ALM | HbA1C | 0.0148 | 207326937 | 0.0019 | -0.0042 | 0.0036 | 0.2476 |
| rs10471339 | ALM | HbA1C | -0.011 | 67823773 | 0.0019 | -0.0017 | 0.0036 | 0.635501 |
| rs10483727 | ALM | HbA1C | -0.0368 | 61072875 | 0.0019 | 0.0079 | 0.0036 | 0.0268102 |
| rs1056747 | ALM | HbA1C | -0.0155 | 35690102 | 0.0019 | 0.0012 | 0.0035 | 0.723299 |
| rs1063582 | ALM | HbA1C | -0.0185 | 23167353 | 0.0022 | -0.0024 | 0.004 | 0.5467 |
| rs10657263 | ALM | HbA1C | -0.013 | 49690460 | 0.0019 | 0.0044 | 0.0034 | 0.1899 |
| rs10748128 | ALM | HbA1C | 0.0255 | 69827658 | 0.002 | -0.0016 | 0.004 | 0.6889 |
| rs10776560 | ALM | HbA1C | -0.0157 | 50542358 | 0.0019 | 0.0023 | 0.0035 | 0.501601 |
| rs10796828 | ALM | HbA1C | 0.0154 | 69490346 | 0.002 | 0.0068 | 0.0035 | 0.0559101 |
| rs10807137 | ALM | HbA1C | -0.0455 | 34183026 | 0.0025 | 0.0069 | 0.0053 | 0.1893 |
| rs10815274 | ALM | HbA1C | 0.0124 | 5728968 | 0.0019 | 0.0015 | 0.0034 | 0.6691 |
| rs10822117 | ALM | HbA1C | -0.0176 | 52786701 | 0.0022 | -0.0051 | 0.0041 | 0.2088 |
| rs10829226 | ALM | HbA1C | -0.0112 | 27573952 | 0.002 | -0.0013 | 0.0035 | 0.719201 |
| rs10832963 | ALM | HbA1C | -0.0203 | 18664241 | 0.0022 | -0.0006 | 0.0039 | 0.8778 |
| rs10845408 | ALM | HbA1C | 0.0255 | 11880581 | 0.002 | 0.0044 | 0.0036 | 0.2204 |
| rs10858246 | ALM | HbA1C | -0.0188 | 139102831 | 0.002 | 0.0026 | 0.0052 | 0.614899 |
| rs10922475 | ALM | HbA1C | 0.0159 | 89142142 | 0.0019 | -0.0019 | 0.0034 | 0.5833 |
| rs10948 | ALM | HbA1C | -0.0252 | 10754905 | 0.002 | 0.0017 | 0.0036 | 0.637901 |
| rs10962212 | ALM | HbA1C | 0.0143 | 15911745 | 0.0019 | -0.0064 | 0.0035 | 0.0699198 |
| rs11009928 | ALM | HbA1C | -0.0148 | 35058712 | 0.0022 | 0.0008 | 0.0039 | 0.83 |
| rs11014285 | ALM | HbA1C | 0.0342 | 25178864 | 0.0026 | -0.0069 | 0.0062 | 0.2655 |
| rs11042717 | ALM | HbA1C | -0.029 | 10303939 | 0.0019 | 0.0049 | 0.0034 | 0.1489 |
| rs11049704 | ALM | HbA1C | -0.0183 | 28691701 | 0.0021 | -0.0005 | 0.0037 | 0.8824 |
| rs11060942 | ALM | HbA1C | -0.0354 | 123434524 | 0.0052 | -0.0086 | 0.0091 | 0.3443 |
| rs11068230 | ALM | HbA1C | 0.0238 | 117349014 | 0.0028 | -0.006 | 0.0079 | 0.4449 |
| rs11098677 | ALM | HbA1C | -0.0263 | 123833516 | 0.0023 | 0.0029 | 0.0061 | 0.6387 |
| rs111365325 | ALM | HbA1C | -0.0271 | 170865229 | 0.0022 | -0.0067 | 0.0095 | 0.4812 |
| rs111622870 | ALM | HbA1C | -0.0282 | 2613109 | 0.0044 | -0.0159 | 0.0091 | 0.0812905 |
| rs11175919 | ALM | HbA1C | 0.0349 | 66180277 | 0.0059 | -0.0023 | 0.0128 | 0.8583 |
| rs111901094 | ALM | HbA1C | -0.0253 | 19513570 | 0.0025 | 0.0044 | 0.0054 | 0.4124 |
| rs11198591 | ALM | HbA1C | 0.0148 | 120515892 | 0.002 | -0.0001 | 0.0041 | 0.9781 |
| rs11210892 | ALM | HbA1C | 0.0118 | 44100084 | 0.002 | -0.0041 | 0.0036 | 0.2639 |
| rs11233117 | ALM | HbA1C | -0.0176 | 69924352 | 0.0019 | 0.002 | 0.0036 | 0.565599 |
| rs11243202 | ALM | HbA1C | 0.0302 | 7719065 | 0.0019 | -0.0007 | 0.0034 | 0.845 |
| rs112537273 | ALM | HbA1C | -0.0212 | 38248306 | 0.0022 | 0.0017 | 0.004 | 0.6773 |
| rs11260035 | ALM | HbA1C | 0.015 | 7898957 | 0.0021 | 0.0037 | 0.0066 | 0.5706 |
| rs11260623 | ALM | HbA1C | 0.0117 | 1781456 | 0.0019 | -0.0009 | 0.0034 | 0.785599 |
| rs113232639 | ALM | HbA1C | 0.0327 | 20715656 | 0.0019 | 0.0051 | 0.0039 | 0.1881 |
| rs113671109 | ALM | HbA1C | -0.015 | 12620885 | 0.0023 | -0.0027 | 0.0044 | 0.537499 |
| rs113898003 | ALM | HbA1C | -0.036 | 130341235 | 0.0021 | -0.0019 | 0.0038 | 0.6155 |
| rs115010283 | ALM | HbA1C | 0.034 | 172162393 | 0.002 | 0.0092 | 0.0037 | 0.0120701 |
| rs115105539 | ALM | HbA1C | 0.0231 | 49409496 | 0.0025 | 0.0095 | 0.0045 | 0.0354201 |
| rs11590254 | ALM | HbA1C | 0.0186 | 92316573 | 0.002 | 0.0019 | 0.0036 | 0.593 |
| rs116052377 | ALM | HbA1C | 0.0225 | 124787756 | 0.0035 | -0.0069 | 0.0064 | 0.2809 |
| rs11605297 | ALM | HbA1C | 0.0146 | 58296806 | 0.0022 | 0.0006 | 0.004 | 0.8885 |
| rs11633371 | ALM | HbA1C | 0.0216 | 89356832 | 0.0019 | 0.0007 | 0.0034 | 0.8367 |
| rs116339650 | ALM | HbA1C | -0.0175 | 26200972 | 0.0029 | 0.0035 | 0.0048 | 0.4689 |
| rs11672848 | ALM | HbA1C | -0.0171 | 37570704 | 0.0019 | -0.0022 | 0.0034 | 0.5125 |
| rs1168768 | ALM | HbA1C | 0.0332 | 66509650 | 0.006 | -0.0294 | 0.0179 | 0.1004 |
| rs11727162 | ALM | HbA1C | -0.017 | 88606761 | 0.0019 | 0.0027 | 0.0034 | 0.4315 |
| rs11778491 | ALM | HbA1C | -0.0247 | 120451362 | 0.0022 | 0.0012 | 0.004 | 0.7662 |
| rs1190540 | ALM | HbA1C | 0.0125 | 102897009 | 0.0021 | 0.0032 | 0.0037 | 0.3849 |
| rs11959466 | ALM | HbA1C | 0.038 | 42803824 | 0.0042 | 0.0009 | 0.0089 | 0.9197 |
| rs1202186 | ALM | HbA1C | -0.012 | 87213258 | 0.002 | 0.0009 | 0.0036 | 0.8104 |
| rs12051245 | ALM | HbA1C | 0.0299 | 783865 | 0.0022 | -0.0057 | 0.0047 | 0.2283 |
| rs12074850 | ALM | HbA1C | 0.0393 | 51248316 | 0.0033 | -0.0215 | 0.0059 | 0.000274397 |
| rs12099669 | ALM | HbA1C | 0.0331 | 46783653 | 0.002 | 0.0006 | 0.0036 | 0.8594 |
| rs12188208 | ALM | HbA1C | -0.0195 | 77442791 | 0.0022 | -0.0087 | 0.0041 | 0.0327597 |
| rs12230946 | ALM | HbA1C | 0.0271 | 53498725 | 0.0033 | 0.0066 | 0.0057 | 0.2488 |
| rs12334478 | ALM | HbA1C | -0.0161 | 141998765 | 0.0019 | 0.0009 | 0.0034 | 0.7863 |
| rs12340775 | ALM | HbA1C | -0.0287 | 13226945 | 0.0043 | -0.018 | 0.0072 | 0.01274 |
| rs12344515 | ALM | HbA1C | -0.0163 | 113801231 | 0.0022 | -0.0001 | 0.0041 | 0.9744 |
| rs12347137 | ALM | HbA1C | -0.046 | 119122721 | 0.0024 | -0.0072 | 0.0043 | 0.0922295 |
| rs12351226 | ALM | HbA1C | 0.0218 | 98405230 | 0.0025 | 0.0075 | 0.0051 | 0.1386 |
| rs12461874 | ALM | HbA1C | -0.0181 | 17180358 | 0.0021 | -0.0036 | 0.0039 | 0.3509 |
| rs12483401 | ALM | HbA1C | -0.0387 | 35443829 | 0.0067 | 0.0059 | 0.0125 | 0.6389 |
| rs12512942 | ALM | HbA1C | -0.0162 | 177766307 | 0.002 | -0.0058 | 0.0038 | 0.1304 |
| rs12517711 | ALM | HbA1C | -0.0147 | 60754661 | 0.0019 | 0.0043 | 0.0035 | 0.2145 |
| rs12519407 | ALM | HbA1C | 0.0181 | 137651012 | 0.0022 | 0.0029 | 0.0038 | 0.4503 |
| rs12533452 | ALM | HbA1C | 0.0237 | 19016871 | 0.0026 | 0.0037 | 0.0047 | 0.4295 |
| rs12541381 | ALM | HbA1C | -0.0319 | 135649848 | 0.0022 | 0.0014 | 0.0045 | 0.7556 |
| rs12563442 | ALM | HbA1C | 0.0122 | 19786695 | 0.0021 | 0.0024 | 0.0039 | 0.5462 |
| rs1260326 | ALM | HbA1C | 0.0323 | 27730940 | 0.0019 | 0.0035 | 0.0035 | 0.3069 |
| rs12616192 | ALM | HbA1C | -0.0261 | 121568931 | 0.0038 | 0.0091 | 0.011 | 0.4104 |
| rs12655296 | ALM | HbA1C | -0.011 | 15890643 | 0.002 | -0.0038 | 0.0035 | 0.2886 |
| rs12672217 | ALM | HbA1C | 0.0139 | 156310948 | 0.002 | -0.006 | 0.0036 | 0.0949489 |
| rs12702693 | ALM | HbA1C | 0.0173 | 8101039 | 0.0019 | 0.0018 | 0.0034 | 0.5966 |
| rs12724708 | ALM | HbA1C | 0.0243 | 219620569 | 0.002 | -0.0062 | 0.0038 | 0.0985689 |
| rs12773500 | ALM | HbA1C | 0.0171 | 81232632 | 0.0028 | -0.0121 | 0.0047 | 0.00971695 |
| rs12831751 | ALM | HbA1C | 0.0172 | 29520017 | 0.0021 | -0.0035 | 0.0061 | 0.5654 |
| rs12882130 | ALM | HbA1C | -0.0202 | 103878774 | 0.002 | -0.0021 | 0.0036 | 0.5651 |
| rs12907139 | ALM | HbA1C | -0.0149 | 73521566 | 0.0019 | 0.0034 | 0.0034 | 0.3169 |
| rs1290786 | ALM | HbA1C | -0.0143 | 169097381 | 0.0019 | 0.0047 | 0.0034 | 0.1673 |
| rs12909863 | ALM | HbA1C | 0.0189 | 75825822 | 0.0022 | -0.0005 | 0.0039 | 0.9021 |
| rs1291114 | ALM | HbA1C | 0.0173 | 35500850 | 0.0031 | 0.011 | 0.0058 | 0.0605494 |
| rs12926103 | ALM | HbA1C | 0.0272 | 86371775 | 0.0038 | 0.0014 | 0.007 | 0.8396 |
| rs12962050 | ALM | HbA1C | 0.0153 | 35179808 | 0.002 | -0.0026 | 0.0036 | 0.4723 |
| rs12997625 | ALM | HbA1C | -0.017 | 202970250 | 0.0019 | -0.0029 | 0.0034 | 0.4033 |
| rs13037813 | ALM | HbA1C | 0.0292 | 47750588 | 0.0022 | 0.0005 | 0.0042 | 0.9086 |
| rs13103161 | ALM | HbA1C | -0.0284 | 106216459 | 0.0019 | -0.0018 | 0.0036 | 0.611499 |
| rs13123591 | ALM | HbA1C | 0.0185 | 120105990 | 0.002 | -0.0009 | 0.0036 | 0.8086 |
| rs13170063 | ALM | HbA1C | -0.0152 | 157895013 | 0.0019 | 0.0064 | 0.0035 | 0.0681695 |
| rs13209685 | ALM | HbA1C | 0.0277 | 7779729 | 0.0026 | -0.0026 | 0.0046 | 0.575999 |
| rs1324538 | ALM | HbA1C | 0.0237 | 45080144 | 0.0019 | 0.0034 | 0.0039 | 0.3801 |
| rs1325596 | ALM | HbA1C | 0.0287 | 176794066 | 0.0019 | -0.0047 | 0.0034 | 0.1654 |
| rs1330826 | ALM | HbA1C | 0.0162 | 85129970 | 0.0023 | 0.0024 | 0.0045 | 0.5882 |
| rs13391980 | ALM | HbA1C | -0.0225 | 165504841 | 0.0029 | -0.0078 | 0.0087 | 0.3678 |
| rs1340022 | ALM | HbA1C | 0.0118 | 131334465 | 0.0019 | -0.0032 | 0.0034 | 0.3522 |
| rs139163241 | ALM | HbA1C | -0.0164 | 76709213 | 0.0027 | -0.0063 | 0.0056 | 0.263 |
| rs1405227 | ALM | HbA1C | 0.0129 | 98873390 | 0.002 | -0.0002 | 0.0037 | 0.9495 |
| rs143384 | ALM | HbA1C | 0.0725 | 34025756 | 0.0019 | -0.0066 | 0.0037 | 0.0755005 |
| rs1443536 | ALM | HbA1C | 0.0218 | 82174165 | 0.0021 | 0.0067 | 0.008 | 0.4046 |
| rs1444628 | ALM | HbA1C | 0.024 | 20563643 | 0.002 | 0.0084 | 0.0086 | 0.3339 |
| rs1472852 | ALM | HbA1C | -0.0638 | 17910236 | 0.0026 | -0.0023 | 0.0047 | 0.6239 |
| rs1478575 | ALM | HbA1C | 0.0312 | 218278555 | 0.002 | 0.001 | 0.0037 | 0.780301 |
| rs1514134 | ALM | HbA1C | -0.0114 | 56116513 | 0.0019 | 0.0019 | 0.0035 | 0.5799 |
| rs1556659 | ALM | HbA1C | 0.0163 | 130834698 | 0.002 | -0.003 | 0.0037 | 0.416 |
| rs1584011 | ALM | HbA1C | 0.0159 | 27080527 | 0.002 | 0.0019 | 0.0035 | 0.592199 |
| rs165849 | ALM | HbA1C | 0.0157 | 19958669 | 0.0021 | 0.004 | 0.0038 | 0.2983 |
| rs17197114 | ALM | HbA1C | 0.0177 | 21894526 | 0.0025 | 0.0019 | 0.0053 | 0.7124 |
| rs17205463 | ALM | HbA1C | -0.0263 | 62381413 | 0.0019 | 0.0027 | 0.0034 | 0.4316 |
| rs17246129 | ALM | HbA1C | 0.0254 | 227259964 | 0.002 | -0.002 | 0.0036 | 0.5872 |
| rs1730028 | ALM | HbA1C | 0.0131 | 157900789 | 0.0019 | 0.0021 | 0.0034 | 0.5398 |
| rs173135 | ALM | HbA1C | -0.0341 | 68172326 | 0.003 | -0.0063 | 0.0055 | 0.248 |
| rs17400325 | ALM | HbA1C | 0.0345 | 178565913 | 0.0047 | -0.0007 | 0.0077 | 0.9272 |
| rs17478946 | ALM | HbA1C | -0.0192 | 24093062 | 0.0021 | 0.0018 | 0.0041 | 0.6659 |
| rs17681189 | ALM | HbA1C | -0.0131 | 65976175 | 0.0019 | -0.0075 | 0.0034 | 0.0290797 |
| rs17718736 | ALM | HbA1C | 0.0115 | 71555205 | 0.002 | -0.0035 | 0.0036 | 0.3378 |
| rs17773965 | ALM | HbA1C | -0.0163 | 217631338 | 0.0027 | 0.0003 | 0.0048 | 0.9504 |
| rs17818592 | ALM | HbA1C | -0.0129 | 86088594 | 0.0019 | -0.0009 | 0.0034 | 0.8011 |
| rs1786263 | ALM | HbA1C | -0.019 | 13116432 | 0.0019 | -0.0019 | 0.0035 | 0.5843 |
| rs1797070 | ALM | HbA1C | 0.0219 | 218630201 | 0.0021 | 0.0035 | 0.0039 | 0.3742 |
| rs1823217 | ALM | HbA1C | -0.0181 | 134380959 | 0.002 | -0.0033 | 0.0038 | 0.3914 |
| rs188617336 | ALM | HbA1C | 0.0138 | 20610730 | 0.0021 | -0.0084 | 0.0037 | 0.0209802 |
| rs1899040 | ALM | HbA1C | 0.0152 | 223901896 | 0.0023 | 0.0021 | 0.0043 | 0.621199 |
| rs1933081 | ALM | HbA1C | 0.0267 | 151651505 | 0.0034 | 0.0018 | 0.0063 | 0.778501 |
| rs199647708 | ALM | HbA1C | 0.0114 | 125352195 | 0.0019 | 0.0014 | 0.0035 | 0.6928 |
| rs200439 | ALM | HbA1C | -0.0128 | 6716083 | 0.0023 | -0.0017 | 0.0042 | 0.6909 |
| rs2005172 | ALM | HbA1C | 0.048 | 61996255 | 0.002 | 0.0105 | 0.0061 | 0.0818597 |
| rs2019203 | ALM | HbA1C | 0.0189 | 36908672 | 0.0019 | -0.0024 | 0.0034 | 0.4766 |
| rs2025609 | ALM | HbA1C | 0.0186 | 67422990 | 0.0026 | 0.0071 | 0.0047 | 0.1344 |
| rs2025808 | ALM | HbA1C | 0.0122 | 184161757 | 0.0022 | -0.0073 | 0.0039 | 0.0608107 |
| rs2035901 | ALM | HbA1C | 0.024 | 145521867 | 0.0019 | -0.0023 | 0.0036 | 0.5254 |
| rs2070598 | ALM | HbA1C | 0.0204 | 75360906 | 0.0019 | 0.0069 | 0.0074 | 0.3498 |
| rs2101017 | ALM | HbA1C | -0.0223 | 122306857 | 0.0028 | -0.0015 | 0.0066 | 0.8143 |
| rs2112617 | ALM | HbA1C | -0.0167 | 46977125 | 0.0019 | 0.0052 | 0.0034 | 0.1223 |
| rs212526 | ALM | HbA1C | 0.0214 | 21584941 | 0.0019 | -0.0045 | 0.0035 | 0.2037 |
| rs2138374 | ALM | HbA1C | -0.0149 | 190014317 | 0.002 | -0.0013 | 0.0037 | 0.7196 |
| rs2140619 | ALM | HbA1C | 0.0113 | 114007270 | 0.0019 | -0.0009 | 0.0035 | 0.8082 |
| rs2142331 | ALM | HbA1C | -0.0165 | 116636719 | 0.0019 | -0.0001 | 0.0035 | 0.9794 |
| rs2174008 | ALM | HbA1C | -0.0192 | 38510456 | 0.0019 | -0.0013 | 0.0034 | 0.713801 |
| rs2181834 | ALM | HbA1C | 0.0254 | 102661251 | 0.0019 | -0.0006 | 0.0034 | 0.8702 |
| rs2188805 | ALM | HbA1C | 0.0114 | 93078400 | 0.002 | -0.0057 | 0.0042 | 0.1714 |
| rs2209098 | ALM | HbA1C | 0.024 | 172167226 | 0.002 | -0.0108 | 0.0037 | 0.003697 |
| rs2212926 | ALM | HbA1C | -0.022 | 38066883 | 0.0023 | -0.0021 | 0.0052 | 0.6815 |
| rs2229840 | ALM | HbA1C | 0.0341 | 124826462 | 0.0026 | -0.0063 | 0.0053 | 0.2368 |
| rs2230033 | ALM | HbA1C | -0.0265 | 39671476 | 0.0019 | -0.0021 | 0.0034 | 0.5394 |
| rs2236096 | ALM | HbA1C | 0.018 | 3266319 | 0.0023 | -0.0071 | 0.0042 | 0.0880197 |
| rs2237485 | ALM | HbA1C | 0.0191 | 50749870 | 0.0023 | -0.0005 | 0.004 | 0.8897 |
| rs2240735 | ALM | HbA1C | 0.0189 | 4027605 | 0.0022 | -0.0031 | 0.008 | 0.7008 |
| rs2268718 | ALM | HbA1C | 0.0141 | 52415023 | 0.0021 | -0.0033 | 0.0038 | 0.3869 |
| rs2270894 | ALM | HbA1C | -0.0332 | 9975386 | 0.0024 | -0.003 | 0.0062 | 0.630999 |
| rs2274351 | ALM | HbA1C | 0.017 | 104264107 | 0.0019 | -0.0031 | 0.0056 | 0.5837 |
| rs2283200 | ALM | HbA1C | -0.0281 | 2729340 | 0.0042 | -0.0019 | 0.0073 | 0.792301 |
| rs2287821 | ALM | HbA1C | -0.0153 | 33935102 | 0.0019 | -0.0076 | 0.0034 | 0.0254501 |
| rs2289629 | ALM | HbA1C | -0.0148 | 27959903 | 0.002 | 0.0012 | 0.0036 | 0.7287 |
| rs2296316 | ALM | HbA1C | -0.0192 | 65520246 | 0.0019 | 0.0024 | 0.0034 | 0.490601 |
| rs2303423 | ALM | HbA1C | 0.0168 | 38120029 | 0.003 | 0.0098 | 0.0054 | 0.0691799 |
| rs2305141 | ALM | HbA1C | 0.0183 | 233684402 | 0.0019 | -0.0026 | 0.0036 | 0.4682 |
| rs2347603 | ALM | HbA1C | -0.0181 | 47297426 | 0.0022 | 0.0039 | 0.0039 | 0.3253 |
| rs2347808 | ALM | HbA1C | -0.0125 | 2750856 | 0.0019 | 0.0028 | 0.0034 | 0.4035 |
| rs2390669 | ALM | HbA1C | 0.0174 | 169091942 | 0.0028 | -0.0009 | 0.0054 | 0.871 |
| rs2454390 | ALM | HbA1C | -0.0176 | 103255613 | 0.0026 | -0.0006 | 0.0047 | 0.8972 |
| rs246177 | ALM | HbA1C | 0.0214 | 14380768 | 0.002 | 0.0029 | 0.006 | 0.6308 |
| rs2490302 | ALM | HbA1C | 0.0221 | 37702435 | 0.0034 | 0.0035 | 0.0056 | 0.524901 |
| rs249677 | ALM | HbA1C | -0.0109 | 141539339 | 0.002 | 0.0014 | 0.0036 | 0.7002 |
| rs2521349 | ALM | HbA1C | 0.0155 | 67503501 | 0.0019 | 0.0036 | 0.0035 | 0.3004 |
| rs2529090 | ALM | HbA1C | 0.0136 | 24662280 | 0.0025 | 0.0002 | 0.0045 | 0.9573 |
| rs2545339 | ALM | HbA1C | 0.0115 | 149911219 | 0.002 | 0 | 0.0036 | 0.9971 |
| rs2578565 | ALM | HbA1C | -0.0141 | 5460569 | 0.002 | 0.0042 | 0.0036 | 0.2351 |
| rs258794 | ALM | HbA1C | 0.0147 | 142540040 | 0.0021 | 0.0025 | 0.0038 | 0.5054 |
| rs2592208 | ALM | HbA1C | -0.0124 | 67408873 | 0.0019 | -0.0065 | 0.0034 | 0.0599198 |
| rs261223 | ALM | HbA1C | 0.0175 | 95901046 | 0.0019 | -0.0052 | 0.0035 | 0.1413 |
| rs2648725 | ALM | HbA1C | 0.0165 | 93015079 | 0.0023 | 0.0015 | 0.0087 | 0.8609 |
| rs2663126 | ALM | HbA1C | -0.0139 | 99563857 | 0.0021 | -0.0002 | 0.0037 | 0.9547 |
| rs2717008 | ALM | HbA1C | -0.0127 | 58149158 | 0.0019 | -0.0008 | 0.0035 | 0.825 |
| rs2748501 | ALM | HbA1C | -0.0195 | 146312258 | 0.0019 | -0.0018 | 0.0034 | 0.604999 |
| rs2754255 | ALM | HbA1C | -0.0153 | 88393572 | 0.0023 | 0.0003 | 0.0042 | 0.9352 |
| rs2763263 | ALM | HbA1C | -0.017 | 168814392 | 0.0022 | -0.0049 | 0.004 | 0.2188 |
| rs2764264 | ALM | HbA1C | 0.0203 | 108934461 | 0.0021 | 0.006 | 0.0037 | 0.1029 |
| rs2788213 | ALM | HbA1C | 0.0123 | 703249 | 0.0021 | 0.0015 | 0.0038 | 0.6995 |
| rs2789365 | ALM | HbA1C | -0.0145 | 235515534 | 0.0019 | 0.0043 | 0.0034 | 0.2086 |
| rs2791654 | ALM | HbA1C | -0.0239 | 11129317 | 0.0022 | 0.0025 | 0.0038 | 0.5146 |
| rs28529055 | ALM | HbA1C | -0.0147 | 92428216 | 0.0019 | 0.0009 | 0.0034 | 0.8011 |
| rs28678024 | ALM | HbA1C | -0.0119 | 25937161 | 0.0021 | -0.0026 | 0.0038 | 0.4825 |
| rs2871865 | ALM | HbA1C | -0.0493 | 99194896 | 0.003 | -0.0061 | 0.0055 | 0.2707 |
| rs2871960 | ALM | HbA1C | 0.0469 | 141121814 | 0.0019 | -0.0016 | 0.0034 | 0.630999 |
| rs28736838 | ALM | HbA1C | -0.0117 | 120148713 | 0.002 | -0.0037 | 0.0036 | 0.3051 |
| rs2923411 | ALM | HbA1C | 0.0127 | 42455206 | 0.0019 | -0.0117 | 0.0035 | 0.000812494 |
| rs2925155 | ALM | HbA1C | -0.015 | 75886297 | 0.0022 | 0.0015 | 0.004 | 0.7141 |
| rs293517 | ALM | HbA1C | -0.013 | 83662455 | 0.0021 | -0.0019 | 0.0038 | 0.6042 |
| rs2971857 | ALM | HbA1C | -0.0119 | 234369487 | 0.0019 | 0.0031 | 0.0038 | 0.4108 |
| rs2978362 | ALM | HbA1C | 0.0106 | 32959397 | 0.0019 | -0.0009 | 0.0034 | 0.7871 |
| rs301807 | ALM | HbA1C | -0.0144 | 8484823 | 0.0019 | 0.0004 | 0.0034 | 0.8974 |
| rs3103223 | ALM | HbA1C | 0.0126 | 42402721 | 0.0022 | 0.0041 | 0.0039 | 0.2945 |
| rs310796 | ALM | HbA1C | 0.0142 | 77453226 | 0.002 | -0.002 | 0.0037 | 0.5963 |
| rs3116194 | ALM | HbA1C | -0.0295 | 233061266 | 0.0032 | -0.0104 | 0.0059 | 0.0799705 |
| rs3116602 | ALM | HbA1C | -0.0612 | 51111355 | 0.0023 | -0.0042 | 0.0041 | 0.3078 |
| rs3184504 | ALM | HbA1C | 0.0183 | 111884608 | 0.0019 | 0.0137 | 0.0035 | 9.95612E-05 |
| rs331917 | ALM | HbA1C | -0.0127 | 98158524 | 0.0019 | -0.0045 | 0.0035 | 0.1992 |
| rs336630 | ALM | HbA1C | -0.0106 | 18607538 | 0.0019 | -0.0034 | 0.0043 | 0.4207 |
| rs34338597 | ALM | HbA1C | -0.0112 | 106301580 | 0.0019 | 0.0057 | 0.0036 | 0.1084 |
| rs34390533 | ALM | HbA1C | -0.0257 | 184030838 | 0.0022 | 0.0027 | 0.0047 | 0.5636 |
| rs34776209 | ALM | HbA1C | -0.0317 | 23513093 | 0.0022 | -0.01 | 0.0039 | 0.0106299 |
| rs34879158 | ALM | HbA1C | -0.0363 | 32300634 | 0.0022 | -0.005 | 0.0041 | 0.2154 |
| rs35073631 | ALM | HbA1C | 0.0112 | 22696964 | 0.0019 | 0.0016 | 0.0034 | 0.6414 |
| rs350832 | ALM | HbA1C | -0.0165 | 4069426 | 0.0023 | -0.0034 | 0.0059 | 0.5623 |
| rs35288270 | ALM | HbA1C | -0.0328 | 4961278 | 0.0028 | 0.0007 | 0.0052 | 0.8869 |
| rs35732917 | ALM | HbA1C | 0.0204 | 73013269 | 0.0021 | 0.0046 | 0.0066 | 0.4869 |
| rs36048468 | ALM | HbA1C | 0.0254 | 122879901 | 0.0023 | -0.0064 | 0.004 | 0.1119 |
| rs3764002 | ALM | HbA1C | 0.028 | 108618630 | 0.0021 | -0.0123 | 0.0064 | 0.0532402 |
| rs3768495 | ALM | HbA1C | -0.0178 | 109935325 | 0.0021 | -0.0023 | 0.0037 | 0.5391 |
| rs3769598 | ALM | HbA1C | 0.0171 | 32679732 | 0.0027 | 0.0003 | 0.005 | 0.9554 |
| rs377599 | ALM | HbA1C | 0.0217 | 2164699 | 0.0019 | -0.0033 | 0.0036 | 0.3564 |
| rs3778858 | ALM | HbA1C | 0.0108 | 129963356 | 0.002 | 0.0026 | 0.0038 | 0.5006 |
| rs3818416 | ALM | HbA1C | 0.0279 | 78474468 | 0.0022 | 0.0068 | 0.004 | 0.0911297 |
| rs3822742 | ALM | HbA1C | 0.0162 | 139059017 | 0.002 | 0.0035 | 0.0036 | 0.3367 |
| rs3828729 | ALM | HbA1C | -0.016 | 155554707 | 0.002 | -0.0005 | 0.0061 | 0.9329 |
| rs3901421 | ALM | HbA1C | 0.0215 | 96204538 | 0.0019 | -0.0009 | 0.0034 | 0.8001 |
| rs395980 | ALM | HbA1C | -0.0184 | 177430072 | 0.0021 | 0.0082 | 0.0062 | 0.1901 |
| rs40270 | ALM | HbA1C | 0.0151 | 55804552 | 0.0022 | -0.0019 | 0.0039 | 0.6321 |
| rs4073154 | ALM | HbA1C | 0.0274 | 129035485 | 0.0023 | -0.0108 | 0.0042 | 0.00996805 |
| rs4076108 | ALM | HbA1C | 0.0174 | 13736088 | 0.0022 | 0.0028 | 0.004 | 0.4793 |
| rs4077103 | ALM | HbA1C | -0.0143 | 49557732 | 0.0026 | 0.0025 | 0.0047 | 0.5986 |
| rs42039 | ALM | HbA1C | 0.0481 | 92244422 | 0.0022 | -0.0069 | 0.0039 | 0.0804192 |
| rs4282339 | ALM | HbA1C | -0.0311 | 168256240 | 0.0023 | -0.0054 | 0.0043 | 0.2016 |
| rs4287835 | ALM | HbA1C | 0.0147 | 31457337 | 0.0019 | 0.0045 | 0.0034 | 0.1837 |
| rs4360494 | ALM | HbA1C | -0.0198 | 38455891 | 0.0019 | -0.0035 | 0.0036 | 0.3272 |
| rs4380799 | ALM | HbA1C | -0.0255 | 32571864 | 0.0021 | 0.0034 | 0.0059 | 0.5689 |
| rs4504126 | ALM | HbA1C | 0.046 | 33600582 | 0.0058 | -0.0216 | 0.0177 | 0.2228 |
| rs4602848 | ALM | HbA1C | 0.016 | 92186933 | 0.002 | -0.0019 | 0.0036 | 0.589299 |
| rs4622329 | ALM | HbA1C | 0.0149 | 102321935 | 0.002 | -0.0024 | 0.0037 | 0.521 |
| rs4640244 | ALM | HbA1C | -0.02 | 21284223 | 0.0019 | -0.0028 | 0.0042 | 0.503499 |
| rs4644481 | ALM | HbA1C | -0.0112 | 155130900 | 0.0019 | 0.0037 | 0.0034 | 0.2866 |
| rs4655345 | ALM | HbA1C | -0.0246 | 214608704 | 0.0019 | 0.0012 | 0.0035 | 0.7326 |
| rs4682483 | ALM | HbA1C | -0.0165 | 112993982 | 0.0026 | 0.0007 | 0.0044 | 0.8688 |
| rs4683435 | ALM | HbA1C | 0.0144 | 142624732 | 0.0022 | 0.0004 | 0.0044 | 0.9299 |
| rs4735761 | ALM | HbA1C | 0.0331 | 78097161 | 0.0021 | 0.0014 | 0.0037 | 0.7186 |
| rs4748008 | ALM | HbA1C | -0.0125 | 12935125 | 0.0019 | 0.0003 | 0.0035 | 0.9417 |
| rs4752689 | ALM | HbA1C | 0.0205 | 124131176 | 0.0019 | 0.0021 | 0.0035 | 0.543 |
| rs4752829 | ALM | HbA1C | 0.0262 | 47396654 | 0.0021 | 0.0016 | 0.0037 | 0.674899 |
| rs4788218 | ALM | HbA1C | 0.0275 | 30055750 | 0.0019 | -0.0023 | 0.0036 | 0.5286 |
| rs4807472 | ALM | HbA1C | -0.0158 | 3448842 | 0.002 | -0.0083 | 0.0037 | 0.0259597 |
| rs4815952 | ALM | HbA1C | -0.0161 | 6934897 | 0.0019 | 0.0004 | 0.0034 | 0.9125 |
| rs4818280 | ALM | HbA1C | -0.0124 | 18114472 | 0.002 | -0.0031 | 0.0035 | 0.3861 |
| rs4847378 | ALM | HbA1C | 0.0136 | 93324634 | 0.0019 | -0.0045 | 0.0035 | 0.2006 |
| rs4852257 | ALM | HbA1C | -0.0231 | 71678520 | 0.0019 | 0.0036 | 0.0036 | 0.309 |
| rs4865956 | ALM | HbA1C | -0.0258 | 54882505 | 0.0021 | -0.0084 | 0.0037 | 0.0244501 |
| rs4870941 | ALM | HbA1C | -0.0297 | 126498828 | 0.0023 | -0.0085 | 0.009 | 0.3438 |
| rs488621 | ALM | HbA1C | 0.0191 | 169707552 | 0.0019 | 0.0021 | 0.0034 | 0.5407 |
| rs4900578 | ALM | HbA1C | -0.0177 | 103926010 | 0.002 | -0.0029 | 0.0035 | 0.4185 |
| rs4932439 | ALM | HbA1C | -0.0151 | 89401109 | 0.0025 | 0.0059 | 0.0044 | 0.1799 |
| rs4938359 | ALM | HbA1C | -0.0156 | 117093560 | 0.0024 | 0.0012 | 0.0042 | 0.770801 |
| rs4940874 | ALM | HbA1C | 0.0148 | 57105638 | 0.0024 | 0.0074 | 0.0043 | 0.0867701 |
| rs4965298 | ALM | HbA1C | -0.0119 | 100802766 | 0.0021 | -0.008 | 0.0039 | 0.0395904 |
| rs496783 | ALM | HbA1C | -0.0124 | 116137961 | 0.0019 | 0.0063 | 0.0036 | 0.0819408 |
| rs4985445 | ALM | HbA1C | -0.0175 | 69867835 | 0.0019 | 0.0063 | 0.0034 | 0.0646994 |
| rs543650 | ALM | HbA1C | 0.025 | 152110943 | 0.002 | -0.0037 | 0.0043 | 0.3878 |
| rs55872725 | ALM | HbA1C | 0.0222 | 53809123 | 0.0019 | 0.0078 | 0.0034 | 0.0229298 |
| rs568267 | ALM | HbA1C | 0.0122 | 8799828 | 0.0022 | -0.0011 | 0.004 | 0.7795 |
| rs57059662 | ALM | HbA1C | 0.0118 | 33217275 | 0.002 | 0.001 | 0.0038 | 0.7978 |
| rs5742915 | ALM | HbA1C | 0.0248 | 74336633 | 0.0019 | -0.004 | 0.0039 | 0.3033 |
| rs5753518 | ALM | HbA1C | 0.0242 | 31631314 | 0.0033 | 0.0023 | 0.0066 | 0.726901 |
| rs5763821 | ALM | HbA1C | 0.0191 | 30549071 | 0.002 | -0.0044 | 0.0034 | 0.1957 |
| rs577289 | ALM | HbA1C | -0.0125 | 40208911 | 0.0021 | 0.0002 | 0.0039 | 0.9676 |
| rs57791149 | ALM | HbA1C | -0.0173 | 54222307 | 0.0019 | -0.0036 | 0.0034 | 0.2987 |
| rs591668 | ALM | HbA1C | -0.0174 | 27535931 | 0.0019 | 0.0076 | 0.0036 | 0.0335498 |
| rs599004 | ALM | HbA1C | -0.0157 | 140439740 | 0.0021 | -0.0006 | 0.0038 | 0.8709 |
| rs59985551 | ALM | HbA1C | -0.0313 | 56106928 | 0.0022 | -0.0011 | 0.0039 | 0.780601 |
| rs6028716 | ALM | HbA1C | -0.021 | 38547459 | 0.0022 | -0.0017 | 0.004 | 0.672201 |
| rs604723 | ALM | HbA1C | -0.0166 | 100610546 | 0.0021 | 0.0051 | 0.0037 | 0.1704 |
| rs6054390 | ALM | HbA1C | -0.0188 | 6592094 | 0.002 | -0.0058 | 0.0035 | 0.1007 |
| rs6066122 | ALM | HbA1C | 0.0127 | 45558573 | 0.0023 | -0.0018 | 0.004 | 0.656999 |
| rs60804050 | ALM | HbA1C | -0.0217 | 118870373 | 0.0021 | 0.0022 | 0.004 | 0.5904 |
| rs6082354 | ALM | HbA1C | -0.024 | 21217976 | 0.002 | 0.0009 | 0.0037 | 0.7961 |
| rs610694 | ALM | HbA1C | 0.0136 | 121304826 | 0.0019 | -0.0054 | 0.0034 | 0.1072 |
| rs61397287 | ALM | HbA1C | 0.0235 | 144223279 | 0.0036 | -0.0027 | 0.0065 | 0.673 |
| rs6142059 | ALM | HbA1C | 0.0116 | 32544327 | 0.0019 | 0.001 | 0.0035 | 0.7784 |
| rs61528919 | ALM | HbA1C | 0.014 | 1004909 | 0.002 | 0.0006 | 0.0035 | 0.872 |
| rs62033029 | ALM | HbA1C | -0.0141 | 50107273 | 0.0023 | -0.0039 | 0.0042 | 0.3456 |
| rs62070319 | ALM | HbA1C | -0.018 | 89573216 | 0.002 | 0.0015 | 0.0034 | 0.663001 |
| rs62515437 | ALM | HbA1C | 0.0369 | 57160328 | 0.0023 | 0.0041 | 0.0041 | 0.317 |
| rs6425817 | ALM | HbA1C | 0.0157 | 33873034 | 0.002 | 0.0071 | 0.0036 | 0.0459304 |
| rs6470771 | ALM | HbA1C | -0.0268 | 130743726 | 0.0025 | 0.0038 | 0.0045 | 0.3955 |
| rs6502935 | ALM | HbA1C | -0.0125 | 1650168 | 0.0022 | -0.0044 | 0.0049 | 0.3725 |
| rs650508 | ALM | HbA1C | -0.013 | 45880122 | 0.002 | -0.0031 | 0.0038 | 0.4125 |
| rs6543146 | ALM | HbA1C | 0.0154 | 103096695 | 0.0019 | 0.0059 | 0.0034 | 0.0882694 |
| rs6570509 | ALM | HbA1C | -0.0244 | 142716286 | 0.0021 | -0.0014 | 0.0038 | 0.720299 |
| rs6582398 | ALM | HbA1C | 0.014 | 42870444 | 0.002 | -0.0002 | 0.0036 | 0.9648 |
| rs6593210 | ALM | HbA1C | 0.0146 | 55254186 | 0.0024 | 0.0017 | 0.0043 | 0.6987 |
| rs664317 | ALM | HbA1C | -0.0177 | 89812230 | 0.0026 | 0.0083 | 0.0045 | 0.0644896 |
| rs6675858 | ALM | HbA1C | -0.0137 | 224559936 | 0.0023 | 0.005 | 0.004 | 0.2199 |
| rs6693481 | ALM | HbA1C | -0.0143 | 203766395 | 0.002 | -0.0055 | 0.0037 | 0.142 |
| rs670318 | ALM | HbA1C | 0.0413 | 63727542 | 0.0044 | 0.0037 | 0.0087 | 0.667999 |
| rs6721191 | ALM | HbA1C | -0.0144 | 10190115 | 0.0019 | -0.0023 | 0.0077 | 0.7659 |
| rs6738207 | ALM | HbA1C | 0.0127 | 105989716 | 0.0019 | -0.0007 | 0.0035 | 0.8357 |
| rs6739278 | ALM | HbA1C | -0.021 | 44401055 | 0.0024 | -0.0003 | 0.0068 | 0.9667 |
| rs67527161 | ALM | HbA1C | -0.0182 | 63781824 | 0.0023 | 0.0022 | 0.0044 | 0.6083 |
| rs6762851 | ALM | HbA1C | -0.0218 | 56686329 | 0.002 | -0.0038 | 0.0035 | 0.272 |
| rs67716382 | ALM | HbA1C | 0.0226 | 46890317 | 0.0023 | -0.001 | 0.0043 | 0.8089 |
| rs6789000 | ALM | HbA1C | 0.0121 | 25188002 | 0.002 | 0.002 | 0.0035 | 0.5642 |
| rs68049170 | ALM | HbA1C | -0.0259 | 72432047 | 0.0021 | 0 | 0.0086 | 0.9991 |
| rs680882 | ALM | HbA1C | 0.0133 | 18325278 | 0.0022 | -0.0063 | 0.0042 | 0.134 |
| rs684905 | ALM | HbA1C | -0.0118 | 10472790 | 0.0019 | 0.0049 | 0.0034 | 0.1499 |
| rs6849302 | ALM | HbA1C | 0.0155 | 156665074 | 0.0024 | -0.005 | 0.0043 | 0.2419 |
| rs6860245 | ALM | HbA1C | 0.0589 | 127367998 | 0.0022 | 0.0036 | 0.004 | 0.3638 |
| rs6902109 | ALM | HbA1C | -0.0167 | 130316559 | 0.0019 | 0.0024 | 0.0034 | 0.4862 |
| rs700677 | ALM | HbA1C | 0.0173 | 198702424 | 0.002 | 0.0049 | 0.0036 | 0.1779 |
| rs7014590 | ALM | HbA1C | -0.0228 | 89335647 | 0.0022 | -0.0044 | 0.0036 | 0.2272 |
| rs7020491 | ALM | HbA1C | -0.0178 | 128144477 | 0.0019 | -0.0011 | 0.0035 | 0.7545 |
| rs702886 | ALM | HbA1C | 0.012 | 65753310 | 0.002 | -0.0005 | 0.0035 | 0.8767 |
| rs704660 | ALM | HbA1C | 0.0153 | 30447998 | 0.0019 | -0.0036 | 0.0035 | 0.3037 |
| rs7082659 | ALM | HbA1C | 0.0156 | 12017584 | 0.0028 | -0.0048 | 0.005 | 0.3402 |
| rs7137546 | ALM | HbA1C | 0.0142 | 577237 | 0.0019 | -0.0003 | 0.0035 | 0.94 |
| rs71414738 | ALM | HbA1C | 0.015 | 127876242 | 0.0025 | -0.0041 | 0.0048 | 0.3971 |
| rs7144307 | ALM | HbA1C | -0.0122 | 69533837 | 0.002 | -0.0033 | 0.0035 | 0.3546 |
| rs7185244 | ALM | HbA1C | -0.0148 | 86546887 | 0.0023 | -0.0022 | 0.0042 | 0.6017 |
| rs718603 | ALM | HbA1C | 0.0131 | 2644245 | 0.0021 | 0.0007 | 0.0038 | 0.8483 |
| rs7220127 | ALM | HbA1C | -0.0105 | 64545922 | 0.0019 | 0.003 | 0.0037 | 0.407 |
| rs7229520 | ALM | HbA1C | -0.0224 | 46516468 | 0.002 | -0.0049 | 0.0038 | 0.1976 |
| rs723149 | ALM | HbA1C | -0.0276 | 46577056 | 0.0019 | 0.0084 | 0.0039 | 0.0322901 |
| rs72657800 | ALM | HbA1C | -0.0219 | 90822051 | 0.0035 | 0.008 | 0.0065 | 0.2184 |
| rs72695791 | ALM | HbA1C | -0.0297 | 184059452 | 0.0051 | 0.0062 | 0.0092 | 0.4984 |
| rs72801843 | ALM | HbA1C | 0.0313 | 53508802 | 0.0021 | -0.008 | 0.0036 | 0.02716 |
| rs72809820 | ALM | HbA1C | -0.0111 | 97360079 | 0.002 | 0.0106 | 0.0036 | 0.003046 |
| rs73006226 | ALM | HbA1C | -0.0182 | 108072728 | 0.0029 | 0.002 | 0.0049 | 0.684499 |
| rs7301341 | ALM | HbA1C | -0.0255 | 94083105 | 0.002 | 0.0033 | 0.0037 | 0.3786 |
| rs73052033 | ALM | HbA1C | -0.0151 | 185828465 | 0.0024 | 0.0009 | 0.0043 | 0.8403 |
| rs7320878 | ALM | HbA1C | -0.015 | 91994132 | 0.0019 | 0.0016 | 0.0034 | 0.6334 |
| rs7328187 | ALM | HbA1C | 0.0116 | 74189974 | 0.0019 | -0.0068 | 0.0036 | 0.0568997 |
| rs73384223 | ALM | HbA1C | -0.0205 | 3869315 | 0.0024 | 0.0053 | 0.0043 | 0.2144 |
| rs7367519 | ALM | HbA1C | 0.0164 | 204479176 | 0.002 | -0.0054 | 0.0037 | 0.1514 |
| rs7418410 | ALM | HbA1C | 0.0155 | 10236402 | 0.0019 | 0.001 | 0.0035 | 0.7819 |
| rs74379684 | ALM | HbA1C | -0.0272 | 94050205 | 0.0036 | 0.0007 | 0.0064 | 0.9119 |
| rs74494415 | ALM | HbA1C | -0.0417 | 74972138 | 0.0049 | -0.0146 | 0.008 | 0.0676099 |
| rs7485647 | ALM | HbA1C | -0.0261 | 131631133 | 0.0026 | 0.0078 | 0.0046 | 0.0900492 |
| rs7522400 | ALM | HbA1C | 0.0129 | 36613380 | 0.0022 | 0.0002 | 0.0041 | 0.9638 |
| rs7543202 | ALM | HbA1C | 0.0129 | 73872885 | 0.0019 | 0.0052 | 0.0035 | 0.132 |
| rs7570235 | ALM | HbA1C | -0.0168 | 242491353 | 0.0019 | 0.0075 | 0.0077 | 0.3303 |
| rs757834 | ALM | HbA1C | 0.0256 | 139717200 | 0.0024 | -0.0072 | 0.0041 | 0.0757408 |
| rs7598430 | ALM | HbA1C | -0.016 | 219193963 | 0.0019 | -0.0028 | 0.0036 | 0.4368 |
| rs7610055 | ALM | HbA1C | -0.0373 | 12388409 | 0.0029 | -0.0039 | 0.0104 | 0.707499 |
| rs7633464 | ALM | HbA1C | 0.0175 | 98715823 | 0.0019 | -0.0014 | 0.0034 | 0.6878 |
| rs76517946 | ALM | HbA1C | -0.0368 | 68354936 | 0.0035 | 0.0062 | 0.0068 | 0.3594 |
| rs7679276 | ALM | HbA1C | -0.033 | 146860186 | 0.0048 | -0.0048 | 0.0185 | 0.793299 |
| rs7689420 | ALM | HbA1C | 0.0466 | 145568352 | 0.0025 | -0.004 | 0.0046 | 0.3795 |
| rs7701233 | ALM | HbA1C | -0.0179 | 171218388 | 0.0019 | -0.0018 | 0.0035 | 0.6102 |
| rs772222 | ALM | HbA1C | 0.0121 | 52356892 | 0.0021 | -0.0034 | 0.0065 | 0.6025 |
| rs7731023 | ALM | HbA1C | 0.0166 | 36181627 | 0.0019 | 0.0016 | 0.0038 | 0.6827 |
| rs7735891 | ALM | HbA1C | 0.0259 | 131597005 | 0.0019 | -0.0002 | 0.0034 | 0.9472 |
| rs77447813 | ALM | HbA1C | 0.0224 | 50827041 | 0.0034 | -0.0024 | 0.0061 | 0.6871 |
| rs7768973 | ALM | HbA1C | -0.024 | 109745325 | 0.0019 | -0.001 | 0.0058 | 0.8649 |
| rs77809369 | ALM | HbA1C | 0.0237 | 9052448 | 0.0039 | 0.0051 | 0.0069 | 0.4626 |
| rs7816345 | ALM | HbA1C | 0.0255 | 36846109 | 0.0025 | 0.0083 | 0.0044 | 0.0559603 |
| rs7826059 | ALM | HbA1C | 0.0114 | 22512068 | 0.002 | -0.002 | 0.0035 | 0.5622 |
| rs7828086 | ALM | HbA1C | 0.0135 | 120843775 | 0.0022 | 0.0003 | 0.0039 | 0.9305 |
| rs7858712 | ALM | HbA1C | 0.0347 | 16738312 | 0.0034 | -0.0147 | 0.0062 | 0.0169301 |
| rs7863102 | ALM | HbA1C | -0.011 | 73963468 | 0.0019 | 0.004 | 0.0034 | 0.2392 |
| rs7902 | ALM | HbA1C | 0.0149 | 95565288 | 0.0019 | -0.0022 | 0.0034 | 0.5224 |
| rs79066296 | ALM | HbA1C | -0.0169 | 76391462 | 0.0022 | 0.0034 | 0.004 | 0.3963 |
| rs7971536 | ALM | HbA1C | -0.0194 | 102373788 | 0.0019 | 0.0033 | 0.0036 | 0.3504 |
| rs798548 | ALM | HbA1C | -0.0359 | 2760935 | 0.0021 | -0.0037 | 0.0037 | 0.3214 |
| rs8000973 | ALM | HbA1C | 0.0134 | 100691367 | 0.0019 | 0.0026 | 0.0035 | 0.4478 |
| rs8017006 | ALM | HbA1C | 0.0122 | 42745052 | 0.002 | 0.0018 | 0.0037 | 0.6326 |
| rs8018486 | ALM | HbA1C | -0.0138 | 39818616 | 0.0024 | -0.0034 | 0.0041 | 0.416 |
| rs8019890 | ALM | HbA1C | 0.025 | 21538067 | 0.0019 | 0.0061 | 0.0096 | 0.525801 |
| rs8020095 | ALM | HbA1C | -0.0145 | 67453858 | 0.0027 | -0.0048 | 0.0048 | 0.325 |
| rs80295797 | ALM | HbA1C | -0.0198 | 23341690 | 0.002 | -0.0078 | 0.0036 | 0.0289301 |
| rs8042578 | ALM | HbA1C | 0.0287 | 66992964 | 0.0022 | -0.0076 | 0.0039 | 0.0527704 |
| rs8054549 | ALM | HbA1C | -0.0251 | 86417234 | 0.0019 | 0.0052 | 0.0034 | 0.1297 |
| rs8084413 | ALM | HbA1C | -0.0127 | 22869123 | 0.0019 | -0.0021 | 0.0036 | 0.5479 |
| rs8136517 | ALM | HbA1C | 0.0267 | 46439433 | 0.0039 | 0.008 | 0.0094 | 0.391 |
| rs839255 | ALM | HbA1C | -0.0126 | 57974580 | 0.0021 | -0.0004 | 0.0036 | 0.9086 |
| rs861674 | ALM | HbA1C | 0.0128 | 112064475 | 0.0019 | 0.0073 | 0.0034 | 0.0318999 |
| rs867529 | ALM | HbA1C | 0.0184 | 88913273 | 0.0021 | -0.001 | 0.0038 | 0.7903 |
| rs876122 | ALM | HbA1C | 0.0162 | 6886297 | 0.0029 | 0.0064 | 0.0057 | 0.2655 |
| rs900399 | ALM | HbA1C | 0.0164 | 156798732 | 0.0019 | 0.0026 | 0.0035 | 0.4608 |
| rs905938 | ALM | HbA1C | 0.0394 | 154991389 | 0.0021 | -0.0063 | 0.0045 | 0.1674 |
| rs909220 | ALM | HbA1C | -0.015 | 75908780 | 0.0019 | -0.0034 | 0.0034 | 0.3231 |
| rs9343327 | ALM | HbA1C | 0.014 | 76606296 | 0.0019 | 0.0058 | 0.0034 | 0.0876193 |
| rs9344126 | ALM | HbA1C | -0.0185 | 81907559 | 0.0019 | -0.0009 | 0.0035 | 0.8022 |
| rs9375188 | ALM | HbA1C | 0.0136 | 98555272 | 0.0019 | 0.0047 | 0.0035 | 0.1786 |
| rs9385002 | ALM | HbA1C | -0.0147 | 117552469 | 0.0022 | -0.0049 | 0.0038 | 0.1898 |
| rs9388490 | ALM | HbA1C | 0.0462 | 126704795 | 0.0019 | 0.0097 | 0.0035 | 0.00504696 |
| rs9391254 | ALM | HbA1C | 0.0166 | 105377347 | 0.002 | -0.0022 | 0.0037 | 0.554201 |
| rs947099 | ALM | HbA1C | 0.0117 | 31129883 | 0.002 | 0.0019 | 0.0035 | 0.5863 |
| rs951366 | ALM | HbA1C | 0.0205 | 205685352 | 0.0019 | 0.0048 | 0.0035 | 0.1686 |
| rs9517483 | ALM | HbA1C | -0.0181 | 99572712 | 0.0021 | -0.0087 | 0.0037 | 0.0177301 |
| rs9525326 | ALM | HbA1C | -0.0184 | 115075715 | 0.0024 | -0.0073 | 0.0097 | 0.4513 |
| rs9568031 | ALM | HbA1C | -0.0115 | 48897520 | 0.0021 | -0.0001 | 0.0039 | 0.9707 |
| rs9590328 | ALM | HbA1C | 0.0153 | 96448383 | 0.0027 | -0.0031 | 0.0047 | 0.5156 |
| rs9594714 | ALM | HbA1C | 0.0144 | 42800481 | 0.0021 | 0.0015 | 0.0039 | 0.704399 |
| rs963317 | ALM | HbA1C | -0.0136 | 45129970 | 0.002 | -0.0102 | 0.0081 | 0.2095 |
| rs9647379 | ALM | HbA1C | 0.0215 | 171785168 | 0.0019 | 0.0062 | 0.0043 | 0.1523 |
| rs9828525 | ALM | HbA1C | 0.0121 | 61552810 | 0.0019 | -0.0011 | 0.0035 | 0.747699 |
| rs9838614 | ALM | HbA1C | -0.0185 | 38537671 | 0.0019 | 0.0015 | 0.0035 | 0.668499 |
| rs987666 | ALM | HbA1C | 0.0185 | 116267938 | 0.0029 | 0.0023 | 0.0055 | 0.6834 |
| rs9890062 | ALM | HbA1C | 0.0267 | 17434352 | 0.0039 | 0.0151 | 0.0089 | 0.0898401 |
| rs9898189 | ALM | HbA1C | -0.0163 | 80480516 | 0.0021 | -0.0005 | 0.0036 | 0.8972 |
| rs990315 | ALM | HbA1C | -0.0115 | 69578811 | 0.002 | -0.007 | 0.0035 | 0.04671 |
| rs9905385 | ALM | HbA1C | -0.0339 | 59498250 | 0.002 | -0.0063 | 0.0037 | 0.0838706 |
| rs9957318 | ALM | HbA1C | 0.0187 | 33039106 | 0.002 | 0.005 | 0.0065 | 0.4427 |
| rs10005035 | ALM | Two-hour glucose challenge | -0.0175 | 12865684 | 0.0021 | 0.0085 | 0.021 | 0.6912 |
| rs10019221 | ALM | Two-hour glucose challenge | -0.0124 | 21785364 | 0.0019 | 0.0029 | 0.019 | 0.8812 |
| rs10036789 | ALM | Two-hour glucose challenge | 0.0163 | 71695918 | 0.0019 | -0.0065 | 0.018 | 0.7258 |
| rs1005723 | ALM | Two-hour glucose challenge | 0.0161 | 243646251 | 0.0024 | -0.034 | 0.023 | 0.1411 |
| rs10068640 | ALM | Two-hour glucose challenge | 0.0112 | 123981977 | 0.002 | -0.02 | 0.019 | 0.3069 |
| rs10075249 | ALM | Two-hour glucose challenge | 0.0143 | 52846505 | 0.0019 | 0.033 | 0.019 | 0.0778305 |
| rs10107388 | ALM | Two-hour glucose challenge | -0.0159 | 145004944 | 0.002 | 0.029 | 0.019 | 0.1309 |
| rs10112506 | ALM | Two-hour glucose challenge | -0.012 | 13164746 | 0.0019 | -0.014 | 0.019 | 0.4752 |
| rs10123619 | ALM | Two-hour glucose challenge | -0.0171 | 119353611 | 0.0026 | -0.0094 | 0.024 | 0.6952 |
| rs10171272 | ALM | Two-hour glucose challenge | 0.0136 | 25946636 | 0.002 | 0.018 | 0.021 | 0.3851 |
| rs10202701 | ALM | Two-hour glucose challenge | 0.0227 | 232328681 | 0.0019 | 0.0094 | 0.018 | 0.6087 |
| rs10202845 | ALM | Two-hour glucose challenge | -0.0288 | 42575820 | 0.003 | -0.016 | 0.027 | 0.5569 |
| rs10203320 | ALM | Two-hour glucose challenge | 0.0138 | 9771620 | 0.002 | 0.014 | 0.02 | 0.5049 |
| rs10203386 | ALM | Two-hour glucose challenge | -0.0238 | 25136866 | 0.0019 | -0.0017 | 0.019 | 0.9297 |
| rs10205141 | ALM | Two-hour glucose challenge | 0.0241 | 11313340 | 0.0044 | 0.0002 | 0.035 | 0.9963 |
| rs10221831 | ALM | Two-hour glucose challenge | 0.03 | 202107829 | 0.0053 | -0.012 | 0.049 | 0.8118 |
| rs10225945 | ALM | Two-hour glucose challenge | -0.0146 | 28250083 | 0.0026 | -0.013 | 0.027 | 0.643701 |
| rs10242866 | ALM | Two-hour glucose challenge | 0.0157 | 17920613 | 0.0019 | -0.051 | 0.019 | 0.00773197 |
| rs10283100 | ALM | Two-hour glucose challenge | 0.0575 | 120596023 | 0.0041 | -0.032 | 0.057 | 0.576301 |
| rs1035583 | ALM | Two-hour glucose challenge | 0.0148 | 207326937 | 0.0019 | 0.026 | 0.02 | 0.1939 |
| rs10471339 | ALM | Two-hour glucose challenge | -0.011 | 67823773 | 0.0019 | -0.0034 | 0.02 | 0.8631 |
| rs1047891 | ALM | Two-hour glucose challenge | 0.0233 | 211540507 | 0.002 | -0.015 | 0.022 | 0.4837 |
| rs10483727 | ALM | Two-hour glucose challenge | -0.0368 | 61072875 | 0.0019 | -0.0087 | 0.019 | 0.6536 |
| rs1056747 | ALM | Two-hour glucose challenge | -0.0155 | 35690102 | 0.0019 | -0.019 | 0.019 | 0.3271 |
| rs1063582 | ALM | Two-hour glucose challenge | -0.0185 | 23167353 | 0.0022 | 0.0099 | 0.022 | 0.647099 |
| rs10657263 | ALM | Two-hour glucose challenge | -0.013 | 49690460 | 0.0019 | -0.0054 | 0.019 | 0.774801 |
| rs10748128 | ALM | Two-hour glucose challenge | 0.0255 | 69827658 | 0.002 | 0.024 | 0.023 | 0.293 |
| rs10776560 | ALM | Two-hour glucose challenge | -0.0157 | 50542358 | 0.0019 | 0.021 | 0.019 | 0.2624 |
| rs10796828 | ALM | Two-hour glucose challenge | 0.0154 | 69490346 | 0.002 | 0.047 | 0.019 | 0.0157101 |
| rs10807137 | ALM | Two-hour glucose challenge | -0.0455 | 34183026 | 0.0025 | 0.042 | 0.029 | 0.1447 |
| rs10815274 | ALM | Two-hour glucose challenge | 0.0124 | 5728968 | 0.0019 | -0.025 | 0.019 | 0.1782 |
| rs10822117 | ALM | Two-hour glucose challenge | -0.0176 | 52786701 | 0.0022 | 0.0003 | 0.022 | 0.9887 |
| rs10829226 | ALM | Two-hour glucose challenge | -0.0112 | 27573952 | 0.002 | 0.018 | 0.02 | 0.3562 |
| rs10832963 | ALM | Two-hour glucose challenge | -0.0203 | 18664241 | 0.0022 | 0.0045 | 0.021 | 0.8251 |
| rs10845408 | ALM | Two-hour glucose challenge | 0.0255 | 11880581 | 0.002 | 0.019 | 0.019 | 0.3243 |
| rs10858246 | ALM | Two-hour glucose challenge | -0.0188 | 139102831 | 0.002 | 0.012 | 0.026 | 0.646501 |
| rs10922475 | ALM | Two-hour glucose challenge | 0.0159 | 89142142 | 0.0019 | 0.019 | 0.019 | 0.3308 |
| rs10948 | ALM | Two-hour glucose challenge | -0.0252 | 10754905 | 0.002 | 0.069 | 0.02 | 0.000461296 |
| rs10962212 | ALM | Two-hour glucose challenge | 0.0143 | 15911745 | 0.0019 | 0.0063 | 0.019 | 0.7417 |
| rs11009928 | ALM | Two-hour glucose challenge | -0.0148 | 35058712 | 0.0022 | -0.034 | 0.021 | 0.1065 |
| rs11014285 | ALM | Two-hour glucose challenge | 0.0342 | 25178864 | 0.0026 | 0.018 | 0.034 | 0.596901 |
| rs11042717 | ALM | Two-hour glucose challenge | -0.029 | 10303939 | 0.0019 | 0.011 | 0.018 | 0.5618 |
| rs11049704 | ALM | Two-hour glucose challenge | -0.0183 | 28691701 | 0.0021 | 0.014 | 0.021 | 0.4972 |
| rs11060942 | ALM | Two-hour glucose challenge | -0.0354 | 123434524 | 0.0052 | -0.029 | 0.048 | 0.5534 |
| rs11068230 | ALM | Two-hour glucose challenge | 0.0238 | 117349014 | 0.0028 | -0.074 | 0.027 | 0.00550402 |
| rs11098677 | ALM | Two-hour glucose challenge | -0.0263 | 123833516 | 0.0023 | 0.012 | 0.024 | 0.6255 |
| rs111622870 | ALM | Two-hour glucose challenge | -0.0282 | 2613109 | 0.0044 | -0.019 | 0.05 | 0.704301 |
| rs11175919 | ALM | Two-hour glucose challenge | 0.0349 | 66180277 | 0.0059 | 0.032 | 0.07 | 0.652701 |
| rs111901094 | ALM | Two-hour glucose challenge | -0.0253 | 19513570 | 0.0025 | 0.0074 | 0.029 | 0.8027 |
| rs11198591 | ALM | Two-hour glucose challenge | 0.0148 | 120515892 | 0.002 | -0.017 | 0.022 | 0.4429 |
| rs11210892 | ALM | Two-hour glucose challenge | 0.0118 | 44100084 | 0.002 | 0.014 | 0.02 | 0.473 |
| rs112153300 | ALM | Two-hour glucose challenge | 0.0261 | 47547474 | 0.0034 | -0.049 | 0.043 | 0.2515 |
| rs11233117 | ALM | Two-hour glucose challenge | -0.0176 | 69924352 | 0.0019 | -0.032 | 0.019 | 0.0995199 |
| rs11243202 | ALM | Two-hour glucose challenge | 0.0302 | 7719065 | 0.0019 | -0.0036 | 0.019 | 0.8521 |
| rs112537273 | ALM | Two-hour glucose challenge | -0.0212 | 38248306 | 0.0022 | 0.025 | 0.022 | 0.2457 |
| rs11260623 | ALM | Two-hour glucose challenge | 0.0117 | 1781456 | 0.0019 | -0.015 | 0.018 | 0.4102 |
| rs113232639 | ALM | Two-hour glucose challenge | 0.0327 | 20715656 | 0.0019 | -0.0064 | 0.021 | 0.7652 |
| rs113671109 | ALM | Two-hour glucose challenge | -0.015 | 12620885 | 0.0023 | 0.0046 | 0.023 | 0.8453 |
| rs113898003 | ALM | Two-hour glucose challenge | -0.036 | 130341235 | 0.0021 | 0.021 | 0.023 | 0.3466 |
| rs115010283 | ALM | Two-hour glucose challenge | 0.034 | 172162393 | 0.002 | 0.017 | 0.02 | 0.381 |
| rs115105539 | ALM | Two-hour glucose challenge | 0.0231 | 49409496 | 0.0025 | -0.027 | 0.026 | 0.2947 |
| rs11590254 | ALM | Two-hour glucose challenge | 0.0186 | 92316573 | 0.002 | -0.034 | 0.02 | 0.0921701 |
| rs116052377 | ALM | Two-hour glucose challenge | 0.0225 | 124787756 | 0.0035 | -0.046 | 0.037 | 0.2065 |
| rs11605297 | ALM | Two-hour glucose challenge | 0.0146 | 58296806 | 0.0022 | -0.011 | 0.021 | 0.5961 |
| rs11633371 | ALM | Two-hour glucose challenge | 0.0216 | 89356832 | 0.0019 | -0.045 | 0.019 | 0.0176901 |
| rs116339650 | ALM | Two-hour glucose challenge | -0.0175 | 26200972 | 0.0029 | 0.047 | 0.03 | 0.118 |
| rs11672848 | ALM | Two-hour glucose challenge | -0.0171 | 37570704 | 0.0019 | 0.032 | 0.018 | 0.0882104 |
| rs1168768 | ALM | Two-hour glucose challenge | 0.0332 | 66509650 | 0.006 | 0.02 | 0.09 | 0.8253 |
| rs11727162 | ALM | Two-hour glucose challenge | -0.017 | 88606761 | 0.0019 | 0.051 | 0.018 | 0.00604004 |
| rs11778491 | ALM | Two-hour glucose challenge | -0.0247 | 120451362 | 0.0022 | 0.0017 | 0.022 | 0.9388 |
| rs1190540 | ALM | Two-hour glucose challenge | 0.0125 | 102897009 | 0.0021 | -0.0068 | 0.02 | 0.7302 |
| rs11959466 | ALM | Two-hour glucose challenge | 0.038 | 42803824 | 0.0042 | 0.093 | 0.049 | 0.0567401 |
| rs1202186 | ALM | Two-hour glucose challenge | -0.012 | 87213258 | 0.002 | 0.036 | 0.02 | 0.0695505 |
| rs12051245 | ALM | Two-hour glucose challenge | 0.0299 | 783865 | 0.0022 | -0.0021 | 0.026 | 0.9366 |
| rs12074850 | ALM | Two-hour glucose challenge | 0.0393 | 51248316 | 0.0033 | 0.022 | 0.034 | 0.5141 |
| rs12099669 | ALM | Two-hour glucose challenge | 0.0331 | 46783653 | 0.002 | 0.026 | 0.02 | 0.1968 |
| rs12188208 | ALM | Two-hour glucose challenge | -0.0195 | 77442791 | 0.0022 | -0.0011 | 0.023 | 0.9609 |
| rs12230946 | ALM | Two-hour glucose challenge | 0.0271 | 53498725 | 0.0033 | -0.014 | 0.03 | 0.643 |
| rs12334478 | ALM | Two-hour glucose challenge | -0.0161 | 141998765 | 0.0019 | 0.017 | 0.018 | 0.3496 |
| rs12340775 | ALM | Two-hour glucose challenge | -0.0287 | 13226945 | 0.0043 | -0.0063 | 0.036 | 0.8611 |
| rs12344515 | ALM | Two-hour glucose challenge | -0.0163 | 113801231 | 0.0022 | 0.0088 | 0.022 | 0.693201 |
| rs12347137 | ALM | Two-hour glucose challenge | -0.046 | 119122721 | 0.0024 | 0.013 | 0.023 | 0.5668 |
| rs12351226 | ALM | Two-hour glucose challenge | 0.0218 | 98405230 | 0.0025 | 0.059 | 0.029 | 0.0419402 |
| rs12461874 | ALM | Two-hour glucose challenge | -0.0181 | 17180358 | 0.0021 | 0.012 | 0.022 | 0.5967 |
| rs12483401 | ALM | Two-hour glucose challenge | -0.0387 | 35443829 | 0.0067 | 0.11 | 0.063 | 0.0837992 |
| rs12512942 | ALM | Two-hour glucose challenge | -0.0162 | 177766307 | 0.002 | -0.0052 | 0.021 | 0.7992 |
| rs12517711 | ALM | Two-hour glucose challenge | -0.0147 | 60754661 | 0.0019 | 0.054 | 0.019 | 0.00450495 |
| rs12519407 | ALM | Two-hour glucose challenge | 0.0181 | 137651012 | 0.0022 | -0.011 | 0.02 | 0.589899 |
| rs12533452 | ALM | Two-hour glucose challenge | 0.0237 | 19016871 | 0.0026 | -0.015 | 0.026 | 0.5703 |
| rs12541381 | ALM | Two-hour glucose challenge | -0.0319 | 135649848 | 0.0022 | 0.066 | 0.025 | 0.00756798 |
| rs12563442 | ALM | Two-hour glucose challenge | 0.0122 | 19786695 | 0.0021 | 0.047 | 0.021 | 0.0270097 |
| rs1260326 | ALM | Two-hour glucose challenge | 0.0323 | 27730940 | 0.0019 | -0.091 | 0.019 | 0.000001525 |
| rs12616192 | ALM | Two-hour glucose challenge | -0.0261 | 121568931 | 0.0038 | -0.013 | 0.045 | 0.776201 |
| rs12655296 | ALM | Two-hour glucose challenge | -0.011 | 15890643 | 0.002 | -0.016 | 0.019 | 0.4026 |
| rs12672217 | ALM | Two-hour glucose challenge | 0.0139 | 156310948 | 0.002 | 0.0067 | 0.019 | 0.7305 |
| rs12702693 | ALM | Two-hour glucose challenge | 0.0173 | 8101039 | 0.0019 | -0.0061 | 0.019 | 0.7465 |
| rs12724708 | ALM | Two-hour glucose challenge | 0.0243 | 219620569 | 0.002 | -0.017 | 0.02 | 0.3997 |
| rs12773500 | ALM | Two-hour glucose challenge | 0.0171 | 81232632 | 0.0028 | 0.0092 | 0.025 | 0.7191 |
| rs12831751 | ALM | Two-hour glucose challenge | 0.0172 | 29520017 | 0.0021 | -0.014 | 0.021 | 0.5032 |
| rs12882130 | ALM | Two-hour glucose challenge | -0.0202 | 103878774 | 0.002 | 0.0032 | 0.02 | 0.871 |
| rs12907139 | ALM | Two-hour glucose challenge | -0.0149 | 73521566 | 0.0019 | 0.019 | 0.018 | 0.2965 |
| rs1290786 | ALM | Two-hour glucose challenge | -0.0143 | 169097381 | 0.0019 | -0.028 | 0.019 | 0.1293 |
| rs12909863 | ALM | Two-hour glucose challenge | 0.0189 | 75825822 | 0.0022 | -0.05 | 0.021 | 0.0200498 |
| rs1291114 | ALM | Two-hour glucose challenge | 0.0173 | 35500850 | 0.0031 | 0.041 | 0.029 | 0.1592 |
| rs12926103 | ALM | Two-hour glucose challenge | 0.0272 | 86371775 | 0.0038 | -0.013 | 0.04 | 0.755 |
| rs12962050 | ALM | Two-hour glucose challenge | 0.0153 | 35179808 | 0.002 | -0.014 | 0.02 | 0.489199 |
| rs12997625 | ALM | Two-hour glucose challenge | -0.017 | 202970250 | 0.0019 | 0.0053 | 0.019 | 0.776599 |
| rs13037813 | ALM | Two-hour glucose challenge | 0.0292 | 47750588 | 0.0022 | 0.018 | 0.022 | 0.4164 |
| rs13103161 | ALM | Two-hour glucose challenge | -0.0284 | 106216459 | 0.0019 | -0.02 | 0.019 | 0.3158 |
| rs13123591 | ALM | Two-hour glucose challenge | 0.0185 | 120105990 | 0.002 | 0.036 | 0.021 | 0.0758298 |
| rs13170063 | ALM | Two-hour glucose challenge | -0.0152 | 157895013 | 0.0019 | -0.023 | 0.019 | 0.2292 |
| rs13209685 | ALM | Two-hour glucose challenge | 0.0277 | 7779729 | 0.0026 | 0.012 | 0.026 | 0.6346 |
| rs1324538 | ALM | Two-hour glucose challenge | 0.0237 | 45080144 | 0.0019 | 0.013 | 0.022 | 0.5568 |
| rs1325596 | ALM | Two-hour glucose challenge | 0.0287 | 176794066 | 0.0019 | -0.063 | 0.018 | 0.000664798 |
| rs1330826 | ALM | Two-hour glucose challenge | 0.0162 | 85129970 | 0.0023 | 0.003 | 0.025 | 0.9045 |
| rs13391980 | ALM | Two-hour glucose challenge | -0.0225 | 165504841 | 0.0029 | -0.041 | 0.03 | 0.1655 |
| rs1340022 | ALM | Two-hour glucose challenge | 0.0118 | 131334465 | 0.0019 | -0.029 | 0.019 | 0.1225 |
| rs1405227 | ALM | Two-hour glucose challenge | 0.0129 | 98873390 | 0.002 | 0.025 | 0.021 | 0.221 |
| rs1443536 | ALM | Two-hour glucose challenge | 0.0218 | 82174165 | 0.0021 | -0.0098 | 0.02 | 0.6269 |
| rs1444628 | ALM | Two-hour glucose challenge | 0.024 | 20563643 | 0.002 | -0.018 | 0.021 | 0.3868 |
| rs1472852 | ALM | Two-hour glucose challenge | -0.0638 | 17910236 | 0.0026 | 0.045 | 0.027 | 0.0949708 |
| rs1478575 | ALM | Two-hour glucose challenge | 0.0312 | 218278555 | 0.002 | -0.022 | 0.019 | 0.2539 |
| rs14976 | ALM | Two-hour glucose challenge | 0.0144 | 85818886 | 0.002 | -0.015 | 0.021 | 0.4823 |
| rs1514134 | ALM | Two-hour glucose challenge | -0.0114 | 56116513 | 0.0019 | 0.0009 | 0.019 | 0.9622 |
| rs1556659 | ALM | Two-hour glucose challenge | 0.0163 | 130834698 | 0.002 | 0.011 | 0.021 | 0.598401 |
| rs1584011 | ALM | Two-hour glucose challenge | 0.0159 | 27080527 | 0.002 | -0.027 | 0.019 | 0.152 |
| rs165849 | ALM | Two-hour glucose challenge | 0.0157 | 19958669 | 0.0021 | 0.013 | 0.021 | 0.541101 |
| rs17197114 | ALM | Two-hour glucose challenge | 0.0177 | 21894526 | 0.0025 | 0.03 | 0.028 | 0.2738 |
| rs17205463 | ALM | Two-hour glucose challenge | -0.0263 | 62381413 | 0.0019 | 0.081 | 0.019 | 1.51199E-05 |
| rs17246129 | ALM | Two-hour glucose challenge | 0.0254 | 227259964 | 0.002 | -0.012 | 0.02 | 0.537 |
| rs1730028 | ALM | Two-hour glucose challenge | 0.0131 | 157900789 | 0.0019 | -0.011 | 0.019 | 0.5496 |
| rs173135 | ALM | Two-hour glucose challenge | -0.0341 | 68172326 | 0.003 | 0.0055 | 0.03 | 0.856 |
| rs17400325 | ALM | Two-hour glucose challenge | 0.0345 | 178565913 | 0.0047 | -0.023 | 0.042 | 0.5882 |
| rs17478946 | ALM | Two-hour glucose challenge | -0.0192 | 24093062 | 0.0021 | -0.013 | 0.023 | 0.5732 |
| rs17681189 | ALM | Two-hour glucose challenge | -0.0131 | 65976175 | 0.0019 | 0.0082 | 0.019 | 0.660999 |
| rs17718736 | ALM | Two-hour glucose challenge | 0.0115 | 71555205 | 0.002 | -0.0038 | 0.02 | 0.8493 |
| rs17773965 | ALM | Two-hour glucose challenge | -0.0163 | 217631338 | 0.0027 | 0.049 | 0.025 | 0.0510105 |
| rs17818592 | ALM | Two-hour glucose challenge | -0.0129 | 86088594 | 0.0019 | 0.012 | 0.019 | 0.5177 |
| rs1786263 | ALM | Two-hour glucose challenge | -0.019 | 13116432 | 0.0019 | 0.015 | 0.019 | 0.4223 |
| rs1797070 | ALM | Two-hour glucose challenge | 0.0219 | 218630201 | 0.0021 | 0.0065 | 0.021 | 0.7604 |
| rs1823217 | ALM | Two-hour glucose challenge | -0.0181 | 134380959 | 0.002 | 0.04 | 0.02 | 0.0500104 |
| rs188617336 | ALM | Two-hour glucose challenge | 0.0138 | 20610730 | 0.0021 | 0.014 | 0.021 | 0.508401 |
| rs1899040 | ALM | Two-hour glucose challenge | 0.0152 | 223901896 | 0.0023 | 0.0043 | 0.024 | 0.8537 |
| rs1933081 | ALM | Two-hour glucose challenge | 0.0267 | 151651505 | 0.0034 | 0.064 | 0.036 | 0.07787 |
| rs199647708 | ALM | Two-hour glucose challenge | 0.0114 | 125352195 | 0.0019 | -0.01 | 0.019 | 0.6004 |
| rs200439 | ALM | Two-hour glucose challenge | -0.0128 | 6716083 | 0.0023 | -0.018 | 0.023 | 0.431 |
| rs2005172 | ALM | Two-hour glucose challenge | 0.048 | 61996255 | 0.002 | -0.026 | 0.02 | 0.1885 |
| rs2019203 | ALM | Two-hour glucose challenge | 0.0189 | 36908672 | 0.0019 | -0.0066 | 0.018 | 0.723601 |
| rs2025609 | ALM | Two-hour glucose challenge | 0.0186 | 67422990 | 0.0026 | -0.0008 | 0.024 | 0.9719 |
| rs2025808 | ALM | Two-hour glucose challenge | 0.0122 | 184161757 | 0.0022 | 0.0046 | 0.021 | 0.8264 |
| rs2035901 | ALM | Two-hour glucose challenge | 0.024 | 145521867 | 0.0019 | -0.015 | 0.019 | 0.4306 |
| rs2101017 | ALM | Two-hour glucose challenge | -0.0223 | 122306857 | 0.0028 | 0.038 | 0.036 | 0.2924 |
| rs2112617 | ALM | Two-hour glucose challenge | -0.0167 | 46977125 | 0.0019 | 0.016 | 0.018 | 0.3842 |
| rs212526 | ALM | Two-hour glucose challenge | 0.0214 | 21584941 | 0.0019 | -0.005 | 0.019 | 0.7945 |
| rs2138374 | ALM | Two-hour glucose challenge | -0.0149 | 190014317 | 0.002 | -0.005 | 0.02 | 0.803 |
| rs2140619 | ALM | Two-hour glucose challenge | 0.0113 | 114007270 | 0.0019 | -0.03 | 0.019 | 0.1218 |
| rs2142331 | ALM | Two-hour glucose challenge | -0.0165 | 116636719 | 0.0019 | 0.0049 | 0.019 | 0.7998 |
| rs2174008 | ALM | Two-hour glucose challenge | -0.0192 | 38510456 | 0.0019 | 0.011 | 0.019 | 0.567799 |
| rs2181834 | ALM | Two-hour glucose challenge | 0.0254 | 102661251 | 0.0019 | 0.0057 | 0.018 | 0.7592 |
| rs2188805 | ALM | Two-hour glucose challenge | 0.0114 | 93078400 | 0.002 | -0.032 | 0.022 | 0.1352 |
| rs2209098 | ALM | Two-hour glucose challenge | 0.024 | 172167226 | 0.002 | -0.0093 | 0.02 | 0.6385 |
| rs2212926 | ALM | Two-hour glucose challenge | -0.022 | 38066883 | 0.0023 | 0.0054 | 0.028 | 0.8456 |
| rs2229840 | ALM | Two-hour glucose challenge | 0.0341 | 124826462 | 0.0026 | 0.0045 | 0.029 | 0.8756 |
| rs2230033 | ALM | Two-hour glucose challenge | -0.0265 | 39671476 | 0.0019 | 0.02 | 0.019 | 0.2732 |
| rs2236096 | ALM | Two-hour glucose challenge | 0.018 | 3266319 | 0.0023 | -0.029 | 0.023 | 0.2031 |
| rs2237485 | ALM | Two-hour glucose challenge | 0.0191 | 50749870 | 0.0023 | -0.0011 | 0.022 | 0.9586 |
| rs2240735 | ALM | Two-hour glucose challenge | 0.0189 | 4027605 | 0.0022 | -0.036 | 0.025 | 0.1472 |
| rs2268718 | ALM | Two-hour glucose challenge | 0.0141 | 52415023 | 0.0021 | 0.0056 | 0.021 | 0.7879 |
| rs2270894 | ALM | Two-hour glucose challenge | -0.0332 | 9975386 | 0.0024 | 0.024 | 0.03 | 0.4202 |
| rs2274351 | ALM | Two-hour glucose challenge | 0.017 | 104264107 | 0.0019 | -0.011 | 0.019 | 0.562501 |
| rs2283200 | ALM | Two-hour glucose challenge | -0.0281 | 2729340 | 0.0042 | 0.0099 | 0.042 | 0.8142 |
| rs2287821 | ALM | Two-hour glucose challenge | -0.0153 | 33935102 | 0.0019 | -0.033 | 0.018 | 0.0719499 |
| rs2289629 | ALM | Two-hour glucose challenge | -0.0148 | 27959903 | 0.002 | 0.03 | 0.019 | 0.1279 |
| rs2296316 | ALM | Two-hour glucose challenge | -0.0192 | 65520246 | 0.0019 | -0.013 | 0.018 | 0.4765 |
| rs2303423 | ALM | Two-hour glucose challenge | 0.0168 | 38120029 | 0.003 | -0.0033 | 0.031 | 0.9162 |
| rs2305141 | ALM | Two-hour glucose challenge | 0.0183 | 233684402 | 0.0019 | -0.021 | 0.02 | 0.2861 |
| rs2324154 | ALM | Two-hour glucose challenge | 0.015 | 24027226 | 0.0019 | -0.0041 | 0.018 | 0.8238 |
| rs2347603 | ALM | Two-hour glucose challenge | -0.0181 | 47297426 | 0.0022 | 0.0037 | 0.021 | 0.8643 |
| rs2347808 | ALM | Two-hour glucose challenge | -0.0125 | 2750856 | 0.0019 | 0.013 | 0.019 | 0.5041 |
| rs2390669 | ALM | Two-hour glucose challenge | 0.0174 | 169091942 | 0.0028 | -0.011 | 0.029 | 0.7121 |
| rs2454390 | ALM | Two-hour glucose challenge | -0.0176 | 103255613 | 0.0026 | 0.028 | 0.027 | 0.2917 |
| rs2490302 | ALM | Two-hour glucose challenge | 0.0221 | 37702435 | 0.0034 | 0.011 | 0.032 | 0.7212 |
| rs249677 | ALM | Two-hour glucose challenge | -0.0109 | 141539339 | 0.002 | 0.0069 | 0.02 | 0.7228 |
| rs2521349 | ALM | Two-hour glucose challenge | 0.0155 | 67503501 | 0.0019 | -0.014 | 0.019 | 0.4741 |
| rs2529090 | ALM | Two-hour glucose challenge | 0.0136 | 24662280 | 0.0025 | -0.012 | 0.025 | 0.6236 |
| rs2545339 | ALM | Two-hour glucose challenge | 0.0115 | 149911219 | 0.002 | -0.0012 | 0.019 | 0.9493 |
| rs2578565 | ALM | Two-hour glucose challenge | -0.0141 | 5460569 | 0.002 | -0.015 | 0.019 | 0.451 |
| rs258794 | ALM | Two-hour glucose challenge | 0.0147 | 142540040 | 0.0021 | -0.021 | 0.021 | 0.3137 |
| rs2592208 | ALM | Two-hour glucose challenge | -0.0124 | 67408873 | 0.0019 | 0.035 | 0.019 | 0.0632703 |
| rs261223 | ALM | Two-hour glucose challenge | 0.0175 | 95901046 | 0.0019 | 0.0086 | 0.019 | 0.6569 |
| rs2648725 | ALM | Two-hour glucose challenge | 0.0165 | 93015079 | 0.0023 | -0.029 | 0.026 | 0.2499 |
| rs2663126 | ALM | Two-hour glucose challenge | -0.0139 | 99563857 | 0.0021 | -0.014 | 0.021 | 0.4848 |
| rs2717008 | ALM | Two-hour glucose challenge | -0.0127 | 58149158 | 0.0019 | -0.01 | 0.019 | 0.5954 |
| rs2748501 | ALM | Two-hour glucose challenge | -0.0195 | 146312258 | 0.0019 | 0.049 | 0.019 | 0.0126299 |
| rs2754255 | ALM | Two-hour glucose challenge | -0.0153 | 88393572 | 0.0023 | -0.0072 | 0.024 | 0.7583 |
| rs2763263 | ALM | Two-hour glucose challenge | -0.017 | 168814392 | 0.0022 | 0.017 | 0.022 | 0.437 |
| rs2764264 | ALM | Two-hour glucose challenge | 0.0203 | 108934461 | 0.0021 | 0.0044 | 0.021 | 0.8316 |
| rs2788213 | ALM | Two-hour glucose challenge | 0.0123 | 703249 | 0.0021 | -0.04 | 0.021 | 0.0560596 |
| rs2789365 | ALM | Two-hour glucose challenge | -0.0145 | 235515534 | 0.0019 | 0.014 | 0.019 | 0.4571 |
| rs2791654 | ALM | Two-hour glucose challenge | -0.0239 | 11129317 | 0.0022 | 0.042 | 0.021 | 0.0415796 |
| rs2807339 | ALM | Two-hour glucose challenge | 0.0162 | 22578063 | 0.0022 | 0.0038 | 0.021 | 0.86 |
| rs28529055 | ALM | Two-hour glucose challenge | -0.0147 | 92428216 | 0.0019 | -0.0077 | 0.019 | 0.6771 |
| rs28678024 | ALM | Two-hour glucose challenge | -0.0119 | 25937161 | 0.0021 | 0.0036 | 0.021 | 0.8635 |
| rs2871865 | ALM | Two-hour glucose challenge | -0.0493 | 99194896 | 0.003 | -0.057 | 0.032 | 0.0771205 |
| rs2871960 | ALM | Two-hour glucose challenge | 0.0469 | 141121814 | 0.0019 | -0.023 | 0.019 | 0.2241 |
| rs28736838 | ALM | Two-hour glucose challenge | -0.0117 | 120148713 | 0.002 | -0.02 | 0.02 | 0.3229 |
| rs291979 | ALM | Two-hour glucose challenge | 0.0242 | 121129797 | 0.0023 | 0.0058 | 0.023 | 0.7988 |
| rs2923411 | ALM | Two-hour glucose challenge | 0.0127 | 42455206 | 0.0019 | -0.0093 | 0.019 | 0.6324 |
| rs2925155 | ALM | Two-hour glucose challenge | -0.015 | 75886297 | 0.0022 | -0.023 | 0.021 | 0.2832 |
| rs293517 | ALM | Two-hour glucose challenge | -0.013 | 83662455 | 0.0021 | 0.0025 | 0.021 | 0.9081 |
| rs2971857 | ALM | Two-hour glucose challenge | -0.0119 | 234369487 | 0.0019 | 0.0032 | 0.02 | 0.8739 |
| rs2978362 | ALM | Two-hour glucose challenge | 0.0106 | 32959397 | 0.0019 | -0.02 | 0.018 | 0.2878 |
| rs301807 | ALM | Two-hour glucose challenge | -0.0144 | 8484823 | 0.0019 | -0.017 | 0.019 | 0.3743 |
| rs3103223 | ALM | Two-hour glucose challenge | 0.0126 | 42402721 | 0.0022 | -0.033 | 0.021 | 0.1214 |
| rs310796 | ALM | Two-hour glucose challenge | 0.0142 | 77453226 | 0.002 | 0.0032 | 0.02 | 0.8747 |
| rs3116194 | ALM | Two-hour glucose challenge | -0.0295 | 233061266 | 0.0032 | 0.0098 | 0.034 | 0.7739 |
| rs3116602 | ALM | Two-hour glucose challenge | -0.0612 | 51111355 | 0.0023 | 0.045 | 0.022 | 0.04341 |
| rs3184504 | ALM | Two-hour glucose challenge | 0.0183 | 111884608 | 0.0019 | -0.03 | 0.019 | 0.1116 |
| rs331917 | ALM | Two-hour glucose challenge | -0.0127 | 98158524 | 0.0019 | -0.0055 | 0.019 | 0.7756 |
| rs332116 | ALM | Two-hour glucose challenge | -0.0206 | 28926099 | 0.0021 | 0.0053 | 0.02 | 0.7951 |
| rs336630 | ALM | Two-hour glucose challenge | -0.0106 | 18607538 | 0.0019 | -0.0036 | 0.025 | 0.8873 |
| rs34338597 | ALM | Two-hour glucose challenge | -0.0112 | 106301580 | 0.0019 | 0.015 | 0.019 | 0.4463 |
| rs34390533 | ALM | Two-hour glucose challenge | -0.0257 | 184030838 | 0.0022 | 0.016 | 0.025 | 0.5311 |
| rs34776209 | ALM | Two-hour glucose challenge | -0.0317 | 23513093 | 0.0022 | 0.018 | 0.021 | 0.3861 |
| rs34879158 | ALM | Two-hour glucose challenge | -0.0363 | 32300634 | 0.0022 | -0.026 | 0.022 | 0.2289 |
| rs35073631 | ALM | Two-hour glucose challenge | 0.0112 | 22696964 | 0.0019 | 0.006 | 0.018 | 0.742499 |
| rs350832 | ALM | Two-hour glucose challenge | -0.0165 | 4069426 | 0.0023 | -0.074 | 0.036 | 0.0380496 |
| rs35288270 | ALM | Two-hour glucose challenge | -0.0328 | 4961278 | 0.0028 | -0.024 | 0.029 | 0.403 |
| rs35732917 | ALM | Two-hour glucose challenge | 0.0204 | 73013269 | 0.0021 | -0.02 | 0.022 | 0.3691 |
| rs36048468 | ALM | Two-hour glucose challenge | 0.0254 | 122879901 | 0.0023 | -0.026 | 0.023 | 0.2608 |
| rs3768495 | ALM | Two-hour glucose challenge | -0.0178 | 109935325 | 0.0021 | -0.0047 | 0.021 | 0.8193 |
| rs3769598 | ALM | Two-hour glucose challenge | 0.0171 | 32679732 | 0.0027 | 0.011 | 0.027 | 0.6991 |
| rs377599 | ALM | Two-hour glucose challenge | 0.0217 | 2164699 | 0.0019 | -0.032 | 0.019 | 0.0894396 |
| rs3778858 | ALM | Two-hour glucose challenge | 0.0108 | 129963356 | 0.002 | 0.018 | 0.021 | 0.3914 |
| rs3818416 | ALM | Two-hour glucose challenge | 0.0279 | 78474468 | 0.0022 | 0.046 | 0.022 | 0.0397301 |
| rs3822742 | ALM | Two-hour glucose challenge | 0.0162 | 139059017 | 0.002 | 0.0054 | 0.056 | 0.9232 |
| rs3828729 | ALM | Two-hour glucose challenge | -0.016 | 155554707 | 0.002 | -0.022 | 0.021 | 0.2906 |
| rs3901421 | ALM | Two-hour glucose challenge | 0.0215 | 96204538 | 0.0019 | 0.0006 | 0.019 | 0.9725 |
| rs395980 | ALM | Two-hour glucose challenge | -0.0184 | 177430072 | 0.0021 | 0.049 | 0.021 | 0.0183299 |
| rs40270 | ALM | Two-hour glucose challenge | 0.0151 | 55804552 | 0.0022 | 0.035 | 0.021 | 0.0864908 |
| rs4073154 | ALM | Two-hour glucose challenge | 0.0274 | 129035485 | 0.0023 | -0.046 | 0.023 | 0.0463095 |
| rs4076108 | ALM | Two-hour glucose challenge | 0.0174 | 13736088 | 0.0022 | -0.029 | 0.022 | 0.1924 |
| rs4077103 | ALM | Two-hour glucose challenge | -0.0143 | 49557732 | 0.0026 | -0.013 | 0.025 | 0.5985 |
| rs42039 | ALM | Two-hour glucose challenge | 0.0481 | 92244422 | 0.0022 | -0.036 | 0.022 | 0.0981409 |
| rs4282339 | ALM | Two-hour glucose challenge | -0.0311 | 168256240 | 0.0023 | 0.0025 | 0.023 | 0.914 |
| rs4287835 | ALM | Two-hour glucose challenge | 0.0147 | 31457337 | 0.0019 | -0.046 | 0.018 | 0.01239 |
| rs4360494 | ALM | Two-hour glucose challenge | -0.0198 | 38455891 | 0.0019 | -0.01 | 0.021 | 0.614899 |
| rs4380799 | ALM | Two-hour glucose challenge | -0.0255 | 32571864 | 0.0021 | 0.001 | 0.031 | 0.9738 |
| rs4602848 | ALM | Two-hour glucose challenge | 0.016 | 92186933 | 0.002 | -0.0053 | 0.019 | 0.7862 |
| rs4622329 | ALM | Two-hour glucose challenge | 0.0149 | 102321935 | 0.002 | -0.02 | 0.021 | 0.3336 |
| rs4640244 | ALM | Two-hour glucose challenge | -0.02 | 21284223 | 0.0019 | 0.034 | 0.023 | 0.1395 |
| rs4644481 | ALM | Two-hour glucose challenge | -0.0112 | 155130900 | 0.0019 | 0.024 | 0.019 | 0.2058 |
| rs4655345 | ALM | Two-hour glucose challenge | -0.0246 | 214608704 | 0.0019 | 0.019 | 0.019 | 0.3134 |
| rs4682483 | ALM | Two-hour glucose challenge | -0.0165 | 112993982 | 0.0026 | 0.013 | 0.023 | 0.5919 |
| rs4683435 | ALM | Two-hour glucose challenge | 0.0144 | 142624732 | 0.0022 | 0.024 | 0.023 | 0.2957 |
| rs4735761 | ALM | Two-hour glucose challenge | 0.0331 | 78097161 | 0.0021 | 0.006 | 0.021 | 0.781801 |
| rs4748008 | ALM | Two-hour glucose challenge | -0.0125 | 12935125 | 0.0019 | -0.0036 | 0.019 | 0.8514 |
| rs4752689 | ALM | Two-hour glucose challenge | 0.0205 | 124131176 | 0.0019 | -0.02 | 0.019 | 0.2957 |
| rs4752829 | ALM | Two-hour glucose challenge | 0.0262 | 47396654 | 0.0021 | -0.0075 | 0.02 | 0.708699 |
| rs4788218 | ALM | Two-hour glucose challenge | 0.0275 | 30055750 | 0.0019 | 0.027 | 0.019 | 0.1656 |
| rs4807472 | ALM | Two-hour glucose challenge | -0.0158 | 3448842 | 0.002 | 0.029 | 0.02 | 0.1557 |
| rs4815952 | ALM | Two-hour glucose challenge | -0.0161 | 6934897 | 0.0019 | -0.019 | 0.018 | 0.3081 |
| rs4818280 | ALM | Two-hour glucose challenge | -0.0124 | 18114472 | 0.002 | -0.017 | 0.019 | 0.3931 |
| rs4847378 | ALM | Two-hour glucose challenge | 0.0136 | 93324634 | 0.0019 | 0.015 | 0.019 | 0.4453 |
| rs4852257 | ALM | Two-hour glucose challenge | -0.0231 | 71678520 | 0.0019 | -0.0049 | 0.019 | 0.7994 |
| rs4865956 | ALM | Two-hour glucose challenge | -0.0258 | 54882505 | 0.0021 | 0.018 | 0.02 | 0.3669 |
| rs4870941 | ALM | Two-hour glucose challenge | -0.0297 | 126498828 | 0.0023 | -0.054 | 0.071 | 0.4437 |
| rs488621 | ALM | Two-hour glucose challenge | 0.0191 | 169707552 | 0.0019 | -0.0067 | 0.019 | 0.723501 |
| rs4900578 | ALM | Two-hour glucose challenge | -0.0177 | 103926010 | 0.002 | -0.0045 | 0.019 | 0.8183 |
| rs4932439 | ALM | Two-hour glucose challenge | -0.0151 | 89401109 | 0.0025 | 0.027 | 0.023 | 0.2464 |
| rs4938359 | ALM | Two-hour glucose challenge | -0.0156 | 117093560 | 0.0024 | 0.0048 | 0.022 | 0.8303 |
| rs4940874 | ALM | Two-hour glucose challenge | 0.0148 | 57105638 | 0.0024 | 0.0023 | 0.023 | 0.9206 |
| rs4965298 | ALM | Two-hour glucose challenge | -0.0119 | 100802766 | 0.0021 | -0.0088 | 0.022 | 0.6902 |
| rs496783 | ALM | Two-hour glucose challenge | -0.0124 | 116137961 | 0.0019 | 0.009 | 0.019 | 0.6383 |
| rs4985445 | ALM | Two-hour glucose challenge | -0.0175 | 69867835 | 0.0019 | 0.02 | 0.019 | 0.2739 |
| rs543650 | ALM | Two-hour glucose challenge | 0.025 | 152110943 | 0.002 | -0.043 | 0.024 | 0.0717893 |
| rs55872725 | ALM | Two-hour glucose challenge | 0.0222 | 53809123 | 0.0019 | 0.0053 | 0.019 | 0.777401 |
| rs568267 | ALM | Two-hour glucose challenge | 0.0122 | 8799828 | 0.0022 | -0.05 | 0.022 | 0.0221901 |
| rs57059662 | ALM | Two-hour glucose challenge | 0.0118 | 33217275 | 0.002 | -0.0017 | 0.02 | 0.9344 |
| rs5742915 | ALM | Two-hour glucose challenge | 0.0248 | 74336633 | 0.0019 | -0.02 | 0.021 | 0.3482 |
| rs5753518 | ALM | Two-hour glucose challenge | 0.0242 | 31631314 | 0.0033 | -0.015 | 0.034 | 0.6693 |
| rs5763821 | ALM | Two-hour glucose challenge | 0.0191 | 30549071 | 0.002 | -0.012 | 0.019 | 0.5043 |
| rs577289 | ALM | Two-hour glucose challenge | -0.0125 | 40208911 | 0.0021 | 0.0057 | 0.021 | 0.7878 |
| rs57791149 | ALM | Two-hour glucose challenge | -0.0173 | 54222307 | 0.0019 | -0.0037 | 0.019 | 0.847 |
| rs591668 | ALM | Two-hour glucose challenge | -0.0174 | 27535931 | 0.0019 | -0.028 | 0.019 | 0.135 |
| rs599004 | ALM | Two-hour glucose challenge | -0.0157 | 140439740 | 0.0021 | 0.043 | 0.022 | 0.0517595 |
| rs59985551 | ALM | Two-hour glucose challenge | -0.0313 | 56106928 | 0.0022 | 0.0029 | 0.021 | 0.8919 |
| rs6028716 | ALM | Two-hour glucose challenge | -0.021 | 38547459 | 0.0022 | 0.021 | 0.022 | 0.3448 |
| rs604723 | ALM | Two-hour glucose challenge | -0.0166 | 100610546 | 0.0021 | -0.0008 | 0.021 | 0.9691 |
| rs6054390 | ALM | Two-hour glucose challenge | -0.0188 | 6592094 | 0.002 | 0.0022 | 0.019 | 0.9097 |
| rs6066122 | ALM | Two-hour glucose challenge | 0.0127 | 45558573 | 0.0023 | 0.015 | 0.023 | 0.5081 |
| rs60804050 | ALM | Two-hour glucose challenge | -0.0217 | 118870373 | 0.0021 | -0.0004 | 0.022 | 0.986 |
| rs6082354 | ALM | Two-hour glucose challenge | -0.024 | 21217976 | 0.002 | 0.0063 | 0.02 | 0.751299 |
| rs610694 | ALM | Two-hour glucose challenge | 0.0136 | 121304826 | 0.0019 | -0.008 | 0.018 | 0.667799 |
| rs61397287 | ALM | Two-hour glucose challenge | 0.0235 | 144223279 | 0.0036 | -0.02 | 0.035 | 0.5622 |
| rs6142059 | ALM | Two-hour glucose challenge | 0.0116 | 32544327 | 0.0019 | 0.022 | 0.019 | 0.2451 |
| rs61528919 | ALM | Two-hour glucose challenge | 0.014 | 1004909 | 0.002 | -0.009 | 0.019 | 0.638 |
| rs62033029 | ALM | Two-hour glucose challenge | -0.0141 | 50107273 | 0.0023 | 0.0023 | 0.023 | 0.919 |
| rs62070319 | ALM | Two-hour glucose challenge | -0.018 | 89573216 | 0.002 | -0.0083 | 0.019 | 0.653899 |
| rs62515437 | ALM | Two-hour glucose challenge | 0.0369 | 57160328 | 0.0023 | 0.012 | 0.022 | 0.593401 |
| rs6425817 | ALM | Two-hour glucose challenge | 0.0157 | 33873034 | 0.002 | 0.035 | 0.02 | 0.0760309 |
| rs6470771 | ALM | Two-hour glucose challenge | -0.0268 | 130743726 | 0.0025 | 0.004 | 0.025 | 0.8745 |
| rs6502935 | ALM | Two-hour glucose challenge | -0.0125 | 1650168 | 0.0022 | 0.011 | 0.035 | 0.760599 |
| rs650508 | ALM | Two-hour glucose challenge | -0.013 | 45880122 | 0.002 | 0.025 | 0.021 | 0.2408 |
| rs6543146 | ALM | Two-hour glucose challenge | 0.0154 | 103096695 | 0.0019 | -0.0018 | 0.019 | 0.9246 |
| rs6570509 | ALM | Two-hour glucose challenge | -0.0244 | 142716286 | 0.0021 | 0.012 | 0.021 | 0.5858 |
| rs6582398 | ALM | Two-hour glucose challenge | 0.014 | 42870444 | 0.002 | -0.039 | 0.02 | 0.0492697 |
| rs6593210 | ALM | Two-hour glucose challenge | 0.0146 | 55254186 | 0.0024 | -0.024 | 0.023 | 0.3014 |
| rs664317 | ALM | Two-hour glucose challenge | -0.0177 | 89812230 | 0.0026 | -0.0037 | 0.025 | 0.8828 |
| rs6675858 | ALM | Two-hour glucose challenge | -0.0137 | 224559936 | 0.0023 | -0.0069 | 0.022 | 0.7552 |
| rs6693481 | ALM | Two-hour glucose challenge | -0.0143 | 203766395 | 0.002 | -0.027 | 0.02 | 0.1815 |
| rs670318 | ALM | Two-hour glucose challenge | 0.0413 | 63727542 | 0.0044 | 0.008 | 0.05 | 0.8735 |
| rs6738207 | ALM | Two-hour glucose challenge | 0.0127 | 105989716 | 0.0019 | 0.046 | 0.019 | 0.0176498 |
| rs6739278 | ALM | Two-hour glucose challenge | -0.021 | 44401055 | 0.0024 | -0.0062 | 0.023 | 0.7896 |
| rs67527161 | ALM | Two-hour glucose challenge | -0.0182 | 63781824 | 0.0023 | 0.0074 | 0.025 | 0.7681 |
| rs6762851 | ALM | Two-hour glucose challenge | -0.0218 | 56686329 | 0.002 | 0.0009 | 0.019 | 0.9641 |
| rs67716382 | ALM | Two-hour glucose challenge | 0.0226 | 46890317 | 0.0023 | -0.006 | 0.024 | 0.8056 |
| rs6789000 | ALM | Two-hour glucose challenge | 0.0121 | 25188002 | 0.002 | -0.0013 | 0.019 | 0.9476 |
| rs680882 | ALM | Two-hour glucose challenge | 0.0133 | 18325278 | 0.0022 | -0.039 | 0.023 | 0.0926894 |
| rs684905 | ALM | Two-hour glucose challenge | -0.0118 | 10472790 | 0.0019 | 0.0013 | 0.019 | 0.9448 |
| rs6849302 | ALM | Two-hour glucose challenge | 0.0155 | 156665074 | 0.0024 | 0.0006 | 0.023 | 0.9799 |
| rs6860245 | ALM | Two-hour glucose challenge | 0.0589 | 127367998 | 0.0022 | 0.0008 | 0.023 | 0.9714 |
| rs6902109 | ALM | Two-hour glucose challenge | -0.0167 | 130316559 | 0.0019 | 0.018 | 0.019 | 0.3353 |
| rs6931421 | ALM | Two-hour glucose challenge | -0.0279 | 80880138 | 0.002 | 0.012 | 0.021 | 0.5587 |
| rs700677 | ALM | Two-hour glucose challenge | 0.0173 | 198702424 | 0.002 | -0.005 | 0.019 | 0.7977 |
| rs7014590 | ALM | Two-hour glucose challenge | -0.0228 | 89335647 | 0.0022 | -0.036 | 0.02 | 0.0688304 |
| rs7020491 | ALM | Two-hour glucose challenge | -0.0178 | 128144477 | 0.0019 | -0.0017 | 0.019 | 0.9272 |
| rs702886 | ALM | Two-hour glucose challenge | 0.012 | 65753310 | 0.002 | 0.0081 | 0.019 | 0.6724 |
| rs704660 | ALM | Two-hour glucose challenge | 0.0153 | 30447998 | 0.0019 | 0.033 | 0.019 | 0.0820106 |
| rs7082659 | ALM | Two-hour glucose challenge | 0.0156 | 12017584 | 0.0028 | 0.038 | 0.027 | 0.1669 |
| rs713467 | ALM | Two-hour glucose challenge | 0.0146 | 84646473 | 0.0019 | -0.012 | 0.018 | 0.5223 |
| rs7137546 | ALM | Two-hour glucose challenge | 0.0142 | 577237 | 0.0019 | 0.019 | 0.019 | 0.326 |
| rs71414738 | ALM | Two-hour glucose challenge | 0.015 | 127876242 | 0.0025 | -0.016 | 0.027 | 0.5524 |
| rs7144307 | ALM | Two-hour glucose challenge | -0.0122 | 69533837 | 0.002 | 0.011 | 0.019 | 0.581599 |
| rs7185244 | ALM | Two-hour glucose challenge | -0.0148 | 86546887 | 0.0023 | -0.019 | 0.023 | 0.4168 |
| rs718603 | ALM | Two-hour glucose challenge | 0.0131 | 2644245 | 0.0021 | 0.0039 | 0.021 | 0.8553 |
| rs7220127 | ALM | Two-hour glucose challenge | -0.0105 | 64545922 | 0.0019 | -0.0062 | 0.019 | 0.742899 |
| rs7229520 | ALM | Two-hour glucose challenge | -0.0224 | 46516468 | 0.002 | -0.0027 | 0.021 | 0.897 |
| rs723149 | ALM | Two-hour glucose challenge | -0.0276 | 46577056 | 0.0019 | 0.032 | 0.021 | 0.1239 |
| rs72657800 | ALM | Two-hour glucose challenge | -0.0219 | 90822051 | 0.0035 | 0.0002 | 0.036 | 0.9957 |
| rs72695791 | ALM | Two-hour glucose challenge | -0.0297 | 184059452 | 0.0051 | 0.0067 | 0.052 | 0.897 |
| rs72801843 | ALM | Two-hour glucose challenge | 0.0313 | 53508802 | 0.0021 | -0.033 | 0.02 | 0.0969103 |
| rs72809820 | ALM | Two-hour glucose challenge | -0.0111 | 97360079 | 0.002 | -0.019 | 0.019 | 0.3396 |
| rs73006226 | ALM | Two-hour glucose challenge | -0.0182 | 108072728 | 0.0029 | -0.03 | 0.025 | 0.2304 |
| rs7301341 | ALM | Two-hour glucose challenge | -0.0255 | 94083105 | 0.002 | 0.0005 | 0.02 | 0.9798 |
| rs73052033 | ALM | Two-hour glucose challenge | -0.0151 | 185828465 | 0.0024 | 0.0099 | 0.025 | 0.6911 |
| rs7320878 | ALM | Two-hour glucose challenge | -0.015 | 91994132 | 0.0019 | 0.011 | 0.019 | 0.548399 |
| rs7328187 | ALM | Two-hour glucose challenge | 0.0116 | 74189974 | 0.0019 | -0.022 | 0.019 | 0.2632 |
| rs73384223 | ALM | Two-hour glucose challenge | -0.0205 | 3869315 | 0.0024 | -0.0081 | 0.024 | 0.733 |
| rs7367519 | ALM | Two-hour glucose challenge | 0.0164 | 204479176 | 0.002 | -0.015 | 0.021 | 0.4747 |
| rs73856768 | ALM | Two-hour glucose challenge | -0.0247 | 157788804 | 0.0035 | -0.052 | 0.035 | 0.1445 |
| rs7418410 | ALM | Two-hour glucose challenge | 0.0155 | 10236402 | 0.0019 | 0.01 | 0.019 | 0.5978 |
| rs74379684 | ALM | Two-hour glucose challenge | -0.0272 | 94050205 | 0.0036 | 0.042 | 0.034 | 0.2205 |
| rs74494415 | ALM | Two-hour glucose challenge | -0.0417 | 74972138 | 0.0049 | -0.042 | 0.041 | 0.3118 |
| rs7485647 | ALM | Two-hour glucose challenge | -0.0261 | 131631133 | 0.0026 | 0.029 | 0.024 | 0.2302 |
| rs7522400 | ALM | Two-hour glucose challenge | 0.0129 | 36613380 | 0.0022 | 0.0007 | 0.021 | 0.9755 |
| rs7543202 | ALM | Two-hour glucose challenge | 0.0129 | 73872885 | 0.0019 | -0.022 | 0.019 | 0.2484 |
| rs757834 | ALM | Two-hour glucose challenge | 0.0256 | 139717200 | 0.0024 | 0.036 | 0.022 | 0.1077 |
| rs7598430 | ALM | Two-hour glucose challenge | -0.016 | 219193963 | 0.0019 | 0.0078 | 0.019 | 0.6911 |
| rs7610055 | ALM | Two-hour glucose challenge | -0.0373 | 12388409 | 0.0029 | -0.084 | 0.027 | 0.00172001 |
| rs7633464 | ALM | Two-hour glucose challenge | 0.0175 | 98715823 | 0.0019 | -0.013 | 0.018 | 0.4795 |
| rs76517946 | ALM | Two-hour glucose challenge | -0.0368 | 68354936 | 0.0035 | -0.023 | 0.04 | 0.5679 |
| rs7679276 | ALM | Two-hour glucose challenge | -0.033 | 146860186 | 0.0048 | -0.027 | 0.099 | 0.788199 |
| rs7689420 | ALM | Two-hour glucose challenge | 0.0466 | 145568352 | 0.0025 | -0.0016 | 0.025 | 0.95 |
| rs7701233 | ALM | Two-hour glucose challenge | -0.0179 | 171218388 | 0.0019 | 0.038 | 0.019 | 0.0512 |
| rs772222 | ALM | Two-hour glucose challenge | 0.0121 | 52356892 | 0.0021 | 0.034 | 0.021 | 0.1068 |
| rs7731023 | ALM | Two-hour glucose challenge | 0.0166 | 36181627 | 0.0019 | 0.022 | 0.02 | 0.2723 |
| rs7735891 | ALM | Two-hour glucose challenge | 0.0259 | 131597005 | 0.0019 | -0.011 | 0.019 | 0.5425 |
| rs77447813 | ALM | Two-hour glucose challenge | 0.0224 | 50827041 | 0.0034 | 0.0011 | 0.034 | 0.9751 |
| rs7768973 | ALM | Two-hour glucose challenge | -0.024 | 109745325 | 0.0019 | 0.028 | 0.019 | 0.1586 |
| rs77809369 | ALM | Two-hour glucose challenge | 0.0237 | 9052448 | 0.0039 | 0.0014 | 0.039 | 0.9709 |
| rs7816345 | ALM | Two-hour glucose challenge | 0.0255 | 36846109 | 0.0025 | -0.027 | 0.025 | 0.2676 |
| rs7826059 | ALM | Two-hour glucose challenge | 0.0114 | 22512068 | 0.002 | -0.014 | 0.019 | 0.4732 |
| rs7828086 | ALM | Two-hour glucose challenge | 0.0135 | 120843775 | 0.0022 | -0.0024 | 0.021 | 0.9108 |
| rs7858712 | ALM | Two-hour glucose challenge | 0.0347 | 16738312 | 0.0034 | -0.012 | 0.034 | 0.727601 |
| rs7863102 | ALM | Two-hour glucose challenge | -0.011 | 73963468 | 0.0019 | 0.021 | 0.019 | 0.2694 |
| rs7902 | ALM | Two-hour glucose challenge | 0.0149 | 95565288 | 0.0019 | -0.012 | 0.019 | 0.5317 |
| rs79066296 | ALM | Two-hour glucose challenge | -0.0169 | 76391462 | 0.0022 | 0.0049 | 0.022 | 0.8259 |
| rs7971536 | ALM | Two-hour glucose challenge | -0.0194 | 102373788 | 0.0019 | -0.014 | 0.019 | 0.4776 |
| rs798548 | ALM | Two-hour glucose challenge | -0.0359 | 2760935 | 0.0021 | -0.011 | 0.02 | 0.5814 |
| rs8000973 | ALM | Two-hour glucose challenge | 0.0134 | 100691367 | 0.0019 | -0.0037 | 0.019 | 0.8438 |
| rs8017006 | ALM | Two-hour glucose challenge | 0.0122 | 42745052 | 0.002 | 0.011 | 0.021 | 0.593999 |
| rs8018486 | ALM | Two-hour glucose challenge | -0.0138 | 39818616 | 0.0024 | -0.014 | 0.023 | 0.5364 |
| rs8019890 | ALM | Two-hour glucose challenge | 0.025 | 21538067 | 0.0019 | -0.029 | 0.022 | 0.1847 |
| rs8020095 | ALM | Two-hour glucose challenge | -0.0145 | 67453858 | 0.0027 | 0.0045 | 0.026 | 0.865 |
| rs80295797 | ALM | Two-hour glucose challenge | -0.0198 | 23341690 | 0.002 | 0.0025 | 0.019 | 0.8955 |
| rs8042578 | ALM | Two-hour glucose challenge | 0.0287 | 66992964 | 0.0022 | -0.015 | 0.022 | 0.485 |
| rs8054549 | ALM | Two-hour glucose challenge | -0.0251 | 86417234 | 0.0019 | -0.011 | 0.019 | 0.5664 |
| rs8084413 | ALM | Two-hour glucose challenge | -0.0127 | 22869123 | 0.0019 | -0.013 | 0.02 | 0.5322 |
| rs8136517 | ALM | Two-hour glucose challenge | 0.0267 | 46439433 | 0.0039 | -0.057 | 0.049 | 0.245 |
| rs839255 | ALM | Two-hour glucose challenge | -0.0126 | 57974580 | 0.0021 | 0.017 | 0.02 | 0.3916 |
| rs861674 | ALM | Two-hour glucose challenge | 0.0128 | 112064475 | 0.0019 | -0.0003 | 0.019 | 0.9868 |
| rs867529 | ALM | Two-hour glucose challenge | 0.0184 | 88913273 | 0.0021 | -0.01 | 0.02 | 0.605701 |
| rs876122 | ALM | Two-hour glucose challenge | 0.0162 | 6886297 | 0.0029 | -0.0017 | 0.034 | 0.9606 |
| rs900399 | ALM | Two-hour glucose challenge | 0.0164 | 156798732 | 0.0019 | -0.029 | 0.02 | 0.1561 |
| rs905938 | ALM | Two-hour glucose challenge | 0.0394 | 154991389 | 0.0021 | -0.0067 | 0.023 | 0.7743 |
| rs909220 | ALM | Two-hour glucose challenge | -0.015 | 75908780 | 0.0019 | -0.028 | 0.019 | 0.1407 |
| rs9343327 | ALM | Two-hour glucose challenge | 0.014 | 76606296 | 0.0019 | -0.022 | 0.019 | 0.2471 |
| rs9344126 | ALM | Two-hour glucose challenge | -0.0185 | 81907559 | 0.0019 | 0.016 | 0.02 | 0.4245 |
| rs9375188 | ALM | Two-hour glucose challenge | 0.0136 | 98555272 | 0.0019 | 0.017 | 0.02 | 0.3996 |
| rs9385002 | ALM | Two-hour glucose challenge | -0.0147 | 117552469 | 0.0022 | 0.0055 | 0.021 | 0.7888 |
| rs9388490 | ALM | Two-hour glucose challenge | 0.0462 | 126704795 | 0.0019 | 0.013 | 0.019 | 0.5025 |
| rs9391254 | ALM | Two-hour glucose challenge | 0.0166 | 105377347 | 0.002 | 0.0049 | 0.02 | 0.809 |
| rs947099 | ALM | Two-hour glucose challenge | 0.0117 | 31129883 | 0.002 | -0.016 | 0.02 | 0.4242 |
| rs951366 | ALM | Two-hour glucose challenge | 0.0205 | 205685352 | 0.0019 | -0.02 | 0.019 | 0.2883 |
| rs9517483 | ALM | Two-hour glucose challenge | -0.0181 | 99572712 | 0.0021 | 0.002 | 0.02 | 0.9199 |
| rs9568031 | ALM | Two-hour glucose challenge | -0.0115 | 48897520 | 0.0021 | -0.016 | 0.021 | 0.438 |
| rs9590328 | ALM | Two-hour glucose challenge | 0.0153 | 96448383 | 0.0027 | -0.001 | 0.026 | 0.9703 |
| rs9594714 | ALM | Two-hour glucose challenge | 0.0144 | 42800481 | 0.0021 | -0.008 | 0.021 | 0.7085 |
| rs9647379 | ALM | Two-hour glucose challenge | 0.0215 | 171785168 | 0.0019 | 0.0077 | 0.021 | 0.714801 |
| rs9828525 | ALM | Two-hour glucose challenge | 0.0121 | 61552810 | 0.0019 | 0.025 | 0.019 | 0.1957 |
| rs9838614 | ALM | Two-hour glucose challenge | -0.0185 | 38537671 | 0.0019 | -0.021 | 0.019 | 0.2565 |
| rs987666 | ALM | Two-hour glucose challenge | 0.0185 | 116267938 | 0.0029 | -0.024 | 0.028 | 0.3982 |
| rs9890062 | ALM | Two-hour glucose challenge | 0.0267 | 17434352 | 0.0039 | 0.03 | 0.032 | 0.3397 |
| rs9898189 | ALM | Two-hour glucose challenge | -0.0163 | 80480516 | 0.0021 | -0.0029 | 0.021 | 0.8905 |
| rs990315 | ALM | Two-hour glucose challenge | -0.0115 | 69578811 | 0.002 | 0.028 | 0.019 | 0.1417 |
| rs9905385 | ALM | Two-hour glucose challenge | -0.0339 | 59498250 | 0.002 | 0.024 | 0.02 | 0.2279 |
| rs9957318 | ALM | Two-hour glucose challenge | 0.0187 | 33039106 | 0.002 | -0.019 | 0.021 | 0.3736 |
| rs10005035 | ALM | Type 2 diabetes | -0.0175 | 12865684 | 0.0021 | 0.007 | 0.0071 | 0.3255 |
| rs10019221 | ALM | Type 2 diabetes | -0.0124 | 21785364 | 0.0019 | 0.0048 | 0.0066 | 0.4668 |
| rs10036789 | ALM | Type 2 diabetes | 0.0163 | 71695918 | 0.0019 | 0.0017 | 0.0064 | 0.7901 |
| rs1005723 | ALM | Type 2 diabetes | 0.0161 | 243646251 | 0.0024 | -0.0181 | 0.0082 | 0.02667 |
| rs10068640 | ALM | Type 2 diabetes | 0.0112 | 123981977 | 0.002 | 0.0039 | 0.0066 | 0.5544 |
| rs10075249 | ALM | Type 2 diabetes | 0.0143 | 52846505 | 0.0019 | -0.0129 | 0.0064 | 0.04341 |
| rs10107388 | ALM | Type 2 diabetes | -0.0159 | 145004944 | 0.002 | -0.0182 | 0.0068 | 0.00749 |
| rs10112506 | ALM | Type 2 diabetes | -0.012 | 13164746 | 0.0019 | -0.0089 | 0.0066 | 0.1773 |
| rs10123619 | ALM | Type 2 diabetes | -0.0171 | 119353611 | 0.0026 | -0.0007 | 0.0087 | 0.9358 |
| rs10128333 | ALM | Type 2 diabetes | -0.0146 | 64570038 | 0.0025 | 0.021 | 0.0085 | 0.01328 |
| rs10202701 | ALM | Type 2 diabetes | 0.0227 | 232328681 | 0.0019 | -0.0083 | 0.0065 | 0.201 |
| rs10202845 | ALM | Type 2 diabetes | -0.0288 | 42575820 | 0.003 | -0.0291 | 0.0101 | 0.00379 |
| rs10203320 | ALM | Type 2 diabetes | 0.0138 | 9771620 | 0.002 | -0.0031 | 0.0068 | 0.6487 |
| rs10203386 | ALM | Type 2 diabetes | -0.0238 | 25136866 | 0.0019 | 0.006 | 0.0063 | 0.3395 |
| rs10205141 | ALM | Type 2 diabetes | 0.0241 | 11313340 | 0.0044 | -0.0193 | 0.0135 | 0.153 |
| rs10221831 | ALM | Type 2 diabetes | 0.03 | 202107829 | 0.0053 | -0.0124 | 0.0174 | 0.4756 |
| rs10225945 | ALM | Type 2 diabetes | -0.0146 | 28250083 | 0.0026 | 0.0474 | 0.0087 | 4.915E-08 |
| rs10242866 | ALM | Type 2 diabetes | 0.0157 | 17920613 | 0.0019 | 0.0017 | 0.0065 | 0.7934 |
| rs10283100 | ALM | Type 2 diabetes | 0.0575 | 120596023 | 0.0041 | -0.0122 | 0.0138 | 0.3774 |
| rs1035583 | ALM | Type 2 diabetes | 0.0148 | 207326937 | 0.0019 | 0.0004 | 0.0065 | 0.9509 |
| rs10453441 | ALM | Type 2 diabetes | -0.0139 | 46363739 | 0.002 | -0.0011 | 0.007 | 0.8754 |
| rs10461725 | ALM | Type 2 diabetes | 0.0134 | 39437129 | 0.002 | -0.0088 | 0.0067 | 0.1891 |
| rs10471339 | ALM | Type 2 diabetes | -0.011 | 67823773 | 0.0019 | 0.0072 | 0.0067 | 0.2826 |
| rs1047891 | ALM | Type 2 diabetes | 0.0233 | 211540507 | 0.002 | -0.0059 | 0.0069 | 0.3932 |
| rs10483727 | ALM | Type 2 diabetes | -0.0368 | 61072875 | 0.0019 | 0.0186 | 0.0065 | 0.004167 |
| rs1056747 | ALM | Type 2 diabetes | -0.0155 | 35690102 | 0.0019 | -0.0049 | 0.0063 | 0.4354 |
| rs1063582 | ALM | Type 2 diabetes | -0.0185 | 23167353 | 0.0022 | -0.0215 | 0.0076 | 0.004909 |
| rs10748128 | ALM | Type 2 diabetes | 0.0255 | 69827658 | 0.002 | 0.0039 | 0.0067 | 0.5606 |
| rs10749157 | ALM | Type 2 diabetes | 0.0113 | 115780129 | 0.002 | -0.0098 | 0.0066 | 0.1374 |
| rs10776560 | ALM | Type 2 diabetes | -0.0157 | 50542358 | 0.0019 | -0.0022 | 0.0064 | 0.7305 |
| rs10793931 | ALM | Type 2 diabetes | -0.0132 | 133436478 | 0.002 | -0.0061 | 0.0067 | 0.3627 |
| rs10796828 | ALM | Type 2 diabetes | 0.0154 | 69490346 | 0.002 | 0.0145 | 0.0071 | 0.04169 |
| rs10807137 | ALM | Type 2 diabetes | -0.0455 | 34183026 | 0.0025 | -0.0033 | 0.0085 | 0.6972 |
| rs10815274 | ALM | Type 2 diabetes | 0.0124 | 5728968 | 0.0019 | -0.0014 | 0.0064 | 0.8265 |
| rs10822117 | ALM | Type 2 diabetes | -0.0176 | 52786701 | 0.0022 | -0.0126 | 0.0075 | 0.09464 |
| rs10824307 | ALM | Type 2 diabetes | -0.0194 | 77185310 | 0.002 | -0.0183 | 0.0068 | 0.007168 |
| rs10829226 | ALM | Type 2 diabetes | -0.0112 | 27573952 | 0.002 | -0.0096 | 0.0067 | 0.152 |
| rs10832963 | ALM | Type 2 diabetes | -0.0203 | 18664241 | 0.0022 | -0.0088 | 0.0072 | 0.2232 |
| rs10845408 | ALM | Type 2 diabetes | 0.0255 | 11880581 | 0.002 | 0.0002 | 0.0067 | 0.9762 |
| rs10858246 | ALM | Type 2 diabetes | -0.0188 | 139102831 | 0.002 | 0.0101 | 0.0072 | 0.1621 |
| rs10922475 | ALM | Type 2 diabetes | 0.0159 | 89142142 | 0.0019 | -0.0161 | 0.0064 | 0.01171 |
| rs10948 | ALM | Type 2 diabetes | -0.0252 | 10754905 | 0.002 | 0.0007 | 0.0069 | 0.9193 |
| rs10962212 | ALM | Type 2 diabetes | 0.0143 | 15911745 | 0.0019 | -0.0103 | 0.0065 | 0.1126 |
| rs10975935 | ALM | Type 2 diabetes | -0.0121 | 6954579 | 0.0022 | -0.0078 | 0.0072 | 0.2803 |
| rs10982888 | ALM | Type 2 diabetes | -0.0328 | 118468947 | 0.003 | -0.0184 | 0.0101 | 0.06716 |
| rs11014285 | ALM | Type 2 diabetes | 0.0342 | 25178864 | 0.0026 | -0.0171 | 0.009 | 0.05756 |
| rs11042717 | ALM | Type 2 diabetes | -0.029 | 10303939 | 0.0019 | 0.0137 | 0.0064 | 0.03195 |
| rs11060942 | ALM | Type 2 diabetes | -0.0354 | 123434524 | 0.0052 | 0.0182 | 0.0178 | 0.3065 |
| rs11068230 | ALM | Type 2 diabetes | 0.0238 | 117349014 | 0.0028 | -0.0133 | 0.0092 | 0.1489 |
| rs11070842 | ALM | Type 2 diabetes | -0.0146 | 51624185 | 0.0026 | 0.0049 | 0.0087 | 0.5729 |
| rs11098677 | ALM | Type 2 diabetes | -0.0263 | 123833516 | 0.0023 | 0.0293 | 0.0077 | 0.0001558 |
| rs11121615 | ALM | Type 2 diabetes | -0.0202 | 10825577 | 0.002 | 0.0014 | 0.007 | 0.8418 |
| rs111365325 | ALM | Type 2 diabetes | -0.0271 | 170865229 | 0.0022 | -0.0044 | 0.0076 | 0.5648 |
| rs111622870 | ALM | Type 2 diabetes | -0.0282 | 2613109 | 0.0044 | 0.009 | 0.0159 | 0.5717 |
| rs11175919 | ALM | Type 2 diabetes | 0.0349 | 66180277 | 0.0059 | 0.0537 | 0.0201 | 0.007557 |
| rs11187838 | ALM | Type 2 diabetes | 0.0394 | 96038686 | 0.0019 | -0.0026 | 0.0064 | 0.6839 |
| rs11191208 | ALM | Type 2 diabetes | 0.0147 | 103838497 | 0.0024 | -0.0208 | 0.008 | 0.008951 |
| rs11198591 | ALM | Type 2 diabetes | 0.0148 | 120515892 | 0.002 | -0.008 | 0.0066 | 0.2252 |
| rs11210892 | ALM | Type 2 diabetes | 0.0118 | 44100084 | 0.002 | 0.0061 | 0.0068 | 0.3701 |
| rs112153300 | ALM | Type 2 diabetes | 0.0261 | 47547474 | 0.0034 | 0.0366 | 0.0116 | 0.001637 |
| rs11217863 | ALM | Type 2 diabetes | -0.0268 | 120293138 | 0.003 | 0.0029 | 0.0099 | 0.7706 |
| rs11221657 | ALM | Type 2 diabetes | 0.0179 | 129181358 | 0.0028 | 0.0098 | 0.0092 | 0.2875 |
| rs11233117 | ALM | Type 2 diabetes | -0.0176 | 69924352 | 0.0019 | 0.0105 | 0.0065 | 0.1058 |
| rs11243202 | ALM | Type 2 diabetes | 0.0302 | 7719065 | 0.0019 | -0.0012 | 0.0064 | 0.851 |
| rs112537273 | ALM | Type 2 diabetes | -0.0212 | 38248306 | 0.0022 | 0.0012 | 0.0076 | 0.8752 |
| rs11260035 | ALM | Type 2 diabetes | 0.015 | 7898957 | 0.0021 | 0.0331 | 0.0072 | 0.000004613 |
| rs112873218 | ALM | Type 2 diabetes | 0.0216 | 1960119 | 0.0031 | 0.0057 | 0.0107 | 0.5935 |
| rs113146332 | ALM | Type 2 diabetes | 0.0311 | 42565977 | 0.0049 | 0.0227 | 0.0178 | 0.2022 |
| rs113671109 | ALM | Type 2 diabetes | -0.015 | 12620885 | 0.0023 | -0.0187 | 0.0077 | 0.0158 |
| rs113827862 | ALM | Type 2 diabetes | -0.0235 | 89849527 | 0.004 | 0.015 | 0.0133 | 2.59E-01 |
| rs11580040 | ALM | Type 2 diabetes | 0.0325 | 155198222 | 0.0035 | 0.0001 | 0.012 | 0.9934 |
| rs11590254 | ALM | Type 2 diabetes | 0.0186 | 92316573 | 0.002 | 0.0064 | 0.0069 | 0.3544 |
| rs115912456 | ALM | Type 2 diabetes | 0.0577 | 82815158 | 0.0047 | -0.0326 | 0.016 | 0.04185 |
| rs116008080 | ALM | Type 2 diabetes | -0.0415 | 67254841 | 0.0063 | 0.0226 | 0.021 | 0.2829 |
| rs116052377 | ALM | Type 2 diabetes | 0.0225 | 124787756 | 0.0035 | -0.0118 | 0.0123 | 0.3354 |
| rs11605297 | ALM | Type 2 diabetes | 0.0146 | 58296806 | 0.0022 | -0.0251 | 0.0075 | 0.0008699 |
| rs11612462 | ALM | Type 2 diabetes | 0.015 | 104411368 | 0.0025 | 0.0023 | 0.0085 | 0.7862 |
| rs11633371 | ALM | Type 2 diabetes | 0.0216 | 89356832 | 0.0019 | -0.004 | 0.0064 | 0.5311 |
| rs116339650 | ALM | Type 2 diabetes | -0.0175 | 26200972 | 0.0029 | 0.0214 | 0.0095 | 0.0247 |
| rs116493405 | ALM | Type 2 diabetes | 0.0287 | 114733556 | 0.0042 | -0.0386 | 0.0141 | 0.006317 |
| rs11684531 | ALM | Type 2 diabetes | -0.0172 | 219835489 | 0.0028 | -0.0012 | 0.0108 | 0.9114 |
| rs1168768 | ALM | Type 2 diabetes | 0.0332 | 66509650 | 0.006 | -0.0115 | 0.0217 | 0.5957 |
| rs116919274 | ALM | Type 2 diabetes | 0.0271 | 17359808 | 0.0046 | 0.0023 | 0.0162 | 0.8873 |
| rs117068593 | ALM | Type 2 diabetes | 0.0403 | 93118229 | 0.0024 | -0.0062 | 0.0086 | 0.4702 |
| rs117203652 | ALM | Type 2 diabetes | -0.0346 | 49857801 | 0.0055 | 0.0044 | 0.0207 | 0.8319 |
| rs11720869 | ALM | Type 2 diabetes | 0.0141 | 185619716 | 0.002 | -0.0103 | 0.0068 | 0.1302 |
| rs11721522 | ALM | Type 2 diabetes | 0.0106 | 156976051 | 0.0019 | -0.002 | 0.0065 | 0.758 |
| rs11727162 | ALM | Type 2 diabetes | -0.017 | 88606761 | 0.0019 | -0.0061 | 0.0064 | 0.3395 |
| rs117335233 | ALM | Type 2 diabetes | -0.0236 | 79914330 | 0.0042 | 0.0097 | 0.015 | 0.5171 |
| rs1177765 | ALM | Type 2 diabetes | -0.0232 | 32829929 | 0.0019 | -0.0022 | 0.0064 | 0.7305 |
| rs11778491 | ALM | Type 2 diabetes | -0.0247 | 120451362 | 0.0022 | 0.0002 | 0.0073 | 0.9782 |
| rs117818446 | ALM | Type 2 diabetes | 0.0423 | 67223589 | 0.0068 | 0.0158 | 0.025 | 0.5278 |
| rs117972846 | ALM | Type 2 diabetes | 0.0335 | 26947476 | 0.0057 | 0.0172 | 0.021 | 0.4138 |
| rs11867855 | ALM | Type 2 diabetes | -0.0132 | 18262584 | 0.0022 | 0.0202 | 0.0074 | 0.006582 |
| rs1190540 | ALM | Type 2 diabetes | 0.0125 | 102897009 | 0.0021 | -0.0091 | 0.007 | 0.1946 |
| rs11959466 | ALM | Type 2 diabetes | 0.038 | 42803824 | 0.0042 | -0.02 | 0.0152 | 0.1877 |
| rs1202186 | ALM | Type 2 diabetes | -0.012 | 87213258 | 0.002 | -0.0071 | 0.0067 | 0.2893 |
| rs12051245 | ALM | Type 2 diabetes | 0.0299 | 783865 | 0.0022 | -0.0066 | 0.008 | 0.4069 |
| rs12074850 | ALM | Type 2 diabetes | 0.0393 | 51248316 | 0.0033 | -0.0663 | 0.0112 | 3.259E-09 |
| rs12099669 | ALM | Type 2 diabetes | 0.0331 | 46783653 | 0.002 | -0.0137 | 0.0068 | 0.04411 |
| rs12150907 | ALM | Type 2 diabetes | -0.0219 | 4940630 | 0.0024 | 0.0444 | 0.0082 | 5.429E-08 |
| rs12185775 | ALM | Type 2 diabetes | -0.0167 | 20293769 | 0.003 | 0.0128 | 0.0105 | 0.2215 |
| rs12188208 | ALM | Type 2 diabetes | -0.0195 | 77442791 | 0.0022 | -0.0157 | 0.0077 | 4.27E-02 |
| rs12230946 | ALM | Type 2 diabetes | 0.0271 | 53498725 | 0.0033 | 0.0004 | 0.0109 | 0.9707 |
| rs12334478 | ALM | Type 2 diabetes | -0.0161 | 141998765 | 0.0019 | 0.0044 | 0.0064 | 0.4909 |
| rs12340775 | ALM | Type 2 diabetes | -0.0287 | 13226945 | 0.0043 | -0.0205 | 0.0146 | 0.159 |
| rs12344515 | ALM | Type 2 diabetes | -0.0163 | 113801231 | 0.0022 | -0.0021 | 0.0075 | 0.7806 |
| rs12347137 | ALM | Type 2 diabetes | -0.046 | 119122721 | 0.0024 | 0.0205 | 0.0079 | 0.009039 |
| rs12351226 | ALM | Type 2 diabetes | 0.0218 | 98405230 | 0.0025 | -0.0146 | 0.0088 | 0.09691 |
| rs12461874 | ALM | Type 2 diabetes | -0.0181 | 17180358 | 0.0021 | 0.0069 | 0.0072 | 0.3395 |
| rs12483401 | ALM | Type 2 diabetes | -0.0387 | 35443829 | 0.0067 | 0.0575 | 0.0228 | 0.01176 |
| rs12512942 | ALM | Type 2 diabetes | -0.0162 | 177766307 | 0.002 | -0.003 | 0.0066 | 0.6493 |
| rs12517711 | ALM | Type 2 diabetes | -0.0147 | 60754661 | 0.0019 | 0.0034 | 0.0066 | 0.6062 |
| rs12519407 | ALM | Type 2 diabetes | 0.0181 | 137651012 | 0.0022 | 0.0184 | 0.0072 | 0.01087 |
| rs12533452 | ALM | Type 2 diabetes | 0.0237 | 19016871 | 0.0026 | -0.0113 | 0.0087 | 0.1935 |
| rs12536902 | ALM | Type 2 diabetes | 0.0479 | 33213009 | 0.0081 | -0.0008 | 0.0294 | 0.9783 |
| rs12541381 | ALM | Type 2 diabetes | -0.0319 | 135649848 | 0.0022 | 0.0183 | 0.0074 | 0.01383 |
| rs12563442 | ALM | Type 2 diabetes | 0.0122 | 19786695 | 0.0021 | 0.0113 | 0.0072 | 0.1178 |
| rs1260326 | ALM | Type 2 diabetes | 0.0323 | 27730940 | 0.0019 | 0.0644 | 0.0066 | 1.621E-22 |
| rs12616192 | ALM | Type 2 diabetes | -0.0261 | 121568931 | 0.0038 | -0.0049 | 0.0126 | 6.97E-01 |
| rs12655296 | ALM | Type 2 diabetes | -0.011 | 15890643 | 0.002 | -0.0047 | 0.0066 | 0.4761 |
| rs12672217 | ALM | Type 2 diabetes | 0.0139 | 156310948 | 0.002 | -0.0042 | 0.0066 | 0.5243 |
| rs12700901 | ALM | Type 2 diabetes | -0.0184 | 28783171 | 0.0019 | -0.0187 | 0.0069 | 0.006808 |
| rs12702693 | ALM | Type 2 diabetes | 0.0173 | 8101039 | 0.0019 | -0.0088 | 0.0064 | 0.1683 |
| rs12713004 | ALM | Type 2 diabetes | 0.0367 | 23896049 | 0.0021 | -0.0026 | 0.0072 | 0.7189 |
| rs12724708 | ALM | Type 2 diabetes | 0.0243 | 219620569 | 0.002 | 0.0305 | 0.0067 | 0.000005324 |
| rs12773500 | ALM | Type 2 diabetes | 0.0171 | 81232632 | 0.0028 | 0.0206 | 0.009 | 0.02215 |
| rs12831751 | ALM | Type 2 diabetes | 0.0172 | 29520017 | 0.0021 | 0.0014 | 0.007 | 0.8418 |
| rs12882130 | ALM | Type 2 diabetes | -0.0202 | 103878774 | 0.002 | 0.0183 | 0.0066 | 0.005532 |
| rs12907139 | ALM | Type 2 diabetes | -0.0149 | 73521566 | 0.0019 | 0.0063 | 0.0064 | 0.3239 |
| rs1290786 | ALM | Type 2 diabetes | -0.0143 | 169097381 | 0.0019 | 0.0058 | 0.0062 | 0.3478 |
| rs12909863 | ALM | Type 2 diabetes | 0.0189 | 75825822 | 0.0022 | -0.0447 | 0.0074 | 1.821E-09 |
| rs1291114 | ALM | Type 2 diabetes | 0.0173 | 35500850 | 0.0031 | 0.002 | 0.0103 | 0.8455 |
| rs12943867 | ALM | Type 2 diabetes | 0.0184 | 79409710 | 0.002 | -0.0053 | 0.0074 | 0.4759 |
| rs12962050 | ALM | Type 2 diabetes | 0.0153 | 35179808 | 0.002 | -0.0186 | 0.0067 | 0.005508 |
| rs12997625 | ALM | Type 2 diabetes | -0.017 | 202970250 | 0.0019 | 0.0219 | 0.0064 | 0.000606 |
| rs13103161 | ALM | Type 2 diabetes | -0.0284 | 106216459 | 0.0019 | 0.0021 | 0.0066 | 0.7502 |
| rs13109280 | ALM | Type 2 diabetes | 0.0131 | 54380513 | 0.002 | 0.0001 | 0.0068 | 0.9883 |
| rs13123591 | ALM | Type 2 diabetes | 0.0185 | 120105990 | 0.002 | -0.0011 | 0.0068 | 0.8716 |
| rs13127468 | ALM | Type 2 diabetes | -0.0123 | 8599658 | 0.0019 | 0.0062 | 0.0067 | 3.55E-01 |
| rs1319012 | ALM | Type 2 diabetes | -0.052 | 41852616 | 0.0037 | 0.0459 | 0.0121 | 0.0001573 |
| rs13209685 | ALM | Type 2 diabetes | 0.0277 | 7779729 | 0.0026 | 0.006 | 0.0086 | 0.4846 |
| rs1324538 | ALM | Type 2 diabetes | 0.0237 | 45080144 | 0.0019 | 0.009 | 0.0066 | 0.1724 |
| rs1325596 | ALM | Type 2 diabetes | 0.0287 | 176794066 | 0.0019 | -0.0094 | 0.0065 | 0.1476 |
| rs1330826 | ALM | Type 2 diabetes | 0.0162 | 85129970 | 0.0023 | -0.0008 | 0.0077 | 9.18E-01 |
| rs13316 | ALM | Type 2 diabetes | 0.0115 | 93407301 | 0.0019 | -0.0099 | 0.0066 | 0.1334 |
| rs13391980 | ALM | Type 2 diabetes | -0.0225 | 165504841 | 0.0029 | -0.0848 | 0.0097 | 3.072E-18 |
| rs1340022 | ALM | Type 2 diabetes | 0.0118 | 131334465 | 0.0019 | 0.0055 | 0.0064 | 0.3892 |
| rs1341215 | ALM | Type 2 diabetes | 0.0229 | 111662350 | 0.0027 | -0.0116 | 0.0092 | 0.208 |
| rs13430869 | ALM | Type 2 diabetes | 0.0272 | 218146818 | 0.0021 | 0.0083 | 0.0072 | 0.2506 |
| rs139921635 | ALM | Type 2 diabetes | 0.0385 | 73181637 | 0.0062 | -0.0086 | 0.0223 | 0.6998 |
| rs140440099 | ALM | Type 2 diabetes | 0.0613 | 50632595 | 0.0063 | 0.0176 | 0.0235 | 0.453 |
| rs1405227 | ALM | Type 2 diabetes | 0.0129 | 98873390 | 0.002 | -0.0087 | 0.0069 | 0.208 |
| rs143076454 | ALM | Type 2 diabetes | -0.0499 | 921179 | 0.007 | -0.0116 | 0.0273 | 0.6712 |
| rs143384 | ALM | Type 2 diabetes | 0.0725 | 34025756 | 0.0019 | 0.01 | 0.0065 | 0.1234 |
| rs144109601 | ALM | Type 2 diabetes | -0.0278 | 50455500 | 0.0048 | -0.0195 | 0.0159 | 0.2205 |
| rs1443536 | ALM | Type 2 diabetes | 0.0218 | 82174165 | 0.0021 | 0.0114 | 0.0068 | 0.09392 |
| rs1444628 | ALM | Type 2 diabetes | 0.024 | 20563643 | 0.002 | -0.0011 | 0.0069 | 0.8735 |
| rs144627572 | ALM | Type 2 diabetes | 0.0439 | 20583907 | 0.0053 | -0.0229 | 0.0185 | 0.2166 |
| rs147110934 | ALM | Type 2 diabetes | -0.0722 | 55993436 | 0.0062 | 0.044 | 0.0231 | 0.05723 |
| rs147233090 | ALM | Type 2 diabetes | -0.0446 | 44028047 | 0.0061 | -0.0474 | 0.0207 | 0.02223 |
| rs1472852 | ALM | Type 2 diabetes | -0.0638 | 17910236 | 0.0026 | 0.0213 | 0.009 | 0.01801 |
| rs1478575 | ALM | Type 2 diabetes | 0.0312 | 218278555 | 0.002 | 0.0029 | 0.0068 | 0.67 |
| rs14976 | ALM | Type 2 diabetes | 0.0144 | 85818886 | 0.002 | -0.0205 | 0.0074 | 0.005822 |
| rs1514134 | ALM | Type 2 diabetes | -0.0114 | 56116513 | 0.0019 | -0.0019 | 0.0065 | 0.7698 |
| rs1556659 | ALM | Type 2 diabetes | 0.0163 | 130834698 | 0.002 | -0.0024 | 0.0062 | 0.6976 |
| rs1584011 | ALM | Type 2 diabetes | 0.0159 | 27080527 | 0.002 | 0.0076 | 0.0066 | 0.2493 |
| rs16989695 | ALM | Type 2 diabetes | -0.0139 | 4505445 | 0.0019 | -0.0124 | 0.0066 | 0.06013 |
| rs17197114 | ALM | Type 2 diabetes | 0.0177 | 21894526 | 0.0025 | 0.0144 | 0.0086 | 0.0935 |
| rs17205463 | ALM | Type 2 diabetes | -0.0263 | 62381413 | 0.0019 | -0.0258 | 0.0065 | 0.00007056 |
| rs17246129 | ALM | Type 2 diabetes | 0.0254 | 227259964 | 0.002 | 0.0091 | 0.007 | 0.1946 |
| rs17278379 | ALM | Type 2 diabetes | 0.0226 | 172381284 | 0.0029 | 0.0174 | 0.0098 | 0.07707 |
| rs1730028 | ALM | Type 2 diabetes | 0.0131 | 157900789 | 0.0019 | -0.0213 | 0.0065 | 0.001034 |
| rs173135 | ALM | Type 2 diabetes | -0.0341 | 68172326 | 0.003 | -0.0329 | 0.0102 | 0.001198 |
| rs17400325 | ALM | Type 2 diabetes | 0.0345 | 178565913 | 0.0047 | 0.0104 | 0.0173 | 0.5472 |
| rs17478946 | ALM | Type 2 diabetes | -0.0192 | 24093062 | 0.0021 | -0.0086 | 0.0071 | 0.2271 |
| rs17681189 | ALM | Type 2 diabetes | -0.0131 | 65976175 | 0.0019 | -0.0115 | 0.0065 | 7.65E-02 |
| rs17718736 | ALM | Type 2 diabetes | 0.0115 | 71555205 | 0.002 | -0.0167 | 0.0068 | 0.01413 |
| rs177591 | ALM | Type 2 diabetes | -0.0191 | 28556199 | 0.0027 | 0.0118 | 0.0091 | 0.1952 |
| rs17773965 | ALM | Type 2 diabetes | -0.0163 | 217631338 | 0.0027 | 0.0045 | 0.009 | 0.6172 |
| rs17818592 | ALM | Type 2 diabetes | -0.0129 | 86088594 | 0.0019 | 0.0057 | 0.0064 | 0.3721 |
| rs1786263 | ALM | Type 2 diabetes | -0.019 | 13116432 | 0.0019 | 0.0066 | 0.0065 | 0.3093 |
| rs1797070 | ALM | Type 2 diabetes | 0.0219 | 218630201 | 0.0021 | 0.0129 | 0.0072 | 0.07416 |
| rs1823217 | ALM | Type 2 diabetes | -0.0181 | 134380959 | 0.002 | -0.0045 | 0.0067 | 0.5019 |
| rs1880318 | ALM | Type 2 diabetes | 0.0147 | 46028167 | 0.0024 | -0.0158 | 0.0081 | 0.05002 |
| rs1899040 | ALM | Type 2 diabetes | 0.0152 | 223901896 | 0.0023 | -0.0073 | 0.008 | 0.3589 |
| rs1933081 | ALM | Type 2 diabetes | 0.0267 | 151651505 | 0.0034 | 0.0035 | 0.0117 | 0.7653 |
| rs200439 | ALM | Type 2 diabetes | -0.0128 | 6716083 | 0.0023 | -0.0191 | 0.0077 | 0.0137 |
| rs2005172 | ALM | Type 2 diabetes | 0.048 | 61996255 | 0.002 | -0.0082 | 0.0067 | 0.2211 |
| rs2019203 | ALM | Type 2 diabetes | 0.0189 | 36908672 | 0.0019 | -0.0001 | 0.0065 | 0.9877 |
| rs2025609 | ALM | Type 2 diabetes | 0.0186 | 67422990 | 0.0026 | -0.0126 | 0.0088 | 0.152 |
| rs2025808 | ALM | Type 2 diabetes | 0.0122 | 184161757 | 0.0022 | 0.0063 | 0.0073 | 0.39 |
| rs2035901 | ALM | Type 2 diabetes | 0.024 | 145521867 | 0.0019 | -0.0158 | 0.0064 | 1.34E-02 |
| rs2070598 | ALM | Type 2 diabetes | 0.0204 | 75360906 | 0.0019 | -0.0096 | 0.0064 | 0.1328 |
| rs2071450 | ALM | Type 2 diabetes | -0.0174 | 54428532 | 0.002 | -0.0252 | 0.0066 | 0.0001333 |
| rs2089111 | ALM | Type 2 diabetes | -0.0172 | 91180019 | 0.0022 | 0.0062 | 0.0073 | 0.3976 |
| rs2101017 | ALM | Type 2 diabetes | -0.0223 | 122306857 | 0.0028 | 0.0024 | 0.0091 | 0.7922 |
| rs2105333 | ALM | Type 2 diabetes | -0.019 | 158755437 | 0.002 | -0.0072 | 0.0067 | 0.2826 |
| rs2112617 | ALM | Type 2 diabetes | -0.0167 | 46977125 | 0.0019 | 0.046 | 0.0064 | 5.92E-13 |
| rs212526 | ALM | Type 2 diabetes | 0.0214 | 21584941 | 0.0019 | -0.0017 | 0.0063 | 0.7867 |
| rs2138374 | ALM | Type 2 diabetes | -0.0149 | 190014317 | 0.002 | -0.001 | 0.0069 | 0.8849 |
| rs2140619 | ALM | Type 2 diabetes | 0.0113 | 114007270 | 0.0019 | 0.0024 | 0.0065 | 0.7116 |
| rs2142331 | ALM | Type 2 diabetes | -0.0165 | 116636719 | 0.0019 | -0.0324 | 0.0066 | 9.021E-07 |
| rs2174008 | ALM | Type 2 diabetes | -0.0192 | 38510456 | 0.0019 | -0.0081 | 0.0064 | 0.2047 |
| rs2181834 | ALM | Type 2 diabetes | 0.0254 | 102661251 | 0.0019 | -0.0014 | 0.0065 | 0.8292 |
| rs2188805 | ALM | Type 2 diabetes | 0.0114 | 93078400 | 0.002 | -0.0045 | 0.0067 | 0.5019 |
| rs2194411 | ALM | Type 2 diabetes | 0.0443 | 185548663 | 0.0029 | -0.0746 | 0.0097 | 1.841E-14 |
| rs2209098 | ALM | Type 2 diabetes | 0.024 | 172167226 | 0.002 | -0.0044 | 0.0068 | 5.18E-01 |
| rs2212926 | ALM | Type 2 diabetes | -0.022 | 38066883 | 0.0023 | 0.0114 | 0.0082 | 0.1627 |
| rs2229840 | ALM | Type 2 diabetes | 0.0341 | 124826462 | 0.0026 | -0.0256 | 0.0088 | 0.003606 |
| rs2230033 | ALM | Type 2 diabetes | -0.0265 | 39671476 | 0.0019 | 0.0088 | 0.0064 | 0.1683 |
| rs2236406 | ALM | Type 2 diabetes | 0.0394 | 98221861 | 0.002 | -0.026 | 0.0067 | 0.0001044 |
| rs2237485 | ALM | Type 2 diabetes | 0.0191 | 50749870 | 0.0023 | -0.0121 | 0.0075 | 0.1085 |
| rs2240735 | ALM | Type 2 diabetes | 0.0189 | 4027605 | 0.0022 | 0.0158 | 0.0075 | 0.03609 |
| rs2268718 | ALM | Type 2 diabetes | 0.0141 | 52415023 | 0.0021 | -0.0108 | 0.0071 | 0.1293 |
| rs2270894 | ALM | Type 2 diabetes | -0.0332 | 9975386 | 0.0024 | 0.0053 | 0.0082 | 0.5164 |
| rs2274351 | ALM | Type 2 diabetes | 0.017 | 104264107 | 0.0019 | 0.0093 | 0.0064 | 0.1454 |
| rs2283200 | ALM | Type 2 diabetes | -0.0281 | 2729340 | 0.0042 | 0.0263 | 0.0141 | 0.06279 |
| rs2287821 | ALM | Type 2 diabetes | -0.0153 | 33935102 | 0.0019 | -0.0302 | 0.0064 | 0.000002262 |
| rs2289629 | ALM | Type 2 diabetes | -0.0148 | 27959903 | 0.002 | 0.0243 | 0.0068 | 0.0003562 |
| rs2296316 | ALM | Type 2 diabetes | -0.0192 | 65520246 | 0.0019 | 0.0148 | 0.0064 | 0.02049 |
| rs2303423 | ALM | Type 2 diabetes | 0.0168 | 38120029 | 0.003 | -0.0245 | 0.0103 | 0.01695 |
| rs2305141 | ALM | Type 2 diabetes | 0.0183 | 233684402 | 0.0019 | -0.0211 | 0.0066 | 0.00138 |
| rs2324154 | ALM | Type 2 diabetes | 0.015 | 24027226 | 0.0019 | -0.0008 | 0.0064 | 0.9003 |
| rs234640 | ALM | Type 2 diabetes | -0.0131 | 184867830 | 0.0019 | 0.0044 | 0.0065 | 0.4979 |
| rs2347603 | ALM | Type 2 diabetes | -0.0181 | 47297426 | 0.0022 | 0.0072 | 0.0073 | 0.3259 |
| rs2347808 | ALM | Type 2 diabetes | -0.0125 | 2750856 | 0.0019 | 0.0002 | 0.0064 | 0.975 |
| rs2362487 | ALM | Type 2 diabetes | 0.0154 | 126208402 | 0.0022 | 0.0089 | 0.0076 | 0.2443 |
| rs2390669 | ALM | Type 2 diabetes | 0.0174 | 169091942 | 0.0028 | -0.0011 | 0.0098 | 0.911 |
| rs244711 | ALM | Type 2 diabetes | 0.0279 | 176509193 | 0.0022 | -0.01 | 0.0077 | 0.1968 |
| rs2454390 | ALM | Type 2 diabetes | -0.0176 | 103255613 | 0.0026 | -0.0074 | 0.009 | 0.4112 |
| rs246177 | ALM | Type 2 diabetes | 0.0214 | 14380768 | 0.002 | 0.0013 | 0.0067 | 0.8462 |
| rs249677 | ALM | Type 2 diabetes | -0.0109 | 141539339 | 0.002 | 0.0117 | 0.0067 | 0.08081 |
| rs2521349 | ALM | Type 2 diabetes | 0.0155 | 67503501 | 0.0019 | 0.0021 | 0.0066 | 0.7502 |
| rs2529090 | ALM | Type 2 diabetes | 0.0136 | 24662280 | 0.0025 | -0.005 | 0.0087 | 0.5651 |
| rs2545339 | ALM | Type 2 diabetes | 0.0115 | 149911219 | 0.002 | 0.0037 | 0.0067 | 0.5808 |
| rs2569888 | ALM | Type 2 diabetes | 0.0133 | 1625803 | 0.0022 | -0.0028 | 0.0075 | 0.7103 |
| rs2578565 | ALM | Type 2 diabetes | -0.0141 | 5460569 | 0.002 | 0.0109 | 0.0067 | 0.1038 |
| rs2592208 | ALM | Type 2 diabetes | -0.0124 | 67408873 | 0.0019 | -0.0021 | 0.0064 | 0.7423 |
| rs2607234 | ALM | Type 2 diabetes | -0.0302 | 35563834 | 0.0043 | -0.0051 | 0.0143 | 0.7222 |
| rs261223 | ALM | Type 2 diabetes | 0.0175 | 95901046 | 0.0019 | 0.0104 | 0.0072 | 0.15 |
| rs2648725 | ALM | Type 2 diabetes | 0.0165 | 93015079 | 0.0023 | -0.0129 | 0.008 | 0.105 |
| rs2663126 | ALM | Type 2 diabetes | -0.0139 | 99563857 | 0.0021 | 0.0089 | 0.007 | 2.05E-01 |
| rs2676298 | ALM | Type 2 diabetes | -0.0269 | 62726707 | 0.0027 | 0.0132 | 0.0089 | 0.138 |
| rs2717008 | ALM | Type 2 diabetes | -0.0127 | 58149158 | 0.0019 | 0.0044 | 0.0066 | 0.5047 |
| rs2748501 | ALM | Type 2 diabetes | -0.0195 | 146312258 | 0.0019 | 0.0135 | 0.0064 | 0.03454 |
| rs2754255 | ALM | Type 2 diabetes | -0.0153 | 88393572 | 0.0023 | 0.001 | 0.0076 | 0.8959 |
| rs2763263 | ALM | Type 2 diabetes | -0.017 | 168814392 | 0.0022 | 0.0069 | 0.0073 | 0.3465 |
| rs2764264 | ALM | Type 2 diabetes | 0.0203 | 108934461 | 0.0021 | 0.0053 | 0.0069 | 0.4431 |
| rs2788213 | ALM | Type 2 diabetes | 0.0123 | 703249 | 0.0021 | -0.0066 | 0.007 | 0.3468 |
| rs2789365 | ALM | Type 2 diabetes | -0.0145 | 235515534 | 0.0019 | -0.0161 | 0.0064 | 0.01171 |
| rs2807339 | ALM | Type 2 diabetes | 0.0162 | 22578063 | 0.0022 | -0.0165 | 0.0074 | 0.02645 |
| rs2812208 | ALM | Type 2 diabetes | 0.1156 | 50707087 | 0.0066 | -0.0244 | 0.0218 | 0.2625 |
| rs28379706 | ALM | Type 2 diabetes | 0.0114 | 50728062 | 0.002 | -0.0265 | 0.0067 | 0.00007664 |
| rs28485212 | ALM | Type 2 diabetes | -0.0188 | 63550026 | 0.0027 | 0.0101 | 0.0092 | 0.273 |
| rs28529426 | ALM | Type 2 diabetes | -0.0168 | 4678264 | 0.0026 | 0.0282 | 0.0088 | 0.001344 |
| rs28592876 | ALM | Type 2 diabetes | 0.03 | 123866429 | 0.0023 | -0.043 | 0.0079 | 4.354E-08 |
| rs28678024 | ALM | Type 2 diabetes | -0.0119 | 25937161 | 0.0021 | -0.0344 | 0.007 | 9.403E-07 |
| rs2871865 | ALM | Type 2 diabetes | -0.0493 | 99194896 | 0.003 | 0.0255 | 0.0104 | 0.01389 |
| rs2871960 | ALM | Type 2 diabetes | 0.0469 | 141121814 | 0.0019 | -0.03 | 0.0067 | 0.000007571 |
| rs28736838 | ALM | Type 2 diabetes | -0.0117 | 120148713 | 0.002 | 0.0014 | 0.007 | 0.8418 |
| rs2885697 | ALM | Type 2 diabetes | -0.0323 | 41544279 | 0.002 | 0.0061 | 0.0067 | 0.3627 |
| rs291979 | ALM | Type 2 diabetes | 0.0242 | 121129797 | 0.0023 | 0.0121 | 0.0076 | 0.1134 |
| rs2923411 | ALM | Type 2 diabetes | 0.0127 | 42455206 | 0.0019 | -0.0096 | 0.0066 | 0.1456 |
| rs2925155 | ALM | Type 2 diabetes | -0.015 | 75886297 | 0.0022 | -0.0053 | 0.0073 | 0.4696 |
| rs293517 | ALM | Type 2 diabetes | -0.013 | 83662455 | 0.0021 | 0.0049 | 0.0071 | 0.4913 |
| rs2971857 | ALM | Type 2 diabetes | -0.0119 | 234369487 | 0.0019 | -0.0155 | 0.0065 | 0.01695 |
| rs2978362 | ALM | Type 2 diabetes | 0.0106 | 32959397 | 0.0019 | 0.007 | 0.0064 | 0.2731 |
| rs301807 | ALM | Type 2 diabetes | -0.0144 | 8484823 | 0.0019 | -0.0024 | 0.0065 | 0.7116 |
| rs3103223 | ALM | Type 2 diabetes | 0.0126 | 42402721 | 0.0022 | 0 | 0.0073 | 1 |
| rs310796 | ALM | Type 2 diabetes | 0.0142 | 77453226 | 0.002 | -0.0155 | 0.0067 | 0.02072 |
| rs3116194 | ALM | Type 2 diabetes | -0.0295 | 233061266 | 0.0032 | 0.0025 | 0.0111 | 0.8218 |
| rs3116602 | ALM | Type 2 diabetes | -0.0612 | 51111355 | 0.0023 | 0.0154 | 0.0077 | 0.04685 |
| rs31196 | ALM | Type 2 diabetes | -0.0107 | 158300798 | 0.0019 | -0.0151 | 0.0064 | 0.01807 |
| rs3184504 | ALM | Type 2 diabetes | 0.0183 | 111884608 | 0.0019 | -0.0193 | 0.0063 | 0.002125 |
| rs3205136 | ALM | Type 2 diabetes | -0.0184 | 136126631 | 0.0033 | 0.0208 | 0.0104 | 0.04479 |
| rs331917 | ALM | Type 2 diabetes | -0.0127 | 98158524 | 0.0019 | -0.0156 | 0.0065 | 0.01626 |
| rs336630 | ALM | Type 2 diabetes | -0.0106 | 18607538 | 0.0019 | -0.0096 | 0.0066 | 0.1456 |
| rs33973388 | ALM | Type 2 diabetes | 0.0249 | 46611842 | 0.0019 | 0.009 | 0.0065 | 0.1656 |
| rs34287 | ALM | Type 2 diabetes | 0.0187 | 67585143 | 0.002 | 0.0037 | 0.0067 | 0.5808 |
| rs34312629 | ALM | Type 2 diabetes | -0.017 | 24079795 | 0.0021 | 0.0053 | 0.0073 | 0.4696 |
| rs34338597 | ALM | Type 2 diabetes | -0.0112 | 106301580 | 0.0019 | -0.0179 | 0.0066 | 0.006654 |
| rs34345560 | ALM | Type 2 diabetes | 0.0219 | 69081998 | 0.0024 | 0.0184 | 0.0082 | 0.02426 |
| rs34390533 | ALM | Type 2 diabetes | -0.0257 | 184030838 | 0.0022 | -0.0025 | 0.0074 | 0.7366 |
| rs34517439 | ALM | Type 2 diabetes | 0.0421 | 78450517 | 0.0029 | -0.0146 | 0.0102 | 1.51E-01 |
| rs34522021 | ALM | Type 2 diabetes | 0.0126 | 23350420 | 0.0019 | 0.021 | 0.0064 | 0.001009 |
| rs34776209 | ALM | Type 2 diabetes | -0.0317 | 23513093 | 0.0022 | 0.0218 | 0.0075 | 0.00383 |
| rs34879158 | ALM | Type 2 diabetes | -0.0363 | 32300634 | 0.0022 | 0.0104 | 0.0074 | 0.1618 |
| rs350832 | ALM | Type 2 diabetes | -0.0165 | 4069426 | 0.0023 | 0.0134 | 0.0082 | 0.1008 |
| rs35268848 | ALM | Type 2 diabetes | 0.0737 | 67927240 | 0.0101 | 0.0381 | 0.0351 | 0.2774 |
| rs35732917 | ALM | Type 2 diabetes | 0.0204 | 73013269 | 0.0021 | 0.0106 | 0.0071 | 0.1365 |
| rs35756741 | ALM | Type 2 diabetes | -0.0378 | 12868701 | 0.0033 | 0.0151 | 0.011 | 0.1696 |
| rs35811052 | ALM | Type 2 diabetes | -0.0148 | 15128416 | 0.0022 | 0.0012 | 0.0073 | 0.8699 |
| rs35816944 | ALM | Type 2 diabetes | -0.1088 | 1828030 | 0.0117 | 0.0026 | 0.0443 | 0.9532 |
| rs35963161 | ALM | Type 2 diabetes | -0.0157 | 49210635 | 0.0019 | 0.005 | 0.0066 | 0.4484 |
| rs36000545 | ALM | Type 2 diabetes | -0.022 | 79093822 | 0.002 | -0.0151 | 0.007 | 0.03136 |
| rs36012032 | ALM | Type 2 diabetes | 0.0298 | 52814709 | 0.0033 | -0.0094 | 0.0112 | 0.4014 |
| rs36048468 | ALM | Type 2 diabetes | 0.0254 | 122879901 | 0.0023 | -0.002 | 0.0079 | 0.799 |
| rs36226649 | ALM | Type 2 diabetes | 0.0485 | 24835500 | 0.0038 | -0.0159 | 0.0128 | 0.2132 |
| rs3764002 | ALM | Type 2 diabetes | 0.028 | 108618630 | 0.0021 | -0.0511 | 0.0073 | 3.122E-12 |
| rs3768495 | ALM | Type 2 diabetes | -0.0178 | 109935325 | 0.0021 | 0.0001 | 0.007 | 0.9886 |
| rs3769598 | ALM | Type 2 diabetes | 0.0171 | 32679732 | 0.0027 | -0.0192 | 0.0092 | 3.72E-02 |
| rs377599 | ALM | Type 2 diabetes | 0.0217 | 2164699 | 0.0019 | -0.0185 | 0.0069 | 0.007425 |
| rs3778858 | ALM | Type 2 diabetes | 0.0108 | 129963356 | 0.002 | 0.0136 | 0.0066 | 3.92E-02 |
| rs3782232 | ALM | Type 2 diabetes | -0.0339 | 57116249 | 0.0037 | 0.0281 | 0.0127 | 0.02655 |
| rs3782811 | ALM | Type 2 diabetes | -0.0165 | 3339927 | 0.0022 | -0.0005 | 0.0074 | 0.9464 |
| rs3792819 | ALM | Type 2 diabetes | 0.021 | 172576296 | 0.0034 | -0.0098 | 0.0115 | 0.3948 |
| rs3818416 | ALM | Type 2 diabetes | 0.0279 | 78474468 | 0.0022 | 0.0005 | 0.0076 | 0.9478 |
| rs3822742 | ALM | Type 2 diabetes | 0.0162 | 139059017 | 0.002 | 0.003 | 0.0067 | 0.6544 |
| rs3828729 | ALM | Type 2 diabetes | -0.016 | 155554707 | 0.002 | 0.0001 | 0.0068 | 0.9883 |
| rs3901421 | ALM | Type 2 diabetes | 0.0215 | 96204538 | 0.0019 | 0.0025 | 0.0064 | 0.6955 |
| rs395980 | ALM | Type 2 diabetes | -0.0184 | 177430072 | 0.0021 | 0.0089 | 0.0071 | 0.2113 |
| rs40270 | ALM | Type 2 diabetes | 0.0151 | 55804552 | 0.0022 | 0.0683 | 0.0075 | 1.304E-19 |
| rs4073154 | ALM | Type 2 diabetes | 0.0274 | 129035485 | 0.0023 | -0.0244 | 0.0079 | 0.001889 |
| rs4076108 | ALM | Type 2 diabetes | 0.0174 | 13736088 | 0.0022 | -0.0127 | 0.0074 | 0.08756 |
| rs4077103 | ALM | Type 2 diabetes | -0.0143 | 49557732 | 0.0026 | 0.0031 | 0.0086 | 0.718 |
| rs4121583 | ALM | Type 2 diabetes | 0.0118 | 125075 | 0.002 | -0.0051 | 0.0069 | 4.61E-01 |
| rs41271299 | ALM | Type 2 diabetes | 0.0616 | 19839415 | 0.0043 | -0.0108 | 0.0156 | 0.4888 |
| rs41311445 | ALM | Type 2 diabetes | -0.0328 | 42070374 | 0.0032 | 0.0166 | 0.0107 | 0.1201 |
| rs42039 | ALM | Type 2 diabetes | 0.0481 | 92244422 | 0.0022 | -0.0251 | 0.0074 | 7.34E-04 |
| rs4244809 | ALM | Type 2 diabetes | -0.0262 | 2164333 | 0.0023 | -0.0217 | 0.0082 | 0.007882 |
| rs4252548 | ALM | Type 2 diabetes | -0.0753 | 55879672 | 0.0065 | -0.0186 | 0.0219 | 0.3953 |
| rs4274112 | ALM | Type 2 diabetes | -0.0217 | 26746199 | 0.002 | 0.0264 | 0.0066 | 0.00006274 |
| rs4282339 | ALM | Type 2 diabetes | -0.0311 | 168256240 | 0.0023 | 0.0029 | 0.008 | 0.7155 |
| rs4287835 | ALM | Type 2 diabetes | 0.0147 | 31457337 | 0.0019 | 0.0021 | 0.0064 | 0.7423 |
| rs4360494 | ALM | Type 2 diabetes | -0.0198 | 38455891 | 0.0019 | -0.0062 | 0.0064 | 0.3317 |
| rs4383083 | ALM | Type 2 diabetes | 0.0111 | 63080442 | 0.002 | 0.0004 | 0.0068 | 0.9531 |
| rs4504126 | ALM | Type 2 diabetes | 0.046 | 33600582 | 0.0058 | 0.028 | 0.0198 | 0.1571 |
| rs45474992 | ALM | Type 2 diabetes | -0.0617 | 47724564 | 0.0051 | -0.0234 | 0.0182 | 0.199 |
| rs45528934 | ALM | Type 2 diabetes | 0.0262 | 23793305 | 0.0026 | -0.0172 | 0.009 | 0.05611 |
| rs4602848 | ALM | Type 2 diabetes | 0.016 | 92186933 | 0.002 | -0.0038 | 0.0068 | 0.5766 |
| rs4622329 | ALM | Type 2 diabetes | 0.0149 | 102321935 | 0.002 | 0.0066 | 0.0067 | 0.3247 |
| rs4640244 | ALM | Type 2 diabetes | -0.02 | 21284223 | 0.0019 | 0.0228 | 0.0067 | 0.0006677 |
| rs4644481 | ALM | Type 2 diabetes | -0.0112 | 155130900 | 0.0019 | 0.012 | 0.0065 | 0.06452 |
| rs4655345 | ALM | Type 2 diabetes | -0.0246 | 214608704 | 0.0019 | 0.0045 | 0.0066 | 0.4951 |
| rs4682483 | ALM | Type 2 diabetes | -0.0165 | 112993982 | 0.0026 | 0.0238 | 0.0086 | 0.00557 |
| rs4683435 | ALM | Type 2 diabetes | 0.0144 | 142624732 | 0.0022 | -0.0043 | 0.0076 | 0.5737 |
| rs4735761 | ALM | Type 2 diabetes | 0.0331 | 78097161 | 0.0021 | -0.0101 | 0.0071 | 0.156 |
| rs4748008 | ALM | Type 2 diabetes | -0.0125 | 12935125 | 0.0019 | 0 | 0.0064 | 1 |
| rs4752689 | ALM | Type 2 diabetes | 0.0205 | 124131176 | 0.0019 | -0.0326 | 0.0065 | 5.12E-07 |
| rs4752829 | ALM | Type 2 diabetes | 0.0262 | 47396654 | 0.0021 | -0.0147 | 0.0071 | 0.03895 |
| rs4788218 | ALM | Type 2 diabetes | 0.0275 | 30055750 | 0.0019 | 0.0401 | 0.0065 | 6.522E-10 |
| rs4807472 | ALM | Type 2 diabetes | -0.0158 | 3448842 | 0.002 | -0.0045 | 0.0071 | 0.5274 |
| rs4815952 | ALM | Type 2 diabetes | -0.0161 | 6934897 | 0.0019 | -0.0005 | 0.0065 | 0.9386 |
| rs4818280 | ALM | Type 2 diabetes | -0.0124 | 18114472 | 0.002 | 0.0037 | 0.0066 | 0.5748 |
| rs4847378 | ALM | Type 2 diabetes | 0.0136 | 93324634 | 0.0019 | -0.0213 | 0.0065 | 0.001034 |
| rs4852257 | ALM | Type 2 diabetes | -0.0231 | 71678520 | 0.0019 | -0.0069 | 0.0066 | 0.2955 |
| rs4865956 | ALM | Type 2 diabetes | -0.0258 | 54882505 | 0.0021 | -0.0138 | 0.0069 | 0.04582 |
| rs4870941 | ALM | Type 2 diabetes | -0.0297 | 126498828 | 0.0023 | -0.0193 | 0.0076 | 0.01157 |
| rs488621 | ALM | Type 2 diabetes | 0.0191 | 169707552 | 0.0019 | -0.0032 | 0.0064 | 0.6164 |
| rs4932439 | ALM | Type 2 diabetes | -0.0151 | 89401109 | 0.0025 | 0.0087 | 0.0084 | 0.299 |
| rs4938359 | ALM | Type 2 diabetes | -0.0156 | 117093560 | 0.0024 | -0.0007 | 0.0079 | 0.929 |
| rs4940874 | ALM | Type 2 diabetes | 0.0148 | 57105638 | 0.0024 | 0.0057 | 0.0082 | 0.4852 |
| rs4965298 | ALM | Type 2 diabetes | -0.0119 | 100802766 | 0.0021 | -0.0021 | 0.0071 | 0.768 |
| rs496783 | ALM | Type 2 diabetes | -0.0124 | 116137961 | 0.0019 | -0.0005 | 0.0065 | 0.9386 |
| rs4976262 | ALM | Type 2 diabetes | -0.0245 | 134379531 | 0.002 | 0.0017 | 0.0068 | 0.8027 |
| rs4985445 | ALM | Type 2 diabetes | -0.0175 | 69867835 | 0.0019 | 0.034 | 0.0065 | 1.627E-07 |
| rs532499 | ALM | Type 2 diabetes | -0.0127 | 30165465 | 0.0022 | 0.0131 | 0.0073 | 0.07388 |
| rs543650 | ALM | Type 2 diabetes | 0.025 | 152110943 | 0.002 | -0.0005 | 0.0066 | 0.9396 |
| rs544136 | ALM | Type 2 diabetes | 0.0121 | 101041229 | 0.0022 | -0.0077 | 0.0073 | 0.2934 |
| rs55717234 | ALM | Type 2 diabetes | 0.0122 | 150999863 | 0.0019 | 0.0019 | 0.007 | 0.7865 |
| rs55758152 | ALM | Type 2 diabetes | 0.0145 | 171317318 | 0.002 | -0.0085 | 0.007 | 0.2256 |
| rs55852614 | ALM | Type 2 diabetes | -0.0393 | 172416869 | 0.0022 | 0.0104 | 0.0074 | 0.1618 |
| rs55872725 | ALM | Type 2 diabetes | 0.0222 | 53809123 | 0.0019 | 0.122 | 0.0065 | 8.509E-79 |
| rs56112295 | ALM | Type 2 diabetes | 0.0154 | 105877057 | 0.0024 | 0.0008 | 0.0089 | 0.9284 |
| rs56207600 | ALM | Type 2 diabetes | 0.0192 | 126196537 | 0.003 | -0.021 | 0.0104 | 0.04277 |
| rs56239180 | ALM | Type 2 diabetes | -0.0459 | 32937951 | 0.0062 | 0.0092 | 0.0228 | 0.6869 |
| rs56363908 | ALM | Type 2 diabetes | -0.0382 | 96611052 | 0.0047 | -0.01 | 0.0158 | 0.5271 |
| rs568267 | ALM | Type 2 diabetes | 0.0122 | 8799828 | 0.0022 | -0.0035 | 0.0074 | 0.6378 |
| rs57059662 | ALM | Type 2 diabetes | 0.0118 | 33217275 | 0.002 | 0.0074 | 0.0068 | 0.2769 |
| rs5742915 | ALM | Type 2 diabetes | 0.0248 | 74336633 | 0.0019 | -0.0198 | 0.0065 | 0.002288 |
| rs57513571 | ALM | Type 2 diabetes | -0.0191 | 2309130 | 0.0024 | -0.0167 | 0.0081 | 0.03832 |
| rs5753518 | ALM | Type 2 diabetes | 0.0242 | 31631314 | 0.0033 | 0.0031 | 0.0109 | 0.7759 |
| rs577289 | ALM | Type 2 diabetes | -0.0125 | 40208911 | 0.0021 | 0.0109 | 0.007 | 0.1202 |
| rs57791149 | ALM | Type 2 diabetes | -0.0173 | 54222307 | 0.0019 | -0.0047 | 0.0068 | 0.4898 |
| rs591668 | ALM | Type 2 diabetes | -0.0174 | 27535931 | 0.0019 | -0.0003 | 0.0066 | 0.9637 |
| rs599004 | ALM | Type 2 diabetes | -0.0157 | 140439740 | 0.0021 | 0.0112 | 0.0071 | 0.1157 |
| rs59950280 | ALM | Type 2 diabetes | -0.0254 | 3452345 | 0.002 | 0.0161 | 0.007 | 0.02173 |
| rs59985551 | ALM | Type 2 diabetes | -0.0313 | 56106928 | 0.0022 | 0.0066 | 0.0075 | 0.3813 |
| rs6028716 | ALM | Type 2 diabetes | -0.021 | 38547459 | 0.0022 | 0.0137 | 0.0073 | 0.06159 |
| rs60389750 | ALM | Type 2 diabetes | -0.0175 | 77182836 | 0.0021 | 0.0154 | 0.0074 | 0.0383 |
| rs60408354 | ALM | Type 2 diabetes | 0.0259 | 70158495 | 0.0036 | 0.0124 | 0.0128 | 0.3317 |
| rs604723 | ALM | Type 2 diabetes | -0.0166 | 100610546 | 0.0021 | 0.0117 | 0.0071 | 0.1003 |
| rs6054390 | ALM | Type 2 diabetes | -0.0188 | 6592094 | 0.002 | 0.005 | 0.0066 | 0.4484 |
| rs6054491 | ALM | Type 2 diabetes | -0.0142 | 6709535 | 0.0022 | -0.0178 | 0.0074 | 0.01665 |
| rs6066122 | ALM | Type 2 diabetes | 0.0127 | 45558573 | 0.0023 | 0.0185 | 0.0075 | 0.01413 |
| rs60804050 | ALM | Type 2 diabetes | -0.0217 | 118870373 | 0.0021 | 0.0039 | 0.0073 | 0.5946 |
| rs610694 | ALM | Type 2 diabetes | 0.0136 | 121304826 | 0.0019 | -0.0337 | 0.0064 | 1.317E-07 |
| rs6142059 | ALM | Type 2 diabetes | 0.0116 | 32544327 | 0.0019 | 0.0245 | 0.0064 | 0.000125 |
| rs61729527 | ALM | Type 2 diabetes | -0.0346 | 77761919 | 0.0043 | -0.0068 | 0.0144 | 0.6379 |
| rs61732778 | ALM | Type 2 diabetes | 0.023 | 187443314 | 0.0037 | -0.0249 | 0.0119 | 0.03697 |
| rs61878760 | ALM | Type 2 diabetes | 0.019 | 12807189 | 0.0034 | -0.0123 | 0.0118 | 0.2985 |
| rs61919240 | ALM | Type 2 diabetes | 0.0137 | 8831954 | 0.002 | -0.024 | 0.0069 | 0.0005146 |
| rs62033029 | ALM | Type 2 diabetes | -0.0141 | 50107273 | 0.0023 | 0.0119 | 0.008 | 0.1348 |
| rs62103240 | ALM | Type 2 diabetes | 0.0212 | 77650637 | 0.0037 | -0.0108 | 0.0134 | 0.4203 |
| rs62106258 | ALM | Type 2 diabetes | -0.0504 | 417167 | 0.0044 | -0.0995 | 0.0161 | 6.792E-10 |
| rs62143873 | ALM | Type 2 diabetes | -0.0115 | 72035050 | 0.0019 | 0.0036 | 0.0065 | 0.5792 |
| rs62370472 | ALM | Type 2 diabetes | -0.0253 | 52767109 | 0.0023 | 0.0355 | 0.0077 | 0.000004609 |
| rs62466110 | ALM | Type 2 diabetes | -0.0371 | 92623541 | 0.0041 | 0.0216 | 0.0137 | 0.1153 |
| rs62501195 | ALM | Type 2 diabetes | -0.0198 | 24041988 | 0.0025 | -0.0041 | 0.0086 | 0.633 |
| rs62515437 | ALM | Type 2 diabetes | 0.0369 | 57160328 | 0.0023 | 0.004 | 0.0075 | 0.5957 |
| rs62621812 | ALM | Type 2 diabetes | 0.0743 | 127015083 | 0.0069 | 0.0275 | 0.021 | 0.1913 |
| rs6470771 | ALM | Type 2 diabetes | -0.0268 | 130743726 | 0.0025 | -0.0106 | 0.0087 | 0.2226 |
| rs6502935 | ALM | Type 2 diabetes | -0.0125 | 1650168 | 0.0022 | 0.0141 | 0.0074 | 0.05786 |
| rs650508 | ALM | Type 2 diabetes | -0.013 | 45880122 | 0.002 | 0.0077 | 0.007 | 0.2724 |
| rs6543146 | ALM | Type 2 diabetes | 0.0154 | 103096695 | 0.0019 | 0.0033 | 0.0065 | 0.6112 |
| rs655113 | ALM | Type 2 diabetes | 0.0188 | 52269151 | 0.0021 | 0.0012 | 0.0069 | 8.62E-01 |
| rs6570509 | ALM | Type 2 diabetes | -0.0244 | 142716286 | 0.0021 | -0.0113 | 0.007 | 0.1072 |
| rs6582398 | ALM | Type 2 diabetes | 0.014 | 42870444 | 0.002 | -0.0007 | 0.0066 | 0.9155 |
| rs6593210 | ALM | Type 2 diabetes | 0.0146 | 55254186 | 0.0024 | -0.0034 | 0.008 | 0.6692 |
| rs6675858 | ALM | Type 2 diabetes | -0.0137 | 224559936 | 0.0023 | 0.0082 | 0.0076 | 0.2833 |
| rs6693481 | ALM | Type 2 diabetes | -0.0143 | 203766395 | 0.002 | 0.0045 | 0.0069 | 0.5149 |
| rs670318 | ALM | Type 2 diabetes | 0.0413 | 63727542 | 0.0044 | -0.0327 | 0.0151 | 0.03009 |
| rs6721191 | ALM | Type 2 diabetes | -0.0144 | 10190115 | 0.0019 | 0.0045 | 0.0066 | 0.4951 |
| rs6738207 | ALM | Type 2 diabetes | 0.0127 | 105989716 | 0.0019 | -0.0025 | 0.0065 | 0.7002 |
| rs6739278 | ALM | Type 2 diabetes | -0.021 | 44401055 | 0.0024 | 0.0156 | 0.008 | 0.04994 |
| rs67527161 | ALM | Type 2 diabetes | -0.0182 | 63781824 | 0.0023 | 0.0143 | 0.0079 | 0.0686 |
| rs67551338 | ALM | Type 2 diabetes | 0.0576 | 3393100 | 0.004 | -0.0121 | 0.0132 | 0.359 |
| rs6762851 | ALM | Type 2 diabetes | -0.0218 | 56686329 | 0.002 | -0.0051 | 0.0068 | 0.4536 |
| rs67716382 | ALM | Type 2 diabetes | 0.0226 | 46890317 | 0.0023 | -0.0241 | 0.0077 | 0.001868 |
| rs68049170 | ALM | Type 2 diabetes | -0.0259 | 72432047 | 0.0021 | 0.0038 | 0.0071 | 0.5935 |
| rs680882 | ALM | Type 2 diabetes | 0.0133 | 18325278 | 0.0022 | -0.0202 | 0.0074 | 0.006582 |
| rs6821305 | ALM | Type 2 diabetes | 0.0204 | 122713863 | 0.0019 | 0.0037 | 0.0067 | 5.81E-01 |
| rs6849302 | ALM | Type 2 diabetes | 0.0155 | 156665074 | 0.0024 | -0.0146 | 0.008 | 0.06654 |
| rs6860245 | ALM | Type 2 diabetes | 0.0589 | 127367998 | 0.0022 | -0.0315 | 0.0076 | 0.00003768 |
| rs6874142 | ALM | Type 2 diabetes | 0.0288 | 172753555 | 0.0031 | -0.0124 | 0.0112 | 0.2684 |
| rs6902109 | ALM | Type 2 diabetes | -0.0167 | 130316559 | 0.0019 | -0.0089 | 0.0064 | 0.1635 |
| rs6931421 | ALM | Type 2 diabetes | -0.0279 | 80880138 | 0.002 | -0.0195 | 0.0068 | 0.004167 |
| rs6977416 | ALM | Type 2 diabetes | 0.0457 | 150542711 | 0.002 | 0.0379 | 0.0068 | 2.564E-08 |
| rs700677 | ALM | Type 2 diabetes | 0.0173 | 198702424 | 0.002 | 0.0144 | 0.0067 | 0.03164 |
| rs7014590 | ALM | Type 2 diabetes | -0.0228 | 89335647 | 0.0022 | -0.0115 | 0.0072 | 0.1114 |
| rs7020491 | ALM | Type 2 diabetes | -0.0178 | 128144477 | 0.0019 | -0.0198 | 0.0064 | 0.001934 |
| rs702886 | ALM | Type 2 diabetes | 0.012 | 65753310 | 0.002 | 0.0007 | 0.0066 | 0.9155 |
| rs704660 | ALM | Type 2 diabetes | 0.0153 | 30447998 | 0.0019 | 0.0052 | 0.0065 | 0.4231 |
| rs7082659 | ALM | Type 2 diabetes | 0.0156 | 12017584 | 0.0028 | -0.0051 | 0.0096 | 0.5965 |
| rs7095472 | ALM | Type 2 diabetes | 0.0267 | 70399109 | 0.0019 | -0.0217 | 0.0064 | 6.80E-04 |
| rs7107356 | ALM | Type 2 diabetes | 0.0133 | 47676170 | 0.0019 | -0.0158 | 0.0064 | 0.01337 |
| rs7129320 | ALM | Type 2 diabetes | -0.0389 | 68388220 | 0.0025 | 0.0236 | 0.0088 | 0.007289 |
| rs713467 | ALM | Type 2 diabetes | 0.0146 | 84646473 | 0.0019 | -0.0115 | 0.0064 | 0.07177 |
| rs7137546 | ALM | Type 2 diabetes | 0.0142 | 577237 | 0.0019 | -0.0033 | 0.0065 | 0.6112 |
| rs71414738 | ALM | Type 2 diabetes | 0.015 | 127876242 | 0.0025 | 0.012 | 0.0085 | 0.1571 |
| rs7144307 | ALM | Type 2 diabetes | -0.0122 | 69533837 | 0.002 | 0.0268 | 0.0066 | 0.00004846 |
| rs7185244 | ALM | Type 2 diabetes | -0.0148 | 86546887 | 0.0023 | 0.0123 | 0.0077 | 0.1124 |
| rs718603 | ALM | Type 2 diabetes | 0.0131 | 2644245 | 0.0021 | -0.0027 | 0.0071 | 0.7045 |
| rs7220127 | ALM | Type 2 diabetes | -0.0105 | 64545922 | 0.0019 | 0.0031 | 0.0065 | 0.633 |
| rs7228151 | ALM | Type 2 diabetes | -0.0185 | 57181694 | 0.0023 | -0.0023 | 0.0077 | 0.7666 |
| rs7229520 | ALM | Type 2 diabetes | -0.0224 | 46516468 | 0.002 | 0.0127 | 0.0067 | 0.05806 |
| rs723149 | ALM | Type 2 diabetes | -0.0276 | 46577056 | 0.0019 | 0.0106 | 0.0065 | 0.1025 |
| rs72656010 | ALM | Type 2 diabetes | -0.0668 | 57122215 | 0.0028 | 0.0067 | 0.0093 | 0.4721 |
| rs72657800 | ALM | Type 2 diabetes | -0.0219 | 90822051 | 0.0035 | 0.0017 | 0.0123 | 0.8896 |
| rs72695791 | ALM | Type 2 diabetes | -0.0297 | 184059452 | 0.0051 | -0.0015 | 0.0171 | 0.93 |
| rs72721979 | ALM | Type 2 diabetes | -0.0229 | 135827942 | 0.0027 | -0.0083 | 0.0093 | 0.3731 |
| rs72726050 | ALM | Type 2 diabetes | -0.0192 | 42270059 | 0.0034 | 0.0258 | 0.0114 | 0.02378 |
| rs72771070 | ALM | Type 2 diabetes | 0.015 | 19993750 | 0.0021 | 0.0028 | 0.0075 | 0.7103 |
| rs72801843 | ALM | Type 2 diabetes | 0.0313 | 53508802 | 0.0021 | -0.0225 | 0.0069 | 0.00113 |
| rs72809820 | ALM | Type 2 diabetes | -0.0111 | 97360079 | 0.002 | 0.0107 | 0.0069 | 0.1215 |
| rs72829852 | ALM | Type 2 diabetes | 0.0309 | 46633974 | 0.0039 | 0.0106 | 0.0132 | 0.4217 |
| rs72841270 | ALM | Type 2 diabetes | 0.0294 | 104642237 | 0.0028 | -0.0113 | 0.0091 | 0.2148 |
| rs72894003 | ALM | Type 2 diabetes | -0.0423 | 34775096 | 0.0038 | -0.006 | 0.0141 | 0.6712 |
| rs73006226 | ALM | Type 2 diabetes | -0.0182 | 108072728 | 0.0029 | 0.0098 | 0.0095 | 0.3037 |
| rs7301341 | ALM | Type 2 diabetes | -0.0255 | 94083105 | 0.002 | 0.0045 | 0.0068 | 0.5085 |
| rs73052033 | ALM | Type 2 diabetes | -0.0151 | 185828465 | 0.0024 | -0.027 | 0.0083 | 0.001098 |
| rs73125634 | ALM | Type 2 diabetes | -0.0195 | 20069826 | 0.0021 | -0.0374 | 0.0071 | 1.497E-07 |
| rs73197345 | ALM | Type 2 diabetes | 0.0211 | 36770120 | 0.0028 | 0.0016 | 0.0094 | 0.8652 |
| rs7321635 | ALM | Type 2 diabetes | -0.0132 | 21472055 | 0.002 | 0.0016 | 0.0066 | 0.8083 |
| rs7328187 | ALM | Type 2 diabetes | 0.0116 | 74189974 | 0.0019 | -0.009 | 0.0065 | 0.1656 |
| rs73384223 | ALM | Type 2 diabetes | -0.0205 | 3869315 | 0.0024 | 0.0257 | 0.0082 | 0.00165 |
| rs73413540 | ALM | Type 2 diabetes | -0.0124 | 3090976 | 0.0023 | -0.014 | 0.0079 | 7.46E-02 |
| rs7367519 | ALM | Type 2 diabetes | 0.0164 | 204479176 | 0.002 | -0.0196 | 0.007 | 0.005206 |
| rs73696333 | ALM | Type 2 diabetes | 0.0191 | 46669400 | 0.0024 | -0.0014 | 0.0081 | 0.8621 |
| rs73856768 | ALM | Type 2 diabetes | -0.0247 | 157788804 | 0.0035 | -0.0091 | 0.0116 | 0.4336 |
| rs7418410 | ALM | Type 2 diabetes | 0.0155 | 10236402 | 0.0019 | -0.0236 | 0.0065 | 0.0002774 |
| rs74379684 | ALM | Type 2 diabetes | -0.0272 | 94050205 | 0.0036 | 0.0108 | 0.0121 | 0.3739 |
| rs74494415 | ALM | Type 2 diabetes | -0.0417 | 74972138 | 0.0049 | -0.0013 | 0.016 | 0.9353 |
| rs7485647 | ALM | Type 2 diabetes | -0.0261 | 131631133 | 0.0026 | 0.0095 | 0.0087 | 0.2743 |
| rs75022676 | ALM | Type 2 diabetes | -0.0163 | 60293216 | 0.0023 | 0.0086 | 0.0081 | 0.2861 |
| rs7522400 | ALM | Type 2 diabetes | 0.0129 | 36613380 | 0.0022 | 0.0147 | 0.0074 | 0.04799 |
| rs75508358 | ALM | Type 2 diabetes | 0.0266 | 96926382 | 0.0045 | 0.001 | 0.0151 | 9.47E-01 |
| rs7563362 | ALM | Type 2 diabetes | 0.0352 | 620297 | 0.0027 | 0.0458 | 0.0092 | 6.668E-07 |
| rs7570235 | ALM | Type 2 diabetes | -0.0168 | 242491353 | 0.0019 | -0.0015 | 0.0067 | 0.8229 |
| rs75702986 | ALM | Type 2 diabetes | -0.0163 | 35566151 | 0.0025 | -0.0024 | 0.0085 | 7.77E-01 |
| rs757834 | ALM | Type 2 diabetes | 0.0256 | 139717200 | 0.0024 | 0.0057 | 0.0082 | 0.4852 |
| rs7598430 | ALM | Type 2 diabetes | -0.016 | 219193963 | 0.0019 | 0.0146 | 0.0065 | 0.02451 |
| rs7610055 | ALM | Type 2 diabetes | -0.0373 | 12388409 | 0.0029 | -0.0998 | 0.0097 | 1.193E-24 |
| rs7633464 | ALM | Type 2 diabetes | 0.0175 | 98715823 | 0.0019 | -0.0165 | 0.0064 | 0.009782 |
| rs76364830 | ALM | Type 2 diabetes | -0.0471 | 13372120 | 0.0039 | -0.0277 | 0.0138 | 0.04504 |
| rs76517946 | ALM | Type 2 diabetes | -0.0368 | 68354936 | 0.0035 | 0.0202 | 0.0119 | 0.09058 |
| rs7679276 | ALM | Type 2 diabetes | -0.033 | 146860186 | 0.0048 | -0.0065 | 0.0173 | 0.7067 |
| rs7689420 | ALM | Type 2 diabetes | 0.0466 | 145568352 | 0.0025 | 0.0021 | 0.0087 | 0.8091 |
| rs76895963 | ALM | Type 2 diabetes | 0.1639 | 4384844 | 0.0073 | -0.4826 | 0.0275 | 9.124E-69 |
| rs7701233 | ALM | Type 2 diabetes | -0.0179 | 171218388 | 0.0019 | -0.0093 | 0.0065 | 0.152 |
| rs772222 | ALM | Type 2 diabetes | 0.0121 | 52356892 | 0.0021 | -0.0098 | 0.0072 | 0.1749 |
| rs7731023 | ALM | Type 2 diabetes | 0.0166 | 36181627 | 0.0019 | -0.0155 | 0.0065 | 0.01695 |
| rs7735891 | ALM | Type 2 diabetes | 0.0259 | 131597005 | 0.0019 | -0.0058 | 0.0064 | 0.3638 |
| rs77364196 | ALM | Type 2 diabetes | -0.033 | 88353016 | 0.0043 | 0.0384 | 0.0153 | 0.012 |
| rs77447813 | ALM | Type 2 diabetes | 0.0224 | 50827041 | 0.0034 | 0.0057 | 0.0111 | 6.08E-01 |
| rs7768382 | ALM | Type 2 diabetes | -0.0201 | 166341870 | 0.0019 | -0.0061 | 0.0064 | 0.3395 |
| rs7768973 | ALM | Type 2 diabetes | -0.024 | 109745325 | 0.0019 | -0.0192 | 0.0065 | 0.0031 |
| rs77809369 | ALM | Type 2 diabetes | 0.0237 | 9052448 | 0.0039 | 0.006 | 0.0134 | 0.6544 |
| rs78000963 | ALM | Type 2 diabetes | 0.0169 | 30502802 | 0.0031 | -0.0129 | 0.0112 | 2.50E-01 |
| rs78051210 | ALM | Type 2 diabetes | 0.0263 | 131379491 | 0.0036 | 0.0061 | 0.012 | 0.6124 |
| rs7816345 | ALM | Type 2 diabetes | 0.0255 | 36846109 | 0.0025 | 0.0299 | 0.0085 | 0.0004226 |
| rs781669 | ALM | Type 2 diabetes | 0.0164 | 57819794 | 0.0019 | 0.0033 | 0.0063 | 0.5994 |
| rs7826059 | ALM | Type 2 diabetes | 0.0114 | 22512068 | 0.002 | -0.0265 | 0.0066 | 0.00005884 |
| rs7828086 | ALM | Type 2 diabetes | 0.0135 | 120843775 | 0.0022 | -0.0024 | 0.0074 | 0.7468 |
| rs78378222 | ALM | Type 2 diabetes | 0.138 | 7571752 | 0.0087 | -0.0286 | 0.0279 | 0.3045 |
| rs78457529 | ALM | Type 2 diabetes | -0.0904 | 24950880 | 0.0088 | -0.0384 | 0.028 | 1.70E-01 |
| rs7858712 | ALM | Type 2 diabetes | 0.0347 | 16738312 | 0.0034 | -0.0238 | 0.0114 | 0.03703 |
| rs78766798 | ALM | Type 2 diabetes | 0.0319 | 7517075 | 0.0035 | 0.0045 | 0.0128 | 0.7246 |
| rs7893378 | ALM | Type 2 diabetes | 0.0175 | 93634095 | 0.0031 | 0.0015 | 0.0111 | 0.8925 |
| rs7902 | ALM | Type 2 diabetes | 0.0149 | 95565288 | 0.0019 | -0.0109 | 0.0064 | 0.08789 |
| rs7952436 | ALM | Type 2 diabetes | -0.0453 | 67024534 | 0.0034 | -0.0116 | 0.013 | 0.3716 |
| rs7971536 | ALM | Type 2 diabetes | -0.0194 | 102373788 | 0.0019 | 0.0134 | 0.0064 | 0.0359 |
| rs798548 | ALM | Type 2 diabetes | -0.0359 | 2760935 | 0.0021 | -0.005 | 0.007 | 0.476 |
| rs8000973 | ALM | Type 2 diabetes | 0.0134 | 100691367 | 0.0019 | -0.0034 | 0.0064 | 0.5945 |
| rs80132799 | ALM | Type 2 diabetes | 0.0231 | 62322896 | 0.0038 | -0.0094 | 0.0135 | 0.4865 |
| rs8018486 | ALM | Type 2 diabetes | -0.0138 | 39818616 | 0.0024 | 0.0242 | 0.008 | 0.002356 |
| rs8019890 | ALM | Type 2 diabetes | 0.025 | 21538067 | 0.0019 | -0.0168 | 0.0066 | 0.01087 |
| rs8020095 | ALM | Type 2 diabetes | -0.0145 | 67453858 | 0.0027 | -0.0154 | 0.0089 | 8.36E-02 |
| rs80280630 | ALM | Type 2 diabetes | -0.0168 | 117030861 | 0.003 | -0.013 | 0.0107 | 0.2235 |
| rs80295797 | ALM | Type 2 diabetes | -0.0198 | 23341690 | 0.002 | 0.001 | 0.0068 | 0.8832 |
| rs8042578 | ALM | Type 2 diabetes | 0.0287 | 66992964 | 0.0022 | -0.0097 | 0.0073 | 1.86E-01 |
| rs8054549 | ALM | Type 2 diabetes | -0.0251 | 86417234 | 0.0019 | 0.008 | 0.0064 | 0.2104 |
| rs8084413 | ALM | Type 2 diabetes | -0.0127 | 22869123 | 0.0019 | 0.0052 | 0.0065 | 0.4231 |
| rs8136517 | ALM | Type 2 diabetes | 0.0267 | 46439433 | 0.0039 | 0.0022 | 0.0139 | 8.75E-01 |
| rs822530 | ALM | Type 2 diabetes | 0.0255 | 148631555 | 0.0024 | -0.0053 | 0.0081 | 0.5109 |
| rs839255 | ALM | Type 2 diabetes | -0.0126 | 57974580 | 0.0021 | 0.0115 | 0.0069 | 0.09608 |
| rs861674 | ALM | Type 2 diabetes | 0.0128 | 112064475 | 0.0019 | 0.0151 | 0.0063 | 0.01623 |
| rs867529 | ALM | Type 2 diabetes | 0.0184 | 88913273 | 0.0021 | -0.0068 | 0.0071 | 0.3395 |
| rs876122 | ALM | Type 2 diabetes | 0.0162 | 6886297 | 0.0029 | 0.007 | 0.0097 | 0.4722 |
| rs8904 | ALM | Type 2 diabetes | -0.0157 | 35871217 | 0.002 | -0.0001 | 0.0066 | 0.9879 |
| rs900399 | ALM | Type 2 diabetes | 0.0164 | 156798732 | 0.0019 | 0.0139 | 0.0065 | 0.03225 |
| rs905938 | ALM | Type 2 diabetes | 0.0394 | 154991389 | 0.0021 | -0.0161 | 0.0072 | 2.58E-02 |
| rs909220 | ALM | Type 2 diabetes | -0.015 | 75908780 | 0.0019 | -0.0072 | 0.0064 | 2.60E-01 |
| rs9343327 | ALM | Type 2 diabetes | 0.014 | 76606296 | 0.0019 | -0.0133 | 0.0064 | 0.03731 |
| rs9344126 | ALM | Type 2 diabetes | -0.0185 | 81907559 | 0.0019 | -0.0137 | 0.0064 | 3.20E-02 |
| rs9375188 | ALM | Type 2 diabetes | 0.0136 | 98555272 | 0.0019 | -0.024 | 0.0064 | 0.0001715 |
| rs9385002 | ALM | Type 2 diabetes | -0.0147 | 117552469 | 0.0022 | -0.0147 | 0.0074 | 0.04799 |
| rs9388490 | ALM | Type 2 diabetes | 0.0462 | 126704795 | 0.0019 | 0.0503 | 0.0064 | 3.392E-15 |
| rs9391254 | ALM | Type 2 diabetes | 0.0166 | 105377347 | 0.002 | 0.0049 | 0.007 | 0.4849 |
| rs947099 | ALM | Type 2 diabetes | 0.0117 | 31129883 | 0.002 | 0.007 | 0.0067 | 0.2962 |
| rs951366 | ALM | Type 2 diabetes | 0.0205 | 205685352 | 0.0019 | 0.0216 | 0.0065 | 0.0008766 |
| rs9517483 | ALM | Type 2 diabetes | -0.0181 | 99572712 | 0.0021 | -0.003 | 0.0069 | 0.6642 |
| rs9525326 | ALM | Type 2 diabetes | -0.0184 | 115075715 | 0.0024 | -0.0102 | 0.0088 | 0.2462 |
| rs9568031 | ALM | Type 2 diabetes | -0.0115 | 48897520 | 0.0021 | -0.0017 | 0.007 | 0.8085 |
| rs9590328 | ALM | Type 2 diabetes | 0.0153 | 96448383 | 0.0027 | 0.0152 | 0.0092 | 0.099 |
| rs9594714 | ALM | Type 2 diabetes | 0.0144 | 42800481 | 0.0021 | -0.0068 | 0.0069 | 0.3251 |
| rs9610447 | ALM | Type 2 diabetes | 0.0152 | 20768891 | 0.0022 | -0.0113 | 0.0074 | 0.1285 |
| rs963317 | ALM | Type 2 diabetes | -0.0136 | 45129970 | 0.002 | 0.0156 | 0.0067 | 0.01991 |
| rs9634212 | ALM | Type 2 diabetes | 0.0471 | 93993266 | 0.0023 | 0.0239 | 0.0076 | 0.001766 |
| rs9636364 | ALM | Type 2 diabetes | 0.011 | 111992435 | 0.0019 | -0.0134 | 0.0064 | 0.0359 |
| rs9640283 | ALM | Type 2 diabetes | -0.0119 | 150485659 | 0.0019 | -0.0125 | 0.0071 | 0.07914 |
| rs9647379 | ALM | Type 2 diabetes | 0.0215 | 171785168 | 0.0019 | 0.0212 | 0.0066 | 0.001309 |
| rs9669278 | ALM | Type 2 diabetes | -0.0496 | 66374587 | 0.0019 | 0.0538 | 0.0064 | 3.652E-17 |
| rs9809116 | ALM | Type 2 diabetes | -0.016 | 72397279 | 0.0019 | 0.0007 | 0.0065 | 0.9141 |
| rs9828525 | ALM | Type 2 diabetes | 0.0121 | 61552810 | 0.0019 | -0.0026 | 0.0065 | 0.6888 |
| rs9832919 | ALM | Type 2 diabetes | -0.0179 | 132184526 | 0.002 | -0.0079 | 0.0066 | 0.2311 |
| rs9838614 | ALM | Type 2 diabetes | -0.0185 | 38537671 | 0.0019 | 0 | 0.0065 | 1 |
| rs985136 | ALM | Type 2 diabetes | 0.0138 | 17497794 | 0.002 | 0.002 | 0.0067 | 0.7653 |
| rs987666 | ALM | Type 2 diabetes | 0.0185 | 116267938 | 0.0029 | -0.0234 | 0.0099 | 0.01865 |
| rs9894577 | ALM | Type 2 diabetes | -0.031 | 43223292 | 0.002 | 0.0187 | 0.0068 | 0.006001 |
| rs9898189 | ALM | Type 2 diabetes | -0.0163 | 80480516 | 0.0021 | -0.0139 | 0.0068 | 0.04111 |
| rs990315 | ALM | Type 2 diabetes | -0.0115 | 69578811 | 0.002 | -0.0061 | 0.0066 | 0.3551 |
| rs9905385 | ALM | Type 2 diabetes | -0.0339 | 59498250 | 0.002 | -0.0089 | 0.0068 | 0.191 |
| rs9957318 | ALM | Type 2 diabetes | 0.0187 | 33039106 | 0.002 | -0.0011 | 0.0066 | 0.8676 |

**Supplementary Table 3.** Genome-wide significant SNPs for Walking pace

| **SNP** | **exposure** | **outcome** | **beta.exposure** | **se.exposure** | **pval.exposure** | **beta.outcome** | **se.outcome** | **pval.outcome** |
| --- | --- | --- | --- | --- | --- | --- | --- | --- |
| rs10750025 | Usual walking pace | Fasting glucose | -0.0083554 | 459915 | 113424042 | 0.0008 | 0.002 | 0.227 |
| rs10828258 | Usual walking pace | Fasting glucose | -0.00932641 | 459915 | 21929734 | -0.0046 | 0.002 | 0.00833297 |
| rs10862220 | Usual walking pace | Fasting glucose | 0.00840767 | 459915 | 81430599 | 0.0025 | 0.0019 | 0.169 |
| rs10883618 | Usual walking pace | Fasting glucose | 0.0078103 | 459915 | 103117653 | 0.0022 | 0.0019 | 0.4554 |
| rs11039324 | Usual walking pace | Fasting glucose | -0.0102105 | 459915 | 47665686 | -0.0006 | 0.0017 | 0.9537 |
| rs11077815 | Usual walking pace | Fasting glucose | -0.00713336 | 459915 | 74389890 | 0.0001 | 0.0019 | 0.9793 |
| rs11150623 | Usual walking pace | Fasting glucose | -0.00968813 | 459915 | 28881001 | -0.0036 | 0.0018 | 0.0485501 |
| rs11152989 | Usual walking pace | Fasting glucose | -0.00751226 | 459915 | 96936061 | -0.002 | 0.002 | 0.3494 |
| rs113825410 | Usual walking pace | Fasting glucose | -0.00881182 | 459915 | 40057543 | 0.0084 | 0.0022 | 8.77708E-05 |
| rs11548200 | Usual walking pace | Fasting glucose | -0.016041 | 459915 | 156290656 | 0.0001 | 0.0045 | 0.8186 |
| rs11682482 | Usual walking pace | Fasting glucose | 0.00801894 | 459915 | 226486479 | -0.0007 | 0.002 | 0.9597 |
| rs11732213 | Usual walking pace | Fasting glucose | 0.0091614 | 459915 | 1704244 | -0.002 | 0.0023 | 0.324 |
| rs11761141 | Usual walking pace | Fasting glucose | -0.00789936 | 459915 | 69423362 | 0.0003 | 0.0019 | 0.697 |
| rs11848096 | Usual walking pace | Fasting glucose | -0.00750831 | 459915 | 100969235 | -0.0041 | 0.0019 | 0.0101899 |
| rs11881338 | Usual walking pace | Fasting glucose | 0.00817022 | 459915 | 18838014 | 0.0013 | 0.0018 | 1 |
| rs12042959 | Usual walking pace | Fasting glucose | 0.0125663 | 459915 | 243533273 | -0.0004 | 0.0025 | 0.707801 |
| rs12461902 | Usual walking pace | Fasting glucose | -0.00801134 | 459915 | 30265235 | 0.001 | 0.002 | 0.704399 |
| rs12747822 | Usual walking pace | Fasting glucose | 0.0117683 | 459915 | 91201451 | -0.0071 | 0.0034 | 0.0275499 |
| rs12883788 | Usual walking pace | Fasting glucose | -0.00779959 | 459915 | 33303540 | 0.0026 | 0.0019 | 0.2757 |
| rs13107325 | Usual walking pace | Fasting glucose | -0.0242429 | 459915 | 103188709 | -0.0031 | 0.0038 | 0.3045 |
| rs139398785 | Usual walking pace | Fasting glucose | -0.00999513 | 459915 | 161132876 | 0.0042 | 0.0027 | 0.1206 |
| rs144333966 | Usual walking pace | Fasting glucose | 0.0304614 | 459915 | 61109385 | -0.0031 | 0.0118 | 0.735099 |
| rs1592 | Usual walking pace | Fasting glucose | 0.0074513 | 459915 | 135722143 | -0.0037 | 0.0019 | 0.06925 |
| rs2037735 | Usual walking pace | Fasting glucose | -0.0112677 | 459915 | 48012469 | 0.0021 | 0.0027 | 0.392 |
| rs205262 | Usual walking pace | Fasting glucose | -0.00870949 | 459915 | 34563164 | -0.0029 | 0.0019 | 0.0658795 |
| rs2170670 | Usual walking pace | Fasting glucose | -0.00708769 | 459915 | 16944621 | 0.0006 | 0.0019 | 0.6893 |
| rs2280406 | Usual walking pace | Fasting glucose | -0.00996145 | 459915 | 49941436 | -0.0001 | 0.0018 | 0.9345 |
| rs2297600 | Usual walking pace | Fasting glucose | -0.0115607 | 459915 | 32207581 | 0.0059 | 0.0024 | 0.00487798 |
| rs2439823 | Usual walking pace | Fasting glucose | -0.0072789 | 459915 | 99778226 | -0.001 | 0.0018 | 0.8694 |
| rs2602731 | Usual walking pace | Fasting glucose | -0.00766675 | 459915 | 4944771 | 0.0029 | 0.002 | 0.2366 |
| rs2644135 | Usual walking pace | Fasting glucose | 0.00751192 | 459915 | 201856256 | -0.0006 | 0.002 | 0.8794 |
| rs2645979 | Usual walking pace | Fasting glucose | 0.00861798 | 459915 | 84017043 | -0.0007 | 0.0019 | 0.9575 |
| rs273512 | Usual walking pace | Fasting glucose | -0.00966507 | 459915 | 18224729 | -0.0045 | 0.0019 | 0.0234698 |
| rs28519617 | Usual walking pace | Fasting glucose | -0.00812933 | 459915 | 135874930 | -0.0018 | 0.0021 | 0.6386 |
| rs35711462 | Usual walking pace | Fasting glucose | -0.0071659 | 459915 | 50847577 | -0.0043 | 0.0018 | 0.0147601 |
| rs4109292 | Usual walking pace | Fasting glucose | 0.00735336 | 459915 | 126710654 | -0.0002 | 0.0018 | 1 |
| rs4516268 | Usual walking pace | Fasting glucose | 0.00983008 | 459915 | 1846831 | -0.0021 | 0.0021 | 0.324 |
| rs45583845 | Usual walking pace | Fasting glucose | -0.0198998 | 459915 | 57858194 | -0.0031 | 0.0077 | 0.8498 |
| rs4643373 | Usual walking pace | Fasting glucose | 0.0077266 | 459915 | 47123423 | -0.0047 | 0.0021 | 0.0573905 |
| rs4715208 | Usual walking pace | Fasting glucose | -0.0083844 | 459915 | 50829471 | -0.0032 | 0.0019 | 0.4395 |
| rs4839898 | Usual walking pace | Fasting glucose | 0.0130564 | 459915 | 97546759 | -0.001 | 0.0034 | 0.6709 |
| rs55680124 | Usual walking pace | Fasting glucose | -0.010742 | 459915 | 105984624 | -0.0009 | 0.0026 | 0.9697 |
| rs57800857 | Usual walking pace | Fasting glucose | 0.00867607 | 459915 | 140863365 | -0.0029 | 0.0019 | 0.0429299 |
| rs613872 | Usual walking pace | Fasting glucose | -0.0147074 | 459915 | 53210302 | -0.0017 | 0.0024 | 0.5513 |
| rs62048402 | Usual walking pace | Fasting glucose | -0.00987537 | 459915 | 53803223 | -0.002 | 0.0017 | 0.0452397 |
| rs6763292 | Usual walking pace | Fasting glucose | 0.00958833 | 459915 | 129044705 | -0.0018 | 0.0022 | 0.3465 |
| rs7789719 | Usual walking pace | Fasting glucose | 0.00854199 | 459915 | 66893916 | 0.0007 | 0.0022 | 0.3354 |
| rs7795394 | Usual walking pace | Fasting glucose | 0.00930493 | 459915 | 113560607 | -0.0004 | 0.0019 | 0.8355 |
| rs7896518 | Usual walking pace | Fasting glucose | 0.00999095 | 459915 | 65104500 | -0.0062 | 0.0017 | 0.000287899 |
| rs8010773 | Usual walking pace | Fasting glucose | -0.00816754 | 459915 | 46956863 | -0.0005 | 0.0019 | 0.8558 |
| rs8011870 | Usual walking pace | Fasting glucose | -0.00782739 | 459915 | 80173397 | -0.0019 | 0.002 | 0.9808 |
| rs8028757 | Usual walking pace | Fasting glucose | 0.0107284 | 459915 | 75822794 | -0.0049 | 0.0036 | 0.147 |
| rs819167 | Usual walking pace | Fasting glucose | -0.0157993 | 459915 | 32903687 | -0.009 | 0.0036 | 0.00626902 |
| rs830627 | Usual walking pace | Fasting glucose | 0.00742054 | 459915 | 71675270 | 0.0018 | 0.0019 | 0.5052 |
| rs891387 | Usual walking pace | Fasting glucose | 0.00786028 | 459915 | 21103909 | -0.0016 | 0.0018 | 0.5837 |
| rs9366651 | Usual walking pace | Fasting glucose | 0.00948542 | 459915 | 26336696 | -0.0023 | 0.0018 | 0.2366 |
| rs9783304 | Usual walking pace | Fasting glucose | 0.00763598 | 459915 | 43660255 | 0.0021 | 0.0019 | 0.1169 |
| rs10750025 | Usual walking pace | Fasting insulin | -0.0083554 | 459915 | 113424042 | 0.0022 | 0.0023 | 0.3361 |
| rs10828258 | Usual walking pace | Fasting insulin | -0.00932641 | 459915 | 21929734 | -0.0002 | 0.0022 | 0.776499 |
| rs10862220 | Usual walking pace | Fasting insulin | 0.00840767 | 459915 | 81430599 | 0.0017 | 0.0021 | 0.6074 |
| rs10883618 | Usual walking pace | Fasting insulin | 0.0078103 | 459915 | 103117653 | 0.0012 | 0.0021 | 0.8192 |
| rs11039324 | Usual walking pace | Fasting insulin | -0.0102105 | 459915 | 47665686 | -0.007 | 0.0019 | 0.0002848 |
| rs11077815 | Usual walking pace | Fasting insulin | -0.00713336 | 459915 | 74389890 | -0.0007 | 0.0021 | 0.7208 |
| rs11150623 | Usual walking pace | Fasting insulin | -0.00968813 | 459915 | 28881001 | 0.0015 | 0.0021 | 0.9254 |
| rs11152989 | Usual walking pace | Fasting insulin | -0.00751226 | 459915 | 96936061 | 0.0014 | 0.0022 | 0.2471 |
| rs113825410 | Usual walking pace | Fasting insulin | -0.00881182 | 459915 | 40057543 | 0.0067 | 0.0025 | 0.00751398 |
| rs11548200 | Usual walking pace | Fasting insulin | -0.016041 | 459915 | 156290656 | 0.0004 | 0.0052 | 0.9534 |
| rs11682482 | Usual walking pace | Fasting insulin | 0.00801894 | 459915 | 226486479 | 0.0041 | 0.0022 | 0.0926403 |
| rs11732213 | Usual walking pace | Fasting insulin | 0.0091614 | 459915 | 1704244 | 0.0001 | 0.0027 | 0.7526 |
| rs11761141 | Usual walking pace | Fasting insulin | -0.00789936 | 459915 | 69423362 | 0.0037 | 0.0022 | 0.0289701 |
| rs11848096 | Usual walking pace | Fasting insulin | -0.00750831 | 459915 | 100969235 | 0.0001 | 0.0022 | 0.776499 |
| rs11881338 | Usual walking pace | Fasting insulin | 0.00817022 | 459915 | 18838014 | 0.0021 | 0.002 | 0.4682 |
| rs12042959 | Usual walking pace | Fasting insulin | 0.0125663 | 459915 | 243533273 | 0.0032 | 0.0029 | 0.2563 |
| rs12461902 | Usual walking pace | Fasting insulin | -0.00801134 | 459915 | 30265235 | -0.0007 | 0.0022 | 0.646501 |
| rs12747822 | Usual walking pace | Fasting insulin | 0.0117683 | 459915 | 91201451 | -0.0059 | 0.0039 | 0.2084 |
| rs12883788 | Usual walking pace | Fasting insulin | -0.00779959 | 459915 | 33303540 | -0.0013 | 0.0022 | 0.4317 |
| rs13107325 | Usual walking pace | Fasting insulin | -0.0242429 | 459915 | 103188709 | -0.0102 | 0.0044 | 0.00937303 |
| rs139398785 | Usual walking pace | Fasting insulin | -0.00999513 | 459915 | 161132876 | 0.0071 | 0.0032 | 0.0475697 |
| rs144333966 | Usual walking pace | Fasting insulin | 0.0304614 | 459915 | 61109385 | 0.0022 | 0.0151 | 0.8214 |
| rs1592 | Usual walking pace | Fasting insulin | 0.0074513 | 459915 | 135722143 | -0.0059 | 0.0022 | 0.01279 |
| rs2037735 | Usual walking pace | Fasting insulin | -0.0112677 | 459915 | 48012469 | 0.0003 | 0.0031 | 0.5469 |
| rs205262 | Usual walking pace | Fasting insulin | -0.00870949 | 459915 | 34563164 | -0.0045 | 0.0021 | 0.0354903 |
| rs2170670 | Usual walking pace | Fasting insulin | -0.00708769 | 459915 | 16944621 | 0.0009 | 0.0021 | 0.4682 |
| rs2280406 | Usual walking pace | Fasting insulin | -0.00996145 | 459915 | 49941436 | 0.0056 | 0.002 | 0.0218399 |
| rs2297600 | Usual walking pace | Fasting insulin | -0.0115607 | 459915 | 32207581 | 0.0032 | 0.0028 | 0.3886 |
| rs2439823 | Usual walking pace | Fasting insulin | -0.0072789 | 459915 | 99778226 | -0.0003 | 0.002 | 0.778901 |
| rs2602731 | Usual walking pace | Fasting insulin | -0.00766675 | 459915 | 4944771 | 0.0066 | 0.0023 | 0.00791699 |
| rs2644135 | Usual walking pace | Fasting insulin | 0.00751192 | 459915 | 201856256 | 0.0027 | 0.0022 | 0.121 |
| rs2645979 | Usual walking pace | Fasting insulin | 0.00861798 | 459915 | 84017043 | -0.0036 | 0.0021 | 0.2193 |
| rs273512 | Usual walking pace | Fasting insulin | -0.00966507 | 459915 | 18224729 | 0.0035 | 0.0022 | 0.1828 |
| rs28519617 | Usual walking pace | Fasting insulin | -0.00812933 | 459915 | 135874930 | 0.0091 | 0.0025 | 0.000845805 |
| rs35711462 | Usual walking pace | Fasting insulin | -0.0071659 | 459915 | 50847577 | 0.0008 | 0.002 | 0.944 |
| rs4109292 | Usual walking pace | Fasting insulin | 0.00735336 | 459915 | 126710654 | -0.001 | 0.002 | 0.778901 |
| rs4516268 | Usual walking pace | Fasting insulin | 0.00983008 | 459915 | 1846831 | -0.0033 | 0.0025 | 0.1879 |
| rs45583845 | Usual walking pace | Fasting insulin | -0.0198998 | 459915 | 57858194 | -0.0068 | 0.0094 | 0.2654 |
| rs4643373 | Usual walking pace | Fasting insulin | 0.0077266 | 459915 | 47123423 | 0.002 | 0.0023 | 0.1948 |
| rs4715208 | Usual walking pace | Fasting insulin | -0.0083844 | 459915 | 50829471 | -0.0006 | 0.0021 | 0.664099 |
| rs4839898 | Usual walking pace | Fasting insulin | 0.0130564 | 459915 | 97546759 | -0.0023 | 0.0039 | 0.3916 |
| rs55680124 | Usual walking pace | Fasting insulin | -0.010742 | 459915 | 105984624 | -0.0041 | 0.003 | 0.1404 |
| rs57800857 | Usual walking pace | Fasting insulin | 0.00867607 | 459915 | 140863365 | 0.0016 | 0.0021 | 0.655101 |
| rs613872 | Usual walking pace | Fasting insulin | -0.0147074 | 459915 | 53210302 | 0.0012 | 0.0028 | 0.7256 |
| rs62048402 | Usual walking pace | Fasting insulin | -0.00987537 | 459915 | 53803223 | -0.0027 | 0.0019 | 0.2661 |
| rs6763292 | Usual walking pace | Fasting insulin | 0.00958833 | 459915 | 129044705 | -0.0048 | 0.0026 | 0.1232 |
| rs7789719 | Usual walking pace | Fasting insulin | 0.00854199 | 459915 | 66893916 | -0.0005 | 0.0025 | 0.6798 |
| rs7795394 | Usual walking pace | Fasting insulin | 0.00930493 | 459915 | 113560607 | 0.0027 | 0.0022 | 0.0739605 |
| rs7896518 | Usual walking pace | Fasting insulin | 0.00999095 | 459915 | 65104500 | -0.0063 | 0.0019 | 0.000506104 |
| rs8010773 | Usual walking pace | Fasting insulin | -0.00816754 | 459915 | 46956863 | -0.0027 | 0.0021 | 0.3146 |
| rs8011870 | Usual walking pace | Fasting insulin | -0.00782739 | 459915 | 80173397 | 0.0002 | 0.0023 | 0.8507 |
| rs8028757 | Usual walking pace | Fasting insulin | 0.0107284 | 459915 | 75822794 | 0.003 | 0.004 | 0.5177 |
| rs819167 | Usual walking pace | Fasting insulin | -0.0157993 | 459915 | 32903687 | 0.0032 | 0.0039 | 0.2286 |
| rs830627 | Usual walking pace | Fasting insulin | 0.00742054 | 459915 | 71675270 | -0.0007 | 0.0021 | 0.766399 |
| rs891387 | Usual walking pace | Fasting insulin | 0.00786028 | 459915 | 21103909 | -0.0048 | 0.002 | 0.0144901 |
| rs9366651 | Usual walking pace | Fasting insulin | 0.00948542 | 459915 | 26336696 | -0.0018 | 0.002 | 0.6406 |
| rs9783304 | Usual walking pace | Fasting insulin | 0.00763598 | 459915 | 43660255 | 0.0018 | 0.0022 | 0.7268 |
| rs10750025 | Usual walking pace | HbA1C | -0.0083554 | 459915 | 113424042 | -0.0023 | 0.0038 | 0.5438 |
| rs10828258 | Usual walking pace | HbA1C | -0.00932641 | 459915 | 21929734 | -0.0061 | 0.0037 | 0.0964406 |
| rs10862220 | Usual walking pace | HbA1C | 0.00840767 | 459915 | 81430599 | 0.0002 | 0.0036 | 0.946 |
| rs10883618 | Usual walking pace | HbA1C | 0.0078103 | 459915 | 103117653 | -0.0005 | 0.0035 | 0.8976 |
| rs11077815 | Usual walking pace | HbA1C | -0.00713336 | 459915 | 74389890 | 0.0036 | 0.0035 | 0.3148 |
| rs11150623 | Usual walking pace | HbA1C | -0.00968813 | 459915 | 28881001 | 0.011 | 0.0059 | 0.0639897 |
| rs113825410 | Usual walking pace | HbA1C | -0.00881182 | 459915 | 40057543 | 0.009 | 0.0041 | 0.0288702 |
| rs11682482 | Usual walking pace | HbA1C | 0.00801894 | 459915 | 226486479 | -0.0053 | 0.0037 | 0.1499 |
| rs11732213 | Usual walking pace | HbA1C | 0.0091614 | 459915 | 1704244 | -0.0123 | 0.0069 | 0.0764293 |
| rs11761141 | Usual walking pace | HbA1C | -0.00789936 | 459915 | 69423362 | 0.001 | 0.0036 | 0.7855 |
| rs11848096 | Usual walking pace | HbA1C | -0.00750831 | 459915 | 100969235 | -0.0048 | 0.0044 | 0.2826 |
| rs12042959 | Usual walking pace | HbA1C | 0.0125663 | 459915 | 243533273 | -0.0069 | 0.0047 | 0.1406 |
| rs12461902 | Usual walking pace | HbA1C | -0.00801134 | 459915 | 30265235 | 0.0025 | 0.0038 | 0.512201 |
| rs12747822 | Usual walking pace | HbA1C | 0.0117683 | 459915 | 91201451 | -0.007 | 0.0067 | 0.2956 |
| rs12883788 | Usual walking pace | HbA1C | -0.00779959 | 459915 | 33303540 | 0.0006 | 0.0041 | 0.8765 |
| rs13107325 | Usual walking pace | HbA1C | -0.0242429 | 459915 | 103188709 | 0.0013 | 0.007 | 0.8555 |
| rs139398785 | Usual walking pace | HbA1C | -0.00999513 | 459915 | 161132876 | 0.0062 | 0.0052 | 0.2295 |
| rs1592 | Usual walking pace | HbA1C | 0.0074513 | 459915 | 135722143 | -0.0034 | 0.0035 | 0.3206 |
| rs2037735 | Usual walking pace | HbA1C | -0.0112677 | 459915 | 48012469 | -0.0047 | 0.0052 | 0.3595 |
| rs205262 | Usual walking pace | HbA1C | -0.00870949 | 459915 | 34563164 | -0.0039 | 0.0038 | 0.312 |
| rs2170670 | Usual walking pace | HbA1C | -0.00708769 | 459915 | 16944621 | 0.0019 | 0.0035 | 0.593401 |
| rs2280406 | Usual walking pace | HbA1C | -0.00996145 | 459915 | 49941436 | -0.001 | 0.0035 | 0.779301 |
| rs2439823 | Usual walking pace | HbA1C | -0.0072789 | 459915 | 99778226 | -0.0004 | 0.0034 | 0.9089 |
| rs2602731 | Usual walking pace | HbA1C | -0.00766675 | 459915 | 4944771 | -0.0146 | 0.004 | 0.000297502 |
| rs2644135 | Usual walking pace | HbA1C | 0.00751192 | 459915 | 201856256 | -0.0016 | 0.0036 | 0.6643 |
| rs2645979 | Usual walking pace | HbA1C | 0.00861798 | 459915 | 84017043 | -0.0019 | 0.0035 | 0.583001 |
| rs273512 | Usual walking pace | HbA1C | -0.00966507 | 459915 | 18224729 | -0.0033 | 0.0039 | 0.3889 |
| rs28519617 | Usual walking pace | HbA1C | -0.00812933 | 459915 | 135874930 | -0.0044 | 0.0038 | 0.246 |
| rs35711462 | Usual walking pace | HbA1C | -0.0071659 | 459915 | 50847577 | 0.0042 | 0.0034 | 0.2185 |
| rs4109292 | Usual walking pace | HbA1C | 0.00735336 | 459915 | 126710654 | -0.0007 | 0.0034 | 0.8276 |
| rs4516268 | Usual walking pace | HbA1C | 0.00983008 | 459915 | 1846831 | -0.0059 | 0.0055 | 0.2847 |
| rs4643373 | Usual walking pace | HbA1C | 0.0077266 | 459915 | 47123423 | -0.0075 | 0.0038 | 0.0471303 |
| rs4715208 | Usual walking pace | HbA1C | -0.0083844 | 459915 | 50829471 | 0.0042 | 0.0039 | 0.2833 |
| rs4839898 | Usual walking pace | HbA1C | 0.0130564 | 459915 | 97546759 | -0.0059 | 0.0059 | 0.321 |
| rs57800857 | Usual walking pace | HbA1C | 0.00867607 | 459915 | 140863365 | 0.0013 | 0.008 | 0.872 |
| rs613872 | Usual walking pace | HbA1C | -0.0147074 | 459915 | 53210302 | -0.0022 | 0.0045 | 0.6295 |
| rs62048402 | Usual walking pace | HbA1C | -0.00987537 | 459915 | 53803223 | 0.0064 | 0.0034 | 0.0625893 |
| rs6763292 | Usual walking pace | HbA1C | 0.00958833 | 459915 | 129044705 | -0.0106 | 0.0042 | 0.0124 |
| rs7789719 | Usual walking pace | HbA1C | 0.00854199 | 459915 | 66893916 | -0.0025 | 0.0041 | 0.5309 |
| rs7795394 | Usual walking pace | HbA1C | 0.00930493 | 459915 | 113560607 | -0.0032 | 0.0036 | 0.3694 |
| rs7896518 | Usual walking pace | HbA1C | 0.00999095 | 459915 | 65104500 | -0.002 | 0.0034 | 0.560701 |
| rs8010773 | Usual walking pace | HbA1C | -0.00816754 | 459915 | 46956863 | 0.0017 | 0.0035 | 0.6294 |
| rs8011870 | Usual walking pace | HbA1C | -0.00782739 | 459915 | 80173397 | 0.0002 | 0.0041 | 0.9614 |
| rs819167 | Usual walking pace | HbA1C | -0.0157993 | 459915 | 32903687 | -0.0073 | 0.0068 | 0.2834 |
| rs830627 | Usual walking pace | HbA1C | 0.00742054 | 459915 | 71675270 | 0.0032 | 0.0034 | 0.3513 |
| rs891387 | Usual walking pace | HbA1C | 0.00786028 | 459915 | 21103909 | -0.0017 | 0.0034 | 0.6111 |
| rs9783304 | Usual walking pace | HbA1C | 0.00763598 | 459915 | 43660255 | -0.0029 | 0.0037 | 0.4202 |
| rs10750025 | Usual walking pace | Two-hour glucose challenge | -0.0083554 | 459915 | 113424042 | -0.027 | 0.022 | 0.2145 |
| rs10828258 | Usual walking pace | Two-hour glucose challenge | -0.00932641 | 459915 | 21929734 | -0.043 | 0.02 | 0.0319499 |
| rs10862220 | Usual walking pace | Two-hour glucose challenge | 0.00840767 | 459915 | 81430599 | -0.019 | 0.019 | 0.3215 |
| rs10883618 | Usual walking pace | Two-hour glucose challenge | 0.0078103 | 459915 | 103117653 | -0.037 | 0.019 | 0.0497496 |
| rs11077815 | Usual walking pace | Two-hour glucose challenge | -0.00713336 | 459915 | 74389890 | -0.012 | 0.02 | 0.5377 |
| rs113825410 | Usual walking pace | Two-hour glucose challenge | -0.00881182 | 459915 | 40057543 | 0.02 | 0.022 | 0.38 |
| rs11682482 | Usual walking pace | Two-hour glucose challenge | 0.00801894 | 459915 | 226486479 | -0.022 | 0.02 | 0.2627 |
| rs11732213 | Usual walking pace | Two-hour glucose challenge | 0.0091614 | 459915 | 1704244 | 0.0019 | 0.023 | 0.9337 |
| rs11761141 | Usual walking pace | Two-hour glucose challenge | -0.00789936 | 459915 | 69423362 | 0.027 | 0.02 | 0.1673 |
| rs11848096 | Usual walking pace | Two-hour glucose challenge | -0.00750831 | 459915 | 100969235 | -0.0084 | 0.022 | 0.7006 |
| rs12042959 | Usual walking pace | Two-hour glucose challenge | 0.0125663 | 459915 | 243533273 | -0.0092 | 0.026 | 0.723 |
| rs12461902 | Usual walking pace | Two-hour glucose challenge | -0.00801134 | 459915 | 30265235 | -0.0084 | 0.021 | 0.6894 |
| rs12747822 | Usual walking pace | Two-hour glucose challenge | 0.0117683 | 459915 | 91201451 | 0.054 | 0.039 | 0.1686 |
| rs12883788 | Usual walking pace | Two-hour glucose challenge | -0.00779959 | 459915 | 33303540 | 0.042 | 0.021 | 0.0523094 |
| rs13107325 | Usual walking pace | Two-hour glucose challenge | -0.0242429 | 459915 | 103188709 | 0.025 | 0.04 | 0.5374 |
| rs139398785 | Usual walking pace | Two-hour glucose challenge | -0.00999513 | 459915 | 161132876 | 0.0038 | 0.027 | 0.8902 |
| rs1592 | Usual walking pace | Two-hour glucose challenge | 0.0074513 | 459915 | 135722143 | -0.0017 | 0.019 | 0.9275 |
| rs2037735 | Usual walking pace | Two-hour glucose challenge | -0.0112677 | 459915 | 48012469 | -0.019 | 0.027 | 0.4936 |
| rs205262 | Usual walking pace | Two-hour glucose challenge | -0.00870949 | 459915 | 34563164 | -0.034 | 0.022 | 0.1177 |
| rs2170670 | Usual walking pace | Two-hour glucose challenge | -0.00708769 | 459915 | 16944621 | -0.042 | 0.019 | 0.0280802 |
| rs2280406 | Usual walking pace | Two-hour glucose challenge | -0.00996145 | 459915 | 49941436 | -0.0091 | 0.019 | 0.6349 |
| rs2439823 | Usual walking pace | Two-hour glucose challenge | -0.0072789 | 459915 | 99778226 | 0.0045 | 0.018 | 0.8062 |
| rs2602731 | Usual walking pace | Two-hour glucose challenge | -0.00766675 | 459915 | 4944771 | -0.025 | 0.022 | 0.2551 |
| rs2644135 | Usual walking pace | Two-hour glucose challenge | 0.00751192 | 459915 | 201856256 | 0.044 | 0.02 | 0.02553 |
| rs2645979 | Usual walking pace | Two-hour glucose challenge | 0.00861798 | 459915 | 84017043 | 0.0007 | 0.019 | 0.9692 |
| rs273512 | Usual walking pace | Two-hour glucose challenge | -0.00966507 | 459915 | 18224729 | 0.015 | 0.021 | 0.4508 |
| rs28519617 | Usual walking pace | Two-hour glucose challenge | -0.00812933 | 459915 | 135874930 | 0.04 | 0.021 | 0.0588207 |
| rs35711462 | Usual walking pace | Two-hour glucose challenge | -0.0071659 | 459915 | 50847577 | -0.027 | 0.018 | 0.1482 |
| rs4109292 | Usual walking pace | Two-hour glucose challenge | 0.00735336 | 459915 | 126710654 | 0.022 | 0.019 | 0.2543 |
| rs4516268 | Usual walking pace | Two-hour glucose challenge | 0.00983008 | 459915 | 1846831 | 0.036 | 0.028 | 0.2 |
| rs4643373 | Usual walking pace | Two-hour glucose challenge | 0.0077266 | 459915 | 47123423 | 0.0075 | 0.02 | 0.7113 |
| rs4715208 | Usual walking pace | Two-hour glucose challenge | -0.0083844 | 459915 | 50829471 | 0.008 | 0.022 | 0.716401 |
| rs4839898 | Usual walking pace | Two-hour glucose challenge | 0.0130564 | 459915 | 97546759 | 0.011 | 0.031 | 0.7272 |
| rs57800857 | Usual walking pace | Two-hour glucose challenge | 0.00867607 | 459915 | 140863365 | 0.011 | 0.019 | 0.5743 |
| rs613872 | Usual walking pace | Two-hour glucose challenge | -0.0147074 | 459915 | 53210302 | -0.0016 | 0.025 | 0.948 |
| rs62048402 | Usual walking pace | Two-hour glucose challenge | -0.00987537 | 459915 | 53803223 | -0.006 | 0.019 | 0.752101 |
| rs6763292 | Usual walking pace | Two-hour glucose challenge | 0.00958833 | 459915 | 129044705 | -0.048 | 0.023 | 0.0397796 |
| rs7789719 | Usual walking pace | Two-hour glucose challenge | 0.00854199 | 459915 | 66893916 | -0.049 | 0.022 | 0.0267898 |
| rs7795394 | Usual walking pace | Two-hour glucose challenge | 0.00930493 | 459915 | 113560607 | 0.018 | 0.02 | 0.372 |
| rs7896518 | Usual walking pace | Two-hour glucose challenge | 0.00999095 | 459915 | 65104500 | 0.035 | 0.019 | 0.0641594 |
| rs8010773 | Usual walking pace | Two-hour glucose challenge | -0.00816754 | 459915 | 46956863 | -0.0062 | 0.019 | 0.746799 |
| rs8011870 | Usual walking pace | Two-hour glucose challenge | -0.00782739 | 459915 | 80173397 | 0.032 | 0.021 | 0.1344 |
| rs819167 | Usual walking pace | Two-hour glucose challenge | -0.0157993 | 459915 | 32903687 | 0.048 | 0.037 | 0.1873 |
| rs830627 | Usual walking pace | Two-hour glucose challenge | 0.00742054 | 459915 | 71675270 | 0.029 | 0.019 | 0.1151 |
| rs891387 | Usual walking pace | Two-hour glucose challenge | 0.00786028 | 459915 | 21103909 | 0.013 | 0.018 | 0.4715 |
| rs9783304 | Usual walking pace | Two-hour glucose challenge | 0.00763598 | 459915 | 43660255 | -0.019 | 0.02 | 0.3336 |
| rs10750025 | Usual walking pace | Type 2 diabetes | -0.0083554 | 459915 | 113424042 | 0.0047 | 0.007 | 0.5029 |
| rs10828258 | Usual walking pace | Type 2 diabetes | -0.00932641 | 459915 | 21929734 | -0.0005 | 0.0068 | 0.9414 |
| rs10862220 | Usual walking pace | Type 2 diabetes | 0.00840767 | 459915 | 81430599 | -0.013 | 0.0068 | 0.05611 |
| rs10883618 | Usual walking pace | Type 2 diabetes | 0.0078103 | 459915 | 103117653 | -0.0233 | 0.0066 | 0.0004119 |
| rs11039324 | Usual walking pace | Type 2 diabetes | -0.0102105 | 459915 | 47665686 | 0.0336 | 0.0065 | 2.267E-07 |
| rs11077815 | Usual walking pace | Type 2 diabetes | -0.00713336 | 459915 | 74389890 | 0.0211 | 0.0066 | 0.00138 |
| rs11150623 | Usual walking pace | Type 2 diabetes | -0.00968813 | 459915 | 28881001 | 0.0255 | 0.0067 | 0.0001416 |
| rs11152989 | Usual walking pace | Type 2 diabetes | -0.00751226 | 459915 | 96936061 | 0.0121 | 0.0068 | 0.07541 |
| rs113825410 | Usual walking pace | Type 2 diabetes | -0.00881182 | 459915 | 40057543 | 0.0745 | 0.0075 | 4.958E-23 |
| rs11548200 | Usual walking pace | Type 2 diabetes | -0.016041 | 459915 | 156290656 | 0.0312 | 0.0136 | 0.02189 |
| rs11682482 | Usual walking pace | Type 2 diabetes | 0.00801894 | 459915 | 226486479 | 0.0076 | 0.007 | 0.2786 |
| rs11732213 | Usual walking pace | Type 2 diabetes | 0.0091614 | 459915 | 1704244 | 0.012 | 0.0081 | 0.1366 |
| rs11761141 | Usual walking pace | Type 2 diabetes | -0.00789936 | 459915 | 69423362 | 0.022 | 0.0068 | 0.001227 |
| rs11848096 | Usual walking pace | Type 2 diabetes | -0.00750831 | 459915 | 100969235 | 0.007 | 0.0066 | 0.2886 |
| rs11881338 | Usual walking pace | Type 2 diabetes | 0.00817022 | 459915 | 18838014 | -0.0215 | 0.0065 | 0.0009263 |
| rs12042959 | Usual walking pace | Type 2 diabetes | 0.0125663 | 459915 | 243533273 | -0.0244 | 0.0093 | 0.008833 |
| rs12461902 | Usual walking pace | Type 2 diabetes | -0.00801134 | 459915 | 30265235 | 0.0116 | 0.0068 | 0.08829 |
| rs12747822 | Usual walking pace | Type 2 diabetes | 0.0117683 | 459915 | 91201451 | -0.0099 | 0.011 | 0.3678 |
| rs12883788 | Usual walking pace | Type 2 diabetes | -0.00779959 | 459915 | 33303540 | 0.0349 | 0.0064 | 4.646E-08 |
| rs13107325 | Usual walking pace | Type 2 diabetes | -0.0242429 | 459915 | 103188709 | 0.046 | 0.0135 | 0.0006598 |
| rs139398785 | Usual walking pace | Type 2 diabetes | -0.00999513 | 459915 | 161132876 | 0.0424 | 0.0096 | 0.00001074 |
| rs144333966 | Usual walking pace | Type 2 diabetes | 0.0304614 | 459915 | 61109385 | 0.0026 | 0.025 | 0.9172 |
| rs1592 | Usual walking pace | Type 2 diabetes | 0.0074513 | 459915 | 135722143 | 0.0175 | 0.0071 | 0.01397 |
| rs2037735 | Usual walking pace | Type 2 diabetes | -0.0112677 | 459915 | 48012469 | 0.0269 | 0.0097 | 0.005735 |
| rs205262 | Usual walking pace | Type 2 diabetes | -0.00870949 | 459915 | 34563164 | -0.0037 | 0.0072 | 0.6085 |
| rs2170670 | Usual walking pace | Type 2 diabetes | -0.00708769 | 459915 | 16944621 | 0.0129 | 0.0065 | 0.0469 |
| rs2280406 | Usual walking pace | Type 2 diabetes | -0.00996145 | 459915 | 49941436 | 0.0305 | 0.0064 | 0.000001793 |
| rs2297600 | Usual walking pace | Type 2 diabetes | -0.0115607 | 459915 | 32207581 | 0.0234 | 0.0085 | 0.005795 |
| rs2439823 | Usual walking pace | Type 2 diabetes | -0.0072789 | 459915 | 99778226 | 0.0103 | 0.0064 | 0.1068 |
| rs2602731 | Usual walking pace | Type 2 diabetes | -0.00766675 | 459915 | 4944771 | 0.0281 | 0.007 | 0.00006184 |
| rs2644135 | Usual walking pace | Type 2 diabetes | 0.00751192 | 459915 | 201856256 | -0.0285 | 0.0068 | 0.00002818 |
| rs2645979 | Usual walking pace | Type 2 diabetes | 0.00861798 | 459915 | 84017043 | -0.0113 | 0.0066 | 0.0867 |
| rs273512 | Usual walking pace | Type 2 diabetes | -0.00966507 | 459915 | 18224729 | -0.0034 | 0.0065 | 0.6004 |
| rs28519617 | Usual walking pace | Type 2 diabetes | -0.00812933 | 459915 | 135874930 | 0.0297 | 0.0072 | 0.00003939 |
| rs35711462 | Usual walking pace | Type 2 diabetes | -0.0071659 | 459915 | 50847577 | 0.0068 | 0.0064 | 0.287 |
| rs4109292 | Usual walking pace | Type 2 diabetes | 0.00735336 | 459915 | 126710654 | -0.0043 | 0.0064 | 0.5008 |
| rs4516268 | Usual walking pace | Type 2 diabetes | 0.00983008 | 459915 | 1846831 | -0.0329 | 0.0082 | 0.00005613 |
| rs45583845 | Usual walking pace | Type 2 diabetes | -0.0198998 | 459915 | 57858194 | -0.0086 | 0.0183 | 0.6388 |
| rs4643373 | Usual walking pace | Type 2 diabetes | 0.0077266 | 459915 | 47123423 | -0.0475 | 0.007 | 1.278E-11 |
| rs4715208 | Usual walking pace | Type 2 diabetes | -0.0083844 | 459915 | 50829471 | 0.031 | 0.0079 | 0.00007891 |
| rs4839898 | Usual walking pace | Type 2 diabetes | 0.0130564 | 459915 | 97546759 | -0.0295 | 0.0105 | 0.00484 |
| rs55680124 | Usual walking pace | Type 2 diabetes | -0.010742 | 459915 | 105984624 | 0.0352 | 0.009 | 0.0000926 |
| rs57800857 | Usual walking pace | Type 2 diabetes | 0.00867607 | 459915 | 140863365 | -0.0283 | 0.0069 | 0.00004216 |
| rs613872 | Usual walking pace | Type 2 diabetes | -0.0147074 | 459915 | 53210302 | 0.0053 | 0.0086 | 0.537 |
| rs62048402 | Usual walking pace | Type 2 diabetes | -0.00987537 | 459915 | 53803223 | 0.1218 | 0.0065 | 1.52E-78 |
| rs6763292 | Usual walking pace | Type 2 diabetes | 0.00958833 | 459915 | 129044705 | -0.0219 | 0.0073 | 0.002808 |
| rs7789719 | Usual walking pace | Type 2 diabetes | 0.00854199 | 459915 | 66893916 | -0.0171 | 0.0076 | 0.02527 |
| rs7795394 | Usual walking pace | Type 2 diabetes | 0.00930493 | 459915 | 113560607 | -0.0141 | 0.0066 | 0.03255 |
| rs7896518 | Usual walking pace | Type 2 diabetes | 0.00999095 | 459915 | 65104500 | -0.0145 | 0.0065 | 0.0255 |
| rs8010773 | Usual walking pace | Type 2 diabetes | -0.00816754 | 459915 | 46956863 | 0.0213 | 0.007 | 0.002395 |
| rs8011870 | Usual walking pace | Type 2 diabetes | -0.00782739 | 459915 | 80173397 | 0.0241 | 0.0071 | 0.0007119 |
| rs8028757 | Usual walking pace | Type 2 diabetes | 0.0107284 | 459915 | 75822794 | -0.0404 | 0.0108 | 0.0001796 |
| rs819167 | Usual walking pace | Type 2 diabetes | -0.0157993 | 459915 | 32903687 | 0.0013 | 0.0125 | 0.9169 |
| rs830627 | Usual walking pace | Type 2 diabetes | 0.00742054 | 459915 | 71675270 | -0.0238 | 0.0065 | 0.0002461 |
| rs891387 | Usual walking pace | Type 2 diabetes | 0.00786028 | 459915 | 21103909 | -0.0264 | 0.0064 | 0.00003573 |
| rs9366651 | Usual walking pace | Type 2 diabetes | 0.00948542 | 459915 | 26336696 | -0.0232 | 0.0065 | 0.0003517 |
| rs9783304 | Usual walking pace | Type 2 diabetes | 0.00763598 | 459915 | 43660255 | -0.0337 | 0.0069 | 0.000001079 |

**Supplementary Table4.** Genome-wide significant SNPs for T2DM

| **SNP** | **exposure** | **outcome** | **beta.exposure** | **se.exposure** | **pval.exposure** | **beta.outcome** | **se.outcome** | **pval.outcome** |
| --- | --- | --- | --- | --- | --- | --- | --- | --- |
| rs1007090 | Type 2 diabetes | Low hand grip strength | -0.044 | 0.0068 | 1.012E-10 | 0.0209 | 0.0078 | 0.00689398 |
| rs10097617 | Type 2 diabetes | Low hand grip strength | 0.0487 | 0.0064 | 2.439E-14 | -0.0054 | 0.0074 | 0.466 |
| rs10406431 | Type 2 diabetes | Low hand grip strength | 0.0603 | 0.0065 | 1.557E-20 | 0.0046 | 0.0074 | 0.5353 |
| rs10419627 | Type 2 diabetes | Low hand grip strength | 0.0431 | 0.0065 | 3.15E-11 | 0.0042 | 0.0075 | 0.572 |
| rs10516495 | Type 2 diabetes | Low hand grip strength | -0.0418 | 0.0069 | 1.458E-09 | -0.0012 | 0.008 | 0.8756 |
| rs1061810 | Type 2 diabetes | Low hand grip strength | 0.0502 | 0.007 | 8.305E-13 | 0.0054 | 0.0081 | 0.5023 |
| rs10750397 | Type 2 diabetes | Low hand grip strength | 0.0394 | 0.0071 | 3.132E-08 | 0.01 | 0.0082 | 0.2262 |
| rs10811660 | Type 2 diabetes | Low hand grip strength | -0.1598 | 0.0086 | 2.541E-77 | -0.0094 | 0.0097 | 0.3332 |
| rs10830963 | Type 2 diabetes | Low hand grip strength | -0.101 | 0.0071 | 1.121E-45 | 0.0075 | 0.0082 | 0.3648 |
| rs10882099 | Type 2 diabetes | Low hand grip strength | 0.1095 | 0.0065 | 7.726E-64 | 0.0034 | 0.0075 | 0.6478 |
| rs10908278 | Type 2 diabetes | Low hand grip strength | -0.0749 | 0.0067 | 5.254E-29 | 0.0031 | 0.0074 | 0.6748 |
| rs10937721 | Type 2 diabetes | Low hand grip strength | 0.085 | 0.0066 | 5.39E-38 | 0.0076 | 0.0076 | 0.3135 |
| rs10974438 | Type 2 diabetes | Low hand grip strength | -0.0514 | 0.0067 | 1.713E-14 | 0.0183 | 0.0077 | 0.0178299 |
| rs11048456 | Type 2 diabetes | Low hand grip strength | -0.0454 | 0.0072 | 3.295E-10 | 0.0114 | 0.0086 | 0.1829 |
| rs11063029 | Type 2 diabetes | Low hand grip strength | 0.0858 | 0.0138 | 5.364E-10 | 0.0171 | 0.0166 | 0.3032 |
| rs11063069 | Type 2 diabetes | Low hand grip strength | -0.056 | 0.0079 | 9.941E-13 | -0.0155 | 0.0091 | 0.0877405 |
| rs1117610 | Type 2 diabetes | Low hand grip strength | -0.0423 | 0.0076 | 3.125E-08 | 0.0332 | 0.0088 | 0.000161102 |
| rs11257655 | Type 2 diabetes | Low hand grip strength | 0.0859 | 0.0077 | 1.455E-28 | 0.0002 | 0.009 | 0.9837 |
| rs1127215 | Type 2 diabetes | Low hand grip strength | -0.0491 | 0.0065 | 3.918E-14 | -0.01 | 0.0074 | 0.18 |
| rs11496066 | Type 2 diabetes | Low hand grip strength | 0.0508 | 0.0083 | 8.171E-10 | 0.0057 | 0.0094 | 0.5469 |
| rs115505614 | Type 2 diabetes | Low hand grip strength | 0.1657 | 0.0149 | 7.58E-29 | 0.0611 | 0.0169 | 0.000290998 |
| rs11680058 | Type 2 diabetes | Low hand grip strength | 0.0581 | 0.0104 | 2.081E-08 | -0.0018 | 0.0119 | 0.88 |
| rs11688682 | Type 2 diabetes | Low hand grip strength | -0.0581 | 0.0076 | 2.928E-14 | -0.0097 | 0.0086 | 0.2622 |
| rs11699802 | Type 2 diabetes | Low hand grip strength | -0.0443 | 0.0065 | 8.838E-12 | -0.0091 | 0.0074 | 0.2169 |
| rs11708067 | Type 2 diabetes | Low hand grip strength | 0.0882 | 0.0077 | 5.046E-30 | -0.0158 | 0.0086 | 0.0646696 |
| rs11709077 | Type 2 diabetes | Low hand grip strength | -0.104 | 0.0097 | 1.255E-26 | 0.0023 | 0.0113 | 0.8375 |
| rs11759026 | Type 2 diabetes | Low hand grip strength | -0.066 | 0.0075 | 2.042E-18 | 0.0157 | 0.0088 | 0.0736105 |
| rs11842871 | Type 2 diabetes | Low hand grip strength | -0.04 | 0.0073 | 4.825E-08 | -0.0038 | 0.0084 | 0.649101 |
| rs11856307 | Type 2 diabetes | Low hand grip strength | 0.047 | 0.0065 | 4.481E-13 | 0.0002 | 0.0074 | 0.9831 |
| rs11958808 | Type 2 diabetes | Low hand grip strength | 0.0403 | 0.0066 | 9.992E-10 | -0.0105 | 0.0075 | 0.1645 |
| rs12001437 | Type 2 diabetes | Low hand grip strength | -0.0402 | 0.0066 | 1.099E-09 | -0.0251 | 0.0076 | 0.000946695 |
| rs12140153 | Type 2 diabetes | Low hand grip strength | -0.0645 | 0.0113 | 1.17E-08 | 0.0004 | 0.0129 | 0.9775 |
| rs12325539 | Type 2 diabetes | Low hand grip strength | -0.041 | 0.0065 | 2.686E-10 | 0.0098 | 0.0075 | 0.193 |
| rs1260326 | Type 2 diabetes | Low hand grip strength | -0.0644 | 0.0066 | 1.621E-22 | 0.0062 | 0.0075 | 0.4111 |
| rs12680692 | Type 2 diabetes | Low hand grip strength | 0.0413 | 0.0071 | 6.602E-09 | -0.0098 | 0.0081 | 0.2282 |
| rs12719778 | Type 2 diabetes | Low hand grip strength | 0.0381 | 0.0065 | 4.38E-09 | -0.0041 | 0.0074 | 0.5824 |
| rs12910361 | Type 2 diabetes | Low hand grip strength | -0.0814 | 0.007 | 3.951E-31 | -0.0191 | 0.0082 | 0.0189099 |
| rs12920022 | Type 2 diabetes | Low hand grip strength | 0.0528 | 0.0092 | 1.001E-08 | -0.012 | 0.0102 | 0.2412 |
| rs13022337 | Type 2 diabetes | Low hand grip strength | -0.0519 | 0.0088 | 3.611E-09 | -0.0059 | 0.0098 | 0.5448 |
| rs13130484 | Type 2 diabetes | Low hand grip strength | 0.0435 | 0.0067 | 8.492E-11 | 0.0078 | 0.0075 | 0.293 |
| rs13330951 | Type 2 diabetes | Low hand grip strength | 0.0361 | 0.0064 | 1.584E-08 | 0.0157 | 0.0074 | 0.0339703 |
| rs13385171 | Type 2 diabetes | Low hand grip strength | 0.0357 | 0.0065 | 3.81E-08 | -0.0124 | 0.0075 | 0.0966207 |
| rs13389219 | Type 2 diabetes | Low hand grip strength | -0.0605 | 0.0065 | 1.165E-20 | 0.0022 | 0.0075 | 0.7659 |
| rs1359790 | Type 2 diabetes | Low hand grip strength | -0.0817 | 0.0071 | 1.76E-30 | 0.003 | 0.0081 | 0.713099 |
| rs1381937 | Type 2 diabetes | Low hand grip strength | 0.0442 | 0.0064 | 4.501E-12 | -0.0046 | 0.0074 | 0.5349 |
| rs139688524 | Type 2 diabetes | Low hand grip strength | 0.1739 | 0.0216 | 7.468E-16 | 0.003 | 0.0252 | 0.904 |
| rs1412234 | Type 2 diabetes | Low hand grip strength | -0.0393 | 0.0068 | 7.713E-09 | 0.002 | 0.0079 | 0.8009 |
| rs141521721 | Type 2 diabetes | Low hand grip strength | 0.1212 | 0.0214 | 1.392E-08 | 0.0452 | 0.0238 | 0.0576196 |
| rs1426371 | Type 2 diabetes | Low hand grip strength | -0.0517 | 0.0073 | 1.738E-12 | -0.0188 | 0.0085 | 0.0272001 |
| rs1431841 | Type 2 diabetes | Low hand grip strength | 0.0429 | 0.0077 | 3.078E-08 | -0.0122 | 0.0091 | 0.1812 |
| rs145678014 | Type 2 diabetes | Low hand grip strength | -0.1048 | 0.0163 | 1.397E-10 | 0.0049 | 0.018 | 0.7878 |
| rs1468906 | Type 2 diabetes | Low hand grip strength | 0.0388 | 0.0067 | 7.029E-09 | -0.0011 | 0.0077 | 0.8834 |
| rs1493694 | Type 2 diabetes | Low hand grip strength | 0.08 | 0.0102 | 3.353E-15 | 0.0143 | 0.0118 | 0.2251 |
| rs1496653 | Type 2 diabetes | Low hand grip strength | 0.0665 | 0.0079 | 2.487E-17 | 0.0053 | 0.0091 | 0.5575 |
| rs1517037 | Type 2 diabetes | Low hand grip strength | -0.0451 | 0.0082 | 3.345E-08 | -0.0022 | 0.0094 | 0.8174 |
| rs1561927 | Type 2 diabetes | Low hand grip strength | -0.042 | 0.0072 | 6.115E-09 | 0.0068 | 0.0084 | 0.4184 |
| rs1562396 | Type 2 diabetes | Low hand grip strength | -0.0555 | 0.0069 | 9.635E-16 | -0.0078 | 0.0079 | 0.3212 |
| rs1573090 | Type 2 diabetes | Low hand grip strength | 0.0458 | 0.0065 | 1.722E-12 | 0.018 | 0.0075 | 0.0165901 |
| rs1665901 | Type 2 diabetes | Low hand grip strength | 0.0398 | 0.0068 | 4.973E-09 | 0.0048 | 0.0078 | 0.5443 |
| rs17030845 | Type 2 diabetes | Low hand grip strength | -0.1191 | 0.0108 | 2.349E-28 | -0.0399 | 0.0119 | 0.000823891 |
| rs1705263 | Type 2 diabetes | Low hand grip strength | -0.0484 | 0.0064 | 3.507E-14 | -0.0043 | 0.0074 | 0.5637 |
| rs1708302 | Type 2 diabetes | Low hand grip strength | -0.0909 | 0.0064 | 5.769E-46 | -0.0057 | 0.0073 | 0.4368 |
| rs17122772 | Type 2 diabetes | Low hand grip strength | -0.0428 | 0.0077 | 3.313E-08 | 0.0084 | 0.0088 | 0.3387 |
| rs17168486 | Type 2 diabetes | Low hand grip strength | 0.0672 | 0.0083 | 4.5E-16 | -0.0215 | 0.0097 | 0.0269401 |
| rs17522122 | Type 2 diabetes | Low hand grip strength | 0.0356 | 0.0064 | 2.49E-08 | -0.0173 | 0.0074 | 0.0193901 |
| rs17744783 | Type 2 diabetes | Low hand grip strength | 0.0577 | 0.0103 | 1.873E-08 | -0.0087 | 0.012 | 0.468 |
| rs17772814 | Type 2 diabetes | Low hand grip strength | -0.0746 | 0.0125 | 2.133E-09 | -0.0038 | 0.0143 | 0.7935 |
| rs17791513 | Type 2 diabetes | Low hand grip strength | 0.1016 | 0.0132 | 1.346E-14 | 0.0064 | 0.0152 | 0.6725 |
| rs1783541 | Type 2 diabetes | Low hand grip strength | 0.0608 | 0.0081 | 4.647E-14 | 0.0018 | 0.009 | 0.8438 |
| rs1800961 | Type 2 diabetes | Low hand grip strength | 0.1602 | 0.0175 | 5.095E-20 | -0.0448 | 0.0214 | 0.0360396 |
| rs1999536 | Type 2 diabetes | Low hand grip strength | -0.0404 | 0.0065 | 4.863E-10 | -0.011 | 0.0075 | 0.139 |
| rs2023681 | Type 2 diabetes | Low hand grip strength | -0.0826 | 0.0115 | 7.399E-13 | 0.0127 | 0.0128 | 0.3208 |
| rs2062213 | Type 2 diabetes | Low hand grip strength | -0.0743 | 0.012 | 6.799E-10 | -0.0065 | 0.0138 | 0.6369 |
| rs2080385 | Type 2 diabetes | Low hand grip strength | -0.0551 | 0.0074 | 1.244E-13 | 0.0072 | 0.0086 | 0.4028 |
| rs2107133 | Type 2 diabetes | Low hand grip strength | 0.0643 | 0.0097 | 4.017E-11 | 0.007 | 0.0108 | 0.5166 |
| rs2215383 | Type 2 diabetes | Low hand grip strength | -0.0641 | 0.0064 | 1.056E-23 | 0.0016 | 0.0074 | 0.8273 |
| rs2237895 | Type 2 diabetes | Low hand grip strength | -0.0892 | 0.0066 | 1.147E-41 | 0.0017 | 0.0075 | 0.816 |
| rs2258238 | Type 2 diabetes | Low hand grip strength | -0.102 | 0.0106 | 5.139E-22 | 0.0012 | 0.0119 | 0.9177 |
| rs2277536 | Type 2 diabetes | Low hand grip strength | -0.0429 | 0.007 | 9.631E-10 | 0.0019 | 0.0081 | 0.8189 |
| rs2290202 | Type 2 diabetes | Low hand grip strength | 0.0666 | 0.0092 | 4.892E-13 | 0.0043 | 0.0109 | 0.693999 |
| rs2292662 | Type 2 diabetes | Low hand grip strength | -0.0645 | 0.0088 | 2.239E-13 | 0.0207 | 0.0102 | 0.0424405 |
| rs231360 | Type 2 diabetes | Low hand grip strength | 0.0576 | 0.0067 | 8.269E-18 | 0.0001 | 0.0076 | 0.9842 |
| rs2351707 | Type 2 diabetes | Low hand grip strength | -0.0644 | 0.007 | 4.301E-20 | -0.0061 | 0.0082 | 0.4598 |
| rs2383205 | Type 2 diabetes | Low hand grip strength | -0.0533 | 0.0065 | 2.199E-16 | -0.0103 | 0.0076 | 0.1713 |
| rs243019 | Type 2 diabetes | Low hand grip strength | -0.0588 | 0.0064 | 3.372E-20 | 0.0174 | 0.0074 | 0.0189099 |
| rs2767036 | Type 2 diabetes | Low hand grip strength | -0.0389 | 0.007 | 2.936E-08 | -0.0035 | 0.0081 | 0.6627 |
| rs2796441 | Type 2 diabetes | Low hand grip strength | -0.0674 | 0.0065 | 2.972E-25 | 0.0019 | 0.0074 | 0.8008 |
| rs2812545 | Type 2 diabetes | Low hand grip strength | 0.0413 | 0.0065 | 1.99E-10 | -0.0184 | 0.0074 | 0.0134499 |
| rs2820441 | Type 2 diabetes | Low hand grip strength | 0.0556 | 0.0069 | 8.562E-16 | -0.0095 | 0.0079 | 0.2279 |
| rs28533815 | Type 2 diabetes | Low hand grip strength | -0.0748 | 0.008 | 5.452E-21 | -0.0086 | 0.0087 | 0.3221 |
| rs28663084 | Type 2 diabetes | Low hand grip strength | -0.0376 | 0.0068 | 3.298E-08 | -0.0017 | 0.0079 | 0.829 |
| rs2896177 | Type 2 diabetes | Low hand grip strength | -0.0367 | 0.0064 | 9.128E-09 | -0.0069 | 0.0074 | 0.3481 |
| rs2925979 | Type 2 diabetes | Low hand grip strength | 0.0546 | 0.007 | 7.068E-15 | -0.0048 | 0.0081 | 0.5525 |
| rs2972144 | Type 2 diabetes | Low hand grip strength | -0.0911 | 0.0066 | 2.192E-43 | 0.0034 | 0.0077 | 0.663201 |
| rs3019208 | Type 2 diabetes | Low hand grip strength | 0.0397 | 0.0068 | 5.431E-09 | 0.0157 | 0.0079 | 0.0477595 |
| rs3094682 | Type 2 diabetes | Low hand grip strength | -0.0619 | 0.0082 | 3.469E-14 | -0.0216 | 0.0096 | 0.0243501 |
| rs320369 | Type 2 diabetes | Low hand grip strength | 0.0372 | 0.0068 | 4.602E-08 | 0.0088 | 0.008 | 0.2696 |
| rs329122 | Type 2 diabetes | Low hand grip strength | 0.0366 | 0.0065 | 1.719E-08 | -0.0037 | 0.0074 | 0.618399 |
| rs340874 | Type 2 diabetes | Low hand grip strength | -0.0678 | 0.0065 | 1.555E-25 | 0.0016 | 0.0074 | 0.8337 |
| rs34341 | Type 2 diabetes | Low hand grip strength | -0.044 | 0.0065 | 1.218E-11 | -0.0062 | 0.0075 | 0.403 |
| rs34715063 | Type 2 diabetes | Low hand grip strength | -0.0772 | 0.0099 | 8.403E-15 | -0.0228 | 0.0112 | 0.0411898 |
| rs348330 | Type 2 diabetes | Low hand grip strength | -0.0492 | 0.0067 | 2.1E-13 | 0.0037 | 0.0077 | 0.6264 |
| rs34990153 | Type 2 diabetes | Low hand grip strength | 0.0476 | 0.0065 | 2.257E-13 | 0.0044 | 0.0074 | 0.5568 |
| rs35011184 | Type 2 diabetes | Low hand grip strength | 0.2822 | 0.0075 | 1E-200 | 0.0119 | 0.0088 | 0.1742 |
| rs35318451 | Type 2 diabetes | Low hand grip strength | 0.0478 | 0.007 | 9.498E-12 | -0.0145 | 0.0079 | 0.0664202 |
| rs35777422 | Type 2 diabetes | Low hand grip strength | -0.0381 | 0.0067 | 1.302E-08 | -0.0157 | 0.0077 | 0.0419102 |
| rs35895680 | Type 2 diabetes | Low hand grip strength | -0.0554 | 0.0069 | 1.084E-15 | -0.0034 | 0.0079 | 0.6633 |
| rs36138276 | Type 2 diabetes | Low hand grip strength | -0.0431 | 0.0066 | 6.403E-11 | 0.0013 | 0.0074 | 0.8612 |
| rs3768321 | Type 2 diabetes | Low hand grip strength | 0.084 | 0.008 | 4.753E-26 | -0.0038 | 0.0092 | 0.6821 |
| rs3783394 | Type 2 diabetes | Low hand grip strength | -0.038 | 0.0067 | 1.421E-08 | -0.0076 | 0.0077 | 0.3236 |
| rs3786900 | Type 2 diabetes | Low hand grip strength | 0.0434 | 0.0072 | 1.886E-09 | -0.0079 | 0.0083 | 0.3446 |
| rs3798519 | Type 2 diabetes | Low hand grip strength | -0.0616 | 0.0082 | 4.602E-14 | -0.0192 | 0.0095 | 0.0436305 |
| rs3802177 | Type 2 diabetes | Low hand grip strength | -0.1077 | 0.0069 | 9.163E-55 | -0.0069 | 0.008 | 0.3838 |
| rs429358 | Type 2 diabetes | Low hand grip strength | 0.0746 | 0.0091 | 2.621E-16 | -0.0184 | 0.0103 | 0.0746999 |
| rs4368494 | Type 2 diabetes | Low hand grip strength | -0.054 | 0.007 | 1.385E-14 | 0.0036 | 0.008 | 0.6565 |
| rs459193 | Type 2 diabetes | Low hand grip strength | -0.0722 | 0.0073 | 6.783E-23 | -0.0084 | 0.0085 | 0.3209 |
| rs4686471 | Type 2 diabetes | Low hand grip strength | -0.0608 | 0.0066 | 3.045E-20 | -0.0141 | 0.0076 | 0.0641904 |
| rs4688760 | Type 2 diabetes | Low hand grip strength | 0.042 | 0.007 | 2.136E-09 | 0.003 | 0.008 | 0.706801 |
| rs4709746 | Type 2 diabetes | Low hand grip strength | -0.0567 | 0.0096 | 3.951E-09 | -0.0131 | 0.0108 | 0.2256 |
| rs474513 | Type 2 diabetes | Low hand grip strength | 0.0399 | 0.0064 | 4.178E-10 | 0.0086 | 0.0074 | 0.245 |
| rs4804833 | Type 2 diabetes | Low hand grip strength | 0.048 | 0.0066 | 3.417E-13 | -0.0108 | 0.0076 | 0.157 |
| rs4812034 | Type 2 diabetes | Low hand grip strength | 0.0416 | 0.0064 | 7.347E-11 | -0.0008 | 0.0074 | 0.9102 |
| rs490689 | Type 2 diabetes | Low hand grip strength | 0.0543 | 0.008 | 8.863E-12 | 0.0085 | 0.0091 | 0.3461 |
| rs4925109 | Type 2 diabetes | Low hand grip strength | 0.0476 | 0.0069 | 5.65E-12 | 0.0013 | 0.008 | 0.874 |
| rs4929965 | Type 2 diabetes | Low hand grip strength | 0.0668 | 0.0068 | 9.669E-23 | -0.0071 | 0.0077 | 0.3522 |
| rs4977213 | Type 2 diabetes | Low hand grip strength | -0.0507 | 0.0069 | 2.187E-13 | 0.0039 | 0.0076 | 0.612099 |
| rs505922 | Type 2 diabetes | Low hand grip strength | -0.0473 | 0.0068 | 3.65E-12 | 0.002 | 0.0079 | 0.8017 |
| rs508419 | Type 2 diabetes | Low hand grip strength | -0.0811 | 0.0075 | 5.433E-27 | -0.0056 | 0.0086 | 0.5139 |
| rs5215 | Type 2 diabetes | Low hand grip strength | -0.0706 | 0.0066 | 9.848E-27 | -0.0013 | 0.0077 | 0.8678 |
| rs55653563 | Type 2 diabetes | Low hand grip strength | 0.0435 | 0.0072 | 1.731E-09 | 0.0045 | 0.0084 | 0.5931 |
| rs55872725 | Type 2 diabetes | Low hand grip strength | 0.122 | 0.0065 | 8.509E-79 | -0.0093 | 0.0075 | 0.2144 |
| rs56187241 | Type 2 diabetes | Low hand grip strength | 0.1014 | 0.0157 | 1.072E-10 | 0.0045 | 0.019 | 0.8119 |
| rs56348580 | Type 2 diabetes | Low hand grip strength | -0.0617 | 0.0069 | 4.313E-19 | 0.0032 | 0.008 | 0.683901 |
| rs5758223 | Type 2 diabetes | Low hand grip strength | 0.0401 | 0.0071 | 1.779E-08 | -0.0027 | 0.0082 | 0.743599 |
| rs576674 | Type 2 diabetes | Low hand grip strength | -0.0538 | 0.0086 | 3.697E-10 | -0.0081 | 0.0098 | 0.4101 |
| rs58432198 | Type 2 diabetes | Low hand grip strength | -0.0636 | 0.0103 | 5.707E-10 | -0.0163 | 0.0116 | 0.1577 |
| rs58642235 | Type 2 diabetes | Low hand grip strength | -0.0566 | 0.0094 | 1.896E-09 | -0.0129 | 0.0107 | 0.2297 |
| rs59147390 | Type 2 diabetes | Low hand grip strength | 0.0648 | 0.0105 | 6.056E-10 | -0.0018 | 0.0117 | 0.8765 |
| rs601945 | Type 2 diabetes | Low hand grip strength | -0.0849 | 0.0086 | 4.662E-23 | -0.0751 | 0.0095 | 2.19584E-15 |
| rs61676547 | Type 2 diabetes | Low hand grip strength | 0.0525 | 0.0081 | 7.417E-11 | 0.0084 | 0.0094 | 0.3743 |
| rs62107261 | Type 2 diabetes | Low hand grip strength | 0.1019 | 0.0161 | 2.621E-10 | 0.0235 | 0.0176 | 0.1816 |
| rs62271373 | Type 2 diabetes | Low hand grip strength | 0.0882 | 0.0144 | 1.033E-09 | -0.0089 | 0.016 | 0.5755 |
| rs62563593 | Type 2 diabetes | Low hand grip strength | -0.0394 | 0.0065 | 1.284E-09 | -0.0017 | 0.0075 | 0.8251 |
| rs6444809 | Type 2 diabetes | Low hand grip strength | 0.0544 | 0.0096 | 1.628E-08 | -0.0039 | 0.0112 | 0.7257 |
| rs6459737 | Type 2 diabetes | Low hand grip strength | -0.0583 | 0.0067 | 3.311E-18 | -0.0055 | 0.0077 | 0.4791 |
| rs648795 | Type 2 diabetes | Low hand grip strength | 0.0391 | 0.0065 | 1.71E-09 | 0.0165 | 0.0075 | 0.0273602 |
| rs6545714 | Type 2 diabetes | Low hand grip strength | -0.0363 | 0.0065 | 2.246E-08 | 0.0052 | 0.0075 | 0.491501 |
| rs6600191 | Type 2 diabetes | Low hand grip strength | 0.0587 | 0.0085 | 4.471E-12 | 0.0075 | 0.0095 | 0.4301 |
| rs6687271 | Type 2 diabetes | Low hand grip strength | -0.0402 | 0.0073 | 4.136E-08 | -0.0013 | 0.0086 | 0.8812 |
| rs672271 | Type 2 diabetes | Low hand grip strength | -0.06 | 0.011 | 4.825E-08 | -0.0258 | 0.013 | 0.0475499 |
| rs67232546 | Type 2 diabetes | Low hand grip strength | 0.0531 | 0.008 | 2.505E-11 | -0.0122 | 0.0091 | 0.1782 |
| rs6821438 | Type 2 diabetes | Low hand grip strength | 0.0397 | 0.0064 | 5.103E-10 | 0.0214 | 0.0074 | 0.00364099 |
| rs6885132 | Type 2 diabetes | Low hand grip strength | 0.077 | 0.011 | 2.488E-12 | 0.022 | 0.0125 | 0.0785905 |
| rs6937438 | Type 2 diabetes | Low hand grip strength | -0.0514 | 0.007 | 2.352E-13 | -0.0087 | 0.0081 | 0.2844 |
| rs702634 | Type 2 diabetes | Low hand grip strength | 0.0503 | 0.0069 | 3.365E-13 | -0.0039 | 0.008 | 0.6273 |
| rs703972 | Type 2 diabetes | Low hand grip strength | -0.0698 | 0.0065 | 5.771E-27 | -0.001 | 0.0074 | 0.8924 |
| rs7178762 | Type 2 diabetes | Low hand grip strength | -0.0387 | 0.0064 | 1.367E-09 | -0.013 | 0.0074 | 0.08007 |
| rs7240767 | Type 2 diabetes | Low hand grip strength | -0.0372 | 0.0066 | 1.705E-08 | 0.0065 | 0.0076 | 0.3877 |
| rs72802358 | Type 2 diabetes | Low hand grip strength | -0.1135 | 0.0107 | 2.215E-26 | -0.0163 | 0.0122 | 0.1801 |
| rs7313918 | Type 2 diabetes | Low hand grip strength | -0.0559 | 0.0094 | 2.99E-09 | 0.0103 | 0.0111 | 0.356 |
| rs7325671 | Type 2 diabetes | Low hand grip strength | 0.0545 | 0.0096 | 1.533E-08 | -0.0144 | 0.011 | 0.1911 |
| rs739846 | Type 2 diabetes | Low hand grip strength | 0.0877 | 0.0119 | 2.02E-13 | 0.0354 | 0.0137 | 0.00972904 |
| rs745805 | Type 2 diabetes | Low hand grip strength | 0.0632 | 0.0084 | 4.516E-14 | -0.0002 | 0.0096 | 0.9864 |
| rs7568172 | Type 2 diabetes | Low hand grip strength | -0.0827 | 0.0135 | 9.187E-10 | -0.0133 | 0.0152 | 0.3832 |
| rs7572970 | Type 2 diabetes | Low hand grip strength | -0.0447 | 0.0072 | 6.12E-10 | -0.0043 | 0.0083 | 0.5987 |
| rs7633675 | Type 2 diabetes | Low hand grip strength | -0.1085 | 0.0068 | 3.206E-57 | -0.0054 | 0.0079 | 0.4963 |
| rs7667864 | Type 2 diabetes | Low hand grip strength | -0.0402 | 0.0071 | 1.64E-08 | -0.0278 | 0.0082 | 0.000674606 |
| rs7669833 | Type 2 diabetes | Low hand grip strength | -0.0572 | 0.007 | 3.524E-16 | 0.0134 | 0.0081 | 0.09834 |
| rs76895963 | Type 2 diabetes | Low hand grip strength | 0.4826 | 0.0275 | 9.124E-69 | 0.0885 | 0.0301 | 0.00327401 |
| rs7732130 | Type 2 diabetes | Low hand grip strength | -0.0606 | 0.007 | 5.693E-18 | -0.024 | 0.0079 | 0.00251802 |
| rs77464186 | Type 2 diabetes | Low hand grip strength | 0.1002 | 0.0087 | 9.304E-31 | 0.0178 | 0.0102 | 0.0796104 |
| rs77864822 | Type 2 diabetes | Low hand grip strength | 0.0753 | 0.0129 | 5.005E-09 | 0.0163 | 0.015 | 0.2773 |
| rs7966976 | Type 2 diabetes | Low hand grip strength | -0.0751 | 0.0081 | 1.217E-20 | -0.0032 | 0.0092 | 0.727301 |
| rs79920718 | Type 2 diabetes | Low hand grip strength | 0.0389 | 0.0067 | 6.431E-09 | 0.0099 | 0.0086 | 0.2461 |
| rs8008910 | Type 2 diabetes | Low hand grip strength | 0.0554 | 0.0076 | 4.223E-13 | -0.0037 | 0.0089 | 0.6788 |
| rs8071043 | Type 2 diabetes | Low hand grip strength | -0.0523 | 0.0068 | 1.533E-14 | -0.0129 | 0.0078 | 0.0989897 |
| rs8097210 | Type 2 diabetes | Low hand grip strength | -0.0537 | 0.0072 | 1.061E-13 | 0.0141 | 0.0084 | 0.0908092 |
| rs8192675 | Type 2 diabetes | Low hand grip strength | 0.0659 | 0.007 | 5.772E-21 | 0.0043 | 0.0081 | 0.5935 |
| rs878521 | Type 2 diabetes | Low hand grip strength | 0.0618 | 0.0074 | 9.308E-17 | 0.0131 | 0.0084 | 0.1211 |
| rs9304665 | Type 2 diabetes | Low hand grip strength | 0.0418 | 0.0076 | 4.529E-08 | 0.0108 | 0.0087 | 0.2162 |
| rs9368222 | Type 2 diabetes | Low hand grip strength | 0.1379 | 0.0071 | 1.42E-83 | -0.0019 | 0.0083 | 0.8197 |
| rs9379084 | Type 2 diabetes | Low hand grip strength | -0.0994 | 0.0106 | 5.478E-21 | 0.0359 | 0.0117 | 0.002208 |
| rs9563615 | Type 2 diabetes | Low hand grip strength | 0.0408 | 0.0072 | 1.628E-08 | 0.002 | 0.0081 | 0.8076 |
| rs9665898 | Type 2 diabetes | Low hand grip strength | -0.0525 | 0.0094 | 2.528E-08 | -0.002 | 0.0108 | 0.8508 |
| rs9873519 | Type 2 diabetes | Low hand grip strength | 0.0371 | 0.0064 | 6.292E-09 | 0.0001 | 0.0074 | 0.9923 |
| rs1007090 | Type 2 diabetes | ALM | -0.044 | 0.0068 | 1.012E-10 | -0.0166 | 0.002 | 8.77001E-17 |
| rs10097617 | Type 2 diabetes | ALM | 0.0487 | 0.0064 | 2.439E-14 | -0.0071 | 0.0019 | 0.000175598 |
| rs10406431 | Type 2 diabetes | ALM | 0.0603 | 0.0065 | 1.557E-20 | 0.0036 | 0.0019 | 0.0603601 |
| rs10419627 | Type 2 diabetes | ALM | 0.0431 | 0.0065 | 3.15E-11 | -0.007 | 0.0019 | 0.000256 |
| rs10516495 | Type 2 diabetes | ALM | -0.0418 | 0.0069 | 1.458E-09 | -0.0014 | 0.002 | 0.4933 |
| rs1061810 | Type 2 diabetes | ALM | 0.0502 | 0.007 | 8.305E-13 | 0.006 | 0.0021 | 0.00405098 |
| rs10750397 | Type 2 diabetes | ALM | 0.0394 | 0.0071 | 3.132E-08 | 0.0046 | 0.0021 | 0.0310799 |
| rs10811660 | Type 2 diabetes | ALM | -0.1598 | 0.0086 | 2.541E-77 | 0.0009 | 0.0025 | 0.733 |
| rs10830963 | Type 2 diabetes | ALM | -0.101 | 0.0071 | 1.121E-45 | -0.0049 | 0.0021 | 0.0194899 |
| rs10882099 | Type 2 diabetes | ALM | 0.1095 | 0.0065 | 7.726E-64 | 0.0001 | 0.0019 | 0.967 |
| rs10908278 | Type 2 diabetes | ALM | -0.0749 | 0.0067 | 5.254E-29 | -0.0019 | 0.0019 | 0.3144 |
| rs10937721 | Type 2 diabetes | ALM | 0.085 | 0.0066 | 5.39E-38 | 0.0028 | 0.0019 | 0.1435 |
| rs10974438 | Type 2 diabetes | ALM | -0.0514 | 0.0067 | 1.713E-14 | -0.0067 | 0.002 | 0.000767397 |
| rs11048456 | Type 2 diabetes | ALM | -0.0454 | 0.0072 | 3.295E-10 | 0.0038 | 0.0022 | 0.0797903 |
| rs11063029 | Type 2 diabetes | ALM | 0.0858 | 0.0138 | 5.364E-10 | -0.003 | 0.0043 | 0.4889 |
| rs11063069 | Type 2 diabetes | ALM | -0.056 | 0.0079 | 9.941E-13 | 0.0115 | 0.0023 | 8.38398E-07 |
| rs1117610 | Type 2 diabetes | ALM | -0.0423 | 0.0076 | 3.125E-08 | -0.033 | 0.0023 | 2.45923E-48 |
| rs11257655 | Type 2 diabetes | ALM | 0.0859 | 0.0077 | 1.455E-28 | 0.0019 | 0.0023 | 0.406 |
| rs1127215 | Type 2 diabetes | ALM | -0.0491 | 0.0065 | 3.918E-14 | -0.0011 | 0.0019 | 0.554799 |
| rs11496066 | Type 2 diabetes | ALM | 0.0508 | 0.0083 | 8.171E-10 | 0.0015 | 0.0024 | 0.529 |
| rs115505614 | Type 2 diabetes | ALM | 0.1657 | 0.0149 | 7.58E-29 | -0.0266 | 0.0043 | 9.09892E-10 |
| rs116425039 | Type 2 diabetes | ALM | -0.272 | 0.0348 | 5.084E-15 | -0.0004 | 0.0105 | 0.966 |
| rs11680058 | Type 2 diabetes | ALM | 0.0581 | 0.0104 | 2.081E-08 | -0.0051 | 0.003 | 0.091791 |
| rs11688682 | Type 2 diabetes | ALM | -0.0581 | 0.0076 | 2.928E-14 | 0.0033 | 0.0022 | 0.1318 |
| rs11699802 | Type 2 diabetes | ALM | -0.0443 | 0.0065 | 8.838E-12 | 0.005 | 0.0019 | 0.00835295 |
| rs11708067 | Type 2 diabetes | ALM | 0.0882 | 0.0077 | 5.046E-30 | 0.0125 | 0.0022 | 1.38599E-08 |
| rs11709077 | Type 2 diabetes | ALM | -0.104 | 0.0097 | 1.255E-26 | -0.0373 | 0.0029 | 1.13397E-37 |
| rs11759026 | Type 2 diabetes | ALM | -0.066 | 0.0075 | 2.042E-18 | -0.0336 | 0.0023 | 3.91381E-50 |
| rs11842871 | Type 2 diabetes | ALM | -0.04 | 0.0073 | 4.825E-08 | 0.0014 | 0.0022 | 0.5312 |
| rs11856307 | Type 2 diabetes | ALM | 0.047 | 0.0065 | 4.481E-13 | 0.0167 | 0.0019 | 3.84415E-18 |
| rs11958808 | Type 2 diabetes | ALM | 0.0403 | 0.0066 | 9.992E-10 | 0.0001 | 0.0019 | 0.9686 |
| rs12001437 | Type 2 diabetes | ALM | -0.0402 | 0.0066 | 1.099E-09 | -0.005 | 0.002 | 0.01007 |
| rs12140153 | Type 2 diabetes | ALM | -0.0645 | 0.0113 | 1.17E-08 | -0.0116 | 0.0033 | 0.000402698 |
| rs12325539 | Type 2 diabetes | ALM | -0.041 | 0.0065 | 2.686E-10 | -0.0275 | 0.0019 | 6.89128E-46 |
| rs1260326 | Type 2 diabetes | ALM | -0.0644 | 0.0066 | 1.621E-22 | -0.0323 | 0.0019 | 6.15886E-64 |
| rs12680692 | Type 2 diabetes | ALM | 0.0413 | 0.0071 | 6.602E-09 | 0.006 | 0.0021 | 0.00395503 |
| rs12719778 | Type 2 diabetes | ALM | 0.0381 | 0.0065 | 4.38E-09 | 0.0018 | 0.0019 | 0.3414 |
| rs12910361 | Type 2 diabetes | ALM | -0.0814 | 0.007 | 3.951E-31 | 0.007 | 0.0021 | 0.000856209 |
| rs12920022 | Type 2 diabetes | ALM | 0.0528 | 0.0092 | 1.001E-08 | 0.0137 | 0.0026 | 1.56599E-07 |
| rs13022337 | Type 2 diabetes | ALM | -0.0519 | 0.0088 | 3.611E-09 | -0.0319 | 0.0025 | 9.47764E-38 |
| rs13130484 | Type 2 diabetes | ALM | 0.0435 | 0.0067 | 8.492E-11 | 0.0016 | 0.0019 | 0.4153 |
| rs13330951 | Type 2 diabetes | ALM | 0.0361 | 0.0064 | 1.584E-08 | -0.0049 | 0.0019 | 0.00924209 |
| rs13385171 | Type 2 diabetes | ALM | 0.0357 | 0.0065 | 3.81E-08 | 0.0152 | 0.0019 | 1.01111E-15 |
| rs13389219 | Type 2 diabetes | ALM | -0.0605 | 0.0065 | 1.165E-20 | -0.0198 | 0.0019 | 4.51128E-25 |
| rs1359790 | Type 2 diabetes | ALM | -0.0817 | 0.0071 | 1.76E-30 | 0.0097 | 0.0021 | 3.92004E-06 |
| rs1381937 | Type 2 diabetes | ALM | 0.0442 | 0.0064 | 4.501E-12 | -0.0037 | 0.0019 | 0.0508698 |
| rs139688524 | Type 2 diabetes | ALM | 0.1739 | 0.0216 | 7.468E-16 | -0.003 | 0.0066 | 0.651799 |
| rs1412234 | Type 2 diabetes | ALM | -0.0393 | 0.0068 | 7.713E-09 | -0.0034 | 0.002 | 0.0927897 |
| rs141521721 | Type 2 diabetes | ALM | 0.1212 | 0.0214 | 1.392E-08 | -0.0056 | 0.0062 | 0.3653 |
| rs1426371 | Type 2 diabetes | ALM | -0.0517 | 0.0073 | 1.738E-12 | 0.028 | 0.0022 | 3.08603E-38 |
| rs1431841 | Type 2 diabetes | ALM | 0.0429 | 0.0077 | 3.078E-08 | -0.0003 | 0.0024 | 0.9084 |
| rs145678014 | Type 2 diabetes | ALM | -0.1048 | 0.0163 | 1.397E-10 | -0.0149 | 0.0046 | 0.001217 |
| rs1468906 | Type 2 diabetes | ALM | 0.0388 | 0.0067 | 7.029E-09 | -0.0001 | 0.002 | 0.9588 |
| rs1493694 | Type 2 diabetes | ALM | 0.08 | 0.0102 | 3.353E-15 | -0.0072 | 0.003 | 0.0167201 |
| rs1496653 | Type 2 diabetes | ALM | 0.0665 | 0.0079 | 2.487E-17 | -0.0038 | 0.0023 | 0.1047 |
| rs1517037 | Type 2 diabetes | ALM | -0.0451 | 0.0082 | 3.345E-08 | -0.0143 | 0.0024 | 3.99098E-09 |
| rs1561927 | Type 2 diabetes | ALM | -0.042 | 0.0072 | 6.115E-09 | -0.0065 | 0.0021 | 0.00256803 |
| rs1562396 | Type 2 diabetes | ALM | -0.0555 | 0.0069 | 9.635E-16 | -0.0161 | 0.002 | 2.273E-15 |
| rs1573090 | Type 2 diabetes | ALM | 0.0458 | 0.0065 | 1.722E-12 | 0.0011 | 0.0019 | 0.5747 |
| rs1665901 | Type 2 diabetes | ALM | 0.0398 | 0.0068 | 4.973E-09 | -0.008 | 0.002 | 6.87702E-05 |
| rs17030845 | Type 2 diabetes | ALM | -0.1191 | 0.0108 | 2.349E-28 | -0.0099 | 0.003 | 0.00102301 |
| rs1705263 | Type 2 diabetes | ALM | -0.0484 | 0.0064 | 3.507E-14 | 0.0083 | 0.0019 | 1.29601E-05 |
| rs1708302 | Type 2 diabetes | ALM | -0.0909 | 0.0064 | 5.769E-46 | -0.0052 | 0.0019 | 0.00569705 |
| rs17122772 | Type 2 diabetes | ALM | -0.0428 | 0.0077 | 3.313E-08 | 0.0002 | 0.0023 | 0.9234 |
| rs17168486 | Type 2 diabetes | ALM | 0.0672 | 0.0083 | 4.5E-16 | 0.002 | 0.0025 | 0.4221 |
| rs17522122 | Type 2 diabetes | ALM | 0.0356 | 0.0064 | 2.49E-08 | -0.0012 | 0.0019 | 0.5394 |
| rs17744783 | Type 2 diabetes | ALM | 0.0577 | 0.0103 | 1.873E-08 | 0.0136 | 0.0031 | 0.0000105 |
| rs17772814 | Type 2 diabetes | ALM | -0.0746 | 0.0125 | 2.133E-09 | -0.0036 | 0.0036 | 0.3199 |
| rs17791513 | Type 2 diabetes | ALM | 0.1016 | 0.0132 | 1.346E-14 | -0.0088 | 0.004 | 0.02666 |
| rs1783541 | Type 2 diabetes | ALM | 0.0608 | 0.0081 | 4.647E-14 | 0.0053 | 0.0023 | 0.0202801 |
| rs1800961 | Type 2 diabetes | ALM | 0.1602 | 0.0175 | 5.095E-20 | 0.0146 | 0.0055 | 0.00774408 |
| rs1999536 | Type 2 diabetes | ALM | -0.0404 | 0.0065 | 4.863E-10 | -0.0048 | 0.0019 | 0.0121899 |
| rs2023681 | Type 2 diabetes | ALM | -0.0826 | 0.0115 | 7.399E-13 | 0.011 | 0.0033 | 0.000803896 |
| rs2062213 | Type 2 diabetes | ALM | -0.0743 | 0.012 | 6.799E-10 | 0.0017 | 0.0035 | 0.6207 |
| rs2080385 | Type 2 diabetes | ALM | -0.0551 | 0.0074 | 1.244E-13 | -0.0024 | 0.0022 | 0.2847 |
| rs2107133 | Type 2 diabetes | ALM | 0.0643 | 0.0097 | 4.017E-11 | 0.0004 | 0.0027 | 0.892 |
| rs2215383 | Type 2 diabetes | ALM | -0.0641 | 0.0064 | 1.056E-23 | 0.0058 | 0.0019 | 0.002139 |
| rs2237895 | Type 2 diabetes | ALM | -0.0892 | 0.0066 | 1.147E-41 | -0.0064 | 0.0019 | 0.000917001 |
| rs2258238 | Type 2 diabetes | ALM | -0.102 | 0.0106 | 5.139E-22 | 0.0026 | 0.0031 | 0.4038 |
| rs2277536 | Type 2 diabetes | ALM | -0.0429 | 0.007 | 9.631E-10 | 0.0119 | 0.0021 | 1.31501E-08 |
| rs2290202 | Type 2 diabetes | ALM | 0.0666 | 0.0092 | 4.892E-13 | 0.0018 | 0.0028 | 0.5322 |
| rs2292662 | Type 2 diabetes | ALM | -0.0645 | 0.0088 | 2.239E-13 | -0.0044 | 0.0026 | 0.0928603 |
| rs231360 | Type 2 diabetes | ALM | 0.0576 | 0.0067 | 8.269E-18 | -0.0012 | 0.0019 | 0.536701 |
| rs2351707 | Type 2 diabetes | ALM | -0.0644 | 0.007 | 4.301E-20 | 0.0045 | 0.0021 | 0.0326896 |
| rs2383205 | Type 2 diabetes | ALM | -0.0533 | 0.0065 | 2.199E-16 | -0.0087 | 0.0019 | 6.01894E-06 |
| rs243019 | Type 2 diabetes | ALM | -0.0588 | 0.0064 | 3.372E-20 | -0.0034 | 0.0019 | 0.0745195 |
| rs2767036 | Type 2 diabetes | ALM | -0.0389 | 0.007 | 2.936E-08 | -0.0015 | 0.0021 | 0.4834 |
| rs2796441 | Type 2 diabetes | ALM | -0.0674 | 0.0065 | 2.972E-25 | 0.0036 | 0.0019 | 0.0566096 |
| rs2812545 | Type 2 diabetes | ALM | 0.0413 | 0.0065 | 1.99E-10 | 0.006 | 0.0019 | 0.00153402 |
| rs2820441 | Type 2 diabetes | ALM | 0.0556 | 0.0069 | 8.562E-16 | 0.0179 | 0.002 | 5.57314E-19 |
| rs28533815 | Type 2 diabetes | ALM | -0.0748 | 0.008 | 5.452E-21 | 0.0045 | 0.0022 | 0.0423097 |
| rs28663084 | Type 2 diabetes | ALM | -0.0376 | 0.0068 | 3.298E-08 | -0.0019 | 0.002 | 0.3351 |
| rs2896177 | Type 2 diabetes | ALM | -0.0367 | 0.0064 | 9.128E-09 | -0.0006 | 0.0019 | 0.739799 |
| rs2925979 | Type 2 diabetes | ALM | 0.0546 | 0.007 | 7.068E-15 | 0.0158 | 0.0021 | 1.77787E-14 |
| rs2972144 | Type 2 diabetes | ALM | -0.0911 | 0.0066 | 2.192E-43 | -0.0235 | 0.002 | 4.54255E-33 |
| rs3019208 | Type 2 diabetes | ALM | 0.0397 | 0.0068 | 5.431E-09 | -0.0001 | 0.002 | 0.9636 |
| rs3094682 | Type 2 diabetes | ALM | -0.0619 | 0.0082 | 3.469E-14 | 0.0205 | 0.0024 | 3.25537E-17 |
| rs320369 | Type 2 diabetes | ALM | 0.0372 | 0.0068 | 4.602E-08 | -0.0043 | 0.002 | 0.0348297 |
| rs329122 | Type 2 diabetes | ALM | 0.0366 | 0.0065 | 1.719E-08 | -0.0032 | 0.0019 | 0.0932911 |
| rs340874 | Type 2 diabetes | ALM | -0.0678 | 0.0065 | 1.555E-25 | 0.0007 | 0.0019 | 0.7251 |
| rs34341 | Type 2 diabetes | ALM | -0.044 | 0.0065 | 1.218E-11 | -0.0068 | 0.0019 | 0.000369301 |
| rs34715063 | Type 2 diabetes | ALM | -0.0772 | 0.0099 | 8.403E-15 | -0.0007 | 0.0028 | 0.8084 |
| rs348330 | Type 2 diabetes | ALM | -0.0492 | 0.0067 | 2.1E-13 | 0.0057 | 0.0019 | 0.003498 |
| rs34990153 | Type 2 diabetes | ALM | 0.0476 | 0.0065 | 2.257E-13 | 0.0031 | 0.0019 | 0.0992407 |
| rs35011184 | Type 2 diabetes | ALM | 0.2822 | 0.0075 | 1E-200 | 0.0057 | 0.0023 | 0.01239 |
| rs35318451 | Type 2 diabetes | ALM | 0.0478 | 0.007 | 9.498E-12 | 0.0031 | 0.002 | 0.1214 |
| rs35777422 | Type 2 diabetes | ALM | -0.0381 | 0.0067 | 1.302E-08 | 0.0137 | 0.002 | 5.44127E-12 |
| rs35895680 | Type 2 diabetes | ALM | -0.0554 | 0.0069 | 1.084E-15 | 0.0076 | 0.002 | 0.000167402 |
| rs36138276 | Type 2 diabetes | ALM | -0.0431 | 0.0066 | 6.403E-11 | 0.0022 | 0.0019 | 0.2618 |
| rs3768321 | Type 2 diabetes | ALM | 0.084 | 0.008 | 4.753E-26 | 0.0123 | 0.0024 | 1.99301E-07 |
| rs3783394 | Type 2 diabetes | ALM | -0.038 | 0.0067 | 1.421E-08 | 0.0023 | 0.002 | 0.2587 |
| rs3786900 | Type 2 diabetes | ALM | 0.0434 | 0.0072 | 1.886E-09 | 0.0131 | 0.0021 | 1.037E-09 |
| rs3798519 | Type 2 diabetes | ALM | -0.0616 | 0.0082 | 4.602E-14 | -0.0124 | 0.0025 | 4.36395E-07 |
| rs3802177 | Type 2 diabetes | ALM | -0.1077 | 0.0069 | 9.163E-55 | 0.0013 | 0.002 | 0.5107 |
| rs429358 | Type 2 diabetes | ALM | 0.0746 | 0.0091 | 2.621E-16 | -0.006 | 0.0026 | 0.0213801 |
| rs4368494 | Type 2 diabetes | ALM | -0.054 | 0.007 | 1.385E-14 | -0.0066 | 0.0021 | 0.001532 |
| rs459193 | Type 2 diabetes | ALM | -0.0722 | 0.0073 | 6.783E-23 | -0.0139 | 0.0022 | 1.196E-10 |
| rs4686471 | Type 2 diabetes | ALM | -0.0608 | 0.0066 | 3.045E-20 | 0.0057 | 0.0019 | 0.003298 |
| rs4688760 | Type 2 diabetes | ALM | 0.042 | 0.007 | 2.136E-09 | 0.0062 | 0.002 | 0.00234498 |
| rs4709746 | Type 2 diabetes | ALM | -0.0567 | 0.0096 | 3.951E-09 | 0.0134 | 0.0028 | 1.47901E-06 |
| rs474513 | Type 2 diabetes | ALM | 0.0399 | 0.0064 | 4.178E-10 | -0.02 | 0.0019 | 3.12176E-26 |
| rs4804833 | Type 2 diabetes | ALM | 0.048 | 0.0066 | 3.417E-13 | 0.0157 | 0.0019 | 7.45933E-16 |
| rs4865436 | Type 2 diabetes | ALM | -0.049 | 0.0075 | 8.037E-11 | 0.0008 | 0.0022 | 0.714301 |
| rs490689 | Type 2 diabetes | ALM | 0.0543 | 0.008 | 8.863E-12 | 0.017 | 0.0023 | 1.50314E-13 |
| rs4925109 | Type 2 diabetes | ALM | 0.0476 | 0.0069 | 5.65E-12 | -0.0073 | 0.0021 | 0.000400599 |
| rs4929965 | Type 2 diabetes | ALM | 0.0668 | 0.0068 | 9.669E-23 | 0.0072 | 0.002 | 0.000217701 |
| rs4977213 | Type 2 diabetes | ALM | -0.0507 | 0.0069 | 2.187E-13 | 0.0064 | 0.0019 | 0.00109199 |
| rs505922 | Type 2 diabetes | ALM | -0.0473 | 0.0068 | 3.65E-12 | 0.0094 | 0.002 | 3.41602E-06 |
| rs508419 | Type 2 diabetes | ALM | -0.0811 | 0.0075 | 5.433E-27 | -0.0011 | 0.0022 | 0.623199 |
| rs5215 | Type 2 diabetes | ALM | -0.0706 | 0.0066 | 9.848E-27 | 0.0007 | 0.002 | 0.7364 |
| rs55653563 | Type 2 diabetes | ALM | 0.0435 | 0.0072 | 1.731E-09 | -0.0071 | 0.0021 | 0.000895901 |
| rs55872725 | Type 2 diabetes | ALM | 0.122 | 0.0065 | 8.509E-79 | 0.0222 | 0.0019 | 1.46285E-30 |
| rs56187241 | Type 2 diabetes | ALM | 0.1014 | 0.0157 | 1.072E-10 | 0.0064 | 0.0049 | 0.1904 |
| rs56348580 | Type 2 diabetes | ALM | -0.0617 | 0.0069 | 4.313E-19 | -0.0036 | 0.002 | 0.0755005 |
| rs5758223 | Type 2 diabetes | ALM | 0.0401 | 0.0071 | 1.779E-08 | 0.0011 | 0.0021 | 0.5937 |
| rs576674 | Type 2 diabetes | ALM | -0.0538 | 0.0086 | 3.697E-10 | -0.0027 | 0.0025 | 0.2814 |
| rs58432198 | Type 2 diabetes | ALM | -0.0636 | 0.0103 | 5.707E-10 | 0.0277 | 0.0029 | 3.66691E-21 |
| rs58642235 | Type 2 diabetes | ALM | -0.0566 | 0.0094 | 1.896E-09 | 0.0078 | 0.0028 | 0.00501095 |
| rs59147390 | Type 2 diabetes | ALM | 0.0648 | 0.0105 | 6.056E-10 | 0.0009 | 0.0029 | 0.7484 |
| rs601945 | Type 2 diabetes | ALM | -0.0849 | 0.0086 | 4.662E-23 | 0.0207 | 0.0024 | 7.38754E-18 |
| rs61676547 | Type 2 diabetes | ALM | 0.0525 | 0.0081 | 7.417E-11 | -0.001 | 0.0024 | 0.6723 |
| rs62107261 | Type 2 diabetes | ALM | 0.1019 | 0.0161 | 2.621E-10 | 0.0499 | 0.0044 | 4.64195E-30 |
| rs62271373 | Type 2 diabetes | ALM | 0.0882 | 0.0144 | 1.033E-09 | 0.023 | 0.004 | 1.21099E-08 |
| rs62563593 | Type 2 diabetes | ALM | -0.0394 | 0.0065 | 1.284E-09 | -0.0015 | 0.0019 | 0.4544 |
| rs6444809 | Type 2 diabetes | ALM | 0.0544 | 0.0096 | 1.628E-08 | -0.0041 | 0.0029 | 0.1566 |
| rs6459737 | Type 2 diabetes | ALM | -0.0583 | 0.0067 | 3.311E-18 | 0.0011 | 0.002 | 0.592899 |
| rs648795 | Type 2 diabetes | ALM | 0.0391 | 0.0065 | 1.71E-09 | 0.0018 | 0.0019 | 0.345 |
| rs6545714 | Type 2 diabetes | ALM | -0.0363 | 0.0065 | 2.246E-08 | -0.0034 | 0.0019 | 0.0747205 |
| rs6600191 | Type 2 diabetes | ALM | 0.0587 | 0.0085 | 4.471E-12 | -0.0067 | 0.0025 | 0.00671104 |
| rs6687271 | Type 2 diabetes | ALM | -0.0402 | 0.0073 | 4.136E-08 | 0.0028 | 0.0022 | 0.2053 |
| rs672271 | Type 2 diabetes | ALM | -0.06 | 0.011 | 4.825E-08 | 0.0018 | 0.0033 | 0.5918 |
| rs67232546 | Type 2 diabetes | ALM | 0.0531 | 0.008 | 2.505E-11 | -0.0012 | 0.0023 | 0.6128 |
| rs6821438 | Type 2 diabetes | ALM | 0.0397 | 0.0064 | 5.103E-10 | 0.0004 | 0.0019 | 0.8375 |
| rs6885132 | Type 2 diabetes | ALM | 0.077 | 0.011 | 2.488E-12 | 0.0022 | 0.0032 | 0.4837 |
| rs6937438 | Type 2 diabetes | ALM | -0.0514 | 0.007 | 2.352E-13 | 0.0057 | 0.0021 | 0.00555098 |
| rs702634 | Type 2 diabetes | ALM | 0.0503 | 0.0069 | 3.365E-13 | -0.0001 | 0.002 | 0.9496 |
| rs703972 | Type 2 diabetes | ALM | -0.0698 | 0.0065 | 5.771E-27 | 0.0039 | 0.0019 | 0.0382904 |
| rs7178762 | Type 2 diabetes | ALM | -0.0387 | 0.0064 | 1.367E-09 | 0.0025 | 0.0019 | 0.1943 |
| rs7240767 | Type 2 diabetes | ALM | -0.0372 | 0.0066 | 1.705E-08 | 0.0013 | 0.002 | 0.5104 |
| rs72802358 | Type 2 diabetes | ALM | -0.1135 | 0.0107 | 2.215E-26 | 0.0169 | 0.0031 | 7.47601E-08 |
| rs7313918 | Type 2 diabetes | ALM | -0.0559 | 0.0094 | 2.99E-09 | 0.0042 | 0.0029 | 0.1391 |
| rs7325671 | Type 2 diabetes | ALM | 0.0545 | 0.0096 | 1.533E-08 | 0.0013 | 0.0028 | 0.6556 |
| rs739846 | Type 2 diabetes | ALM | 0.0877 | 0.0119 | 2.02E-13 | -0.0255 | 0.0036 | 1.20893E-12 |
| rs745805 | Type 2 diabetes | ALM | 0.0632 | 0.0084 | 4.516E-14 | 0.001 | 0.0025 | 0.6762 |
| rs7568172 | Type 2 diabetes | ALM | -0.0827 | 0.0135 | 9.187E-10 | 0.0026 | 0.0039 | 0.5068 |
| rs7572970 | Type 2 diabetes | ALM | -0.0447 | 0.0072 | 6.12E-10 | 0.0054 | 0.0021 | 0.00928795 |
| rs7633675 | Type 2 diabetes | ALM | -0.1085 | 0.0068 | 3.206E-57 | 0.0062 | 0.002 | 0.00225201 |
| rs7667864 | Type 2 diabetes | ALM | -0.0402 | 0.0071 | 1.64E-08 | 0.0339 | 0.0021 | 5.76235E-59 |
| rs7669833 | Type 2 diabetes | ALM | -0.0572 | 0.007 | 3.524E-16 | -0.0023 | 0.0021 | 0.265 |
| rs76895963 | Type 2 diabetes | ALM | 0.4826 | 0.0275 | 9.124E-69 | -0.1639 | 0.0073 | 8.2224E-112 |
| rs7732130 | Type 2 diabetes | ALM | -0.0606 | 0.007 | 5.693E-18 | 0.0052 | 0.002 | 0.0097609 |
| rs77464186 | Type 2 diabetes | ALM | 0.1002 | 0.0087 | 9.304E-31 | -0.0107 | 0.0026 | 4.27996E-05 |
| rs77864822 | Type 2 diabetes | ALM | 0.0753 | 0.0129 | 5.005E-09 | -0.0109 | 0.0039 | 0.00488495 |
| rs7966976 | Type 2 diabetes | ALM | -0.0751 | 0.0081 | 1.217E-20 | 0.0019 | 0.0024 | 0.4174 |
| rs79920718 | Type 2 diabetes | ALM | 0.0389 | 0.0067 | 6.431E-09 | 0.0018 | 0.002 | 0.3643 |
| rs8008910 | Type 2 diabetes | ALM | 0.0554 | 0.0076 | 4.223E-13 | 0.0037 | 0.0023 | 0.105 |
| rs8071043 | Type 2 diabetes | ALM | -0.0523 | 0.0068 | 1.533E-14 | -0.0003 | 0.002 | 0.8914 |
| rs8097210 | Type 2 diabetes | ALM | -0.0537 | 0.0072 | 1.061E-13 | -0.0422 | 0.0022 | 1.36616E-85 |
| rs8192675 | Type 2 diabetes | ALM | 0.0659 | 0.007 | 5.772E-21 | -0.0055 | 0.0021 | 0.00837896 |
| rs878521 | Type 2 diabetes | ALM | 0.0618 | 0.0074 | 9.308E-17 | -0.0075 | 0.0022 | 0.000538406 |
| rs9304665 | Type 2 diabetes | ALM | 0.0418 | 0.0076 | 4.529E-08 | 0.0077 | 0.0022 | 0.000599294 |
| rs9368222 | Type 2 diabetes | ALM | 0.1379 | 0.0071 | 1.42E-83 | -0.0047 | 0.0021 | 0.0292799 |
| rs9379084 | Type 2 diabetes | ALM | -0.0994 | 0.0106 | 5.478E-21 | -0.0257 | 0.003 | 2.37575E-17 |
| rs9563615 | Type 2 diabetes | ALM | 0.0408 | 0.0072 | 1.628E-08 | 0.004 | 0.0021 | 0.0570099 |
| rs9665898 | Type 2 diabetes | ALM | -0.0525 | 0.0094 | 2.528E-08 | -0.0005 | 0.0028 | 0.849 |
| rs9873519 | Type 2 diabetes | ALM | 0.0371 | 0.0064 | 6.292E-09 | 0.0029 | 0.0019 | 0.1224 |
| rs1007090 | Type 2 diabetes | Usual walking pace | -0.044 | 0.0068 | 1.01E-10 | -0.000916997 | 0.00132988 | 0.49 |
| rs10097617 | Type 2 diabetes | Usual walking pace | 0.0487 | 0.0064 | 2.44E-14 | -0.00175349 | 0.00126815 | 0.17 |
| rs10406431 | Type 2 diabetes | Usual walking pace | 0.0603 | 0.0065 | 1.56E-20 | -0.00269216 | 0.0012728 | 0.0340001 |
| rs10419627 | Type 2 diabetes | Usual walking pace | 0.0431 | 0.0065 | 3.15E-11 | -0.000986303 | 0.00128274 | 0.44 |
| rs1061810 | Type 2 diabetes | Usual walking pace | 0.0502 | 0.007 | 8.31E-13 | -0.0057742 | 0.00139205 | 3.40E-05 |
| rs10750397 | Type 2 diabetes | Usual walking pace | 0.0394 | 0.0071 | 3.13E-08 | -0.00161522 | 0.00141591 | 0.25 |
| rs10811660 | Type 2 diabetes | Usual walking pace | -0.1598 | 0.0086 | 2.54E-77 | 0.000277014 | 0.00167288 | 0.87 |
| rs10830963 | Type 2 diabetes | Usual walking pace | -0.101 | 0.0071 | 1.12E-45 | 0.00124929 | 0.0014156 | 0.38 |
| rs10882099 | Type 2 diabetes | Usual walking pace | 0.1095 | 0.0065 | 7.73E-64 | 0.00111672 | 0.00128801 | 0.39 |
| rs10908278 | Type 2 diabetes | Usual walking pace | -0.0749 | 0.0067 | 5.25E-29 | -0.00125708 | 0.0012736 | 0.32 |
| rs10937721 | Type 2 diabetes | Usual walking pace | 0.085 | 0.0066 | 5.39E-38 | -0.00156156 | 0.00129673 | 0.23 |
| rs11048456 | Type 2 diabetes | Usual walking pace | -0.0454 | 0.0072 | 3.30E-10 | -0.00270627 | 0.00147277 | 0.0659994 |
| rs11063029 | Type 2 diabetes | Usual walking pace | 0.0858 | 0.0138 | 5.36E-10 | -0.00185158 | 0.00288706 | 0.52 |
| rs11063069 | Type 2 diabetes | Usual walking pace | -0.056 | 0.0079 | 9.94E-13 | 0.000536172 | 0.00156488 | 0.73 |
| rs11257655 | Type 2 diabetes | Usual walking pace | 0.0859 | 0.0077 | 1.46E-28 | -0.000221098 | 0.00155854 | 0.89 |
| rs1127215 | Type 2 diabetes | Usual walking pace | -0.0491 | 0.0065 | 3.92E-14 | -0.00093589 | 0.00128143 | 0.47 |
| rs11496066 | Type 2 diabetes | Usual walking pace | 0.0508 | 0.0083 | 8.17E-10 | 0.00172238 | 0.00162485 | 0.29 |
| rs115505614 | Type 2 diabetes | Usual walking pace | 0.1657 | 0.0149 | 7.58E-29 | -0.00226439 | 0.00292041 | 0.44 |
| rs116425039 | Type 2 diabetes | Usual walking pace | -0.272 | 0.0348 | 5.08E-15 | 0.00130018 | 0.00707142 | 0.85 |
| rs11680058 | Type 2 diabetes | Usual walking pace | 0.0581 | 0.0104 | 2.08E-08 | -0.00204586 | 0.00202621 | 0.31 |
| rs11688682 | Type 2 diabetes | Usual walking pace | -0.0581 | 0.0076 | 2.93E-14 | 0.00154018 | 0.00147195 | 0.3 |
| rs11699802 | Type 2 diabetes | Usual walking pace | -0.0443 | 0.0065 | 8.84E-12 | 0.000578364 | 0.00127694 | 0.649999 |
| rs11759026 | Type 2 diabetes | Usual walking pace | -0.066 | 0.0075 | 2.04E-18 | -0.0033734 | 0.00151379 | 0.0259998 |
| rs11842871 | Type 2 diabetes | Usual walking pace | -0.04 | 0.0073 | 4.83E-08 | 0.00455897 | 0.00144537 | 0.0016 |
| rs11856307 | Type 2 diabetes | Usual walking pace | 0.047 | 0.0065 | 4.48E-13 | 2.88E-05 | 0.00128188 | 0.98 |
| rs11958808 | Type 2 diabetes | Usual walking pace | 0.0403 | 0.0066 | 9.99E-10 | 0.00150137 | 0.00129845 | 0.25 |
| rs12001437 | Type 2 diabetes | Usual walking pace | -0.0402 | 0.0066 | 1.10E-09 | 0.00236095 | 0.00131271 | 0.0719996 |
| rs12140153 | Type 2 diabetes | Usual walking pace | -0.0645 | 0.0113 | 1.17E-08 | 0.0070444 | 0.00222048 | 0.0015 |
| rs12325539 | Type 2 diabetes | Usual walking pace | -0.041 | 0.0065 | 2.69E-10 | 0.000619056 | 0.00129038 | 0.630001 |
| rs1260326 | Type 2 diabetes | Usual walking pace | -0.0644 | 0.0066 | 1.62E-22 | -0.000938332 | 0.00129237 | 0.47 |
| rs12680692 | Type 2 diabetes | Usual walking pace | 0.0413 | 0.0071 | 6.60E-09 | -0.00206529 | 0.00139376 | 0.14 |
| rs12719778 | Type 2 diabetes | Usual walking pace | 0.0381 | 0.0065 | 4.38E-09 | 0.00233077 | 0.00126857 | 0.0659994 |
| rs12910361 | Type 2 diabetes | Usual walking pace | -0.0814 | 0.007 | 3.95E-31 | 0.000714029 | 0.00139795 | 0.61 |
| rs12920022 | Type 2 diabetes | Usual walking pace | 0.0528 | 0.0092 | 1.00E-08 | -0.00159778 | 0.00174779 | 0.36 |
| rs13330951 | Type 2 diabetes | Usual walking pace | 0.0361 | 0.0064 | 1.58E-08 | -0.0049324 | 0.00126922 | 0.0001 |
| rs13385171 | Type 2 diabetes | Usual walking pace | 0.0357 | 0.0065 | 3.81E-08 | 0.0010042 | 0.00128217 | 0.43 |
| rs13389219 | Type 2 diabetes | Usual walking pace | -0.0605 | 0.0065 | 1.17E-20 | -0.000584529 | 0.00129529 | 0.649999 |
| rs1359790 | Type 2 diabetes | Usual walking pace | -0.0817 | 0.0071 | 1.76E-30 | -0.00236951 | 0.00139945 | 0.0899995 |
| rs1381937 | Type 2 diabetes | Usual walking pace | 0.0442 | 0.0064 | 4.50E-12 | 0.0020995 | 0.00127458 | 0.1 |
| rs139688524 | Type 2 diabetes | Usual walking pace | 0.1739 | 0.0216 | 7.47E-16 | -0.000229395 | 0.00439265 | 0.96 |
| rs1412234 | Type 2 diabetes | Usual walking pace | -0.0393 | 0.0068 | 7.71E-09 | 0.00368669 | 0.00135182 | 0.0064 |
| rs141521721 | Type 2 diabetes | Usual walking pace | 0.1212 | 0.0214 | 1.39E-08 | -0.000356708 | 0.00412687 | 0.93 |
| rs1426371 | Type 2 diabetes | Usual walking pace | -0.0517 | 0.0073 | 1.74E-12 | 0.00292146 | 0.001456 | 0.0449997 |
| rs1431841 | Type 2 diabetes | Usual walking pace | 0.0429 | 0.0077 | 3.08E-08 | -0.00426175 | 0.00157274 | 0.00669993 |
| rs145678014 | Type 2 diabetes | Usual walking pace | -0.1048 | 0.0163 | 1.40E-10 | 0.00847904 | 0.00308045 | 0.00589997 |
| rs1468906 | Type 2 diabetes | Usual walking pace | 0.0388 | 0.0067 | 7.03E-09 | 0.000887183 | 0.00132831 | 0.5 |
| rs1493694 | Type 2 diabetes | Usual walking pace | 0.08 | 0.0102 | 3.35E-15 | -0.00281062 | 0.00203823 | 0.17 |
| rs1496653 | Type 2 diabetes | Usual walking pace | 0.0665 | 0.0079 | 2.49E-17 | -0.00012112 | 0.00157018 | 0.94 |
| rs1517037 | Type 2 diabetes | Usual walking pace | -0.0451 | 0.0082 | 3.35E-08 | 0.000230958 | 0.00162123 | 0.89 |
| rs1561927 | Type 2 diabetes | Usual walking pace | -0.042 | 0.0072 | 6.12E-09 | -0.00137493 | 0.00143635 | 0.34 |
| rs1562396 | Type 2 diabetes | Usual walking pace | -0.0555 | 0.0069 | 9.64E-16 | -0.00232508 | 0.00135867 | 0.0870001 |
| rs1573090 | Type 2 diabetes | Usual walking pace | 0.0458 | 0.0065 | 1.72E-12 | 9.55E-05 | 0.00129938 | 0.94 |
| rs1665901 | Type 2 diabetes | Usual walking pace | 0.0398 | 0.0068 | 4.97E-09 | -0.00228427 | 0.00134566 | 0.0899995 |
| rs17030845 | Type 2 diabetes | Usual walking pace | -0.1191 | 0.0108 | 2.35E-28 | 0.00173479 | 0.00204555 | 0.4 |
| rs1705263 | Type 2 diabetes | Usual walking pace | -0.0484 | 0.0064 | 3.51E-14 | -0.000111315 | 0.00127582 | 0.93 |
| rs1708302 | Type 2 diabetes | Usual walking pace | -0.0909 | 0.0064 | 5.77E-46 | -0.00170051 | 0.00126544 | 0.18 |
| rs17122772 | Type 2 diabetes | Usual walking pace | -0.0428 | 0.0077 | 3.31E-08 | -0.00110728 | 0.0015065 | 0.46 |
| rs17168486 | Type 2 diabetes | Usual walking pace | 0.0672 | 0.0083 | 4.50E-16 | -0.00113194 | 0.00167947 | 0.5 |
| rs17744783 | Type 2 diabetes | Usual walking pace | 0.0577 | 0.0103 | 1.87E-08 | -0.0011176 | 0.00206297 | 0.59 |
| rs17772814 | Type 2 diabetes | Usual walking pace | -0.0746 | 0.0125 | 2.13E-09 | -0.00198983 | 0.00241871 | 0.41 |
| rs17791513 | Type 2 diabetes | Usual walking pace | 0.1016 | 0.0132 | 1.35E-14 | -0.00912511 | 0.00265051 | 0.000580003 |
| rs1783541 | Type 2 diabetes | Usual walking pace | 0.0608 | 0.0081 | 4.65E-14 | -0.00461117 | 0.00153548 | 0.00269998 |
| rs1800961 | Type 2 diabetes | Usual walking pace | 0.1602 | 0.0175 | 5.10E-20 | 0.00239528 | 0.00365534 | 0.51 |
| rs1999536 | Type 2 diabetes | Usual walking pace | -0.0404 | 0.0065 | 4.86E-10 | -0.00121933 | 0.00128262 | 0.34 |
| rs2023681 | Type 2 diabetes | Usual walking pace | -0.0826 | 0.0115 | 7.40E-13 | -0.00139629 | 0.00219183 | 0.52 |
| rs2062213 | Type 2 diabetes | Usual walking pace | -0.0743 | 0.012 | 6.80E-10 | 0.00837637 | 0.00236356 | 0.000389996 |
| rs2080385 | Type 2 diabetes | Usual walking pace | -0.0551 | 0.0074 | 1.24E-13 | -0.00032729 | 0.0014841 | 0.83 |
| rs2107133 | Type 2 diabetes | Usual walking pace | 0.0643 | 0.0097 | 4.02E-11 | -0.00171167 | 0.00184082 | 0.35 |
| rs2215383 | Type 2 diabetes | Usual walking pace | -0.0641 | 0.0064 | 1.06E-23 | -0.0015098 | 0.00127239 | 0.24 |
| rs2237895 | Type 2 diabetes | Usual walking pace | -0.0892 | 0.0066 | 1.15E-41 | -0.00271856 | 0.00128189 | 0.0340001 |
| rs2258238 | Type 2 diabetes | Usual walking pace | -0.102 | 0.0106 | 5.14E-22 | 0.00425566 | 0.00205628 | 0.0379997 |
| rs2277536 | Type 2 diabetes | Usual walking pace | -0.0429 | 0.007 | 9.63E-10 | -0.00319615 | 0.00139854 | 0.0219999 |
| rs2290202 | Type 2 diabetes | Usual walking pace | 0.0666 | 0.0092 | 4.89E-13 | 0.00286626 | 0.00188863 | 0.13 |
| rs2292662 | Type 2 diabetes | Usual walking pace | -0.0645 | 0.0088 | 2.24E-13 | 0.00224464 | 0.00177158 | 0.21 |
| rs231360 | Type 2 diabetes | Usual walking pace | 0.0576 | 0.0067 | 8.27E-18 | 0.0014184 | 0.00130114 | 0.28 |
| rs2351707 | Type 2 diabetes | Usual walking pace | -0.0644 | 0.007 | 4.30E-20 | 0.00199927 | 0.00140882 | 0.16 |
| rs2383205 | Type 2 diabetes | Usual walking pace | -0.0533 | 0.0065 | 2.20E-16 | 0.000979734 | 0.00129391 | 0.450001 |
| rs243019 | Type 2 diabetes | Usual walking pace | -0.0588 | 0.0064 | 3.37E-20 | -9.24E-05 | 0.00127419 | 0.94 |
| rs2767036 | Type 2 diabetes | Usual walking pace | -0.0389 | 0.007 | 2.94E-08 | -0.000505824 | 0.0013993 | 0.719999 |
| rs2796441 | Type 2 diabetes | Usual walking pace | -0.0674 | 0.0065 | 2.97E-25 | 4.40E-05 | 0.0012808 | 0.97 |
| rs2812545 | Type 2 diabetes | Usual walking pace | 0.0413 | 0.0065 | 1.99E-10 | 0.00060816 | 0.00127766 | 0.630001 |
| rs2820441 | Type 2 diabetes | Usual walking pace | 0.0556 | 0.0069 | 8.56E-16 | 0.00335392 | 0.00135913 | 0.0140001 |
| rs28533815 | Type 2 diabetes | Usual walking pace | -0.0748 | 0.008 | 5.45E-21 | 0.00379406 | 0.00147231 | 0.01 |
| rs28663084 | Type 2 diabetes | Usual walking pace | -0.0376 | 0.0068 | 3.30E-08 | -0.00138693 | 0.00135086 | 0.3 |
| rs2896177 | Type 2 diabetes | Usual walking pace | -0.0367 | 0.0064 | 9.13E-09 | 0.00102309 | 0.0012702 | 0.42 |
| rs2925979 | Type 2 diabetes | Usual walking pace | 0.0546 | 0.007 | 7.07E-15 | -0.00256232 | 0.00138183 | 0.064 |
| rs2972144 | Type 2 diabetes | Usual walking pace | -0.0911 | 0.0066 | 2.19E-43 | 0.000633145 | 0.00132359 | 0.630001 |
| rs3019208 | Type 2 diabetes | Usual walking pace | 0.0397 | 0.0068 | 5.43E-09 | -0.000221035 | 0.0013601 | 0.87 |
| rs3094682 | Type 2 diabetes | Usual walking pace | -0.0619 | 0.0082 | 3.47E-14 | 0.00219787 | 0.0016356 | 0.18 |
| rs320369 | Type 2 diabetes | Usual walking pace | 0.0372 | 0.0068 | 4.60E-08 | 0.000421979 | 0.00136855 | 0.760001 |
| rs329122 | Type 2 diabetes | Usual walking pace | 0.0366 | 0.0065 | 1.72E-08 | 0.00134601 | 0.00128396 | 0.29 |
| rs340874 | Type 2 diabetes | Usual walking pace | -0.0678 | 0.0065 | 1.56E-25 | -0.00113347 | 0.00127494 | 0.37 |
| rs34715063 | Type 2 diabetes | Usual walking pace | -0.0772 | 0.0099 | 8.40E-15 | 0.00335528 | 0.00190345 | 0.0779992 |
| rs348330 | Type 2 diabetes | Usual walking pace | -0.0492 | 0.0067 | 2.10E-13 | 0.000661727 | 0.00131683 | 0.62 |
| rs34990153 | Type 2 diabetes | Usual walking pace | 0.0476 | 0.0065 | 2.26E-13 | -0.00483428 | 0.00127874 | 0.00016 |
| rs35011184 | Type 2 diabetes | Usual walking pace | 0.2822 | 0.0075 | 1.00E-200 | 0.00070184 | 0.0015134 | 0.64 |
| rs35318451 | Type 2 diabetes | Usual walking pace | 0.0478 | 0.007 | 9.50E-12 | 0.00071426 | 0.00134958 | 0.6 |
| rs35777422 | Type 2 diabetes | Usual walking pace | -0.0381 | 0.0067 | 1.30E-08 | 0.00142556 | 0.00132878 | 0.28 |
| rs36138276 | Type 2 diabetes | Usual walking pace | -0.0431 | 0.0066 | 6.40E-11 | -0.000125479 | 0.00128158 | 0.92 |
| rs3786900 | Type 2 diabetes | Usual walking pace | 0.0434 | 0.0072 | 1.89E-09 | 0.00156667 | 0.00143215 | 0.27 |
| rs3802177 | Type 2 diabetes | Usual walking pace | -0.1077 | 0.0069 | 9.16E-55 | 0.00366252 | 0.00137081 | 0.00749998 |
| rs4368494 | Type 2 diabetes | Usual walking pace | -0.054 | 0.007 | 1.39E-14 | -0.000882489 | 0.00138644 | 0.52 |
| rs459193 | Type 2 diabetes | Usual walking pace | -0.0722 | 0.0073 | 6.78E-23 | 0.0013088 | 0.00145196 | 0.37 |
| rs4686471 | Type 2 diabetes | Usual walking pace | -0.0608 | 0.0066 | 3.05E-20 | 0.000795169 | 0.00130319 | 0.54 |
| rs4709746 | Type 2 diabetes | Usual walking pace | -0.0567 | 0.0096 | 3.95E-09 | -0.00441871 | 0.00187 | 0.0179999 |
| rs474513 | Type 2 diabetes | Usual walking pace | 0.0399 | 0.0064 | 4.18E-10 | -0.00128983 | 0.00126786 | 0.31 |
| rs4804833 | Type 2 diabetes | Usual walking pace | 0.048 | 0.0066 | 3.42E-13 | 0.00115593 | 0.0012984 | 0.37 |
| rs4812034 | Type 2 diabetes | Usual walking pace | 0.0416 | 0.0064 | 7.35E-11 | -0.00131074 | 0.00127125 | 0.3 |
| rs4865436 | Type 2 diabetes | Usual walking pace | -0.049 | 0.0075 | 8.04E-11 | -0.00183383 | 0.00145332 | 0.21 |
| rs4925109 | Type 2 diabetes | Usual walking pace | 0.0476 | 0.0069 | 5.65E-12 | -0.0047293 | 0.00137992 | 0.00061 |
| rs4929965 | Type 2 diabetes | Usual walking pace | 0.0668 | 0.0068 | 9.67E-23 | 0.00162516 | 0.00130647 | 0.21 |
| rs4977213 | Type 2 diabetes | Usual walking pace | -0.0507 | 0.0069 | 2.19E-13 | 0.00131963 | 0.00130469 | 0.31 |
| rs505922 | Type 2 diabetes | Usual walking pace | -0.0473 | 0.0068 | 3.65E-12 | 0.000498924 | 0.00135729 | 0.709999 |
| rs508419 | Type 2 diabetes | Usual walking pace | -0.0811 | 0.0075 | 5.43E-27 | 0.000531417 | 0.00147833 | 0.719999 |
| rs5215 | Type 2 diabetes | Usual walking pace | -0.0706 | 0.0066 | 9.85E-27 | -0.00251819 | 0.00132054 | 0.0569994 |
| rs55653563 | Type 2 diabetes | Usual walking pace | 0.0435 | 0.0072 | 1.73E-09 | -0.000953466 | 0.00143679 | 0.51 |
| rs56187241 | Type 2 diabetes | Usual walking pace | 0.1014 | 0.0157 | 1.07E-10 | 0.0026555 | 0.00327715 | 0.42 |
| rs56348580 | Type 2 diabetes | Usual walking pace | -0.0617 | 0.0069 | 4.31E-19 | 0.00246053 | 0.00137486 | 0.0739997 |
| rs5758223 | Type 2 diabetes | Usual walking pace | 0.0401 | 0.0071 | 1.78E-08 | 0.0014988 | 0.00141217 | 0.29 |
| rs576674 | Type 2 diabetes | Usual walking pace | -0.0538 | 0.0086 | 3.70E-10 | -0.000893247 | 0.00169147 | 0.6 |
| rs58432198 | Type 2 diabetes | Usual walking pace | -0.0636 | 0.0103 | 5.71E-10 | -0.00358231 | 0.00198119 | 0.0710003 |
| rs58642235 | Type 2 diabetes | Usual walking pace | -0.0566 | 0.0094 | 1.90E-09 | 0.00263939 | 0.00184885 | 0.15 |
| rs59147390 | Type 2 diabetes | Usual walking pace | 0.0648 | 0.0105 | 6.06E-10 | -0.00418789 | 0.00202081 | 0.0379997 |
| rs601945 | Type 2 diabetes | Usual walking pace | -0.0849 | 0.0086 | 4.66E-23 | 0.00586418 | 0.00161682 | 0.000290001 |
| rs61676547 | Type 2 diabetes | Usual walking pace | 0.0525 | 0.0081 | 7.42E-11 | -0.00202718 | 0.00162464 | 0.21 |
| rs62107261 | Type 2 diabetes | Usual walking pace | 0.1019 | 0.0161 | 2.62E-10 | -0.00829042 | 0.00295922 | 0.0051 |
| rs62271373 | Type 2 diabetes | Usual walking pace | 0.0882 | 0.0144 | 1.03E-09 | -0.00233815 | 0.00271506 | 0.39 |
| rs62563593 | Type 2 diabetes | Usual walking pace | -0.0394 | 0.0065 | 1.28E-09 | 0.000227372 | 0.00129769 | 0.86 |
| rs6444809 | Type 2 diabetes | Usual walking pace | 0.0544 | 0.0096 | 1.63E-08 | -0.00193099 | 0.00192698 | 0.32 |
| rs6459737 | Type 2 diabetes | Usual walking pace | -0.0583 | 0.0067 | 3.31E-18 | 0.00104173 | 0.00132669 | 0.43 |
| rs648795 | Type 2 diabetes | Usual walking pace | 0.0391 | 0.0065 | 1.71E-09 | -0.00020161 | 0.00128716 | 0.88 |
| rs6600191 | Type 2 diabetes | Usual walking pace | 0.0587 | 0.0085 | 4.47E-12 | -0.000770793 | 0.00164271 | 0.64 |
| rs6687271 | Type 2 diabetes | Usual walking pace | -0.0402 | 0.0073 | 4.14E-08 | 0.00171488 | 0.0014776 | 0.25 |
| rs672271 | Type 2 diabetes | Usual walking pace | -0.06 | 0.011 | 4.83E-08 | 0.00537794 | 0.00223511 | 0.016 |
| rs67232546 | Type 2 diabetes | Usual walking pace | 0.0531 | 0.008 | 2.51E-11 | -0.00321842 | 0.00155773 | 0.0389996 |
| rs6821438 | Type 2 diabetes | Usual walking pace | 0.0397 | 0.0064 | 5.10E-10 | -0.00133177 | 0.0012684 | 0.29 |
| rs6885132 | Type 2 diabetes | Usual walking pace | 0.077 | 0.011 | 2.49E-12 | -0.00197929 | 0.00214406 | 0.36 |
| rs6937438 | Type 2 diabetes | Usual walking pace | -0.0514 | 0.007 | 2.35E-13 | 0.00291455 | 0.00139145 | 0.0359998 |
| rs702634 | Type 2 diabetes | Usual walking pace | 0.0503 | 0.0069 | 3.37E-13 | 0.0024765 | 0.00137447 | 0.0719996 |
| rs703972 | Type 2 diabetes | Usual walking pace | -0.0698 | 0.0065 | 5.77E-27 | 0.0015425 | 0.00127014 | 0.22 |
| rs7178762 | Type 2 diabetes | Usual walking pace | -0.0387 | 0.0064 | 1.37E-09 | 0.00181316 | 0.00127462 | 0.15 |
| rs7240767 | Type 2 diabetes | Usual walking pace | -0.0372 | 0.0066 | 1.71E-08 | -0.0016185 | 0.00130124 | 0.21 |
| rs72802358 | Type 2 diabetes | Usual walking pace | -0.1135 | 0.0107 | 2.22E-26 | -0.000613484 | 0.00209709 | 0.77 |
| rs7313918 | Type 2 diabetes | Usual walking pace | -0.0559 | 0.0094 | 2.99E-09 | 0.00223474 | 0.00192542 | 0.25 |
| rs7325671 | Type 2 diabetes | Usual walking pace | 0.0545 | 0.0096 | 1.53E-08 | -0.00534345 | 0.00188402 | 0.00460002 |
| rs739846 | Type 2 diabetes | Usual walking pace | 0.0877 | 0.0119 | 2.02E-13 | -0.00278188 | 0.00239823 | 0.25 |
| rs745805 | Type 2 diabetes | Usual walking pace | 0.0632 | 0.0084 | 4.52E-14 | 0.00160243 | 0.00166324 | 0.34 |
| rs7568172 | Type 2 diabetes | Usual walking pace | -0.0827 | 0.0135 | 9.19E-10 | -0.00652846 | 0.00262265 | 0.0129999 |
| rs7572970 | Type 2 diabetes | Usual walking pace | -0.0447 | 0.0072 | 6.12E-10 | 0.00420105 | 0.0014091 | 0.00290001 |
| rs7633675 | Type 2 diabetes | Usual walking pace | -0.1085 | 0.0068 | 3.21E-57 | 0.00298323 | 0.0013616 | 0.0280001 |
| rs7669833 | Type 2 diabetes | Usual walking pace | -0.0572 | 0.007 | 3.52E-16 | 0.000202261 | 0.00140078 | 0.89 |
| rs76895963 | Type 2 diabetes | Usual walking pace | 0.4826 | 0.0275 | 9.12E-69 | -0.00520636 | 0.00490124 | 0.29 |
| rs7732130 | Type 2 diabetes | Usual walking pace | -0.0606 | 0.007 | 5.69E-18 | 0.000148427 | 0.00135975 | 0.91 |
| rs77864822 | Type 2 diabetes | Usual walking pace | 0.0753 | 0.0129 | 5.01E-09 | -0.00101639 | 0.0025961 | 0.7 |
| rs7966976 | Type 2 diabetes | Usual walking pace | -0.0751 | 0.0081 | 1.22E-20 | -0.00243569 | 0.00159231 | 0.13 |
| rs79920718 | Type 2 diabetes | Usual walking pace | 0.0389 | 0.0067 | 6.43E-09 | 0.00109011 | 0.00131951 | 0.41 |
| rs8008910 | Type 2 diabetes | Usual walking pace | 0.0554 | 0.0076 | 4.22E-13 | -0.00346626 | 0.00152401 | 0.0230001 |
| rs8071043 | Type 2 diabetes | Usual walking pace | -0.0523 | 0.0068 | 1.53E-14 | -7.48E-05 | 0.00135166 | 0.96 |
| rs8097210 | Type 2 diabetes | Usual walking pace | -0.0537 | 0.0072 | 1.06E-13 | -0.000117709 | 0.00143442 | 0.93 |
| rs8192675 | Type 2 diabetes | Usual walking pace | 0.0659 | 0.007 | 5.77E-21 | 0.00402344 | 0.0013946 | 0.00389996 |
| rs878521 | Type 2 diabetes | Usual walking pace | 0.0618 | 0.0074 | 9.31E-17 | -0.00402262 | 0.00145969 | 0.00589997 |
| rs9304665 | Type 2 diabetes | Usual walking pace | 0.0418 | 0.0076 | 4.53E-08 | -0.00342753 | 0.00149264 | 0.0219999 |
| rs9368222 | Type 2 diabetes | Usual walking pace | 0.1379 | 0.0071 | 1.42E-83 | -1.50E-05 | 0.00143778 | 0.99 |
| rs9379084 | Type 2 diabetes | Usual walking pace | -0.0994 | 0.0106 | 5.48E-21 | -0.00119038 | 0.00203971 | 0.56 |
| rs9563615 | Type 2 diabetes | Usual walking pace | 0.0408 | 0.0072 | 1.63E-08 | -0.00329711 | 0.00138973 | 0.0179999 |
| rs9665898 | Type 2 diabetes | Usual walking pace | -0.0525 | 0.0094 | 2.53E-08 | 0.000184303 | 0.00188257 | 0.92 |
| rs9873519 | Type 2 diabetes | Usual walking pace | 0.0371 | 0.0064 | 6.29E-09 | -0.0043256 | 0.00127014 | 0.000659994 |

**Supplementary Table 5.** Genome-wide significant SNPs for Fasting glucose

| **SNP** | **exposure** | **outcome** | **beta.exposure** | **se.exposure** | **pval.exposure** | **beta.outcome** | **se.outcome** | **pval.outcome** |
| --- | --- | --- | --- | --- | --- | --- | --- | --- |
| rs10305457 | Fasting glucose | Low hand grip strength | 0.0235 | 0.0032 | 1.20893E-14 | -0.0106 | 0.0125 | 0.3962 |
| rs10487796 | Fasting glucose | Low hand grip strength | -0.0261 | 0.0016 | 4.61849E-52 | 0.0024 | 0.0074 | 0.7418 |
| rs1057394 | Fasting glucose | Low hand grip strength | -0.0124 | 0.0018 | 1.91117E-12 | 0.0049 | 0.0075 | 0.5193 |
| rs10811660 | Fasting glucose | Low hand grip strength | -0.0223 | 0.0022 | 7.9378E-25 | -0.0094 | 0.0097 | 0.3332 |
| rs10830963 | Fasting glucose | Low hand grip strength | 0.0772 | 0.0019 | 1E-200 | -0.0075 | 0.0082 | 0.3648 |
| rs10838524 | Fasting glucose | Low hand grip strength | -0.0238 | 0.0016 | 1.55883E-40 | -0.0024 | 0.0074 | 0.749601 |
| rs10838693 | Fasting glucose | Low hand grip strength | 0.0177 | 0.0018 | 3.44191E-23 | -0.0104 | 0.0079 | 0.1853 |
| rs10974438 | Fasting glucose | Low hand grip strength | 0.0198 | 0.0017 | 9.84691E-31 | -0.0183 | 0.0077 | 0.0178299 |
| rs11603349 | Fasting glucose | Low hand grip strength | -0.0236 | 0.0022 | 3.11602E-25 | -0.0177 | 0.0102 | 0.0821599 |
| rs11610045 | Fasting glucose | Low hand grip strength | 0.0144 | 0.0019 | 3.25987E-13 | -0.0074 | 0.0074 | 0.3177 |
| rs11619319 | Fasting glucose | Low hand grip strength | 0.0173 | 0.002 | 3.41193E-20 | -0.0061 | 0.0088 | 0.488 |
| rs11708067 | Fasting glucose | Low hand grip strength | -0.0281 | 0.002 | 1.62592E-43 | 0.0158 | 0.0086 | 0.0646696 |
| rs12055786 | Fasting glucose | Low hand grip strength | 0.012 | 0.0017 | 1.166E-11 | -0.0032 | 0.0075 | 0.673101 |
| rs12541643 | Fasting glucose | Low hand grip strength | 0.0118 | 0.0019 | 4.51097E-09 | -0.0013 | 0.0074 | 0.8618 |
| rs1260326 | Fasting glucose | Low hand grip strength | 0.0282 | 0.0017 | 4.48126E-65 | -0.0062 | 0.0075 | 0.4111 |
| rs12784552 | Fasting glucose | Low hand grip strength | -0.0329 | 0.003 | 2.86286E-31 | -0.0103 | 0.0128 | 0.4234 |
| rs12888855 | Fasting glucose | Low hand grip strength | -0.0135 | 0.002 | 6.01866E-12 | -0.007 | 0.0088 | 0.4249 |
| rs12898997 | Fasting glucose | Low hand grip strength | -0.0098 | 0.0017 | 4.64098E-09 | 0.0017 | 0.0078 | 0.829 |
| rs157512 | Fasting glucose | Low hand grip strength | -0.0134 | 0.0021 | 5.426E-10 | -0.0064 | 0.0086 | 0.4554 |
| rs1604038 | Fasting glucose | Low hand grip strength | -0.0198 | 0.0018 | 4.46786E-28 | -0.0038 | 0.0081 | 0.636999 |
| rs16851397 | Fasting glucose | Low hand grip strength | -0.0327 | 0.0042 | 1.26009E-12 | -0.0248 | 0.0175 | 0.1553 |
| rs16913693 | Fasting glucose | Low hand grip strength | -0.0394 | 0.0049 | 2.81968E-16 | 0.0235 | 0.0229 | 0.304 |
| rs17168486 | Fasting glucose | Low hand grip strength | 0.028 | 0.0021 | 4.16965E-36 | -0.0215 | 0.0097 | 0.0269401 |
| rs17265513 | Fasting glucose | Low hand grip strength | 0.0158 | 0.0021 | 5.10152E-14 | -0.0014 | 0.0092 | 0.8785 |
| rs17270243 | Fasting glucose | Low hand grip strength | 0.0104 | 0.0021 | 3.61601E-08 | 0.0076 | 0.0086 | 0.3762 |
| rs17437560 | Fasting glucose | Low hand grip strength | -0.0175 | 0.0032 | 3.33096E-08 | 0.0061 | 0.0124 | 0.623499 |
| rs174583 | Fasting glucose | Low hand grip strength | -0.0168 | 0.0017 | 3.37132E-22 | -0.0062 | 0.0077 | 0.4216 |
| rs1820176 | Fasting glucose | Low hand grip strength | -0.0247 | 0.002 | 1.90502E-34 | -0.0051 | 0.0082 | 0.536 |
| rs189548 | Fasting glucose | Low hand grip strength | -0.0123 | 0.002 | 2.81099E-09 | 0.0178 | 0.0082 | 0.0306598 |
| rs194518 | Fasting glucose | Low hand grip strength | 0.0102 | 0.0018 | 8.75709E-09 | -0.0074 | 0.0074 | 0.3179 |
| rs2075423 | Fasting glucose | Low hand grip strength | -0.0161 | 0.0017 | 3.18273E-21 | 0.0027 | 0.0078 | 0.7294 |
| rs2238435 | Fasting glucose | Low hand grip strength | -0.0112 | 0.0019 | 3.82402E-09 | 0.0079 | 0.0076 | 0.2978 |
| rs2302593 | Fasting glucose | Low hand grip strength | -0.0106 | 0.0017 | 5.66905E-10 | 0.0088 | 0.0074 | 0.234 |
| rs2461385 | Fasting glucose | Low hand grip strength | -0.0217 | 0.0024 | 2.73023E-19 | -0.0035 | 0.0101 | 0.7296 |
| rs2595701 | Fasting glucose | Low hand grip strength | -0.0189 | 0.0021 | 4.47816E-19 | 0.0033 | 0.008 | 0.675899 |
| rs2657879 | Fasting glucose | Low hand grip strength | 0.0119 | 0.0022 | 7.33398E-09 | -0.0096 | 0.0095 | 0.3118 |
| rs2839671 | Fasting glucose | Low hand grip strength | -0.016 | 0.0022 | 8.37915E-14 | -0.0192 | 0.0096 | 0.0460299 |
| rs348330 | Fasting glucose | Low hand grip strength | -0.0122 | 0.002 | 3.03599E-10 | 0.0037 | 0.0077 | 0.6264 |
| rs35889227 | Fasting glucose | Low hand grip strength | -0.013 | 0.0019 | 3.36504E-10 | 0.01 | 0.0076 | 0.1901 |
| rs3778321 | Fasting glucose | Low hand grip strength | -0.0186 | 0.0021 | 3.15718E-17 | -0.0232 | 0.0097 | 0.0161599 |
| rs3829109 | Fasting glucose | Low hand grip strength | -0.0163 | 0.002 | 1.08693E-15 | -0.0089 | 0.0084 | 0.2843 |
| rs3842753 | Fasting glucose | Low hand grip strength | -0.0134 | 0.0022 | 2.84099E-09 | -0.0182 | 0.0082 | 0.0266698 |
| rs39713 | Fasting glucose | Low hand grip strength | -0.0169 | 0.0031 | 1.76701E-08 | 0.0109 | 0.0128 | 0.3934 |
| rs4760278 | Fasting glucose | Low hand grip strength | -0.011 | 0.002 | 3.33096E-08 | -0.006 | 0.0088 | 0.4975 |
| rs4862423 | Fasting glucose | Low hand grip strength | 0.0123 | 0.0019 | 4.44703E-10 | 0.0042 | 0.0076 | 0.5764 |
| rs507666 | Fasting glucose | Low hand grip strength | 0.0164 | 0.0021 | 6.99198E-17 | -0.0068 | 0.0094 | 0.4664 |
| rs537183 | Fasting glucose | Low hand grip strength | 0.0663 | 0.0017 | 1E-200 | -0.0079 | 0.0078 | 0.3056 |
| rs58925536 | Fasting glucose | Low hand grip strength | 0.0306 | 0.0053 | 5.82103E-09 | 0.0102 | 0.0202 | 0.6137 |
| rs6113722 | Fasting glucose | Low hand grip strength | -0.0424 | 0.0044 | 7.65949E-25 | 0.0047 | 0.0191 | 0.8045 |
| rs6489811 | Fasting glucose | Low hand grip strength | 0.011 | 0.0018 | 3.26701E-09 | -0.0142 | 0.0073 | 0.05243 |
| rs6538804 | Fasting glucose | Low hand grip strength | -0.0142 | 0.0019 | 9.41239E-14 | -0.007 | 0.0076 | 0.36 |
| rs6598541 | Fasting glucose | Low hand grip strength | -0.0114 | 0.0017 | 4.12382E-12 | -0.0092 | 0.0077 | 0.2348 |
| rs6662924 | Fasting glucose | Low hand grip strength | 0.0143 | 0.0023 | 3.34103E-10 | -0.0055 | 0.0097 | 0.5673 |
| rs6808574 | Fasting glucose | Low hand grip strength | 0.0127 | 0.0017 | 7.20941E-14 | 0.0144 | 0.0076 | 0.0574804 |
| rs7012637 | Fasting glucose | Low hand grip strength | -0.018 | 0.0017 | 9.74541E-25 | 0.0164 | 0.0074 | 0.02711 |
| rs7095788 | Fasting glucose | Low hand grip strength | -0.0106 | 0.0018 | 1.97601E-09 | 0.0094 | 0.0076 | 0.2204 |
| rs7163757 | Fasting glucose | Low hand grip strength | -0.0217 | 0.0016 | 2.64119E-36 | 0.0026 | 0.0074 | 0.7294 |
| rs7178572 | Fasting glucose | Low hand grip strength | 0.0121 | 0.0018 | 7.08598E-10 | 0.0204 | 0.0081 | 0.0122101 |
| rs7708285 | Fasting glucose | Low hand grip strength | -0.0133 | 0.0019 | 1.253E-09 | -0.0229 | 0.008 | 0.00428598 |
| rs77981966 | Fasting glucose | Low hand grip strength | -0.0246 | 0.0035 | 1.58016E-14 | -0.0403 | 0.014 | 0.00397796 |
| rs78132593 | Fasting glucose | Low hand grip strength | -0.0147 | 0.0022 | 2.59801E-10 | 0.0115 | 0.0089 | 0.1992 |
| rs7903146 | Fasting glucose | Low hand grip strength | 0.0259 | 0.0019 | 1.9948E-35 | 0.0115 | 0.0081 | 0.1563 |
| rs878521 | Fasting glucose | Low hand grip strength | 0.0549 | 0.002 | 2.6485E-174 | 0.0131 | 0.0084 | 0.1211 |
| rs896854 | Fasting glucose | Low hand grip strength | -0.0099 | 0.0016 | 5.61203E-09 | 0.003 | 0.0074 | 0.687 |
| rs9348441 | Fasting glucose | Low hand grip strength | 0.0176 | 0.0018 | 4.40251E-20 | -0.0016 | 0.0083 | 0.8513 |
| rs9650069 | Fasting glucose | Low hand grip strength | -0.0286 | 0.0018 | 8.30615E-58 | -0.0035 | 0.008 | 0.661601 |
| rs10305457 | Fasting glucose | ALM | 0.0235 | 0.0032 | 1.20893E-14 | -0.0069 | 0.0032 | 0.0315 |
| rs10487796 | Fasting glucose | ALM | -0.0261 | 0.0016 | 4.61849E-52 | 0.0058 | 0.0019 | 0.00215501 |
| rs1057394 | Fasting glucose | ALM | -0.0124 | 0.0018 | 1.91117E-12 | -0.0035 | 0.0019 | 0.0710003 |
| rs10811660 | Fasting glucose | ALM | -0.0223 | 0.0022 | 7.9378E-25 | 0.0009 | 0.0025 | 0.733 |
| rs10830963 | Fasting glucose | ALM | 0.0772 | 0.0019 | 1E-200 | 0.0049 | 0.0021 | 0.0194899 |
| rs10838524 | Fasting glucose | ALM | -0.0238 | 0.0016 | 1.55883E-40 | 0.0031 | 0.0019 | 0.1001 |
| rs10838693 | Fasting glucose | ALM | 0.0177 | 0.0018 | 3.44191E-23 | 0.0187 | 0.002 | 2.2532E-20 |
| rs10974438 | Fasting glucose | ALM | 0.0198 | 0.0017 | 9.84691E-31 | 0.0067 | 0.002 | 0.000767397 |
| rs11603349 | Fasting glucose | ALM | -0.0236 | 0.0022 | 3.11602E-25 | 0.0107 | 0.0026 | 4.36305E-05 |
| rs11610045 | Fasting glucose | ALM | 0.0144 | 0.0019 | 3.25987E-13 | -0.0031 | 0.0019 | 0.0967097 |
| rs11619319 | Fasting glucose | ALM | 0.0173 | 0.002 | 3.41193E-20 | -0.0051 | 0.0023 | 0.0247503 |
| rs11708067 | Fasting glucose | ALM | -0.0281 | 0.002 | 1.62592E-43 | -0.0125 | 0.0022 | 1.38599E-08 |
| rs12055786 | Fasting glucose | ALM | 0.012 | 0.0017 | 1.166E-11 | -0.0001 | 0.0019 | 0.9529 |
| rs12541643 | Fasting glucose | ALM | 0.0118 | 0.0019 | 4.51097E-09 | 0.0026 | 0.0019 | 0.1752 |
| rs1260326 | Fasting glucose | ALM | 0.0282 | 0.0017 | 4.48126E-65 | 0.0323 | 0.0019 | 6.15886E-64 |
| rs12784552 | Fasting glucose | ALM | -0.0329 | 0.003 | 2.86286E-31 | -0.0019 | 0.0033 | 0.5559 |
| rs12888855 | Fasting glucose | ALM | -0.0135 | 0.002 | 6.01866E-12 | -0.0107 | 0.0023 | 0.000002066 |
| rs12898997 | Fasting glucose | ALM | -0.0098 | 0.0017 | 4.64098E-09 | 0.0015 | 0.002 | 0.4591 |
| rs157512 | Fasting glucose | ALM | -0.0134 | 0.0021 | 5.426E-10 | -0.0151 | 0.0022 | 8.97635E-12 |
| rs1604038 | Fasting glucose | ALM | -0.0198 | 0.0018 | 4.46786E-28 | 0.0054 | 0.0021 | 0.00943909 |
| rs16851397 | Fasting glucose | ALM | -0.0327 | 0.0042 | 1.26009E-12 | 0.0399 | 0.0045 | 3.05492E-19 |
| rs16913693 | Fasting glucose | ALM | -0.0394 | 0.0049 | 2.81968E-16 | 0.0378 | 0.0061 | 4.48405E-10 |
| rs17168486 | Fasting glucose | ALM | 0.028 | 0.0021 | 4.16965E-36 | 0.002 | 0.0025 | 0.4221 |
| rs17265513 | Fasting glucose | ALM | 0.0158 | 0.0021 | 5.10152E-14 | -0.0123 | 0.0024 | 2.41702E-07 |
| rs17270243 | Fasting glucose | ALM | 0.0104 | 0.0021 | 3.61601E-08 | 0.0023 | 0.0022 | 0.3056 |
| rs17437560 | Fasting glucose | ALM | -0.0175 | 0.0032 | 3.33096E-08 | -0.001 | 0.0032 | 0.754899 |
| rs174583 | Fasting glucose | ALM | -0.0168 | 0.0017 | 3.37132E-22 | -0.0129 | 0.002 | 7.23769E-11 |
| rs1820176 | Fasting glucose | ALM | -0.0247 | 0.002 | 1.90502E-34 | 0.0169 | 0.0021 | 6.25749E-16 |
| rs189548 | Fasting glucose | ALM | -0.0123 | 0.002 | 2.81099E-09 | 0.0063 | 0.0021 | 0.00238699 |
| rs194518 | Fasting glucose | ALM | 0.0102 | 0.0018 | 8.75709E-09 | -0.001 | 0.0019 | 0.583001 |
| rs2075423 | Fasting glucose | ALM | -0.0161 | 0.0017 | 3.18273E-21 | 0.0018 | 0.002 | 0.369 |
| rs2238435 | Fasting glucose | ALM | -0.0112 | 0.0019 | 3.82402E-09 | 0.0142 | 0.002 | 3.02273E-13 |
| rs2302593 | Fasting glucose | ALM | -0.0106 | 0.0017 | 5.66905E-10 | -0.0008 | 0.0019 | 0.670199 |
| rs2461385 | Fasting glucose | ALM | -0.0217 | 0.0024 | 2.73023E-19 | 0.0045 | 0.0026 | 0.07993 |
| rs2595701 | Fasting glucose | ALM | -0.0189 | 0.0021 | 4.47816E-19 | 0.002 | 0.002 | 0.3168 |
| rs2657879 | Fasting glucose | ALM | 0.0119 | 0.0022 | 7.33398E-09 | -0.0067 | 0.0024 | 0.00583203 |
| rs2839671 | Fasting glucose | ALM | -0.016 | 0.0022 | 8.37915E-14 | 0.0002 | 0.0025 | 0.9358 |
| rs348330 | Fasting glucose | ALM | -0.0122 | 0.002 | 3.03599E-10 | 0.0057 | 0.0019 | 0.003498 |
| rs35889227 | Fasting glucose | ALM | -0.013 | 0.0019 | 3.36504E-10 | 0.0036 | 0.002 | 0.0659204 |
| rs3778321 | Fasting glucose | ALM | -0.0186 | 0.0021 | 3.15718E-17 | -0.0031 | 0.0025 | 0.2086 |
| rs3829109 | Fasting glucose | ALM | -0.0163 | 0.002 | 1.08693E-15 | 0.0041 | 0.0021 | 0.0531105 |
| rs3842753 | Fasting glucose | ALM | -0.0134 | 0.0022 | 2.84099E-09 | -0.0081 | 0.0021 | 0.0001148 |
| rs39713 | Fasting glucose | ALM | -0.0169 | 0.0031 | 1.76701E-08 | 0.011 | 0.0033 | 0.000802694 |
| rs4760278 | Fasting glucose | ALM | -0.011 | 0.002 | 3.33096E-08 | 0.0041 | 0.0023 | 0.0718092 |
| rs4862423 | Fasting glucose | ALM | 0.0123 | 0.0019 | 4.44703E-10 | -0.0003 | 0.0019 | 0.8757 |
| rs507666 | Fasting glucose | ALM | 0.0164 | 0.0021 | 6.99198E-17 | -0.0015 | 0.0024 | 0.5496 |
| rs537183 | Fasting glucose | ALM | 0.0663 | 0.0017 | 1E-200 | 0.0062 | 0.002 | 0.00150598 |
| rs58925536 | Fasting glucose | ALM | 0.0306 | 0.0053 | 5.82103E-09 | -0.0101 | 0.0052 | 0.0541502 |
| rs6113722 | Fasting glucose | ALM | -0.0424 | 0.0044 | 7.65949E-25 | 0.0185 | 0.005 | 0.000217 |
| rs6489811 | Fasting glucose | ALM | 0.011 | 0.0018 | 3.26701E-09 | 0.007 | 0.0019 | 0.000200701 |
| rs6538804 | Fasting glucose | ALM | -0.0142 | 0.0019 | 9.41239E-14 | -0.0006 | 0.0019 | 0.7769 |
| rs6598541 | Fasting glucose | ALM | -0.0114 | 0.0017 | 4.12382E-12 | -0.0015 | 0.002 | 0.4424 |
| rs6662924 | Fasting glucose | ALM | 0.0143 | 0.0023 | 3.34103E-10 | -0.0022 | 0.0025 | 0.3724 |
| rs6808574 | Fasting glucose | ALM | 0.0127 | 0.0017 | 7.20941E-14 | -0.0058 | 0.0019 | 0.00267701 |
| rs7012637 | Fasting glucose | ALM | -0.018 | 0.0017 | 9.74541E-25 | -0.0026 | 0.0019 | 0.1669 |
| rs7095788 | Fasting glucose | ALM | -0.0106 | 0.0018 | 1.97601E-09 | 0.0012 | 0.002 | 0.533801 |
| rs7163757 | Fasting glucose | ALM | -0.0217 | 0.0016 | 2.64119E-36 | -0.0167 | 0.0019 | 3.61576E-18 |
| rs7178572 | Fasting glucose | ALM | 0.0121 | 0.0018 | 7.08598E-10 | -0.007 | 0.0021 | 0.000807793 |
| rs7708285 | Fasting glucose | ALM | -0.0133 | 0.0019 | 1.253E-09 | 0.0054 | 0.002 | 0.00837298 |
| rs77981966 | Fasting glucose | ALM | -0.0246 | 0.0035 | 1.58016E-14 | -0.0155 | 0.0035 | 0.00001166 |
| rs78132593 | Fasting glucose | ALM | -0.0147 | 0.0022 | 2.59801E-10 | -0.0108 | 0.0023 | 2.21702E-06 |
| rs7903146 | Fasting glucose | ALM | 0.0259 | 0.0019 | 1.9948E-35 | 0.0027 | 0.0021 | 0.1998 |
| rs878521 | Fasting glucose | ALM | 0.0549 | 0.002 | 2.6485E-174 | -0.0075 | 0.0022 | 0.000538406 |
| rs896854 | Fasting glucose | ALM | -0.0099 | 0.0016 | 5.61203E-09 | 0.0061 | 0.0019 | 0.001199 |
| rs9348441 | Fasting glucose | ALM | 0.0176 | 0.0018 | 4.40251E-20 | -0.0048 | 0.0021 | 0.0254601 |
| rs9650069 | Fasting glucose | ALM | -0.0286 | 0.0018 | 8.30615E-58 | 0.0014 | 0.0021 | 0.4967 |
| rs10305457 | Fasting glucose | Usual walking pace | 0.0235 | 0.0032 | 1.20893E-14 | 0.00244281 | 0.00216389 | 0.26 |
| rs10487796 | Fasting glucose | Usual walking pace | -0.0261 | 0.0016 | 4.61849E-52 | -0.00144086 | 0.00127307 | 0.26 |
| rs10811660 | Fasting glucose | Usual walking pace | -0.0223 | 0.0022 | 7.9378E-25 | 0.000277014 | 0.00167288 | 0.87 |
| rs10830963 | Fasting glucose | Usual walking pace | 0.0772 | 0.0019 | 1E-200 | -0.00124929 | 0.0014156 | 0.38 |
| rs10838524 | Fasting glucose | Usual walking pace | -0.0238 | 0.0016 | 1.55883E-40 | 0.000982956 | 0.00127468 | 0.44 |
| rs10974438 | Fasting glucose | Usual walking pace | 0.0198 | 0.0017 | 9.84691E-31 | 0.00545424 | 0.00132689 | 3.89996E-05 |
| rs11603349 | Fasting glucose | Usual walking pace | -0.0236 | 0.0022 | 3.11602E-25 | -0.00525291 | 0.00174448 | 0.00259998 |
| rs11610045 | Fasting glucose | Usual walking pace | 0.0144 | 0.0019 | 3.25987E-13 | 0.000329729 | 0.0012648 | 0.79 |
| rs11619319 | Fasting glucose | Usual walking pace | 0.0173 | 0.002 | 3.41193E-20 | -0.00321799 | 0.00152495 | 0.0350002 |
| rs12055786 | Fasting glucose | Usual walking pace | 0.012 | 0.0017 | 1.166E-11 | 0.00256806 | 0.00129046 | 0.0470002 |
| rs12541643 | Fasting glucose | Usual walking pace | 0.0118 | 0.0019 | 4.51097E-09 | 0.000492161 | 0.0012774 | 0.7 |
| rs12784552 | Fasting glucose | Usual walking pace | -0.0329 | 0.003 | 2.86286E-31 | 0.00391982 | 0.00220436 | 0.0749998 |
| rs12888855 | Fasting glucose | Usual walking pace | -0.0135 | 0.002 | 6.01866E-12 | -0.000367033 | 0.00150826 | 0.81 |
| rs157512 | Fasting glucose | Usual walking pace | -0.0134 | 0.0021 | 5.426E-10 | 0.00187658 | 0.00148322 | 0.21 |
| rs1604038 | Fasting glucose | Usual walking pace | -0.0198 | 0.0018 | 4.46786E-28 | -0.00411613 | 0.0014003 | 0.00329997 |
| rs16913693 | Fasting glucose | Usual walking pace | -0.0394 | 0.0049 | 2.81968E-16 | 0.00690091 | 0.00405985 | 0.089 |
| rs17168486 | Fasting glucose | Usual walking pace | 0.028 | 0.0021 | 4.16965E-36 | -0.00113194 | 0.00167947 | 0.5 |
| rs17270243 | Fasting glucose | Usual walking pace | 0.0104 | 0.0021 | 3.61601E-08 | 0.0043899 | 0.00148388 | 0.00309999 |
| rs17437560 | Fasting glucose | Usual walking pace | -0.0175 | 0.0032 | 3.33096E-08 | -0.000794762 | 0.00215505 | 0.709999 |
| rs1820176 | Fasting glucose | Usual walking pace | -0.0247 | 0.002 | 1.90502E-34 | -0.00309244 | 0.00140454 | 0.0280001 |
| rs189548 | Fasting glucose | Usual walking pace | -0.0123 | 0.002 | 2.81099E-09 | 0.00135814 | 0.00140665 | 0.33 |
| rs2075423 | Fasting glucose | Usual walking pace | -0.0161 | 0.0017 | 3.18273E-21 | -0.00104979 | 0.00134256 | 0.43 |
| rs2302593 | Fasting glucose | Usual walking pace | -0.0106 | 0.0017 | 5.66905E-10 | 0.00336355 | 0.00126707 | 0.00790005 |
| rs2595701 | Fasting glucose | Usual walking pace | -0.0189 | 0.0021 | 4.47816E-19 | -0.000655542 | 0.0013624 | 0.630001 |
| rs2657879 | Fasting glucose | Usual walking pace | 0.0119 | 0.0022 | 7.33398E-09 | 0.00338028 | 0.0016353 | 0.0389996 |
| rs2839671 | Fasting glucose | Usual walking pace | -0.016 | 0.0022 | 8.37915E-14 | -0.00261776 | 0.0016584 | 0.11 |
| rs348330 | Fasting glucose | Usual walking pace | -0.0122 | 0.002 | 3.03599E-10 | 0.000661727 | 0.00131683 | 0.62 |
| rs35889227 | Fasting glucose | Usual walking pace | -0.013 | 0.0019 | 3.36504E-10 | -0.00246307 | 0.00131374 | 0.061 |
| rs3778321 | Fasting glucose | Usual walking pace | -0.0186 | 0.0021 | 3.15718E-17 | 0.000994617 | 0.00165769 | 0.55 |
| rs3842753 | Fasting glucose | Usual walking pace | -0.0134 | 0.0022 | 2.84099E-09 | -0.000903731 | 0.00140366 | 0.52 |
| rs4862423 | Fasting glucose | Usual walking pace | 0.0123 | 0.0019 | 4.44703E-10 | 0.00402604 | 0.00130143 | 0.002 |
| rs6113722 | Fasting glucose | Usual walking pace | -0.0424 | 0.0044 | 7.65949E-25 | 0.00394844 | 0.0033356 | 0.24 |
| rs6538804 | Fasting glucose | Usual walking pace | -0.0142 | 0.0019 | 9.41239E-14 | -0.000369743 | 0.00130788 | 0.780001 |
| rs6598541 | Fasting glucose | Usual walking pace | -0.0114 | 0.0017 | 4.12382E-12 | 0.00458278 | 0.00132507 | 0.000539995 |
| rs6662924 | Fasting glucose | Usual walking pace | 0.0143 | 0.0023 | 3.34103E-10 | 0.00168744 | 0.00166708 | 0.31 |
| rs6808574 | Fasting glucose | Usual walking pace | 0.0127 | 0.0017 | 7.20941E-14 | -0.000848336 | 0.00130327 | 0.52 |
| rs7163757 | Fasting glucose | Usual walking pace | -0.0217 | 0.0016 | 2.64119E-36 | -0.000453961 | 0.00128033 | 0.719999 |
| rs7178572 | Fasting glucose | Usual walking pace | 0.0121 | 0.0018 | 7.08598E-10 | -0.000718053 | 0.00139689 | 0.61 |
| rs7708285 | Fasting glucose | Usual walking pace | -0.0133 | 0.0019 | 1.253E-09 | 4.17453E-05 | 0.00136997 | 0.98 |
| rs7903146 | Fasting glucose | Usual walking pace | 0.0259 | 0.0019 | 1.9948E-35 | 0.000793195 | 0.0013927 | 0.57 |
| rs896854 | Fasting glucose | Usual walking pace | -0.0099 | 0.0016 | 5.61203E-09 | 0.00112763 | 0.00126635 | 0.37 |
| rs9650069 | Fasting glucose | Usual walking pace | -0.0286 | 0.0018 | 8.30615E-58 | 0.00372729 | 0.00138146 | 0.00700003 |

**Supplementary Table 6.** Genome-wide significant SNPs for Fasting insulin

| **SNP** | **exposure** | **outcome** | **beta.exposure** | **se.exposure** | **pval.exposure** | **beta.outcome** | **se.outcome** | **pval.outcome** |
| --- | --- | --- | --- | --- | --- | --- | --- | --- |
| rs10050393 | Fasting insulin | Low hand grip strength | -0.009 | 0.0019 | 4.84295E-08 | 0.0049 | 0.0074 | 0.5034 |
| rs10865959 | Fasting insulin | Low hand grip strength | 0.0138 | 0.0022 | 1.992E-08 | -0.0184 | 0.0079 | 0.0201099 |
| rs116141873 | Fasting insulin | Low hand grip strength | 0.0428 | 0.0059 | 1.42298E-11 | 0.0238 | 0.0176 | 0.1752 |
| rs11708067 | Fasting insulin | Low hand grip strength | 0.0135 | 0.0023 | 1.299E-09 | 0.0158 | 0.0086 | 0.0646696 |
| rs11727676 | Fasting insulin | Low hand grip strength | 0.0203 | 0.0039 | 2.89801E-08 | -0.0043 | 0.0125 | 0.731499 |
| rs118164457 | Fasting insulin | Low hand grip strength | 0.0345 | 0.0057 | 3.85798E-10 | -0.0056 | 0.0184 | 0.7617 |
| rs1206760 | Fasting insulin | Low hand grip strength | -0.0112 | 0.0019 | 8.82104E-10 | -0.0123 | 0.0074 | 0.0982992 |
| rs12454712 | Fasting insulin | Low hand grip strength | -0.0142 | 0.0025 | 1.77701E-09 | -0.0079 | 0.0077 | 0.3037 |
| rs1260326 | Fasting insulin | Low hand grip strength | 0.0231 | 0.0019 | 8.41783E-38 | -0.0062 | 0.0075 | 0.4111 |
| rs13258890 | Fasting insulin | Low hand grip strength | -0.0128 | 0.0025 | 2.76503E-08 | 0.0134 | 0.0085 | 0.115 |
| rs13389219 | Fasting insulin | Low hand grip strength | -0.0199 | 0.0019 | 5.83848E-28 | 0.0022 | 0.0075 | 0.7659 |
| rs1351394 | Fasting insulin | Low hand grip strength | 0.0111 | 0.0018 | 2.70801E-09 | 0.0281 | 0.0073 | 0.0001276 |
| rs1474696 | Fasting insulin | Low hand grip strength | 0.0147 | 0.0018 | 3.01926E-16 | -0.0161 | 0.0074 | 0.0292799 |
| rs17036126 | Fasting insulin | Low hand grip strength | 0.0209 | 0.003 | 1.279E-10 | 0.0143 | 0.0109 | 0.1917 |
| rs17331151 | Fasting insulin | Low hand grip strength | -0.0162 | 0.0031 | 1.52398E-08 | -0.0059 | 0.0116 | 0.61 |
| rs2108349 | Fasting insulin | Low hand grip strength | -0.0115 | 0.002 | 1.133E-08 | 0.0024 | 0.0079 | 0.7604 |
| rs2780215 | Fasting insulin | Low hand grip strength | -0.0392 | 0.0063 | 1.064E-09 | -0.0123 | 0.0173 | 0.4783 |
| rs2845885 | Fasting insulin | Low hand grip strength | -0.0204 | 0.0039 | 1.17999E-08 | 0.0213 | 0.0162 | 0.1876 |
| rs2943646 | Fasting insulin | Low hand grip strength | 0.025 | 0.0019 | 8.47227E-39 | -0.0032 | 0.0077 | 0.675601 |
| rs35000407 | Fasting insulin | Low hand grip strength | -0.0258 | 0.0028 | 1.50383E-21 | 0.0032 | 0.0113 | 0.774 |
| rs3775380 | Fasting insulin | Low hand grip strength | 0.0119 | 0.0018 | 1.47707E-11 | -0.0119 | 0.0074 | 0.1052 |
| rs459193 | Fasting insulin | Low hand grip strength | 0.0181 | 0.0021 | 1.12305E-18 | 0.0084 | 0.0085 | 0.3209 |
| rs4865796 | Fasting insulin | Low hand grip strength | 0.0165 | 0.002 | 7.32825E-17 | -0.003 | 0.008 | 0.703701 |
| rs5017305 | Fasting insulin | Low hand grip strength | -0.0137 | 0.0026 | 1.06699E-08 | 0.0098 | 0.0107 | 0.3607 |
| rs62271373 | Fasting insulin | Low hand grip strength | 0.0256 | 0.0048 | 1.59599E-08 | -0.0089 | 0.016 | 0.5755 |
| rs6487237 | Fasting insulin | Low hand grip strength | 0.0154 | 0.0026 | 4.68198E-09 | 0.0008 | 0.0092 | 0.9284 |
| rs6674544 | Fasting insulin | Low hand grip strength | 0.0177 | 0.002 | 6.97268E-21 | -0.0143 | 0.0082 | 0.0826399 |
| rs6855363 | Fasting insulin | Low hand grip strength | -0.0125 | 0.002 | 4.03896E-08 | 0.0008 | 0.0078 | 0.9203 |
| rs6905288 | Fasting insulin | Low hand grip strength | 0.0112 | 0.0019 | 7.74997E-09 | -0.0249 | 0.0075 | 0.000855796 |
| rs7012814 | Fasting insulin | Low hand grip strength | -0.0219 | 0.0019 | 8.34257E-30 | 0.016 | 0.0074 | 0.0309799 |
| rs7133378 | Fasting insulin | Low hand grip strength | -0.0127 | 0.002 | 5.99653E-11 | 0.0165 | 0.0079 | 0.0355697 |
| rs73013411 | Fasting insulin | Low hand grip strength | -0.018 | 0.0032 | 2.08401E-08 | -0.0114 | 0.0109 | 0.2948 |
| rs731839 | Fasting insulin | Low hand grip strength | -0.0121 | 0.0019 | 3.86456E-11 | 0.0056 | 0.0078 | 0.476 |
| rs75179845 | Fasting insulin | Low hand grip strength | 0.0216 | 0.0035 | 6.05062E-11 | -0.0108 | 0.0154 | 0.484 |
| rs7903146 | Fasting insulin | Low hand grip strength | -0.0116 | 0.0021 | 1.237E-09 | 0.0115 | 0.0081 | 0.1563 |
| rs860598 | Fasting insulin | Low hand grip strength | 0.0177 | 0.0025 | 6.8786E-12 | 0.0102 | 0.01 | 0.3075 |
| rs972283 | Fasting insulin | Low hand grip strength | 0.0105 | 0.0019 | 1.093E-08 | 0.0046 | 0.0074 | 0.53 |
| rs9884482 | Fasting insulin | Low hand grip strength | 0.0125 | 0.0019 | 2.87872E-11 | 0.0191 | 0.0076 | 0.01184 |
| rs10050393 | Fasting insulin | ALM | -0.009 | 0.0019 | 4.84295E-08 | -0.003 | 0.0019 | 0.1173 |
| rs10865959 | Fasting insulin | ALM | 0.0138 | 0.0022 | 1.992E-08 | -0.0065 | 0.002 | 0.001376 |
| rs116141873 | Fasting insulin | ALM | 0.0428 | 0.0059 | 1.42298E-11 | 0.033 | 0.0044 | 7.3215E-14 |
| rs11708067 | Fasting insulin | ALM | 0.0135 | 0.0023 | 1.299E-09 | -0.0125 | 0.0022 | 1.38599E-08 |
| rs11727676 | Fasting insulin | ALM | 0.0203 | 0.0039 | 2.89801E-08 | 0.0044 | 0.0032 | 0.1667 |
| rs118164457 | Fasting insulin | ALM | 0.0345 | 0.0057 | 3.85798E-10 | -0.0044 | 0.0047 | 0.3466 |
| rs1206760 | Fasting insulin | ALM | -0.0112 | 0.0019 | 8.82104E-10 | -0.0049 | 0.0019 | 0.0110701 |
| rs12454712 | Fasting insulin | ALM | -0.0142 | 0.0025 | 1.77701E-09 | 0.0085 | 0.002 | 0.0000137 |
| rs1260326 | Fasting insulin | ALM | 0.0231 | 0.0019 | 8.41783E-38 | 0.0323 | 0.0019 | 6.15886E-64 |
| rs13258890 | Fasting insulin | ALM | -0.0128 | 0.0025 | 2.76503E-08 | -0.0044 | 0.0022 | 0.0442497 |
| rs13389219 | Fasting insulin | ALM | -0.0199 | 0.0019 | 5.83848E-28 | -0.0198 | 0.0019 | 4.51128E-25 |
| rs1351394 | Fasting insulin | ALM | 0.0111 | 0.0018 | 2.70801E-09 | -0.0487 | 0.0019 | 3.3189E-147 |
| rs1474696 | Fasting insulin | ALM | 0.0147 | 0.0018 | 3.01926E-16 | -0.0046 | 0.0019 | 0.0154099 |
| rs17036126 | Fasting insulin | ALM | 0.0209 | 0.003 | 1.279E-10 | 0.0096 | 0.0028 | 0.000572598 |
| rs17331151 | Fasting insulin | ALM | -0.0162 | 0.0031 | 1.52398E-08 | 0.0292 | 0.003 | 7.47997E-23 |
| rs2108349 | Fasting insulin | ALM | -0.0115 | 0.002 | 1.133E-08 | 0.0022 | 0.002 | 0.2707 |
| rs2780215 | Fasting insulin | ALM | -0.0392 | 0.0063 | 1.064E-09 | 0.046 | 0.004 | 3.93731E-30 |
| rs2845885 | Fasting insulin | ALM | -0.0204 | 0.0039 | 1.17999E-08 | -0.0051 | 0.0041 | 0.2153 |
| rs2943646 | Fasting insulin | ALM | 0.025 | 0.0019 | 8.47227E-39 | 0.0233 | 0.002 | 1.68811E-32 |
| rs35000407 | Fasting insulin | ALM | -0.0258 | 0.0028 | 1.50383E-21 | -0.0372 | 0.0029 | 1.65691E-37 |
| rs3775380 | Fasting insulin | ALM | 0.0119 | 0.0018 | 1.47707E-11 | 0.0112 | 0.0019 | 3.73001E-09 |
| rs459193 | Fasting insulin | ALM | 0.0181 | 0.0021 | 1.12305E-18 | 0.0139 | 0.0022 | 1.196E-10 |
| rs4865796 | Fasting insulin | ALM | 0.0165 | 0.002 | 7.32825E-17 | -0.0003 | 0.002 | 0.8944 |
| rs5017305 | Fasting insulin | ALM | -0.0137 | 0.0026 | 1.06699E-08 | 0.032 | 0.0025 | 7.33838E-38 |
| rs62271373 | Fasting insulin | ALM | 0.0256 | 0.0048 | 1.59599E-08 | 0.023 | 0.004 | 1.21099E-08 |
| rs6487237 | Fasting insulin | ALM | 0.0154 | 0.0026 | 4.68198E-09 | 0.0082 | 0.0024 | 0.000513405 |
| rs6674544 | Fasting insulin | ALM | 0.0177 | 0.002 | 6.97268E-21 | 0.0198 | 0.0019 | 3.99945E-25 |
| rs6855363 | Fasting insulin | ALM | -0.0125 | 0.002 | 4.03896E-08 | -0.0108 | 0.002 | 8.89099E-08 |
| rs6905288 | Fasting insulin | ALM | 0.0112 | 0.0019 | 7.74997E-09 | -0.0034 | 0.0019 | 0.0727595 |
| rs7012814 | Fasting insulin | ALM | -0.0219 | 0.0019 | 8.34257E-30 | -0.0026 | 0.0019 | 0.1663 |
| rs7133378 | Fasting insulin | ALM | -0.0127 | 0.002 | 5.99653E-11 | -0.0202 | 0.002 | 2.36919E-23 |
| rs73013411 | Fasting insulin | ALM | -0.018 | 0.0032 | 2.08401E-08 | 0.0136 | 0.0028 | 1.21099E-06 |
| rs731839 | Fasting insulin | ALM | -0.0121 | 0.0019 | 3.86456E-11 | -0.0116 | 0.002 | 6.95505E-09 |
| rs75179845 | Fasting insulin | ALM | 0.0216 | 0.0035 | 6.05062E-11 | -0.029 | 0.004 | 3.47696E-13 |
| rs7903146 | Fasting insulin | ALM | -0.0116 | 0.0021 | 1.237E-09 | 0.0027 | 0.0021 | 0.1998 |
| rs860598 | Fasting insulin | ALM | 0.0177 | 0.0025 | 6.8786E-12 | -0.0103 | 0.0026 | 6.43102E-05 |
| rs972283 | Fasting insulin | ALM | 0.0105 | 0.0019 | 1.093E-08 | 0.0175 | 0.0019 | 2.09411E-20 |
| rs9884482 | Fasting insulin | ALM | 0.0125 | 0.0019 | 2.87872E-11 | 0.017 | 0.002 | 2.40326E-18 |
| rs10050393 | Fasting insulin | Usual walking pace | -0.009 | 0.0019 | 4.84295E-08 | -0.000938889 | 0.00127572 | 0.46 |
| rs10865959 | Fasting insulin | Usual walking pace | 0.0138 | 0.0022 | 1.992E-08 | -0.00569423 | 0.0013562 | 2.69998E-05 |
| rs116141873 | Fasting insulin | Usual walking pace | 0.0428 | 0.0059 | 1.42298E-11 | 0.00431891 | 0.00296961 | 0.15 |
| rs11708067 | Fasting insulin | Usual walking pace | 0.0135 | 0.0023 | 1.299E-09 | -0.00553168 | 0.00147589 | 0.000179999 |
| rs11727676 | Fasting insulin | Usual walking pace | 0.0203 | 0.0039 | 2.89801E-08 | -0.00211279 | 0.00214208 | 0.32 |
| rs118164457 | Fasting insulin | Usual walking pace | 0.0345 | 0.0057 | 3.85798E-10 | -0.00380343 | 0.00314878 | 0.23 |
| rs1206760 | Fasting insulin | Usual walking pace | -0.0112 | 0.0019 | 8.82104E-10 | -0.0012319 | 0.00128194 | 0.34 |
| rs12454712 | Fasting insulin | Usual walking pace | -0.0142 | 0.0025 | 1.77701E-09 | -0.00259473 | 0.00130585 | 0.0470002 |
| rs1260326 | Fasting insulin | Usual walking pace | 0.0231 | 0.0019 | 8.41783E-38 | 0.000938332 | 0.00129237 | 0.47 |
| rs13258890 | Fasting insulin | Usual walking pace | -0.0128 | 0.0025 | 2.76503E-08 | -0.00193937 | 0.001474 | 0.19 |
| rs13389219 | Fasting insulin | Usual walking pace | -0.0199 | 0.0019 | 5.83848E-28 | -0.000584529 | 0.00129529 | 0.649999 |
| rs1351394 | Fasting insulin | Usual walking pace | 0.0111 | 0.0018 | 2.70801E-09 | -0.00449067 | 0.0012659 | 0.000389996 |
| rs1474696 | Fasting insulin | Usual walking pace | 0.0147 | 0.0018 | 3.01926E-16 | -0.00132544 | 0.00126625 | 0.3 |
| rs17036126 | Fasting insulin | Usual walking pace | 0.0209 | 0.003 | 1.279E-10 | -0.00272191 | 0.00188129 | 0.15 |
| rs17331151 | Fasting insulin | Usual walking pace | -0.0162 | 0.0031 | 1.52398E-08 | -0.00337033 | 0.00199035 | 0.0899995 |
| rs2108349 | Fasting insulin | Usual walking pace | -0.0115 | 0.002 | 1.133E-08 | -0.000618953 | 0.00135947 | 0.649999 |
| rs2845885 | Fasting insulin | Usual walking pace | -0.0204 | 0.0039 | 1.17999E-08 | -0.000645123 | 0.00276414 | 0.82 |
| rs2943646 | Fasting insulin | Usual walking pace | 0.025 | 0.0019 | 8.47227E-39 | -0.000638483 | 0.00132269 | 0.630001 |
| rs35000407 | Fasting insulin | Usual walking pace | -0.0258 | 0.0028 | 1.50383E-21 | -0.00599201 | 0.00195535 | 0.00219999 |
| rs3775380 | Fasting insulin | Usual walking pace | 0.0119 | 0.0018 | 1.47707E-11 | 0.00557653 | 0.00126822 | 1.09999E-05 |
| rs459193 | Fasting insulin | Usual walking pace | 0.0181 | 0.0021 | 1.12305E-18 | -0.0013088 | 0.00145196 | 0.37 |
| rs4865796 | Fasting insulin | Usual walking pace | 0.0165 | 0.002 | 7.32825E-17 | 0.0024118 | 0.00137178 | 0.0790005 |
| rs5017305 | Fasting insulin | Usual walking pace | -0.0137 | 0.0026 | 1.06699E-08 | -0.0084259 | 0.0016766 | 0.0000005 |
| rs62271373 | Fasting insulin | Usual walking pace | 0.0256 | 0.0048 | 1.59599E-08 | -0.00233815 | 0.00271506 | 0.39 |
| rs6487237 | Fasting insulin | Usual walking pace | 0.0154 | 0.0026 | 4.68198E-09 | -0.000529211 | 0.00158083 | 0.74 |
| rs6674544 | Fasting insulin | Usual walking pace | 0.0177 | 0.002 | 6.97268E-21 | 0.00463019 | 0.0012857 | 0.00032 |
| rs6855363 | Fasting insulin | Usual walking pace | -0.0125 | 0.002 | 4.03896E-08 | -0.000971285 | 0.00135159 | 0.47 |
| rs6905288 | Fasting insulin | Usual walking pace | 0.0112 | 0.0019 | 7.74997E-09 | 0.00164337 | 0.00127716 | 0.2 |
| rs7012814 | Fasting insulin | Usual walking pace | -0.0219 | 0.0019 | 8.34257E-30 | -0.00355884 | 0.00127662 | 0.00530005 |
| rs7133378 | Fasting insulin | Usual walking pace | -0.0127 | 0.002 | 5.99653E-11 | -0.00401545 | 0.00135998 | 0.0032 |
| rs73013411 | Fasting insulin | Usual walking pace | -0.018 | 0.0032 | 2.08401E-08 | -0.00499205 | 0.00187939 | 0.00790005 |
| rs731839 | Fasting insulin | Usual walking pace | -0.0121 | 0.0019 | 3.86456E-11 | -0.0041974 | 0.00134224 | 0.00179999 |
| rs75179845 | Fasting insulin | Usual walking pace | 0.0216 | 0.0035 | 6.05062E-11 | 0.000338667 | 0.0026632 | 0.9 |
| rs7903146 | Fasting insulin | Usual walking pace | -0.0116 | 0.0021 | 1.237E-09 | 0.000793195 | 0.0013927 | 0.57 |
| rs860598 | Fasting insulin | Usual walking pace | 0.0177 | 0.0025 | 6.8786E-12 | -0.00181143 | 0.00172829 | 0.29 |
| rs972283 | Fasting insulin | Usual walking pace | 0.0105 | 0.0019 | 1.093E-08 | 0.00157984 | 0.00126438 | 0.21 |
| rs9884482 | Fasting insulin | Usual walking pace | 0.0125 | 0.0019 | 2.87872E-11 | 0.00375564 | 0.00130892 | 0.00409996 |

**Supplementary Table 7.** Genome-wide significant SNPs for HbA1c

| **SNP** | **exposure** | **outcome** | **beta.exposure** | **se.exposure** | **pval.exposure** | **beta.outcome** | **se.outcome** | **pval.outcome** |
| --- | --- | --- | --- | --- | --- | --- | --- | --- |
| rs10100688 | HbA1c | Low hand grip strength | 0.0285 | 0.0057 | 4.83E-07 | 0.0033 | 0.0105 | 0.755899 |
| rs1046896 | HbA1c | Low hand grip strength | 0.0346 | 0.0032 | 1.58E-26 | 0.0181 | 0.0079 | 0.0222802 |
| rs10806742 | HbA1c | Low hand grip strength | -0.0215 | 0.0047 | 4.75E-06 | 0.0021 | 0.0077 | 0.779701 |
| rs11231694 | HbA1c | Low hand grip strength | 0.035 | 0.0076 | 3.95E-06 | -0.0233 | 0.0161 | 0.1485 |
| rs11964178 | HbA1c | Low hand grip strength | -0.0168 | 0.0034 | 8.79E-07 | -0.0026 | 0.0074 | 0.720701 |
| rs12580246 | HbA1c | Low hand grip strength | 0.0284 | 0.0061 | 0.000003755 | -0.0057 | 0.0135 | 0.671699 |
| rs12819124 | HbA1c | Low hand grip strength | -0.0161 | 0.0034 | 1.83E-06 | -0.0209 | 0.0074 | 0.00445995 |
| rs1387153 | HbA1c | Low hand grip strength | 0.0258 | 0.0039 | 3.96E-11 | -0.0027 | 0.0081 | 0.7415 |
| rs16926246 | HbA1c | Low hand grip strength | -0.089 | 0.0057 | 3.11E-54 | 0.011 | 0.011 | 0.3165 |
| rs17533945 | HbA1c | Low hand grip strength | 0.0179 | 0.0038 | 1.93E-06 | 0.0103 | 0.0076 | 0.1739 |
| rs17789266 | HbA1c | Low hand grip strength | 0.023 | 0.0047 | 8.14E-07 | -0.0235 | 0.0101 | 0.0203002 |
| rs1789891 | HbA1c | Low hand grip strength | -0.0207 | 0.0045 | 4.67E-06 | 0.0059 | 0.0098 | 0.545801 |
| rs1799884 | HbA1c | Low hand grip strength | 0.038 | 0.0041 | 1.45E-20 | 0.0072 | 0.0096 | 0.452101 |
| rs1800562 | HbA1c | Low hand grip strength | -0.0636 | 0.0069 | 2.59E-20 | 0.0338 | 0.014 | 0.0161901 |
| rs2779116 | HbA1c | Low hand grip strength | 0.0237 | 0.004 | 2.75E-09 | 0.0079 | 0.0083 | 0.3432 |
| rs3782123 | HbA1c | Low hand grip strength | -0.0201 | 0.0042 | 1.67E-06 | 0.0038 | 0.0083 | 0.6483 |
| rs4737009 | HbA1c | Low hand grip strength | 0.0269 | 0.0039 | 6.12E-12 | -0.0012 | 0.0087 | 0.8877 |
| rs4844390 | HbA1c | Low hand grip strength | -0.0204 | 0.0041 | 6.90E-07 | -0.0068 | 0.0088 | 0.4447 |
| rs552976 | HbA1c | Low hand grip strength | 0.029 | 0.0034 | 8.16E-18 | -0.0021 | 0.0077 | 0.7902 |
| rs6453220 | HbA1c | Low hand grip strength | 0.0456 | 0.0099 | 4.19E-06 | -0.0035 | 0.0221 | 0.8748 |
| rs6474359 | HbA1c | Low hand grip strength | -0.0601 | 0.0105 | 1.18E-08 | -0.0184 | 0.0196 | 0.3489 |
| rs6844670 | HbA1c | Low hand grip strength | 0.0203 | 0.0039 | 2.12E-07 | 0.0038 | 0.0077 | 0.619001 |
| rs7355559 | HbA1c | Low hand grip strength | -0.0241 | 0.0052 | 3.86E-06 | -0.0165 | 0.011 | 0.1328 |
| rs7644261 | HbA1c | Low hand grip strength | -0.0179 | 0.0037 | 1.46E-06 | -0.0042 | 0.0081 | 0.6047 |
| rs7805661 | HbA1c | Low hand grip strength | 0.0366 | 0.0079 | 4.09E-06 | 0.0077 | 0.0169 | 0.6503 |
| rs7998202 | HbA1c | Low hand grip strength | 0.0307 | 0.0053 | 5.24E-09 | 0.0114 | 0.0106 | 0.2794 |
| rs837763 | HbA1c | Low hand grip strength | 0.0245 | 0.0048 | 2.70E-07 | 0.0169 | 0.0074 | 0.0229699 |
| rs855791 | HbA1c | Low hand grip strength | -0.0271 | 0.0036 | 2.74E-14 | 0.0001 | 0.0075 | 0.9898 |
| rs9318651 | HbA1c | Low hand grip strength | 0.0367 | 0.0079 | 0.00000315 | -0.0123 | 0.0076 | 0.1073 |
| rs9354939 | HbA1c | Low hand grip strength | -0.0163 | 0.0035 | 3.10E-06 | 0.0049 | 0.0075 | 0.5109 |
| rs10100688 | HbA1c | ALM | 0.0285 | 0.0057 | 4.82603E-07 | 0.0051 | 0.0027 | 0.0581795 |
| rs1046896 | HbA1c | ALM | 0.0346 | 0.0032 | 1.57616E-26 | 0.0043 | 0.002 | 0.0330301 |
| rs10806742 | HbA1c | ALM | -0.0215 | 0.0047 | 4.75401E-06 | -0.0006 | 0.002 | 0.777 |
| rs11231694 | HbA1c | ALM | 0.035 | 0.0076 | 3.95103E-06 | 0.0026 | 0.0042 | 0.527499 |
| rs11964178 | HbA1c | ALM | -0.0168 | 0.0034 | 8.78598E-07 | 0.0012 | 0.0019 | 0.541101 |
| rs12580246 | HbA1c | ALM | 0.0284 | 0.0061 | 0.000003755 | 0.0031 | 0.0035 | 0.3699 |
| rs12819124 | HbA1c | ALM | -0.0161 | 0.0034 | 1.83101E-06 | 0.0079 | 0.0019 | 2.70097E-05 |
| rs1387153 | HbA1c | ALM | 0.0258 | 0.0039 | 3.96096E-11 | 0.0036 | 0.0021 | 0.0807291 |
| rs16926246 | HbA1c | ALM | -0.089 | 0.0057 | 3.11028E-54 | -0.0066 | 0.0028 | 0.0184098 |
| rs17533945 | HbA1c | ALM | 0.0179 | 0.0038 | 1.93299E-06 | -0.0074 | 0.002 | 0.000171799 |
| rs17789266 | HbA1c | ALM | 0.023 | 0.0047 | 8.13598E-07 | 0.0059 | 0.0026 | 0.0223398 |
| rs1789891 | HbA1c | ALM | -0.0207 | 0.0045 | 4.67498E-06 | 0.0037 | 0.0025 | 0.1392 |
| rs1799884 | HbA1c | ALM | 0.038 | 0.0041 | 1.45111E-20 | -0.0047 | 0.0025 | 0.0577205 |
| rs1800562 | HbA1c | ALM | -0.0636 | 0.0069 | 2.59298E-20 | 0.0153 | 0.0035 | 0.00001456 |
| rs2723517 | HbA1c | ALM | -0.0159 | 0.0035 | 4.01698E-06 | 0.0102 | 0.0019 | 7.92994E-08 |
| rs2779116 | HbA1c | ALM | 0.0237 | 0.004 | 2.751E-09 | 0.0008 | 0.0021 | 0.6895 |
| rs3782123 | HbA1c | ALM | -0.0201 | 0.0042 | 1.67201E-06 | 0.0011 | 0.0021 | 0.6076 |
| rs4737009 | HbA1c | ALM | 0.0269 | 0.0039 | 6.11505E-12 | -0.0012 | 0.0022 | 0.5858 |
| rs4844390 | HbA1c | ALM | -0.0204 | 0.0041 | 6.89906E-07 | 0.0144 | 0.0023 | 1.74301E-10 |
| rs552976 | HbA1c | ALM | 0.029 | 0.0034 | 8.15643E-18 | 0.0055 | 0.002 | 0.00517595 |
| rs6453220 | HbA1c | ALM | 0.0456 | 0.0099 | 4.18601E-06 | 0.0013 | 0.0059 | 0.818 |
| rs6474359 | HbA1c | ALM | -0.0601 | 0.0105 | 1.17801E-08 | -0.0054 | 0.005 | 0.2727 |
| rs6844670 | HbA1c | ALM | 0.0203 | 0.0039 | 2.123E-07 | -0.0037 | 0.002 | 0.0629202 |
| rs7355559 | HbA1c | ALM | -0.0241 | 0.0052 | 3.86198E-06 | 0.0343 | 0.0028 | 3.76184E-35 |
| rs7644261 | HbA1c | ALM | -0.0179 | 0.0037 | 1.45801E-06 | 0.0055 | 0.0021 | 0.00796893 |
| rs7805661 | HbA1c | ALM | 0.0366 | 0.0079 | 4.08997E-06 | 0.0127 | 0.0043 | 0.00340997 |
| rs7998202 | HbA1c | ALM | 0.0307 | 0.0053 | 5.23504E-09 | 0.0025 | 0.0027 | 0.3505 |
| rs837763 | HbA1c | ALM | 0.0245 | 0.0048 | 2.696E-07 | -0.0145 | 0.0019 | 2.89068E-14 |
| rs855791 | HbA1c | ALM | -0.0271 | 0.0036 | 2.74221E-14 | -0.0016 | 0.0019 | 0.4114 |
| rs9318651 | HbA1c | ALM | 0.0367 | 0.0079 | 0.00000315 | -0.0018 | 0.002 | 0.3581 |
| rs9354939 | HbA1c | ALM | -0.0163 | 0.0035 | 3.09999E-06 | -0.0023 | 0.0019 | 0.2268 |
| rs10100688 | HbA1c | Usual walking pace | 0.0285 | 0.0057 | 4.82603E-07 | -0.00104838 | 0.00181371 | 0.56 |
| rs1046896 | HbA1c | Usual walking pace | 0.0346 | 0.0032 | 1.57616E-26 | 0.000726068 | 0.00136432 | 0.59 |
| rs10806742 | HbA1c | Usual walking pace | -0.0215 | 0.0047 | 4.75401E-06 | -0.000314708 | 0.00132338 | 0.81 |
| rs11231694 | HbA1c | Usual walking pace | 0.035 | 0.0076 | 3.95103E-06 | 0.000946005 | 0.00277719 | 0.73 |
| rs11964178 | HbA1c | Usual walking pace | -0.0168 | 0.0034 | 8.78598E-07 | 0.00286965 | 0.00127879 | 0.025 |
| rs12580246 | HbA1c | Usual walking pace | 0.0284 | 0.0061 | 0.000003755 | 7.31666E-05 | 0.00232279 | 0.97 |
| rs12819124 | HbA1c | Usual walking pace | -0.0161 | 0.0034 | 1.83101E-06 | 0.00200242 | 0.00127031 | 0.11 |
| rs1387153 | HbA1c | Usual walking pace | 0.0258 | 0.0039 | 3.96096E-11 | -0.00230754 | 0.00139294 | 0.0980009 |
| rs16926246 | HbA1c | Usual walking pace | -0.089 | 0.0057 | 3.11028E-54 | 0.00132679 | 0.00188057 | 0.48 |
| rs17533945 | HbA1c | Usual walking pace | 0.0179 | 0.0038 | 1.93299E-06 | 0.00260244 | 0.00130778 | 0.0470002 |
| rs17789266 | HbA1c | Usual walking pace | 0.023 | 0.0047 | 8.13598E-07 | 0.00350587 | 0.00174228 | 0.0439997 |
| rs1789891 | HbA1c | Usual walking pace | -0.0207 | 0.0045 | 4.67498E-06 | -0.00190494 | 0.00167381 | 0.26 |
| rs1799884 | HbA1c | Usual walking pace | 0.038 | 0.0041 | 1.45111E-20 | -0.00161553 | 0.00165298 | 0.33 |
| rs1800562 | HbA1c | Usual walking pace | -0.0636 | 0.0069 | 2.59298E-20 | -0.00323418 | 0.00237219 | 0.17 |
| rs2723517 | HbA1c | Usual walking pace | -0.0159 | 0.0035 | 4.01698E-06 | 0.000428326 | 0.0012697 | 0.74 |
| rs2779116 | HbA1c | Usual walking pace | 0.0237 | 0.004 | 2.751E-09 | -0.00118637 | 0.0014304 | 0.41 |
| rs3782123 | HbA1c | Usual walking pace | -0.0201 | 0.0042 | 1.67201E-06 | -0.000424619 | 0.00143613 | 0.77 |
| rs4737009 | HbA1c | Usual walking pace | 0.0269 | 0.0039 | 6.11505E-12 | 0.00268712 | 0.00148865 | 0.0710003 |
| rs4844390 | HbA1c | Usual walking pace | -0.0204 | 0.0041 | 6.89906E-07 | -0.00162516 | 0.00152249 | 0.29 |
| rs552976 | HbA1c | Usual walking pace | 0.029 | 0.0034 | 8.15643E-18 | -0.000158034 | 0.00132451 | 0.91 |
| rs6453220 | HbA1c | Usual walking pace | 0.0456 | 0.0099 | 4.18601E-06 | -0.00282265 | 0.00390597 | 0.47 |
| rs6474359 | HbA1c | Usual walking pace | -0.0601 | 0.0105 | 1.17801E-08 | -0.00161437 | 0.00332106 | 0.630001 |
| rs6844670 | HbA1c | Usual walking pace | 0.0203 | 0.0039 | 2.123E-07 | -0.000851889 | 0.00133276 | 0.52 |
| rs7355559 | HbA1c | Usual walking pace | -0.0241 | 0.0052 | 3.86198E-06 | 0.00272165 | 0.00186974 | 0.15 |
| rs7644261 | HbA1c | Usual walking pace | -0.0179 | 0.0037 | 1.45801E-06 | -0.00406983 | 0.00140146 | 0.00369999 |
| rs7805661 | HbA1c | Usual walking pace | 0.0366 | 0.0079 | 4.08997E-06 | 0.00251146 | 0.00289841 | 0.39 |
| rs7998202 | HbA1c | Usual walking pace | 0.0307 | 0.0053 | 5.23504E-09 | 0.00231932 | 0.0018127 | 0.2 |
| rs837763 | HbA1c | Usual walking pace | 0.0245 | 0.0048 | 2.696E-07 | -0.00438222 | 0.00127324 | 0.000580003 |
| rs855791 | HbA1c | Usual walking pace | -0.0271 | 0.0036 | 2.74221E-14 | 0.00188029 | 0.00128052 | 0.14 |
| rs9318651 | HbA1c | Usual walking pace | 0.0367 | 0.0079 | 0.00000315 | -0.00355713 | 0.00131593 | 0.00690001 |
| rs9354939 | HbA1c | Usual walking pace | -0.0163 | 0.0035 | 3.09999E-06 | -0.00321163 | 0.00129069 | 0.0129999 |

**Supplementary Table 8.** Genome-wide significant SNPs for Two-hour glucose challenge

| **SNP** | **exposure** | **outcome** | **beta.exposure** | **se.exposure** | **pval.exposure** | **beta.outcome** | **se.outcome** | **pval.outcome** |
| --- | --- | --- | --- | --- | --- | --- | --- | --- |
| rs10037968 | Two-hour glucose challenge | Low hand grip strength | 0.19 | 3.31E-07 | 5 | 0.031 | 0.0129 | 0.0160199 |
| rs10423928 | Two-hour glucose challenge | Low hand grip strength | 0.15 | 3.33E-06 | 19 | 0.0131 | 0.0092 | 0.1566 |
| rs12243326 | Two-hour glucose challenge | Low hand grip strength | 0.13 | 1.20E-09 | 10 | 0.0093 | 0.0082 | 0.2546 |
| rs1360245 | Two-hour glucose challenge | Low hand grip strength | 0.1 | 1.92E-07 | 10 | -0.0095 | 0.0078 | 0.2214 |
| rs17205365 | Two-hour glucose challenge | Low hand grip strength | 0.11 | 5.21E-07 | 15 | 0.0093 | 0.0081 | 0.2497 |
| rs309795 | Two-hour glucose challenge | Low hand grip strength | 0.092 | 0.000002216 | 4 | -0.0051 | 0.0075 | 0.4975 |
| rs780094 | Two-hour glucose challenge | Low hand grip strength | -0.091 | 0.000001447 | 2 | -0.0076 | 0.0076 | 0.3127 |
| rs10037968 | Two-hour glucose challenge | ALM | 0.19 | 3.30803E-07 | 5 | -0.0163 | 0.0033 | 9.17403E-07 |
| rs10423928 | Two-hour glucose challenge | ALM | 0.15 | 3.33403E-06 | 19 | 0.0013 | 0.0024 | 0.5937 |
| rs12243326 | Two-hour glucose challenge | ALM | 0.13 | 1.19501E-09 | 10 | 0.0023 | 0.0021 | 0.2814 |
| rs1360245 | Two-hour glucose challenge | ALM | 0.1 | 1.91999E-07 | 10 | 0.0006 | 0.002 | 0.754899 |
| rs17205365 | Two-hour glucose challenge | ALM | 0.11 | 5.21195E-07 | 15 | -0.0222 | 0.0021 | 1.56207E-26 |
| rs309795 | Two-hour glucose challenge | ALM | 0.092 | 0.000002216 | 4 | -0.0015 | 0.0019 | 0.4379 |
| rs780094 | Two-hour glucose challenge | ALM | -0.091 | 0.000001447 | 2 | 0.0308 | 0.0019 | 1.04809E-57 |
| rs10037968 | Two-hour glucose challenge | Usual walking pace | 0.19 | 3.30803E-07 | 5 | -0.0027847 | 0.00222518 | 0.21 |
| rs10423928 | Two-hour glucose challenge | Usual walking pace | 0.15 | 3.33403E-06 | 19 | 0.00779329 | 0.00159666 | 1.09999E-06 |
| rs12243326 | Two-hour glucose challenge | Usual walking pace | 0.13 | 1.19501E-09 | 10 | 0.000882419 | 0.00140435 | 0.53 |
| rs1360245 | Two-hour glucose challenge | Usual walking pace | 0.1 | 1.91999E-07 | 10 | -0.000227503 | 0.00134013 | 0.87 |
| rs17205365 | Two-hour glucose challenge | Usual walking pace | 0.11 | 5.21195E-07 | 15 | -0.00134111 | 0.00139222 | 0.34 |
| rs309795 | Two-hour glucose challenge | Usual walking pace | 0.092 | 0.000002216 | 4 | 0.000253461 | 0.00128552 | 0.84 |
| rs780094 | Two-hour glucose challenge | Usual walking pace | -0.091 | 0.000001447 | 2 | 0.000878277 | 0.00129879 | 0.5 |
